# Supplementary material for: The Effect of Antioxidant Polyphenol Supplementation on Cardiometabolic Risk Factors: A Systematic Review and Meta-Analysis
Source: Nutrients. 2024 Dec 5;16(23):4206. doi: 10.3390/nu16234206 (PMC11644654; doi:10.3390/nu16234206)
Supplement: Supplementary file 1 [file nutrients-16-04206-s001.zip › nutrients-3353859-supplementary.pdf]

Contents of Supplemental Materials

APPENDIX 1 CHARACTERISTICS OF THE PUBLICATIONS INCLUDED IN THE META-ANALYSIS 1

APPENDIX 2 FOREST PLOT 28

APPENDIX 3 RISK OF BIAS 78

APPENDIX 4 FUNNEL PLOT 90

APPENDIX 5 GRADE 140

APPENDIX 6 RESULTS OF SENSITIVITY ANALYSIS OF THE SYSTEMATIC REMOVAL OF EACH STUDY 180

Appendix 1 - Characteristics of the publications included in the meta-analysis

Table S1-1. Characteristics of the publications included in the meta-analysis (Anthocyanin).

Article(first author, year)	Location	Study design	Health status	Gender	Intervention duration	Intervention substance	Comparison	Age, year	n (Ctrl, Inter)	Extracted outcome(s)
Bakuradze 2019	Germany	R	healthy	M	8w	205.5mg anthocyanin	placebo	24±3	57(30,27)	HDL-C, LDL-C, TG, TC
Basu 2018	USA	R, DB, C	obesity	M/F	12w	154mg anthocyanin	placebo	57±3	34(17,17)	Insulin
Basu(1) 2010	USA	R, SB	metabolic syndrome	M/F	8w	742mg anthocyanin	placebo	51.5±3	48(25,23)	SBP, DBP, HDL-C, LDL-C, TG, TC, FBS, HbA1c
Basu(1) 2014	USA	R	obesity	M/F	12w	78mg anthocyanin	placebo	50±10	30(15,15)	SBP, DBP, HDL-C, LDL-C, TC, FBS, Insulin, HbA1c
Basu(2) 2010	USA	R	metabolic syndrome	M/F	8w	154mg anthocyanin	placebo	45±3	27(12,15)	SBP, DBP, HDL-C, LDL-C, TG, TC, FBS
Basu(2) 2014	USA	R	obesity	M/F	12w	155mg anthocyanin	placebo	49±11	30(15,15)	SBP, DBP, HDL-C, LDL-C, TC, FBS, Insulin, HbA2c
Curtis 2009	UK	R, DB	healthy	F	12w	500mg anthocyanin	placebo	58.3±5.8	52(26,26)	HDL-C, LDL-C, TG, TC, FBS
Curtis 2019	UK	R, DB	metabolic syndrome	M/F	6m	182mg anthocyanin	placebo	62.6±7.2	78(39,39)	SBP, DBP, HDL-C, LDL-C, TG, TC, FBS, Insulin, HbA1c
Curtis 2019	UK	R, DB	metabolic syndrome	M/F	6m	364mg anthocyanin	placebo	63±5.9	76(39,37)	SBP, DBP, HDL-C, LDL-C, TG, TC, FBS, Insulin, HbA1c
Davinelli 2015	Italy	R, DB	healthy	M/F	4w	Anthocyanin	placebo	45-65	42(16,26)	SBP, DBP, HDL-C, LDL-C, TG, TC

Gamel 2020	Canada	R, SB	obesity	M/F	8w	1.65mg anthocyanin	placebo	18-65	28(15,13)	SBP, DBP, HDL-C, LDL-C, TG, TC, FBS, Insulin
Guo(1) 2020	China	R, DB	healthy	M/F	2w	20mg anthocyanin	placebo	21.8±1.1	35(20,15)	HDL-C, LDL-C, TG, TC, FBS
Guo(2) 2020	China	R, DB	healthy	M/F	2w	40mg anthocyanin	placebo	21.5±1.4	35(20,15)	HDL-C, LDL-C, TG, TC, FBS
Guo(3) 2020	China	R, DB	healthy	M/F	2w	80mg anthocyanin	placebo	21.3±1	35(20,15)	HDL-C, LDL-C, TG, TC, FBS
Guo(4) 2020	China	R, DB	healthy	M/F	2w	160mg anthocyanin	placebo	21.4±1.3	35(20,15)	HDL-C, LDL-C, TG, TC, FBS
Guo(5) 2020	China	R, DB	healthy	M/F	2w	320mg anthocyanin	placebo	21.3±1.3	35(20,15)	HDL-C, LDL-C, TG, TC, FBS
Gurrola-Díaz 2010	Mexico	R	healthy	M/F	1m	100mg anthocyanin	placebo	30-71	54(27,27)	SBP, DBP, HDL-C, LDL-C, TG, TC, FBS
Hassellund 2012	Norway	R, DB, C	healthy	M	4w	320mg anthocyanin	placebo	41±3	27(13,14)	SBP, DBP
Hisa 2020	USA	R, DB	obesity	M/F	8w	6.5mg anthocyanin	placebo	47±16	35(17,18)	SBP, DBP, FBS, Insulin
Hollands 2018	UK	R, DB	healthy	M/F	4w	50mg anthocyanin	blonde orange juice without anthocyanins	52.2±13.6	38(19,19)	SBP, DBP, HDL-C, LDL-C, TG, TC
Johnson 2020	USA	R, SB	metabolic syndrome	M/F	12w	88mg anthocyanin	placebo	29.3±1.1	19(10,9)	SBP, DBP, HDL-C, LDL-C, TG, FBS, Insulin
Kent 2017	Australia	R	mild-to-moderate dementia	M/F	12w	300ml anthocyanin-rich cherry juice	placebo	79.5±5.8	42(21,21)	SBP, DBP
Khan(1) 2014	UK	R, DB	healthy	M/F	6w	40mg anthocyanin	placebo	55±10	43(21,22)	SBP, DBP, TC
Khan(2) 2014	UK	R, DB	healthy	M/F	6w	143mg anthocyanin	placebo	51±11	42(21,21)	SBP, DBP, TC

Kianbakht 2014	Iran	R, DB	hyperlipidemia	M/F	2m	350mg anthocyanin	placebo	51.3±15.27	80(40,40)	HDL-C, LDL-C, TG, TC
Krikorian 2012	USA	R, DB	mild cognitive impairment	M/F	16w	150mg anthocyanin	placebo	75±6	21(10,11)	SBP, DBP, FBS, Insulin
Kusunoki 2015	Japan	R	diabetes	M/F	2m	62mg anthocyanin	placebo	54±11	36(18,18)	HDL-C, LDL-C, TG, TC, FBS, HbA1c
Lee 2016	Korea	R, DB	obesity	M/F	8w	31.45mg anthocyanin	placebo	30.3±9.42	80(40,40)	SBP, DBP, HDL-C, LDL-C, TG, TC, FBS
Li 2015	China	R, DB	diabetes	M/F	24w	320mg anthocyanin	placebo	57.6±3.4	58(29,29)	SBP, DBP, HDL-C, LDL-C, TG, TC, FBS, Insulin, HbA1c
Loo 2016	Finland	R, SB, C	hypertension	M/F	8w	1024mg anthocyanin	placebo	18-65	74(37,37)	SBP, DBP, HDL-C, TG, TC, FBS
Mirfeizi 2016	Iran	R, DB	diabetes	M/F	90d	98mg anthocyanin	placebo	52±13	60(30,30)	HDL-C, LDL-C, TG, TC, FBS, Insulin, HbA1c
Odai(1) 2019	Japan	R, DB	hypertension	M/F	12w	200mg anthocyanin	placebo	53.5±7.6	20(10,10)	SBP, DBP, HDL-C, LDL-C, TG, TC
Odai(2) 2019	Japan	R, DB	hypertension	M/F	12w	400mg anthocyanin	placebo	53.5±7.6	20(10,10)	SBP, DBP, HDL-C, LDL-C, TG, TC
Qin 2009	China	R, DB	Dyslipidemia	M/F	12w	160mg anthocyanin	placebo	55.1±5.4	120(60,60)	SBP, DBP, HDL-C, LDL-C, TG, TC, FBS
Riso 2013	Italy	R, C	healthy	M/F	6w	375mg anthocyanin	placebo	47.8±9.7	18(9,9)	SBP, DBP, HDL-C, LDL-C, TG, TC, FBS
Schell 2019	USA	R, C	diabetes	M/F	4w	225mg anthocyanin	placebo	54±4.2	22(11,11)	SBP, DBP, TG, FBS
Soltani 2014	Iran	R, DB	Hyperlipidemia	M/F	4w	45mg anthocyanin	placebo	48.08±16.39	50(25,25)	HDL-C, LDL-C, TG, TC
Stote 2020	USA	R, DB	diabetes	M	8w	261.8mg anthocyanin	placebo	51-75	52(26,26)	SBP, DBP, HDL-C, LDL-C, TG, TC, FBS, Insulin, HbA1c

Stull 2010	USA	R, DB	obesity	M/F	6w	668mg anthocyanin	placebo	54±3	32(15,17)	SBP, DBP, HDL-C, LDL-C, TG, TC, FBS, Insulin
Stull 2015	USA	R, DB	metabolic syndrome	M/F	6w	580.6mg anthocyanin	placebo	55±2	54(23,21)	SBP, DBP, HDL-C, LDL-C, TG, TC, FBS, Insulin
Yang 2017	China	R, DB	diabetes	M/F	12w	320mg anthocyanin	placebo	60.8±7.9	160(80,80)	HDL-C, LDL-C, TG, TC, FBS, Insulin, HbA1c
Zhu 2011	China	R, C	Hypercholesterolemia	M/F	12w	320mg anthoycanin	placebo	40-65	146(73,73)	SBP, DBP, HDL-C, LDL-C, TG, TC, FBS, Insulin
Zhu 2013	China	R, DB	Hypercholesterolemia	M/F	24w	320mg anthoycanin	placebo	40-65	146(73,73)	HDL-C, LDL-C, TG, TC

Data are presented as mean ± SD or as a range.

R, randomized; SB, single-blinded; DB, double-blinded; C, crossover design; F, female; M, male; Ctrl, control group; Inter, Intervention group; W, week; M, month; D, day; SBP, systolic blood pressure; DBP, diastolic blood pressure; TG, triglyceride; TC, total cholesterol; HDL-C, high-density lipoprotein cholesterol; LDL-C, low-density lipoprotein-cholesterol; FBG, fasting blood glucose.

Table S1-2. Characteristics of the publications included in the meta-analysis (Catechin).

Article(first author, year)	Location	Study design	Health status	Gender	Intervention duration	Intervention substance	Comparison	Age, year	n (Ctrl, Inter)	Extracted outcome(s)
Basu 2011	USA	R, SB	metabolic syndrome	M/F	8w	928mg catechin	placbeo	42.5±1.7	22(10,12)	SBP, DBP, HDL-C, LDL-C, TG, TC, FBS, HbA1c
Bogdanski 2012	Poland	R, DB	PSA	M/F	3m	208mg EGCG	placbeo	30-60	56(28,28)	SBP, DBP, HDL-C, LDL-C, TG, FBS, Insulin

Brown 2009	UK	R	obesity	M	8w	400mg EGCG	placebo	51.55±6.45	88(42,46)	SBP, DBP, HDL-C, LDL-C, TG, TC, FBS, Insulin, HbA1c
Brown 2011	UK	R, DB, C	obesity	M	6w	800mg catechin	placbeo	40-69	135(69,66)	SBP, DBP, HDL-C, LDL-C, TG, TC, FBS, Insulin
Chan 2006	China	R	obesity	F	3m	661.3mg catechin	placbeo	25-40	34(18,16)	HDL-C, LDL-C, TG, TC, FBS, Insulin
Chen 2016	China	R, DB	obesity	F	12w	1344mg catechin	placbeo	44.5±11.4	77(38.39)	SBP, DBP, HDL-C, LDL-C, TG, TC, FBS, Insulin, HbA1c
Diepvens 2006	Netherlands	R, DB	obesity	F	12w	1125mg catechin	placbeo	19-57	46(23,23)	SBP, DBP, HDL-C, LDL-C, TC, FBS
Dostal 2016	USA	R, DB	Obese Postmenopausal Women	F	12m	1315mg catechin	placbeo	60.7±5	237(117,120)	FBS, Insulin
Dower 2015	Netherlands	R, DB, C	healthy	M/F	4w	100mg (-)-epicatechin	placebo	66.4±7.9	70(35,35)	SBP, DBP
Dower 2016	UK	R, C	healthy	M/F	4w	150mg (-)-epicatechin	placebo	61.8±9.3	40(20,20)	SBP, DBP, HDL-C, LDL-C, TG, TC
Frank 2009	UK	R, DB	healthy	M	3w	672mg catechin	placbeo	18-55	33(17,16)	SBP, DBP, HDL-C, TG, TC, FBS
Fukino 2005	Japan	R	diabetes	M/F	2m	456mg catechin	placbeo	53.5±8	66(33,33)	SBP, DBP, FBS, Insulin, HbA1c
Gutiérrez-Salmeán 2016	Mexico	R, DB	hypertriglyceridemia	M/F	4w	100mg (-)-epicatechin	placebo	18-55	22(8,14)	SBP, DBP, HDL-C, LDL-C, TG, TC
Hill 2007	Australia	R, DB, C	obese postmenopausal women	F	12w	300mg EGCG	placbeo	45-70	42(21,21)	SBP, DBP, FBS, Insulin
Hsu 2008	China	R, DB	obesity	F	3m	613.5mg catechin	placbeo	16-60	78(37,41)	SBP, DBP, HDL-C, LDL-C, TG, TC, FBS, Insulin

Hursel(1) 2009	Netherlands	R, DB	obesity	M/F	13w	270mg catechin	placbeo	18-60	40(20,20)	TG, FBS, Insulin
Hursel(2) 2009	Netherlands	R, DB	obesity	M/F	13w	270mg catechin	placbeo	18-60	40(20,20)	TG, FBS, Insulin
Kirch 2018	Germany	R, DB, C	overweight	M/F	2w	25mg (-)-epicatechin	placebo	36±11	48(24,24)	SBP
Kovacs 2004	Netherlands	R, DB	obesity	M/F	13w	573mg catechin	placbeo	18-60	104(51,53)	FBS, Insulin
Lane(1) 2018	UK	R	PSA	M	6m	600mg EGCG	placebo	63.7±4.8	133(43,45)	SBP
Lane(2) 2018	UK	R	PSA	M	6m	600mg EGCG	placebo	63.7±4.8	133(43,45)	SBP, DBP, HDL-C, LDL-C, TG, TC
Liu 2014	China	R, DB	diabetes	M/F	16w	1344mg catechin	placbeo	54.3±6.8	77(38.39)	SBP, DBP, HDL-C, LDL-C, TG, TC, FBS, Insulin, HbA1c
Lu 2016	China	R, DB	women with post-adolescent acne	F	4w	1344mg catechin	placbeo	29.1±8.9	64(33,31)	SBP, DBP, HDL-C, LDL-C, TG, TC, FBS
Matsuyama 2008	Japan	R, DB	obesity	M/F	24w	75mg catechin	placebo	11.1±0.5	42(19,23)	SBP, DBP, HDL-C, LDL-C, TG, TC
Mielgo-Ayuso 2014	Spain	R, DB	obesity	F	12w	300mg EGCG	placbeo	18-49	83(40,43)	HDL-C, LDL-C, TG, TC, FBS, Insulin
Miyazaki 2013	Japan	R, DB	healthy	M/F	14w	630.9mg catechin	placbeo	68.7±6.3	50(25,25)	SBP, DBP, HDL-C, LDL-C, TG, TC, FBS, HbA1c
Nagao 2007	Japan	R, DB	healthy	M/F	12w	583mg catechin	placebo	41.7±9.9	240(117,123)	SBP, DBP, TG, TC, FBS
Nagao 2009	Japan	R, DB	diabetes	M/F	12w	582.8mg catechin	placebo	64.9±1.8	43(20,23)	SBP, DBP, HDL-C, LDL-C, TG, TC, FBS, Insulin, HbA1c
Nagao 2005	Japan	R, DB	healthy	M	12w	690mg catechin	placbeo	24-46	35(18,17)	HDL-C, LDL-C, TG, TC, FBS, Insulin

Perkin 2023	UK	R, DB, C	healthy	M/F	3w	63mg catechin	placebo	26±7	50(25,25)	HDL-C, LDL-C, TG, FBS, Insulin
Rodriguez-Mateos(1) 2018	Germany	R	healthy	M	1m	130mg (-)-epicatechin	placebo	24±2	45(15,15/15)	SBP, DBP, HDL-C, LDL-C, TG, TC
Rodriguez-Mateos(2) 2018	Germany	R	healthy	M	1m	20mg (-)-epicatechin	placebo	24±2	45(15,15/15)	SBP, DBP, HDL-C, LDL-C, TG, TC
Saarenhovi 2017	Finland	R, DB, C	hypertension	M/F	4w	100mg (-)-epicatechin	placebo	55.3±7.3	60(30,30)	SBP, DBP, HDL-C, LDL-C, TG, TC
Sone 2011	Japan	R, DB	healthy	M/F	9w	400mg catechin	placbeo	20-70	51(25,26)	SBP, DBP, HDL-C, LDL-C, TG, TC, FBS
Suliburska 2012	Poland	R, DB	obesity	M/F	3m	208mg EGCG	placbeo	30-60	46(23,23)	SBP, DBP, HDL-C, LDL-C, FBS
Westerterp- Plantenga 2005	Netherlands	R, DB	obesity	M/F	13w	270mg catechin	placbeo	25-35	38(19,19)	FBS, Insulin
Wilasrusmee 2024	Thailand	R, DB	obesity	M/F	8w	150mg EGCG	placebo	36.3±2.4	30(15,15)	SBP, DBP
Wu(1) 2012	USA	R, DB	Healthy Postmenopausal Women	F	2m	400mg EGCG	placbeo	45-70	100(50,50)	HDL-C, LDL-C, TG, TC, FBS, Insulin, HbA1c
Wu(2) 2012	USA	R, DB	Healthy Postmenopausal Women	F	2m	800mg EGCG	placbeo	45-70	100(50,50)	HDL-C, LDL-C, TG, TC, FBS, Insulin, HbA1c
Yang 2012	China	R	obesity	M/F	8w	Catechin	placbeo	26.3±1.7	30(15,15)	SBP, DBP, HDL-C, LDL-C, TG, TC
Zeng(1) 2024	USA	R, DB	healthy	M/F	4w	890mg catechin	placebo	34±2	40(20,20)	SBP, DBP, HDL-C, TG, TC, Insulin
Zeng(2) 2024	USA	R, DB	metabolic syndrome	M/F	4w	890mg catechin	placebo	34±2	40(20,20)	SBP, DBP, HDL-C, TG, TC, Insulin

Data are presented as mean ± SD or as a range.

R, randomized; DB, double-blinded; C, crossover design; F, female; M, male; Ctrl, control group; Inter, Intervention group; W, week; M, month; PSA, prostate cancer; SBP, systolic blood pressure; DBP, diastolic blood pressure; TG, triglyceride; TC, total cholesterol; HDL-C, high density lipoprotein cholesterol; LDL-C, low density lipoprotein-cholesterol; FBG, fasting blood glucose.

Table S1-3. Characteristics of the publications included in the meta-analysis (chlorogenic acid).

Article(first author, year)	Location	Study design	Health status	Gender	Intervention duration	Intervention substance	Comparison	Age, year	n (Ctrl, Inter)	Extracted outcome(s)
Agudelo-Ochoa (1) 2016	USA	R, SB	health	M/F	8w	420mg chlorogenic acid	placebo	20-60	50(25,25)	SBP, DBP, HDL-C, LDL-C, TG, TC
Agudelo-Ochoa (2) 2016	USA	R, SB	health	M/F	8w	780mg chlorogenic acid	placebo	20-60	50(25,25)	SBP, DBP, HDL-C, LDL-C, TG, TC
Al-Dujaili 2016	Jordan	R, SB, C	health	M/F	2w	500mg chlorogenic acid	placebo	19-32	16(8,8)	SBP, DBP
Alperet 2019	Switzerland	R, DB	obesity	M/F	24w	45.4mg chlorogenic acid	placebo	36-67	126(64,62)	SBP, DBP, HDL-C, TG, FBG
Banitalebi 2019	Iran	R, SB	obesity	F	8w	250mg chlorogenic acid	placebo	30-50	30(15,15)	HDL-C, TG, FBG
Fasihi 2019	Iran	R, DB	metabolic syndrome	M/F	8w	376mg chlorogenic acid	placebo	25-50	43(22,21)	SBP, DBP, HDL-C, TG
Fukagawa 2017	Japan	R, DB	health	F	8w	270mg chlorogenic acid	placebo	25-40	49(26,23)	HDL-C, LDL-C, TG, TC, FBG, Insulin
Haidari 2017	Iran	R, DB	obesity	F	8w	180mg chlorogenic acid	placebo	20-45	64(34,30)	HDL-C, LDL-C, TG, TC, FBG, Insulin

Kim 2012	USA	R, DB	obesity	F	8w	100mg chlorogenic acid	placebo	18-70	20(10,10)	TC, FBG
Kozuma (1) 2005	Japan	R, DB	hypertension	M	4w	25mg chlorogenic acid	placebo	42.9±8.2	60(30,30)	HDL-C, LDL-C, TG, TC
Kozuma (2) 2005	Japan	R, DB	hypertension	M	4w	50mg chlorogenic acid	placebo	42.9±8.2	60(30,30)	HDL-C, LDL-C, TG, TC
Kozuma (3) 2005	Japan	R, DB	hypertension	M	4w	100mg chlorogenic acid	placebo	42.9±8.2	60(30,30)	HDL-C, LDL-C, TG, TC
Leverrier 2019	France	R, DB	obesity	M/F	12w	200mg chlorogenic acid	placebo	40.5±9.57	50(20,30)	HDL-C, LDL-C, TG, TC, FBG, HbA1c
Lopez (1) 2019	Spain	R, SB, C	health	M/F	8w	445.2mg chlorogenic acid	placebo	18-45	52(26,26)	HDL-C, LDL-C, TG, TC
Lopez (2) 2019	Spain	R, SB, C	Hypercholesterolemics	M/F	8w	445.2mg chlorogenic acid	placebo	18-45	52(26,26)	HDL-C, LDL-C, TG, TC
Ochiai 2004	Japan	R	health	M	16w	140mg chlorogenic acid	placebo	18-70	20(10,10)	HDL-C, LDL-C, TG, TC, FBG
Park 2010	USA	R, DB	obesity	F	8w	56.8mg chlorogenic acid	placebo	33.1±9.20	43(20,23)	HDL-C, LDL-C, TG, TC, FBG, Insulin
Revuelta-Iniesta 2014	UK	R, C	health	M/F	2w	chlorogenic acid	placebo	18-45	18(9,9)	SBP, DBP
Roshan 2018	Iran	R, DB	metabolic syndrome	M/F	8w	368mg chlorogenic acid	placebo	52.76±9.83	43(22,21)	HDL-C, LDL-C, TG, TC, FBG, Insulin
Sarria (1) 2018	Spain	R, SB, C	health	M/F	8w	445.2mg chlorogenic acid	placebo	18-45	52(26,26)	SBP, DBP, TG, FBG, Insulin
Sarria (2) 2018	Spain	R, SB, C	Hypercholesterolemics	M/F	8w	445.2mg chlorogenic acid	placebo	18-45	52(26,26)	SBP, DBP, TG, FBG, Insulin

Shahmohammadi 2017	USA	R, DB	obesity	M/F	8w	500mg chlorogenic acid	placebo	41.36±7.69	44(22,22)	HDL-C, LDL-C, TG, TC, FBG, Insulin
Suzuki 2019	Japan	R, DB	health	M	2w	300mg chlorogenic acid	placebo	44.6±6.2	16(8,8)	HDL-C, LDL-C, TG, TC, FBG
Terzo 2023	Italy	R, DB	obesity	M/F	6m	15mg chlorogenic acid	placebo	18-70	50(22,28)	HDL-C, LDL-C, TG, TC, HbA1c
Vinson 2012	India	R, DB, C	obesity	M/F	22w	500mg chlorogenic acid	placebo	22-46	16(8,8)	SBP, DBP
Watanabe 2006	Japan	R, DB	hypertension	M/F	12w	140mg chlorogenic acid	placebo	52±11	28(14,14)	SBP, DBP, HDL-C, LDL-C, TG, TC, FBG
Zuniga 2018	Mexico	R, DB	diabetes	M/F	12w	1200mg chlorogenic acid	placebo	30-60	26(14,12)	HDL-C, LDL-C, TG, TC, FBG

Data are presented as mean ± SD or as a range.

R, randomized; SB, single-blinded; DB, double-blinded; C, crossover design; F, female; M, male; Ctrl, control group; Inter, Intervention group; W, week; SBP, systolic blood pressure; DBP, diastolic blood pressure; TG, triglyceride; TC, total cholesterol; HDL-C, high density lipoprotein cholesterol; LDL-C, low density lipoprotein-cholesterol; FBG, fasting blood glucose.

Table S1-4. Characteristics of the publications included in the meta-analysis (Curcumin).

Article(first author, year)	Location	Study design	Health status	Gender	Intervention duration	Intervention substance	Comparison	Age, year	n (Ctrl, Inter)	Extracted outcome(s)
Alidadi 2021	Iran	R, DB	metabolic syndrome	M/F	12w	500mg curcumin	placebo	30–60	66(33,33)	SBP, DBP, HDL-C, LDL-C, TG, TC

Amin 2015	Pakistan	R, DB	metabolic syndrome	M	8w	2400mg Theracurumin	placebo	42.4±13.7	126(63,63)	SBP, DBP, HDL-C, LDL-C, TG, TC, FBS
Asadi 2019	Iran	R, DB	diabetes	M/F	8w	80mg nano-curumin	placebo	53.3±6.5	80(40,40)	FBS, HbA1c
Asghari 2024	Iran	R, DB	diabetes	M/F	12w	80mg curcumin	placebo	54.56±8.3	50(25,25)	HDL-C, LDL-C, TG, TC, FBS, Insulin, HbA1c
Bateni 2021	Iran	R, DB	metabolic syndrome	M/F	12w	80mg nano-curumin	placebo	18-70	50(25,25)	SBP, DBP, HDL-C, FBS, Insulin, HbA1c
Campbell 2019	USA	R, DB	obesity	M	12w	500mg curcumin	placebo	18-35	22(11,11)	SBP, DBP, HDL-C, LDL-C, TG, TC
Cicero 2020	Italy	R, DB	overweight	M/F	8w	800mg curcumin	placebo	18-70	80(40,40)	SBP, DBP, HDL-C, LDL-C, TG
Funamoto 2016	Japan	R, DB	chronic obstructive pulmonary diseases	M/F	24w	180mg Theracurumin	placebo	69.6±6.6	39(22,17)	SBP, DBP, HDL-C, LDL-C, TG, TC
Heshmati 2020	Iran	R, DB	PCOS	F	12w	500mg curcumin	placebo	31±5.2	67(34,33)	FBS, Insulin
Jamilian 2020	Iran	R, DB	PCOS	F	12w	500mg curcumin	placebo	28.6±4.7	50(24,26)	HDL-C, LDL-C, TG, TC, FBS, Insulin
Jazayeri- Tehrani 2019	Iran	R, DB	overweight	M/F	3m	80mg nano-curumin	placebo	41.8±5.6	84(42,42)	SBP, DBP, TG, FBS, Insulin, HbA1c
Kuszewski.H 2020	Australia	R, DB	overweight	M/F	16w	160mg curcumin	placebo	65.2±1.2	126(64,62)	SBP, DBP
Na 2012	China	R, DB	diabetes	M/F	12w	300mg curcuminoid	placebo	55.42±6.4	100(50,50)	HDL-C, LDL-C, TG, TC, FBS, HbA1c
Osali 2020	Iran	R, DB	metabolic syndrome	F	6w	80mg curcumin	placebo	62.3±1.23	17(11,6)	SBP, HDL-C, TG, FBS
Panahi 2015	Iran	R, DB	metabolic syndrome	M/F	8w	1000mg curcuminoid	placebo	44.13±9.2	117(58,59)	SBP, DBP, HDL-C, LDL-C, TG, TC, FBS, HbA1c

Rahimi 2016	Iran	R, DB	diabetes	M/F	12w	80mg curcumin	placebo	56.34±11.17	70(35,35)	HDL-C, LDL-C, TG, TC, FBS, HbA1c
Santos-Parker 2017	USA	R	healthy	M/F	12w	2000mg curcumin	placebo	62±2	44(21,23)	SBP, DBP, HDL-C, LDL-C, TG, TC
Saraf-Bank 2019	Iran	R	obesity	F	10w	500mg curcumin	placebo	13-18	60(30,30)	SBP, DBP, HDL-C, LDL-C
Sohaei 2019	Iran	R, DB	obese women with PCOS	F	6w	500mg curcumin	placebo	29.4±5.3	51(27,24)	HDL-C, LDL-C, TG, TC, FBS, Insulin
Sugawara 2012	japan	R, DB	Postmenopausal Women	F	8w	150mg curcumin	placebo	60±2	22(11,11)	SBP, DBP
Thota 2019	Australia	R, DB	diabetes	M/F	12w	1000mg curcumin	placebo	55±2.8	31(15,16)	HDL-C, LDL-C, TC, FBS, HbA1c
Yaikwawong 2024	Thailand	R, DB	diabetes	M/F	12m	1500mg curcumin	placebo	20-70	230(115,115)	FBS, Insulin, HbA1c
Yaikwawong 2024	Thailand	R, DB	diabetes	M/F	12m	250mg curcumin	placebo	62.26±0.81	227(114,113)	LDL-C, FBS, HbA1c
Zare'i 2024	Iran	R, DB	chronic obstructive pulmonary diseases	M/F	3m	80mg curcumin	placebo	59.97±4.63	60(30,30)	SBP, DBP

Data are presented as mean ± SD or as a range.

R, randomized; DB, double-blinded; C, crossover design; F, female; M, male; Ctrl, control group; Inter, Intervention group; W, week; M, month; PCOS, polycystic ovary syndrome; SBP, systolic blood pressure; DBP, diastolic blood pressure; TG, triglyceride; TC, total cholesterol; HDL-C, high density lipoprotein cholesterol; LDL-C, low density lipoprotein-cholesterol.

Table S1-5. Characteristics of the publications included in the meta-analysis (Flavanol).

Article(first author, year)	Location	Study design	Health status	Gender	Intervention duration	Intervention substance	Comparison	Age, year	n (Ctrl, Inter)	Extracted outcome(s)
Almoosawi 2012	UK	R, SB	healthy	F	4w	200mg cocoa flavanol	placebo	45-70	42(21,21)	SBP, DBP, FBS, Insulin
Baba 2007	Japan	R	healthy	M/F	12w	199mg cocoa flavanol	placebo	38±1	25(12,13)	HDL-C, LDL-C, TG, TC, FBS
Babar 2018	Canada	R, DB	preeclampsia	F	12w	high-flavanol chocolate	high-theobromine chocolate	28.6±3.56	131(60,71)	SBP
Balzer 2008	Germany	R	Diabetes	M/F	4w	963mg cocoa flavanol	placebo	64.7±9.9	41(20,21)	HDL-C, LDL-C, TG, TC, FBS, HbA1c
Curtis 2012	UK	R, DB	Diabetes	F	52w	850mg cocoa flavanol	placebo	62.13±0.73	109(50,59)	SBP, HDL-C, LDL-C, TG, TC, FBS, Insulin, HbA1c
D'Anna 2014	Italy	R	metabolic syndrome	M	24w	2110mg cocoa flavanol	placebo	56.3±3.8	60(30,30)	HDL-C, TG, FBS
Davison(1) 2008	Australia	R, DB	overweight	M/F	12w	902mg cocoa flavanol	placebo	45.2±3	26(13,13)	SBP, DBP, HDL-C, LDL-C, TG, TC, FBS, Insulin
Davison(2) 2008	Australia	R, DB	overweight	M/F	12w	902mg cocoa flavanol	placebo	45.2±3	23(11,12)	SBP, DBP, HDL-C, LDL-C, TG, TC, FBS, Insulin
Desideri 2012	Italy	R, DB	Cognitively impaired	M/F	8w	990mg cocoa flavanol	placebo	45-70	60(30,30)	SBP, DBP, HDL-C, LDL-C, TG, TC, FBS, Insulin
Grassi 2008	Italy	R	hypertension	M/F	15d	high-polyphenol dark chocolate	flavanol-free white chocolate	44.8±8	19(10,9)	SBP, DBP, HDL-C, LDL-C, TG, TC

Hollands(1) 2018	Ireland	R, DB	healthy	M/F	4w	70mg monomeric flavanols	placbeo	63±7	42(21,21)	SBP, DBP, HDL-C, LDL-C, TG, TC
Hollands(2) 2018	Ireland	R, DB	healthy	M/F	4w	140mg monomeric flavanols	placbeo	63±7	42(21,21)	SBP, DBP, HDL-C, LDL-C, TG, TC
Hollands(3) 2018	Ireland	R, DB	healthy	M/F	4w	6.5mg monomeric flavanols	placbeo	63±7	42(21,21)	SBP, DBP, LDL-C, TC
Ibero-Baraibar 2014	Spain	R, DB	healthy	M/F	4w	645.3mg flavanol	placbeo	57.26±5.24	47(24,23)	SBP, DBP
Mellor 2010	UK	R, DB	Diabetes	M/F	8w	166mg cocoa flavanol	placebo	68±1.5	24(12,12)	SBP, DBP, HDL-C, LDL-C, TG, TC, FBS, Insulin, HbA1c
Mogollon 2013	Canada	R, DB	pregnant	F	12w	flavanol-rich chocolate	low-flavanol chocolate	29.4±3.43	44(21,23)	SBP, DBP
Muniyappa 2008	USA	R, DB	hypertension	M/F	2w	902mg cocoa flavanol	placebo	51±1.5	40(20,20)	SBP, DBP, HDL-C, LDL-C, TG, TC, FBS, Insulin
Njike 2011	USA	R, DB	overweight	M/F	6w	805mg cocoa flavanol	placebo	52.5±10.4	77(38,39)	SBP, DBP, HDL-C, LDL-C, TG, TC, FBS
Ottaviani 2015	USA	R	healthy	M/F	6w	cocoa flavanol	placebo	40±8	74(28,46)	SBP, DBP
Pereira 2019	Portugal	R, DB	healthy	M/F	30d	20g cocoa flavanol	chocolate with lower cocoa content	19.9±1.7	30(15,15)	SBP, DBP
Ried 2009	Australia	R	prehypertension	M/F	12w	Flavanol-rich chocolate	placebo	53.8±12.7	21(10,11)	SBP, DBP
Sarria(1) 2014	Spain	R	healthy	M/F	2w	400mg cocoa flavanol	placebo	28.1±7.9	48(24,24)	HDL-C, LDL-C, TG, TC, FBS

Sarria(2) 2014	Spain	R	hypercholesrerolaemia	M/F	2w	400mg cocoa flavanol	placebo	28.1±7.9	40(20,20)	HDL-C, LDL-C, TG, TC, FBS
Sudarma 2011	Indonesia	R	prehypertension	M/F	15d	Flavanol-rich chocolate	placebo	34.5±6.54	32(16,16)	HDL-C, LDL-C, TC
Suominen 2020	Finland	R, DB	healthy	M/F	8w	Flavanol-rich chocolate	placebo	65-74	100(50,50)	SBP, DBP
Vauzour 2023	Australia	R, DB	older adults with memory complaints	M/F	12m	500mg flavanol	placebo	65.5±6.5	246(121,125)	HDL-C, TG, TC, FBS
West 2014	USA	R, DB	healthy	M	4w	814mg cocoa flavanol	placebo	51.7±1.2	60(30,30)	HDL-C, LDL-C, TG, TC, FBS, Insulin

Data are presented as mean ± SD or as a range.

R, randomized; DB, double-blinded; TB, triple-blinded; C, crossover design; F, female; M, male; Ctrl, control group; Inter, Intervention group; W, week; M, month; D, day; SBP, systolic blood pressure; DBP, diastolic blood pressure; TG, triglyceride; TC, total cholesterol; HDL-C, high density lipoprotein cholesterol; LDL-C, low density lipoprotein-cholesterol; FBG, fasting blood glucose.

Table S1-6. Characteristics of the publications included in the meta-analysis (Flavonoid).

Article(first author, year)	Location	Study design	Health status	Gender	Intervention duration	Intervention substance	Comparison	Age, year	n (Ctrl, Inter)	Extracted outcome(s)
Bazyar 2023	Iran	R, DB	diabetes	M/F	3m	500mg curcumin	placebo	52.4±5.62	50(25,25)	HDL-C, LDL-C, TG, TC
Bondonno 2012	Australia	R, C	healthy	M/F	10m	Flavonoid-rich apples	nitrate-rich spinach	47.3±13.6	30(15,15)	SBP, DBP, HDL-C, LDL-C, TG, TC

Curtis 2012	UK	R	Postmenopausal Women	F	12m	27g flavonoid-enriched chocolate	placebo	62.57±0.78	93(46,47)	SBP, DBP
Curtis 2013	UK	R, DB	Postmenopausal Women	M/F	1y	27g flavonoid-enriched chocolate	placebo	62.7±0.7	93(46,47)	SBP, DBP, HDL-C, TG, TC
de Jesús Romero-Prado 2015	Mexico	R	hypertension	M/F	6m	Flavonoid	placebo	42.2±7.5	79(39,40)	SBP, DBP, HDL-C, LDL-C, TG, TC
Engler 2004	USA	R, DB	healthy	M/F	2w	Flavonoid-rich dark chocolate	placebo	32.2±3.1	21(10,11)	SBP, DBP
Grassi 2009	Netherlands	R, DB, C	healthy	M	5w	tea flavonoids	placbeo	32.9±10.2	19(10,9)	SBP, DBP
Grassi 2015	Netherlands	R, DB, C	healthy	M/F	5w	500mg flavanol	placbeo	18-70	20(10,10)	SBP, DBP
Macready(1) 2014	UK	R, SB	hypertension	M/F	18w	high-flavonoid	placebo	52±2	174(57,59)	SBP, DBP
Macready(2) 2014	UK	R, SB	hypertension	M/F	18w	low-flavonoid	placebo	52±2	174(57,58)	SBP, DBP, HDL-C, LDL-C, TG, TC
Naruszewicz 2007	Poland	R, DB	healthy	M/F	6w	Flavonoid	placebo	65.97±8.1	44(22,22)	SBP, DBP
Reshef 2005	Israel	R, DB, C	healthy	M/F	5w	high-flavonoid fruit juice	low-flavonoid fruit juice	52.1±10.1	12(6,6)	HDL-C, LDL-C, TG, TC
Woolf 2023	USA	R, DB	Postmenopausal Women	F	12w	Flavonoid	placebo	60±1	43(21,22)	HDL-C, LDL-C, TG, TC

Data are presented as mean ± SD or as a range.

R, randomized; DB, double-blinded; F, female; M, male; Ctrl, control group; Inter, Intervention group; W, week; M, month; Y, year; TG, triglyceride; TC, total cholesterol; HDL-C, high density lipoprotein cholesterol; LDL-C, low density lipoprotein-cholesterol.

Table S1-7. Characteristics of the publications included in the meta-analysis (Gallic acid).

Article(first author, year)	Location	Study design	Health status	Gender	Intervention duration	Intervention substance	Comparison	Age, year	n (Ctrl, Inter)	Extracted outcome(s)
Bahorun(1) 2012	Republic of Mauritius	R	health	M	12w	50mg gallic acid	placebo	25-60	80(40,40)	HDL-C, LDL-C, TG, TC, FBG
Bahorun(2) 2012	Republic of Mauritius	R	health	F	12w	50mg gallic acid	placebo	25-60	80(40,40)	HDL-C, LDL-C, TG, TC, FBG
Chiu 2022	China	R, DB, C	health	M/F	6m	3.92g gallic acid	placebo	25-60	40(20,20)	SBP, DBP, HDL-C, LDL-C, TG, TC
Fairus 2018	Malaysia	R, SB	health	M/F	60d	450mg gallic acid	placebo	29.24±4.31	35(18,17)	SBP, DBP, HDL-C, LDL-C, TG, TC, FBG
Kubota 2011	Japan	R, DB	health	M/F	12w	gallic aicd	placebo	50.7±3.3	36(18,18)	SBP, DBP, HDL-C, LDL-C, TG, TC, FBG
Pokimica (1) 2019	USA	R, DB	Individuals at Cardiovascular Risk	M/F	4w	1177.11mg gallic acid	placebo	40.5±7.1	84(42,42)	SBP, DBP, LDL-C, TG, TC
Pokimica (2) 2019	USA	R, DB	Individuals at Cardiovascular Risk	M/F	4w	294.28mg gallic acid	placebo	40.5±7.1	84(42,42)	SBP, DBP, LDL-C, TG, TC

Data are presented as mean ± SD or as a range.

R, randomized; DB, double-blinded; SB, single-blinded; C, crossover; F, female; M, male; Ctrl, control group; Inter, Intervention group; W, week; M, month; D, day; TG, triglyceride; TC, total cholesterol; HDL-C, high density lipoprotein cholesterol; LDL-C, low density lipoprotein-cholesterol.

Table S1-8. Characteristics of the publications included in the meta-analysis (Genistein).

Article(first author, year)	Location	Study design	Health status	Gender	Intervention duration	Intervention substance	Comparison	Age, year	n (Ctrl, Inter)	Extracted outcome(s)
Atteritano 2007	Italy	R, DB	Healthy	F	2y	54mg genistein	placebo	54.7±0.25	389(198,191)	HDL-C, LDL-C, TG, TC, FBS, Insulin
Braxas 2019	Iran	R, DB	diabetes	F	12w	54mg genistein	placebo	57.92±5.72	54(28,26)	SBP, DBP, HDL-C, LDL-C, TG, TC, FBS, Insulin, HbA1c
Crisafulli 2005	Italy	R, DB	healthy postmenopausal women	F	6m	54mg genistein	placebo	56±7	60(30,30)	FBS, Insulin
De Gregorio 2017	Italy	R, DB	metabolic syndrome	F	1y	54mg genistein	placebo	18-70	22(11,11)	SBP, DBP, HDL-C, LDL-C, TG, TC
Irace 2013	Italy	R, DB	metabolic syndrome	F	6m	54mg genistein	placebo	60.1±5.9	35(20,15)	HDL-C, LDL-C, TG, TC, FBS, Insulin
Kaygusuz 2010	Turkey	R	Healthy	F	6m	50mg genistein	placebo	18-70	38(21,17)	HDL-C, TG, FBS, Insulin
Marini 2010	Italy	R, DB	postmenopausal women	F	3y	54mg genistein	placebo	53.8±0.34	138(71,67)	SBP, DBP, HDL-C, LDL-C, TG, TC, FBS, Insulin
Squadrito 2002	Italy	R, DB	Healthy	F	6m	54mg genistein	placebo	54.97	60(30,30)	SBP, DBP, HDL-C, LDL-C, TG, TC
Squadrito 2013	Italy	R, DB	metabolic syndrome	F	1y	54mg genistein	placebo	55.6±4.6	120(60,60)	HDL-C, LDL-C, TG, TC, FBS, Insulin
Usategui-Martín 2019	Spain	R, DB	Healthy	F	12w	90mg genistein	placebo	55.42±3.81	102(51,51)	HDL-C, LDL-C, TG, TC
Zhang 2019	China	R	hyperlipidemia	F	6m	60mg genistein	placebo	57.2±5.2	298(149,149)	HDL-C, LDL-C, TG, TC

Data are presented as mean ± SD or as a range.

R, randomized; DB, double-blinded; F, female; M, male; Ctrl, control group; Inter, Intervention group; W, week; M, month; Y, year; TG, triglyceride; TC, total cholesterol; HDL-C, high density lipoprotein cholesterol; LDL-C, low density lipoprotein-cholesterol.

Table S1-9. Characteristics of the publications included in the meta-analysis (Hesperidin).

Article(first author, year)	Location	Study design	Health status	Gender	Intervention duration	Intervention substance	Comparison	Age, year	n (Ctrl, Inter)	Extracted outcome(s)
Aptekmann 2010	Brazil	R	overweight	F	13w	hesperidin	placebo	30-48	26(13,13)	HDL-C, LDL-C, TG, TC
Homayouni 2018	Iran	R, DB	diabetes	M/F	6w	500mg hesperidin	placebo	51.26±8.64	60(29,31)	FBS
Morand 2011	Iran	R, C	overweight	M	12w	292mg hesperidin	placebo	56±1	24(12,12)	SBP, DBP, HDL-C, LDL-C, TG, TC, FBS, Insulin
Rangel-Huerta 2015	Spain	R, DB	obesity	M/F	12w	500mg hesperidin	placebo	18-65	100(46,54)	SBP, DBP, HDL-C, LDL-C, TG, TC, FBS, Insulin
Rizza 2011	Italy	R, DB	metabolic syndrome	M/F	3w	500mg hesperidin	placebo	52±2	24(12,12)	SBP, LDL-C, TG, TC, FBS, Insulin
Salden 2016	Netherlands	R, DB	healthy	M/F	6w	450mg hesperidin	placebo	53±14	68(34,34)	SBP, DBP, HDL-C, LDL-C, TG, TC, FBS, Insulin
Yari 2020	Iran	R	metabolic syndrome	M/F	12w	500mg hesperidin	placebo	45.19±11.11	49(24,25)	SBP, DBP, HDL-C, LDL-C, TG, TC, FBS, Insulin

Data are presented as mean ± SD or as a range.

R, randomized; DB, double-blinded; C, crossover; F, female; M, male; Ctrl, control group; Inter, Intervention group; W, week; TG, triglyceride; TC, total cholesterol; HDL-C, high density lipoprotein cholesterol; LDL-C, low density lipoprotein-cholesterol.

Table S1-10. Characteristics of the publications included in the meta-analysis (Isoflavone).

Article(first author, year)	Location	Study design	Health status	Gender	Intervention duration	Intervention substance	Comparison	Age, year	n (Ctrl, Inter)	Extracted outcome(s)
Atkinson 2004	Australia	R, DB	healthy	F	12m	43.5mg isoflavone	placebo	49-65	177(100,77)	SBP, DBP
Aubertin-Leheudre 2008	Canada	R, DB	obese postmenopausal women	F	6m	70mg isoflavone	placebo	57.1±5.6	50(25,25)	SBP, DBP, HDL-C, LDL-C, TG, TC, FBS, Insulin
Azadbakht 2008	Iran	R	diabetes	M/F	4y	43mg isoflavone	placebo	62.1±12.1	41(20,21)	SBP, DBP, HDL-C, LDL-C, TG, TC, FBS
Bosland 2021	USA	R	healthy	M	2y	41mg isoflavone	placebo	61.5±2.3	93(43,50)	SBP, DBP, HDL-C, LDL-C, TG
Chan 2008	China	R, DB	healthy	M/F	12w	80mg isoflavone	placebo	66±10	102(52,20)	SBP, DBP, HDL-C, LDL-C, TG, TC
Charles 2009	USA	R, DB	healthy postmenopausal women	F	12w	160mg isoflavone	placebo	56.1±0.84	75(43,32)	FBS, Insulin
Chilibeck 2013	Canada	R, DB	healthy	F	2y	165mg isoflavone	placebo	56.6±6.7	149(73,76)	HDL-C, LDL-C, TG, TC
Hall 2006	UK	R, DB, C	healthy postmenopausal women	F	8w	50mg isoflavone	placebo	45-70	230(115,115)	HDL-C, LDL-C, TG, TC, FBS, Insulin
Han 2002	Brazil	R, DB	healthy	F	4m	100mg soy isoflavone	placebo	48±1.2	80(40,40)	SBP, DBP, HDL-C, LDL-C, TG, TC

Hermansen 2001	Norway	R, DB	diabetes	M/F	6w	165mg isoflavone	placebo	63.6±7.5	20(10,10)	SBP, DBP, FBS, Insulin, HbA1c
Hidalgo 2005	Ecuador	R, DB	healthy	F	90d	80mg isoflavone	placebo	51.3±3.5	53(26,27)	SBP, DBP, HDL-C, LDL-C, TG, TC
Howes 2003	Australia	R, DB, C	Postmenopausal Women	F	4w	50mg isoflavone	placebo	62±2	16(8,8)	SBP, DBP, HDL-C, LDL-C, TG, TC
Jayagopal 2002	UK	R, DB, C	Postmenopausal Women With Diabetes	F	12w	135mg isoflavone	placebo	63.5±12.1	64(32,32)	SBP, DBP, HDL-C, LDL-C, TG, TC, FBS, Insulin, HbA1c
Jenkins(1) 2002	USA	R	hyperlipidemic	M/F	3m	73mg isoflavone	placebo	62±2	41(20,21)	SBP, DBP, HDL-C, LDL-C, TG, TC
Jenkins(2) 2002	USA	R	hpostmenopausal women	M/F	3m	10mg isoflavone	placebo	62±2	41(20,21)	HDL-C, LDL-C, TG, TC
Khaodhiar (1) 2008	USA	R, DB	menopausal women	F	12w	40mg isoflavone	placebo	52.2±4.8	93(45,48)	SBP, DBP, HDL-C, LDL-C, TG, TC, FBS, Insulin
Khaodhiar (2) 2008	USA	R, DB	menopausal women	F	12w	80mg isoflavone	placebo	53.2±5.6	94(45,49)	SBP, DBP, HDL-C, LDL-C, TG, TC, FBS, Insulin
Konya(1) 2019	Qatar	R, DB	diabetes	M/F	8w	32mg isoflavone	placebo	65.1±7.3	26(13,13)	SBP, DBP, HDL-C, LDL-C, TG, TC, FBS, Insulin, HbA1c
Konya(2) 2019	Qatar	R, DB	diabetes	M/F	8w	32mg isoflavone	placebo	65.1±7.3	23(12,11)	SBP, DBP, HDL-C, LDL-C, TG, TC, FBS, Insulin, HbA1c
Lambert 2017	Denmark	R, DB	peri-menopausal	F	2w	34mg isoflavone	placebo	52.34±3.44	59(29,30)	HDL-C, LDL-C, TG, TC
Liu 2010	China	R, DB	women with prediabetes or untreated early diabetes	F	6m	25mg isoflavone	placebo	24.5±3.7	120(60,60)	FBS, Insulin
Nestel(1) 1999	Australia	R	healthy	F	10w	40mg isoflavone	placebo	55.7±1	17(8,9)	HDL-C, LDL-C, TG, TC

Nestel(2) 1999	Australia	R	healthy	F	10w	80mg isoflavone	placebo	55.7±1	17(8,9)	SBP, DBP, HDL-C, LDL-C, TG, TC
Richter(1) 2017	USA	R, C	hypertension	M/F	8w	isoflavone-containing soya protein	placebo	51.4±2.1	20(10,10)	SBP, DBP, HDL-C, LDL-C, TG, TC
Richter(2) 2017	USA	R, C	hypertension	M/F	8w	isoflavone-containing soya protein	placebo	51.4±2.1	20(10,10)	SBP, DBP, HDL-C, TC
Sagara 2004	Japan	R, DB	hypertension	M	5w	80mg isoflavone	placebo	52.2±4.1	50(25,25)	SBP, DBP, HDL-C, LDL-C, TG, TC
Sathyapalan 2017	UK	R, DB	menopausal women	F	6m	66mg isoflavone	soy protein	53±2.1	200(100,100)	SBP, DBP, HDL-C, LDL-C, TG, FBS, Insulin
Sathyapalan 2018	UK	R	menopausal women	F	6m	66mg isoflavone	placebo	52±1	200(100,100)	SBP, DBP, TC
Teede 2003	Australia	R, DB	healthy	M/F	12w	80mg isoflavone	placebo	45-75	80(40,40)	SBP, DBP, HDL-C, LDL-C, TG, TC
Teixeira 2004	Portugal	R, DB	diabetes	M	8w	25mg isoflavone	placebo	53-73	14(7,7)	HDL-C, LDL-C, TG, TC, HbA1c
Uesugi 2004	Japan	R, DB, C	climacteric	F	8w	40mg isoflavone	placebo	58±7	58(30,28)	SBP, DBP
Wong 2012	USA	R, DB	healthy	F	6w	80mg isoflavone	placebo	55.7±4.3	72(36,36)	SBP, DBP

Data are presented as mean ± SD or as a range.

R, randomized; DB, double-blinded; C, crossover design; F, female; M, male; Ctrl, control group; Inter, Intervention group; W, week; M, month; D, day; SBP, systolic blood pressure; DBP, diastolic blood pressure; TG, triglyceride; TC, total cholesterol; HDL-C, high density lipoprotein cholesterol; LDL-C, low density lipoprotein-cholesterol; FBG, fasting blood glucose.

Table S1-11. Characteristics of the publications included in the meta-analysis (Quercetin).

Article(first author, year)	Location	Study design	Health status	Gender	Intervention duration	Intervention substance	Comparison	Age, year	n (Ctrl, Inter)	Extracted outcome(s)
Bondonno(1) 2016	Australia	R, C	healthy	M/F	1w	50 mg quercetin-3-O-glucoside	placebo	60.8±9.3	15(7,8)	SBP, DBP
Bondonno(2) 2016	Australia	R, C	healthy	M/F	1w	100 mg quercetin-3-O-glucoside	placebo	60.8±9.3	15(7,8)	SBP, DBP
Bondonno(3) 2016	Australia	R, C	healthy	M/F	1w	200 mg quercetin-3-O-glucoside	placebo	60.8±9.3	15(7,8)	SBP, DBP
Bondonno(4) 2016	Australia	R, C	healthy	M/F	1w	400 mg quercetin-3-O-glucoside	placebo	60.8±9.3	15(7,8)	SBP, DBP, HDL-C, LDL-C, TG, TC
Brüll 2015	Germany	R, DB, C	overweight-to-obese subjects with pre-hypertension and stage I hypertension	M/F	6w	162mg quercetin	placebo	47.4±10.5	70(35,35)	SBP, DBP, HDL-C, LDL-C, TG, TC, FBS, Insulin, HbA1c
Brüll 2017	Germany	R, DB, C	overweight-to-obese subjects with pre-hypertension and stage I hypertension	M/F	6w	54mg quercetin	placebo	47.4±10.5	70(35,35)	SBP, DBP, HDL-C, LDL-C, TG, TC
Burak 2019	Germany	R, DB, C	healthy	M/F	8w	190mg quercetin	placebo	24.6±3.9	67(33,34)	SBP, DBP, HDL-C, LDL-C, TG, TC
Choi 2015	Korea	R, DB	healthy obese individuals	M/F	12w	100mg quercetin	placebo	42.5±8.9	62(28,34)	HDL-C, LDL-C, TG, TC, FBS

Conquer 1998	Italy	R	healthy	M/F	28d	1g quercetin	placebo	41.7±2.8	27(14,13)	SBP, DBP, HDL-C, LDL-C, TG, TC
Edwards(1) 2007	USA	R, DB, C	hypertension	M/F	28d	730mg quercetin	placebo	50±2	50(25,25)	SBP, DBP, HDL-C, LDL-C, TG, TC, FBS
Edwards(2) 2007	USA	R, DB, C	hypertension	M/F	28d	730mg quercetin	placebo	50±2	50(25,25)	SBP, DBP, HDL-C, LDL-C, TG, TC, FBS
Egert 2009	Germany	R, DB, C	overweight	M/F	6w	150mg quercetin	placebo	45.1±10.53	93(50,43)	HDL-C, LDL-C, TG, TC
Egert(1) 2010	Germany	R, DB, C	healthy	M/F	6w	150mg quercetin	placebo	45±10.5	60(26,34)	HDL-C, LDL-C, TG, TC
Egert(2) 2010	Germany	R, DB, C	healthy	M/F	6w	150mg quercetin	placebo	45±10.5	60(26,34)	SBP, DBP, HDL-C, LDL-C, TG, TC
Khorshidi 2018	Iran	R, DB	obese women with PCOS	F	12w	1000mg quercetin	placebo	20-40	78(39,39)	FBS, Insulin
Lee 2016	Korea	R, DB	obesity	M/F	12w	100mg quercetin	placebo	42.6±9.4	72(36,36)	HDL-C, LDL-C, TG, TC, FBS
Lee 2011	Korea	R, DB	healthy	M	10w	100mg quercetin	placebo	42.4±8.2	92(46,46)	SBP, DBP, HDL-C, LDL-C, TG, TC, FBS
Pfeuffer(1) 2013	Australia	R, DB, C	obesity	M	4w	150mg quercetin	placebo	38.3±3.2	24(12,12)	SBP, HDL-C, LDL-C, TG, TC, FBS
Pfeuffer(2) 2013	Australia	R, DB, C	obesity	M	4w	150mg quercetin	placebo	38.3±3.2	24(12,12)	SBP, HDL-C, LDL-C, TG, TC, FBS
Rezvan 2017	Iran	R, DB	PCOS	F	12w	1000mg quercetin	placebo	29.45±4.09	82(41,41)	FBS, Insulin

Data are presented as mean ± SD or as a range.

R, randomized; DB, double-blinded; C, crossover design; F, female; M, male; Ctrl, control group; Inter, Intervention group; W, week; D, day; SBP, systolic blood pressure; DBP, diastolic blood pressure; TG, triglyceride; TC, total cholesterol; HDL-C, high density lipoprotein cholesterol; LDL-C, low density lipoprotein-cholesterol; FBG, fasting blood glucose.

Table S1-12. Characteristics of the publications included in the meta-analysis (Resveratrol).

Article(first author, year)	Location	Study design	Health status	Gender	Intervention duration	Intervention substance	Comparison	Age, year	n (Ctrl, Inter)	Extracted outcome(s)
Abdollahi 2019	Iran	R, DB	diabetes	M/F	8w	1g resveratrol	placebo	50.14±7.38	71(35,36)	HDL-C, LDL-C, TG, TC, FBS, Insulin, HbA1c
Anton(1) 2014	USA	R, DB	overweight	M/F	12w	300mg resveratrol	placebo	73±7	32(10,12)	SBP, DBP
Anton(2) 2014	USA	R, DB	overweight	M/F	12w	1000mg resveratrol	placebo	73±7	32(10,12)	SBP, DBP, HDL-C, LDL-C, TG, TC
Arzola-Paniagua 2016	Mexico	R, DB	obesity	M/F	28w	120mg resveratrol	placebo	40.9±10	39(24,15)	TG, FBS, Insulin
Banaszewska 2016	Poland	R, DB	PCOS	F	12w	1500mg resveratrol	placebo	26.8±1.1	30(15,15)	HDL-C, LDL-C, TG, TC, FBS, Insulin
Bhatt 2013	India	R	diabetes	M/F	6m	250mg resveratrol	placebo	30-70	57(29,28)	SBP, DBP, HDL-C, TG, TC, FBS, HbA1c
Chachay 2014	Australia	R, DB	obesity	M	8w	3g resveratrol	placebo	48.8±12.2	20(10,10)	SBP, DBP, HDL-C, LDL-C, TG, TC, FBS, Insulin
Hassan 2023	Pskistan	R, DB	PCOS	F	12w	2000mg resveratrol	placebo	26.52=4.41	110(55,55)	SBP, DBP
Imamura 2017	Japan	R, DB	diabetes	M/F	12w	100mg resveratrol	placebo	57.8±10.4	50(25,25)	SBP, DBP, HDL-C, LDL-C, TG, TC, FBS, HbA1c
Kantartzis 2018	Germany	R, DB	obesity	M/F	12w	150mg resveratrol	placebo	18-70	108(54,54)	SBP, DBP, HDL-C, LDL-C, TG, TC, FBS, HbA1c
Khodabandehloo 2018	Tehran, Iran	R, DB	diabetes	M/F	8w	800mg resveratrol	placebo	58.75±6.11	45(20,25)	SBP, DBP, HDL-C, LDL-C, TG, TC, FBS, Insulin, HbA1c

Kjær(1) 2017	Aarhus, Denmark	R, DB	metabolic syndrome	M	16w	1000mg resveratrol	placebo	49.5±0.796	66(33,33)	SBP, DBP, FBS, Insulin
Kjær(2) 2017	Aarhus, Denmark	R, DB	metabolic syndrome	M	16w	150mg resveratrol	placebo	49.5±0.796	66(33,33)	SBP, DBP, FBS, Insulin
Kobe 2017	Germany	R, DB	mild cognitive imppairment	M/F	26w	200mg resveratrol	placebo	65±9	40(18,22)	FBS, Insulin, HbA1c
Marques 2018	Rio de Janeiro, Brazil	R, DB, C	hypertension	M/F	2m	300mg trans-resveratrol	placebo	54±1	24(12,12)	SBP, DBP
Movahed 2016	USA	R, DB, C	hypertension	M/F	12w	500mg resveratrol	placebo	54±1	100(50,50)	SBP, DBP, HDL-C, LDL-C, TG, TC
Movahed 2013	Iran	R, DB	diabetes	M/F	6w	1g resveratrol	placebo	51.81±6.99	66(33,33)	SBP, DBP, HDL-C, LDL-C, TG, TC, FBS, Insulin, HbA1c
Poulsen 2013	Denmark	R, DB	obesity	M	4w	500mg resveratrol	placebo	31.9±2.9	24(12,12)	HDL-C, LDL-C, TG, TC, FBS, Insulin, HbA1c
Sattarinezhad 2019	Iran	R, DB	diabetes	M/F	12w	500mg resveratrol	placebo	56.8±9.7	60(30,30)	SBP, DBP, FBS, Insulin, HbA1c
Seyyedebrahimi 2018	Tehran,Iran	R, DB	diabetes	M/F	2m	800mg resveratrol	placebo	56.84±6.16	48(25,23)	SBP, DBP, HDL-C, LDL-C, TG, TC, FBS, HbA1c
Thazhath 2016	Australia	R, DB	diabetes	M/F	5w	1000mg resveratrol	placebo	67.5±1.6	14(7,7)	FBS, HbA1c
Timmers 2011	Netherlands	R, DB, C	obesity	M	30d	150mg resveratrol	placebo	52.5±2.1	11(5,6)	SBP, DBP, HDL-C, LDL-C, TG, TC, FBS, Insulin
Timmers 2016	Netherlands	R, DB, C	diabetes	M	30d	150mg resveratrol	placebo	64（59.19-67.28）	34(17,17)	SBP, DBP
Witte 2014	Germany	R, DB	overweight	M/F	26w	200mg resveratrol	placebo	64.8±6.8	46(23,23)	TC, FBS, Insulin, HbA1c

Wong 2013	Australia	R	obesity	M/F	12w	75mg resveratrol	placebo	61±1.3	31(15,16)	SBP, DBP
Yoshino 2012	Switzerland	R, DB	overweight	F	12w	75mg resveratrol	placebo	59.8±4.3	29(14,15)	SBP, DBP, HDL-C, LDL-C, TG, TC, FBS, Insulin
Zare 2017	Iran	R, DB	diabetes	M/F	4w	480mg resveratrol	placebo	50.9±8.9	43(22,21)	TG, FBS, Insulin
Zhou(1) 2023	China	R, DB	dyslipidemia	M/F	8w	100mg resveratrol	placebo	61.3=8.96	80(40,40)	HDL-C, LDL-C, TG, TC, FBS, Insulin
Zhou(2) 2023	China	R, DB	dyslipidemia	M/F	8w	300mg resveratrol	placebo	61.3=8.96	80(40,40)	HDL-C, LDL-C, TG, TC, FBS, Insulin
Zhou(3) 2023	China	R, DB	dyslipidemia	M/F	8w	600mg resveratrol	placebo	61.3=8.96	80(40,40)	HDL-C, LDL-C, TG, TC, FBS, Insulin

Data are presented as mean ± SD or as a range.

R, randomized; DB, double-blinded; C, crossover design; F, female; M, male; Ctrl, control group; Inter, Intervention group; W, week; M, month; D, day; SBP, systolic blood pressure; DBP, diastolic blood pressure; TG, triglyceride; TC, total cholesterol; HDL-C, high density lipoprotein cholesterol; LDL-C, low density lipoprotein-cholesterol; FBG, fasting blood glucose.

Appendix 2-Forest plot

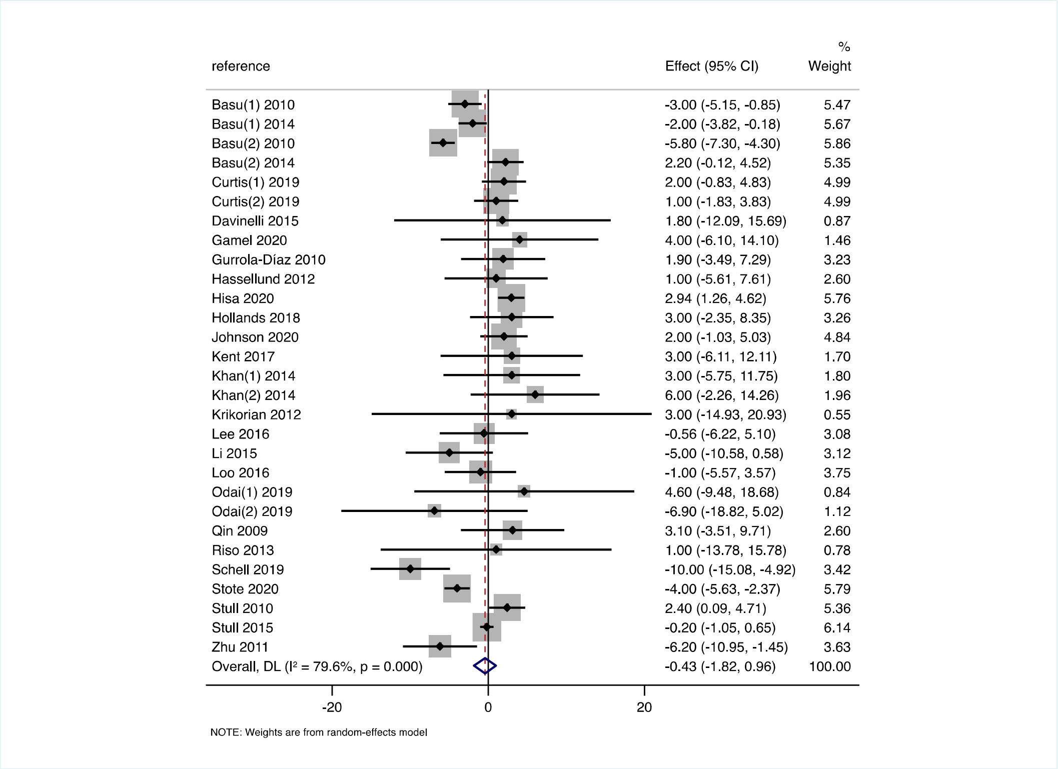

Figure S1-1-1 Forest plot of RCTs investigating the effect of anthocyanin supplementation on SBP.

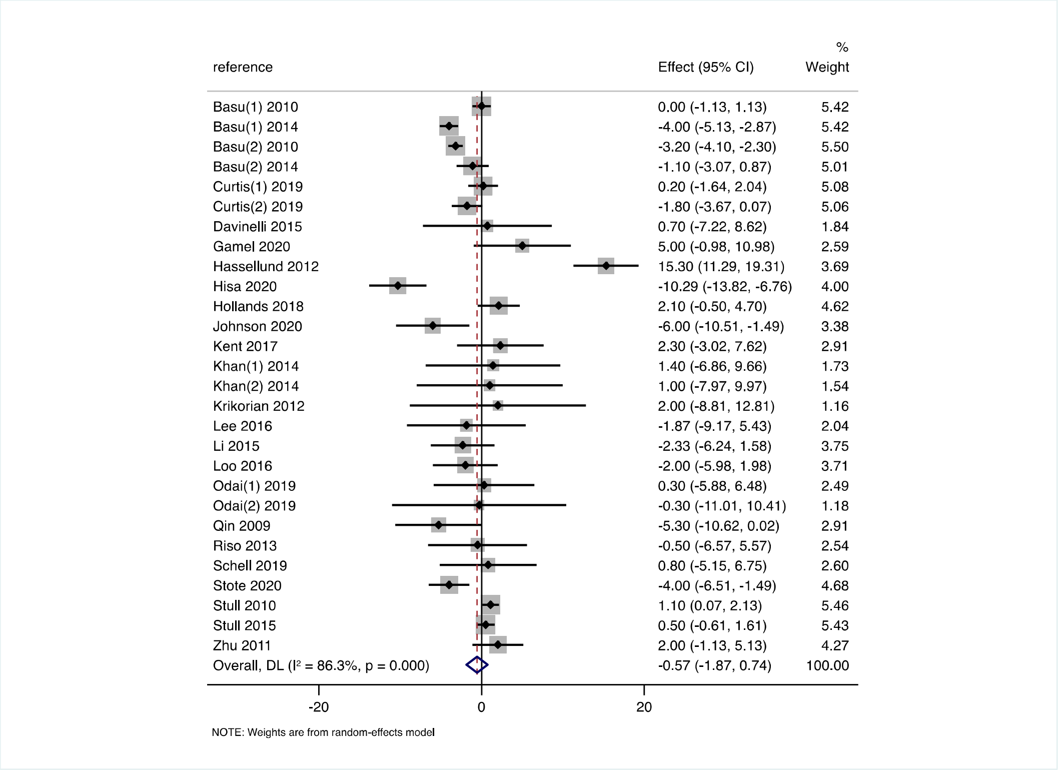

Figure S1-1-2 Forest plot of RCTs investigating the effect of anthocyanin supplementation on DBP.

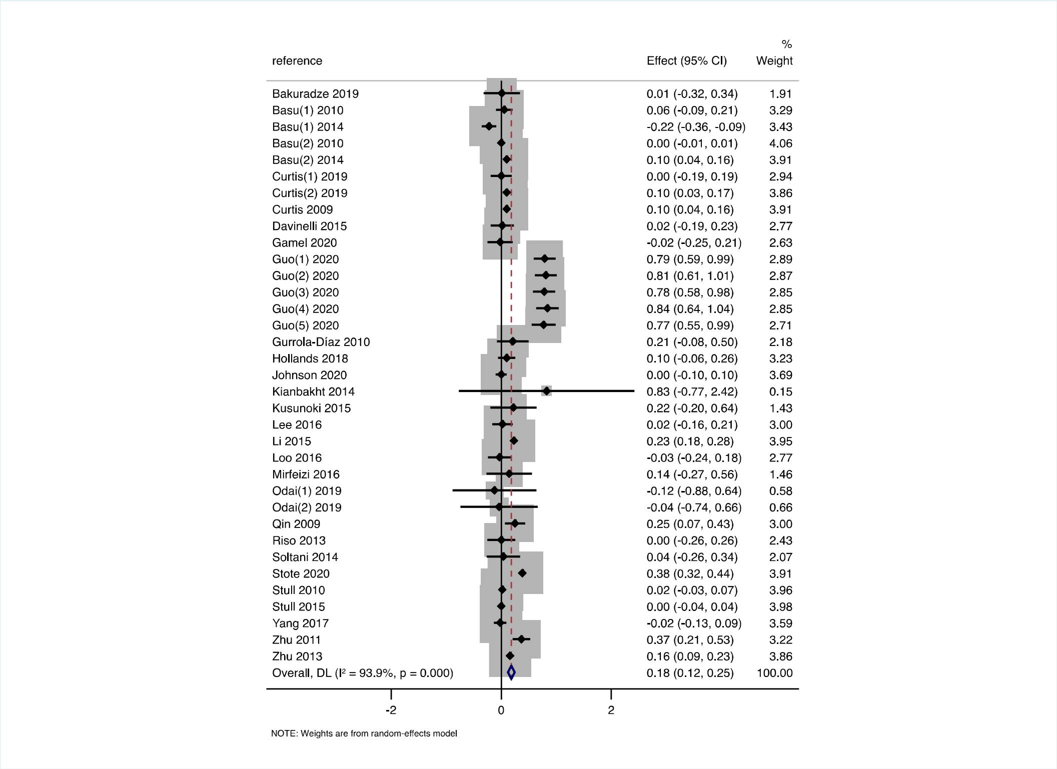

Figure S1-1-3 Forest plot of RCTs investigating the effect of anthocyanin supplementation on HDL-C.

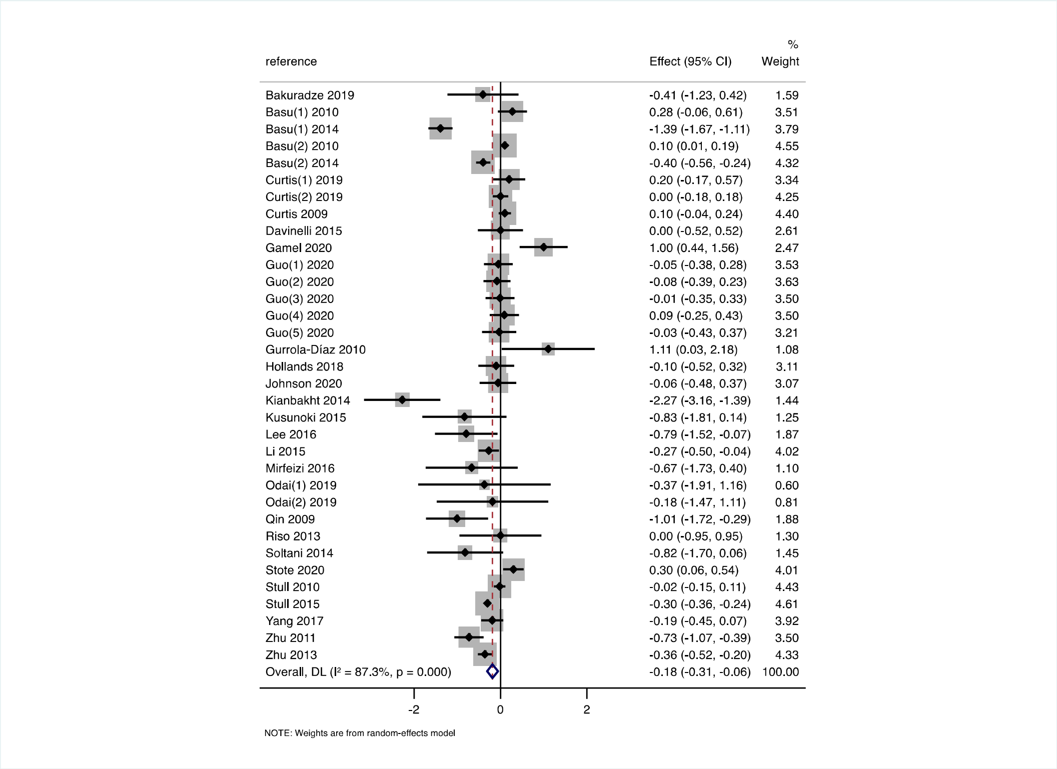

Figure S1-1-4 Forest plot of RCTs investigating the effect of anthocyanin supplementation on LDL-C.

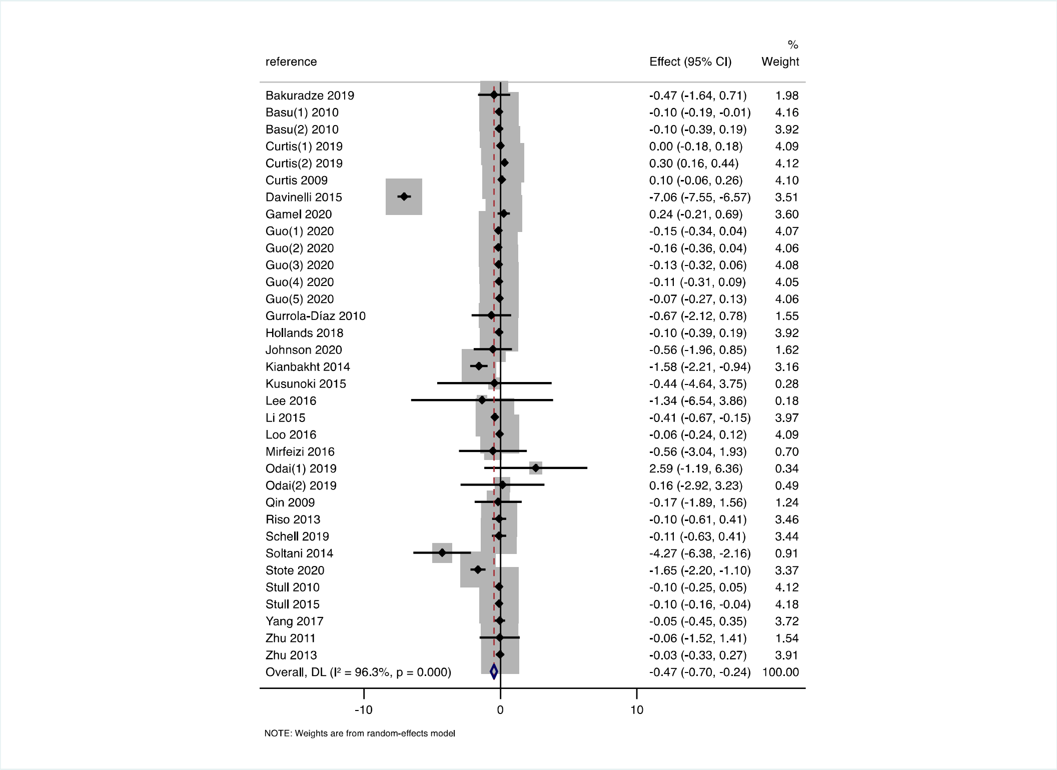

Figure S1-1-5 Forest plot of RCTs investigating the effect of anthocyanin supplementation on TG

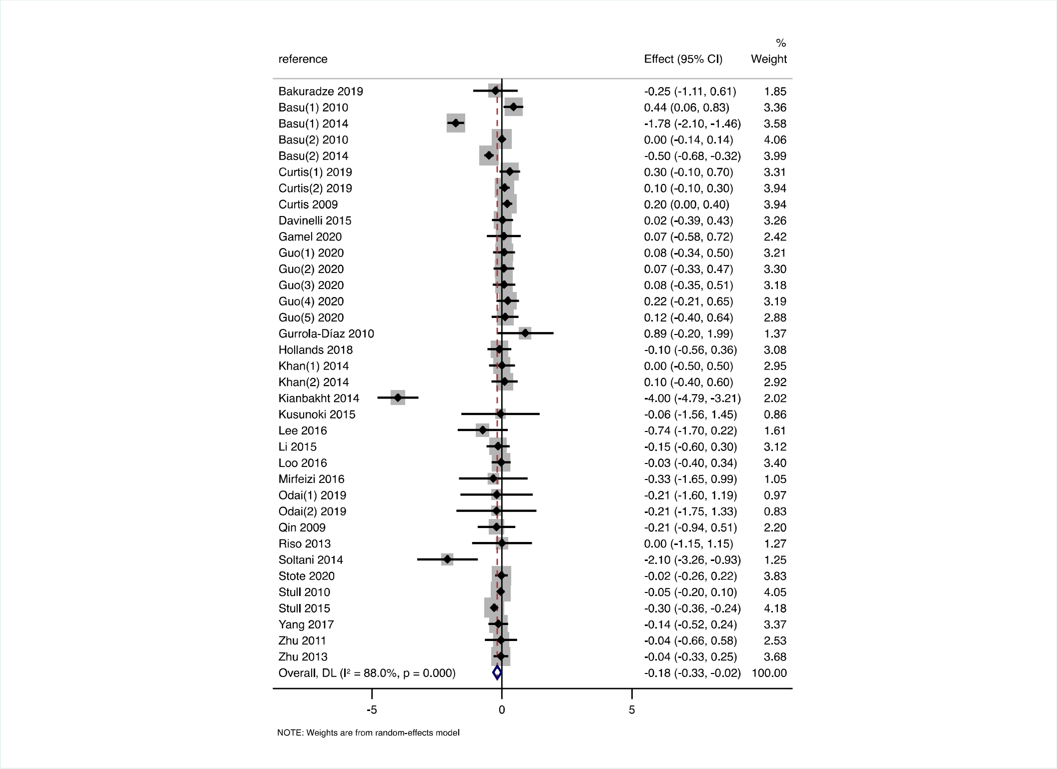

Figure S1-1-6 Forest plot of RCTs investigating the effect of anthocyanin supplementation on TC.

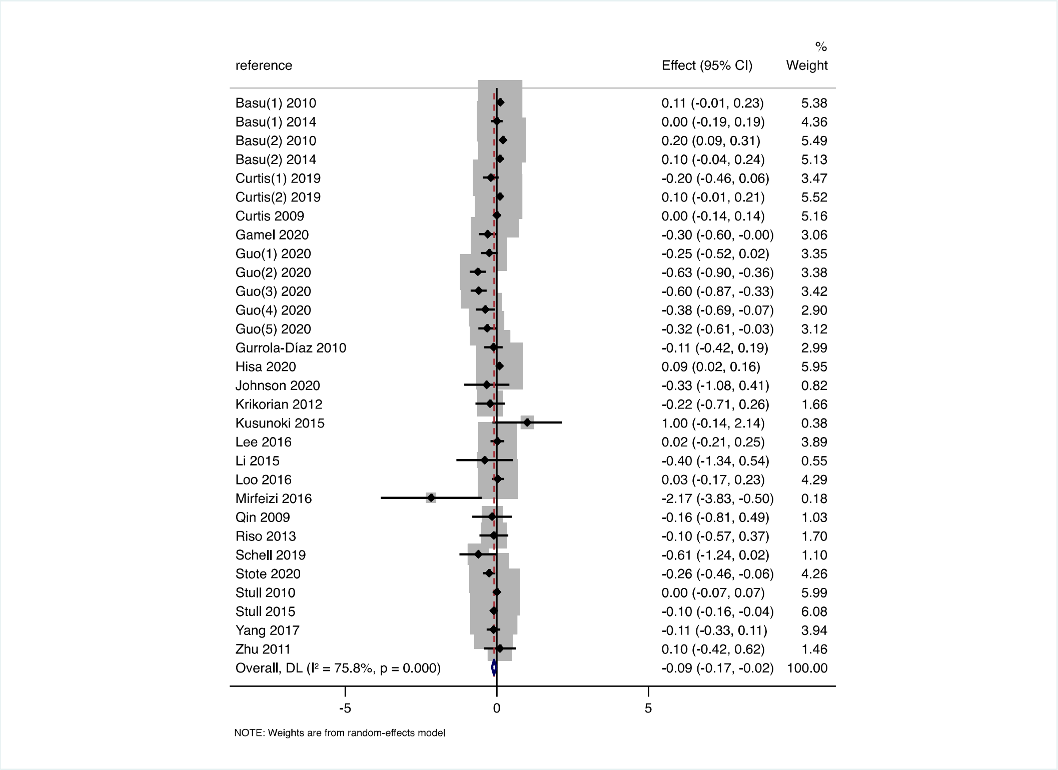

Figure S1-1-7 Forest plot of RCTs investigating the effect of anthocyanin supplementation on FBG.

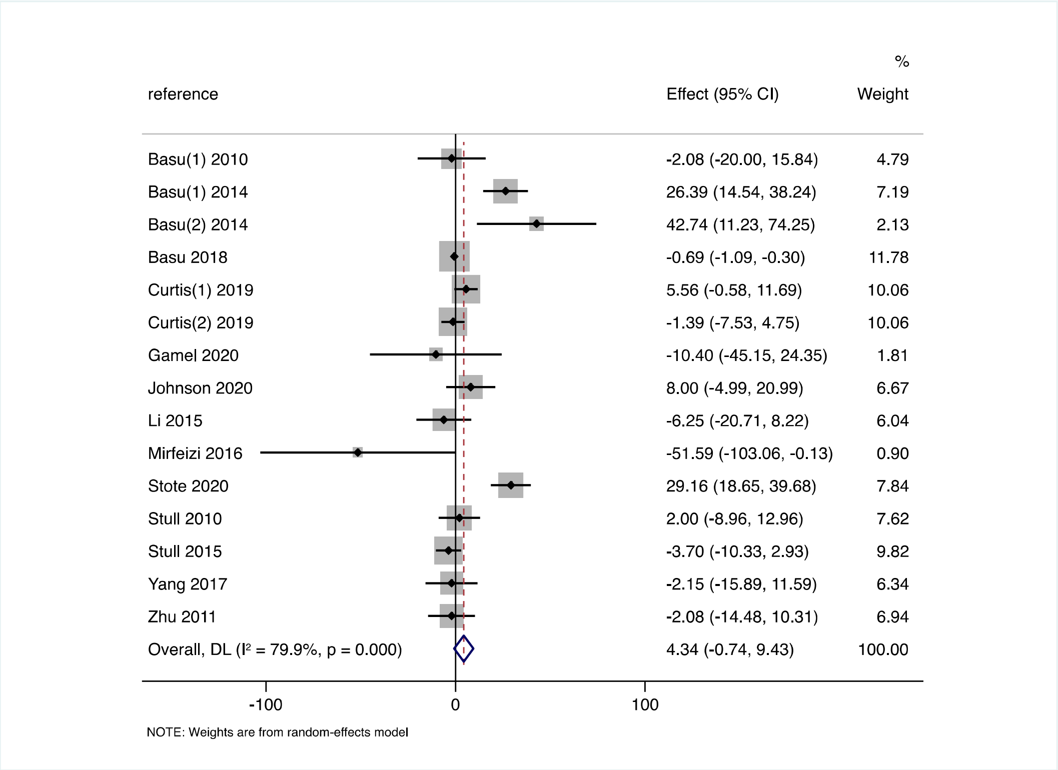

Figure S1-1-8 Forest plot of RCTs investigating the effect of anthocyanin supplementation on FBI.

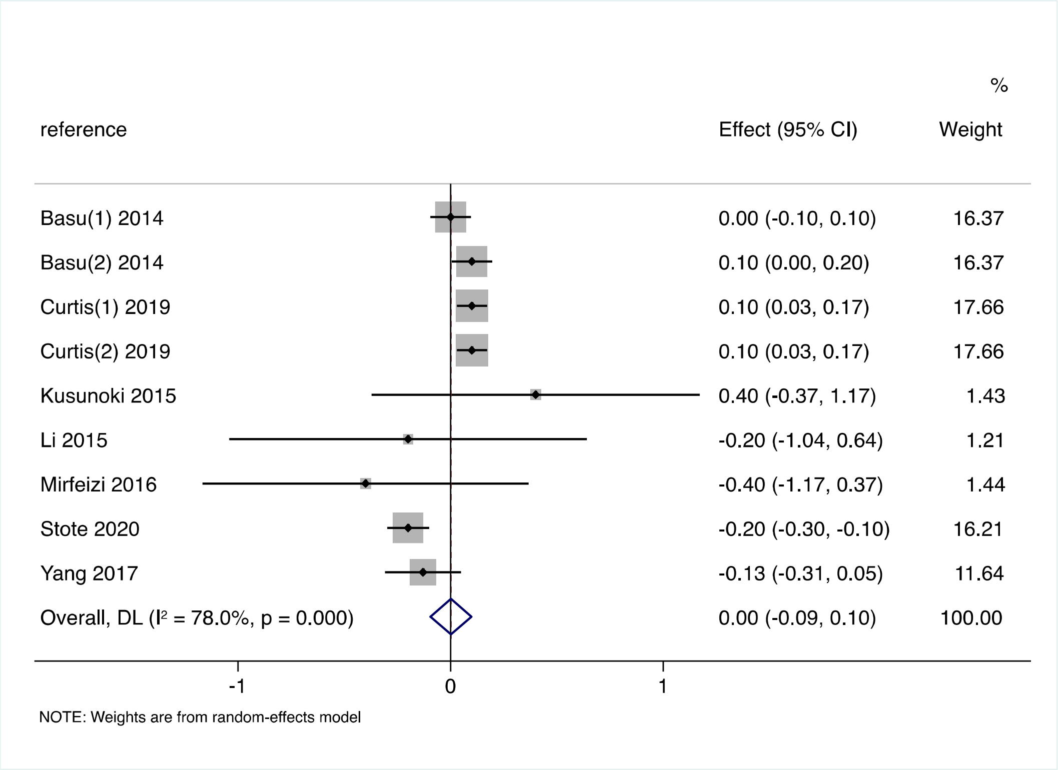

Figure S1-1-9 Forest plot of RCTs investigating the effect of anthocyanin supplementation on A1C.

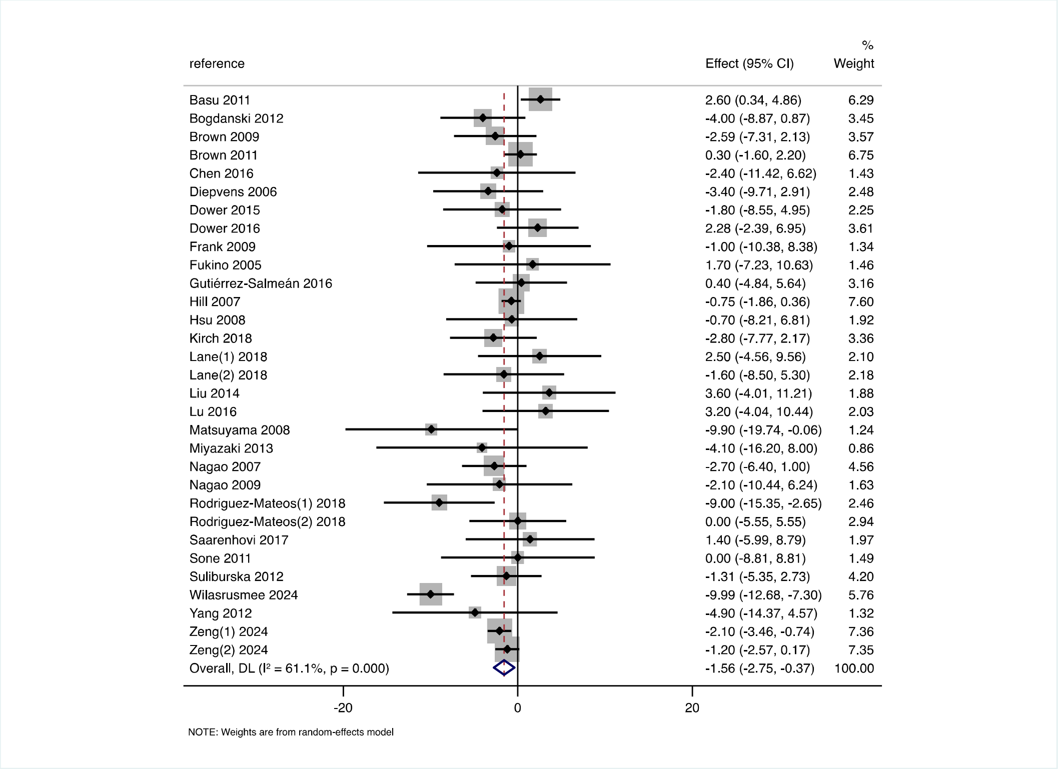

Figure S1-2-1 Forest plot of RCTs investigating the effect of catechin supplementation on SBP.

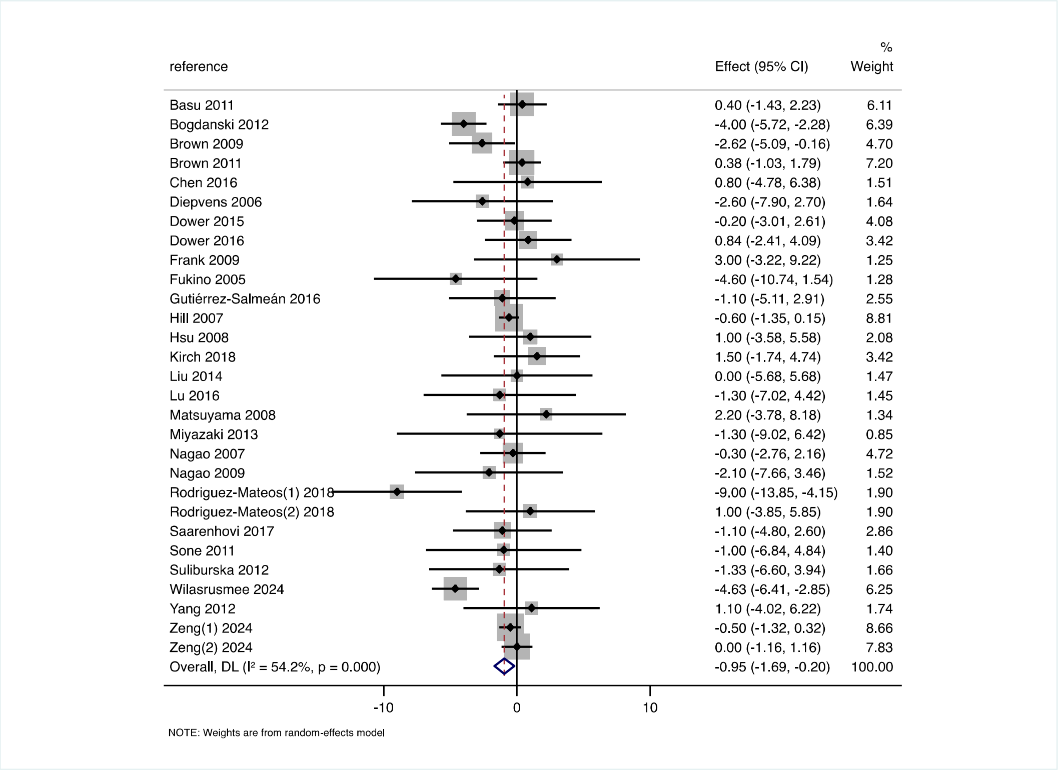

Figure S1-2-2 Forest plot of RCTs investigating the effect of catechin supplementation on DBP.

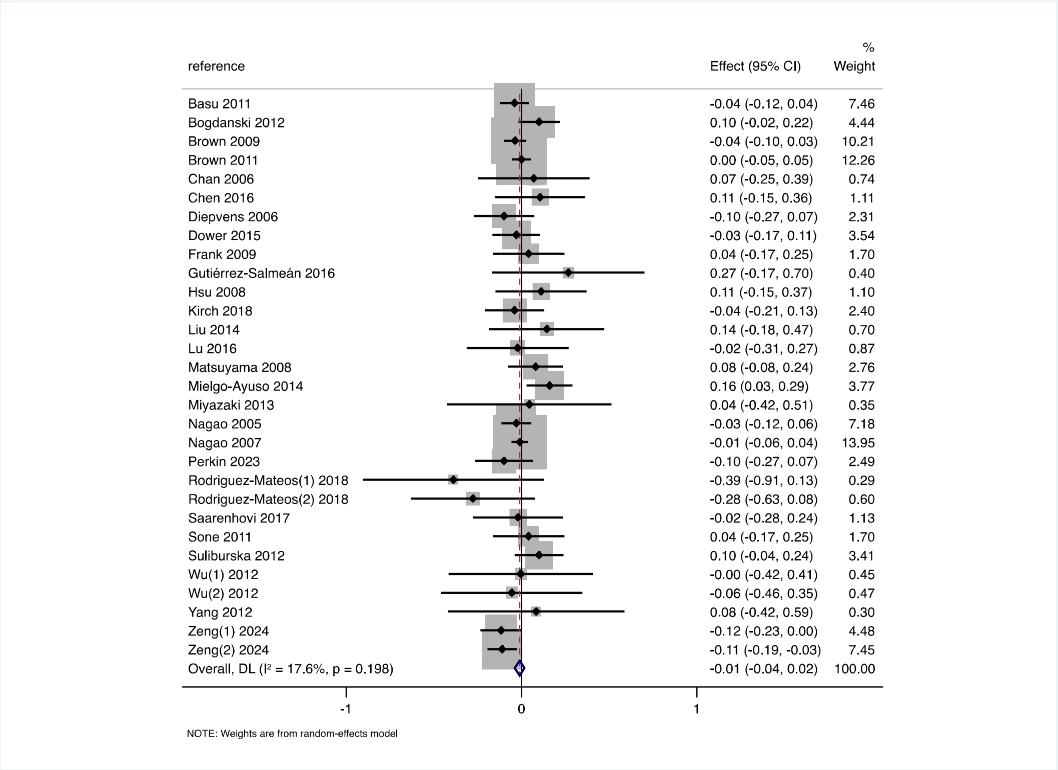

Figure S1-2-3 Forest plot of RCTs investigating the effect of catechin supplementation on HDL-C.

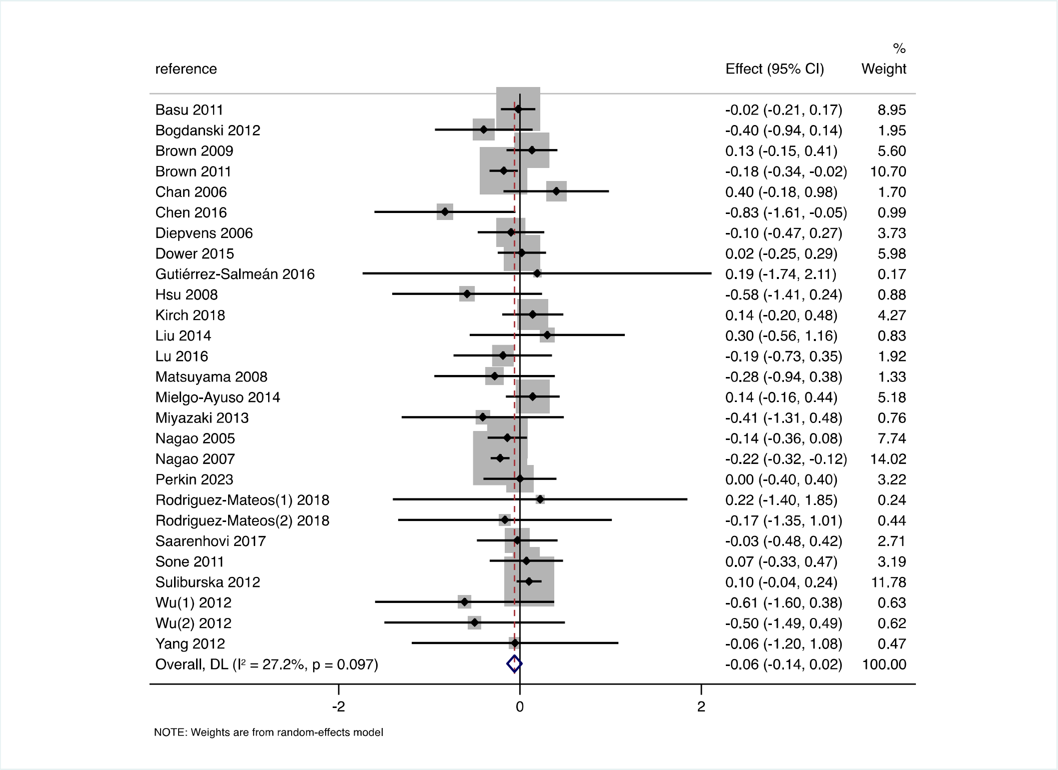

Figure S1-2-4 Forest plot of RCTs investigating the effect of catechin supplementation on LDL-C.

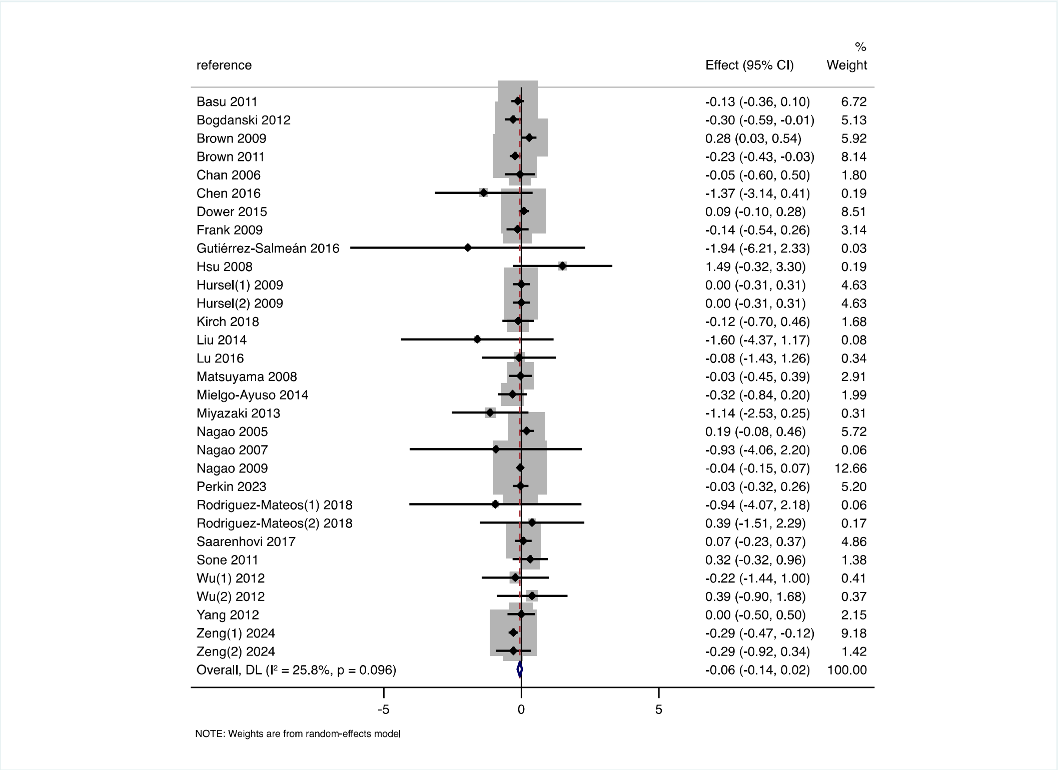

Figure S1-2-5 Forest plot of RCTs investigating the effect of catechin supplementation on TG

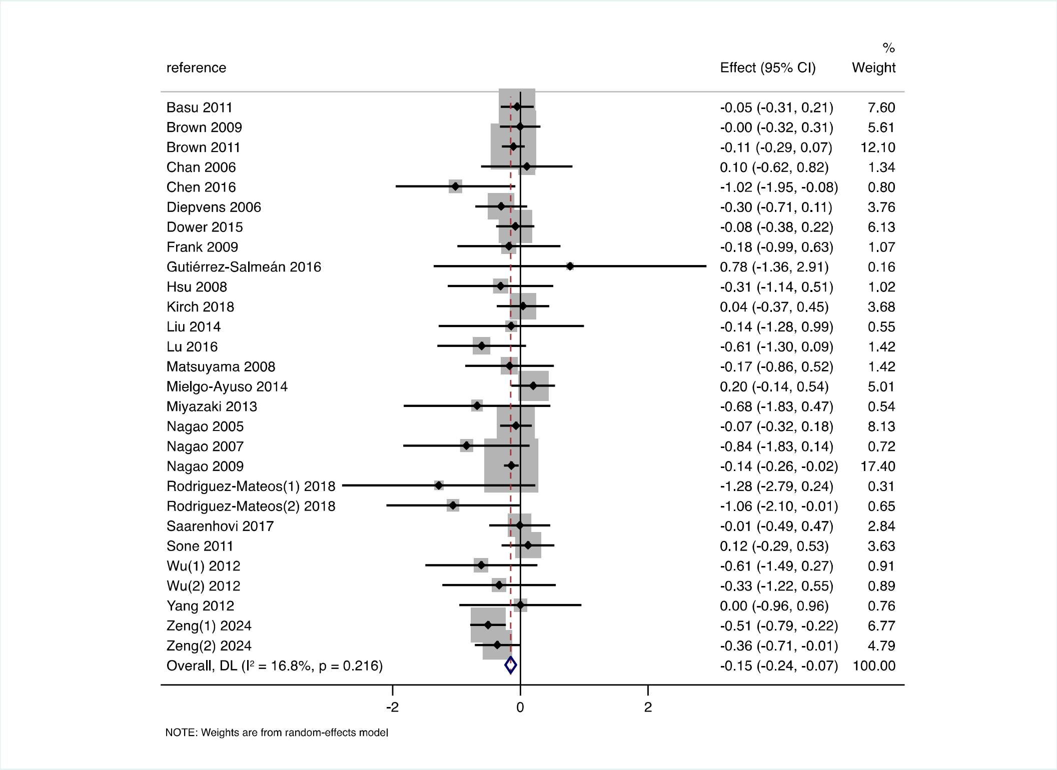

Figure S1-2-6 Forest plot of RCTs investigating the effect of catechin supplementation on TC.

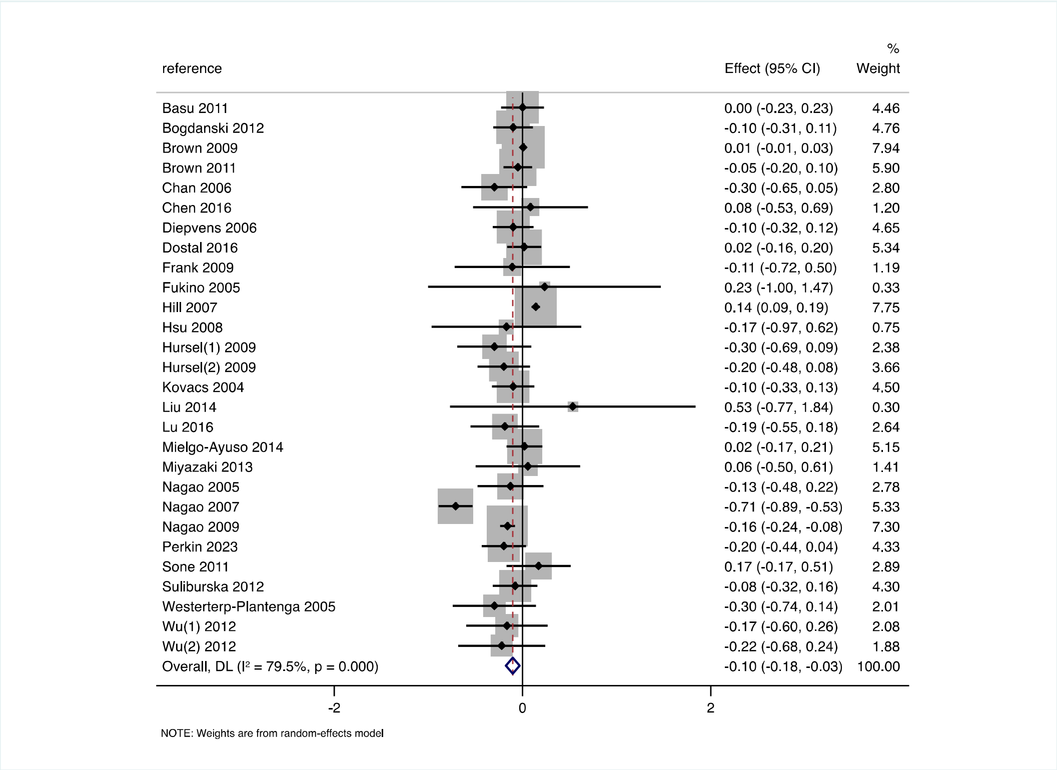

Figure S1-2-7 Forest plot of RCTs investigating the effect of catechin supplementation on FBG.

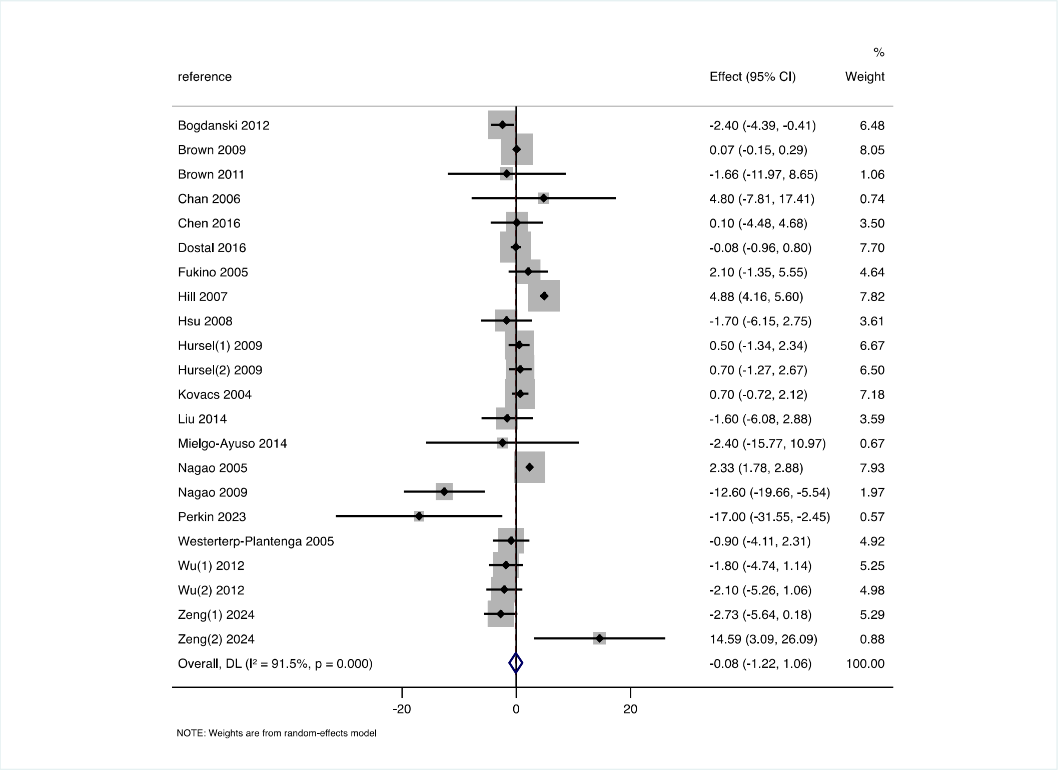

Figure S1-2-8 Forest plot of RCTs investigating the effect of catechin supplementation on FBI.

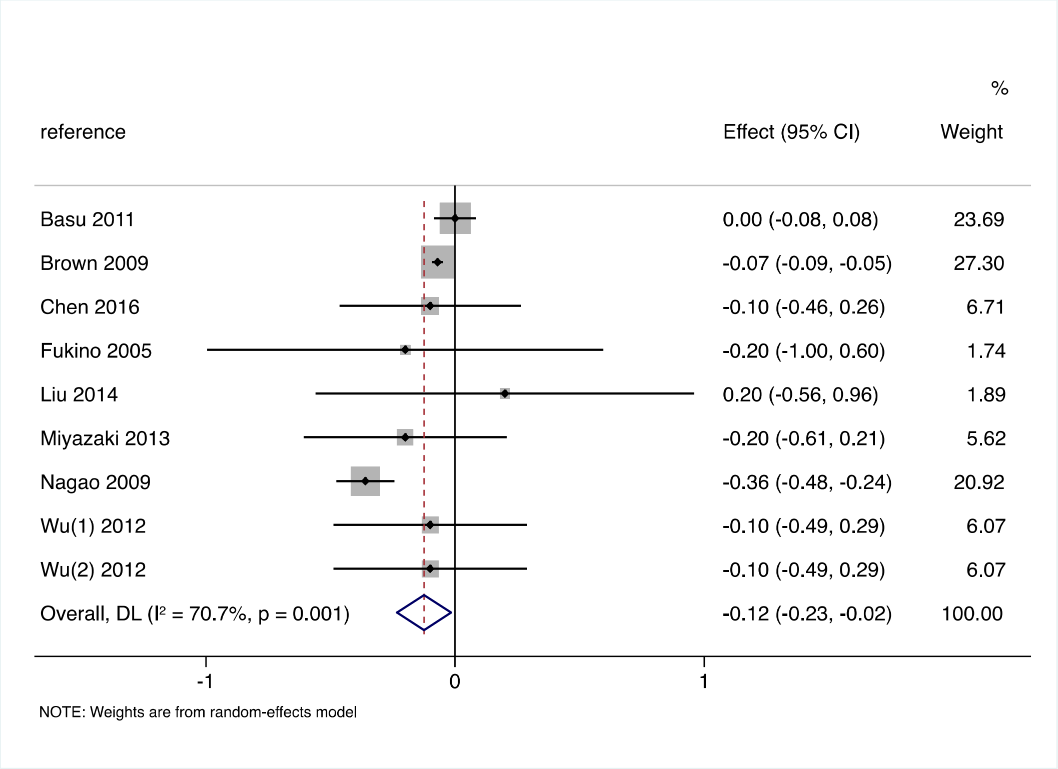

Figure S1-2-9 Forest plot of RCTs investigating the effect of catechin supplementation on A1C.

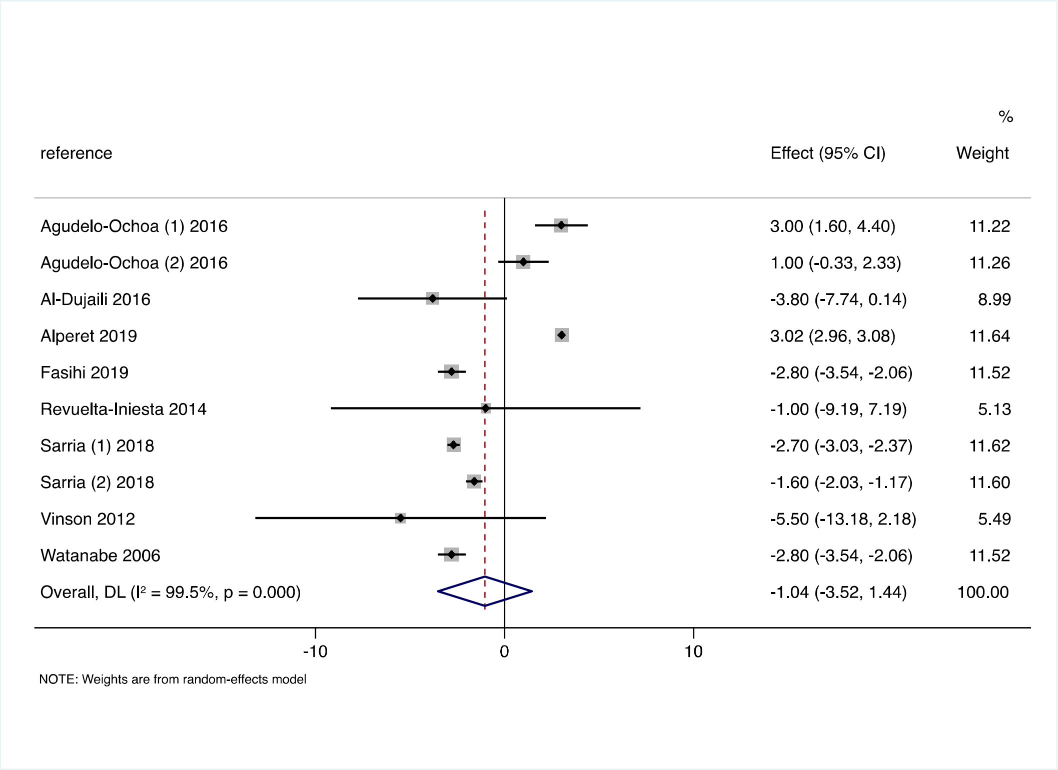

Figure S1-3-1 Forest plot of RCTs investigating the effect of chlorogenic acid supplementation on SBP.

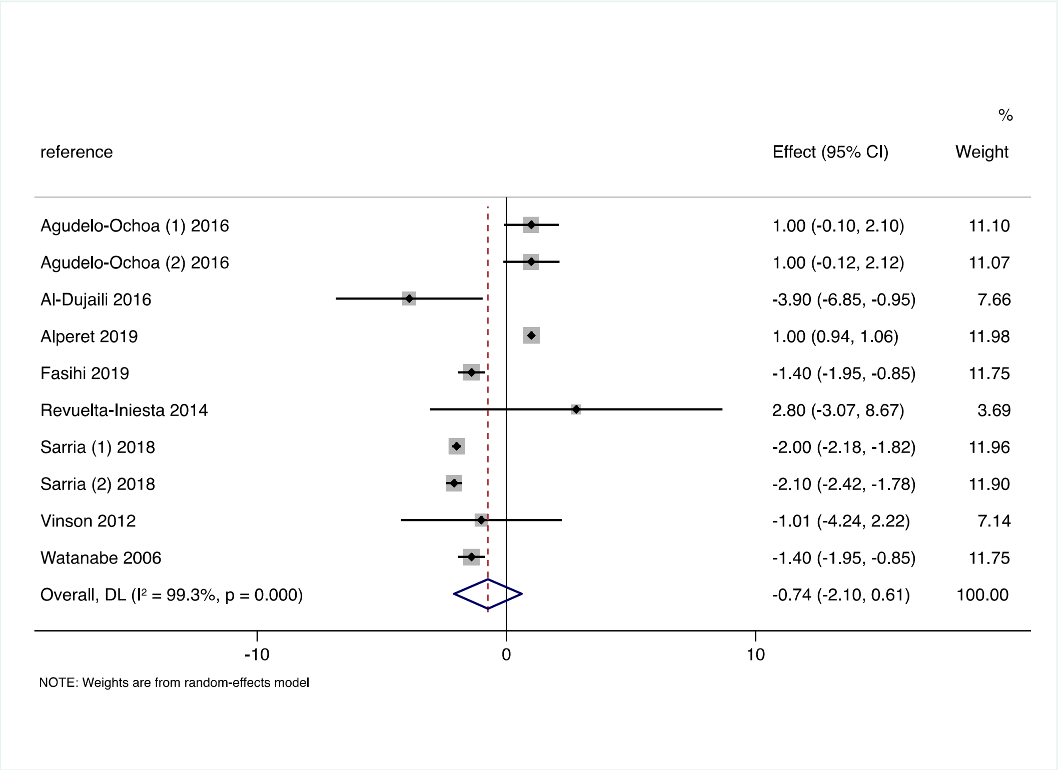

Figure S1-3-2 Forest plot of RCTs investigating the effect of chlorogenic acid supplementation on DBP.

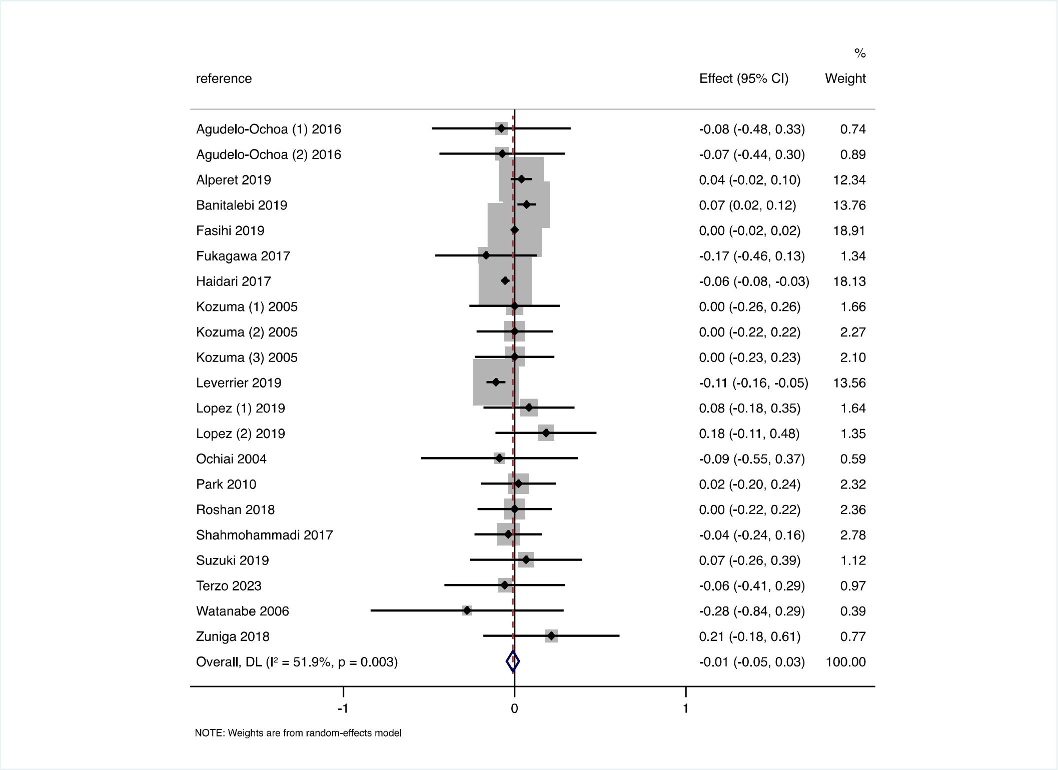

Figure S1-3-3 Forest plot of RCTs investigating the effect of chlorogenic acid supplementation on HDL-C.

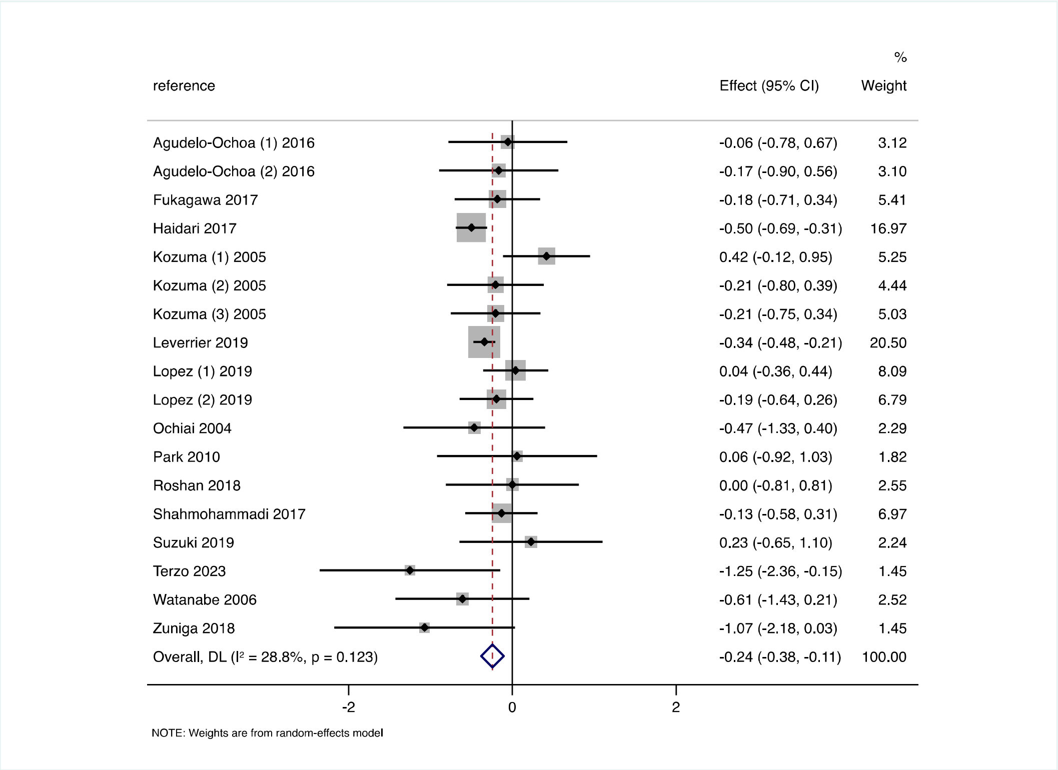

Figure S1-3-4 Forest plot of RCTs investigating the effect of chlorogenic acid supplementation on LDL-C.

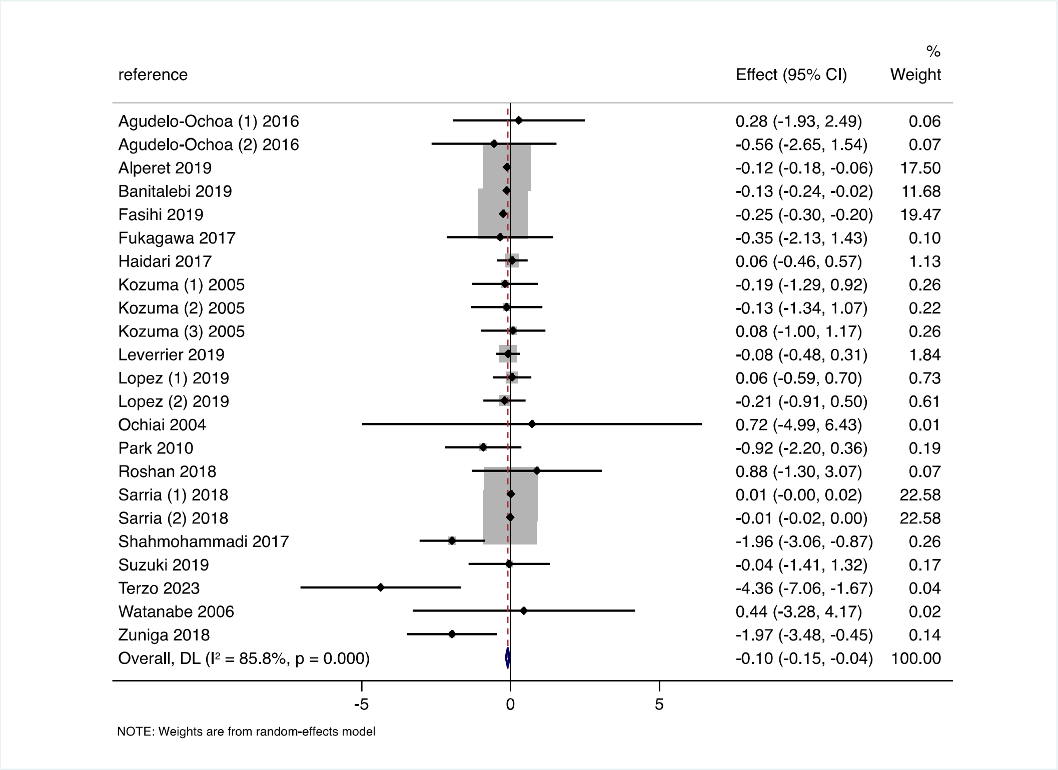

Figure S1-3-5 Forest plot of RCTs investigating the effect of chlorogenic acid supplementation on TG

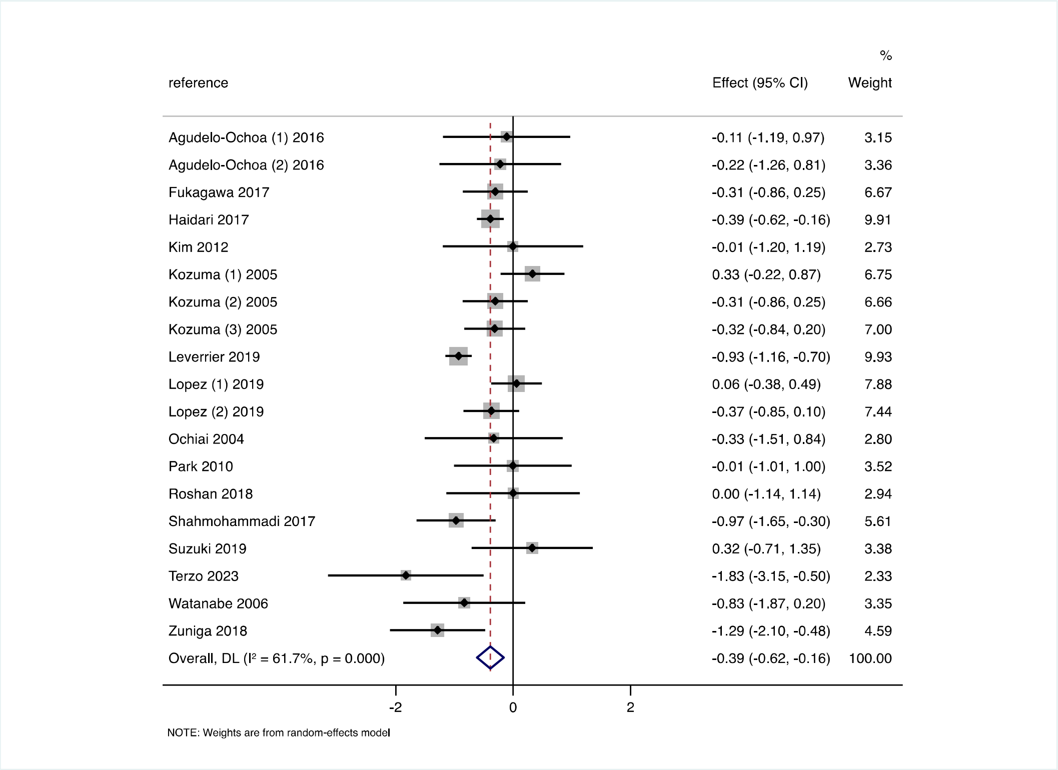

Figure S1-3-6 Forest plot of RCTs investigating the effect of chlorogenic acid supplementation on TC.

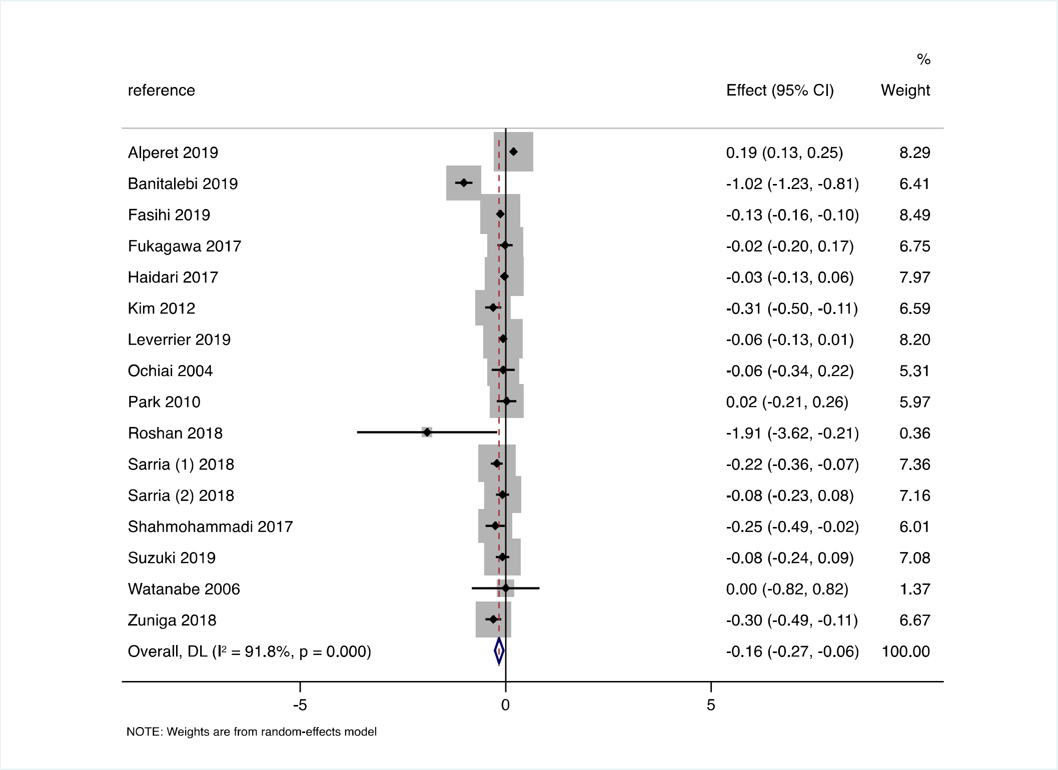

Figure S1-3-7 Forest plot of RCTs investigating the effect of chlorogenic acid supplementation on FBG.

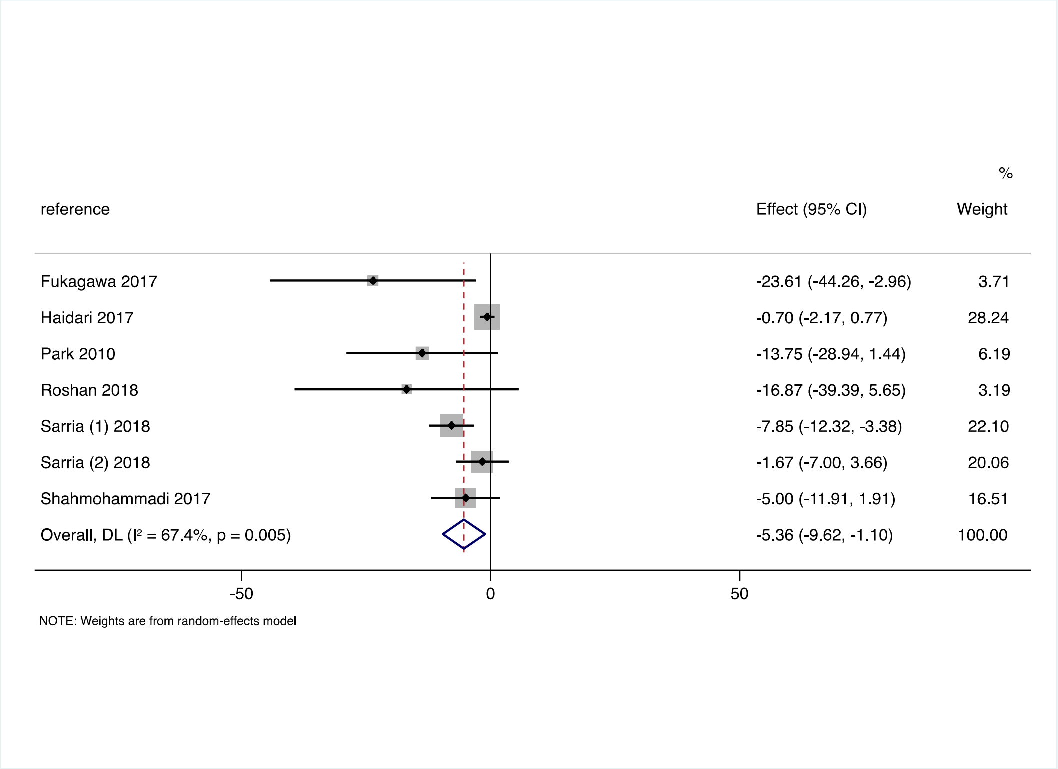

Figure S1-3-8 Forest plot of RCTs investigating the effect of chlorogenic acid supplementation on FBI.

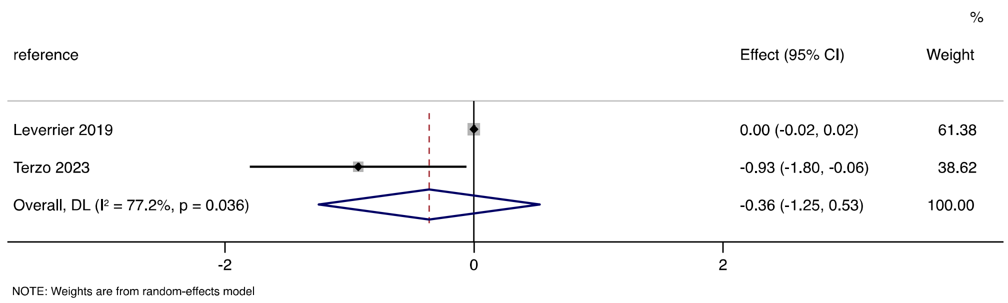

Figure S1-3-9 Forest plot of RCTs investigating the effect of chlorogenic acid supplementation on A1C.

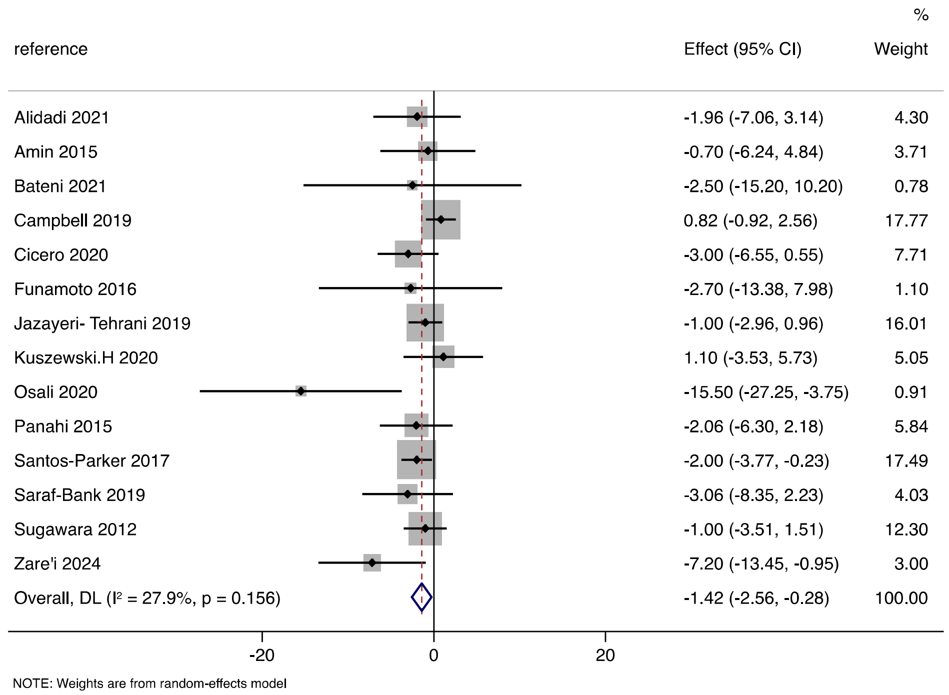

Figure S1-4-1 Forest plot of RCTs investigating the effect of curcumin supplementation on SBP.

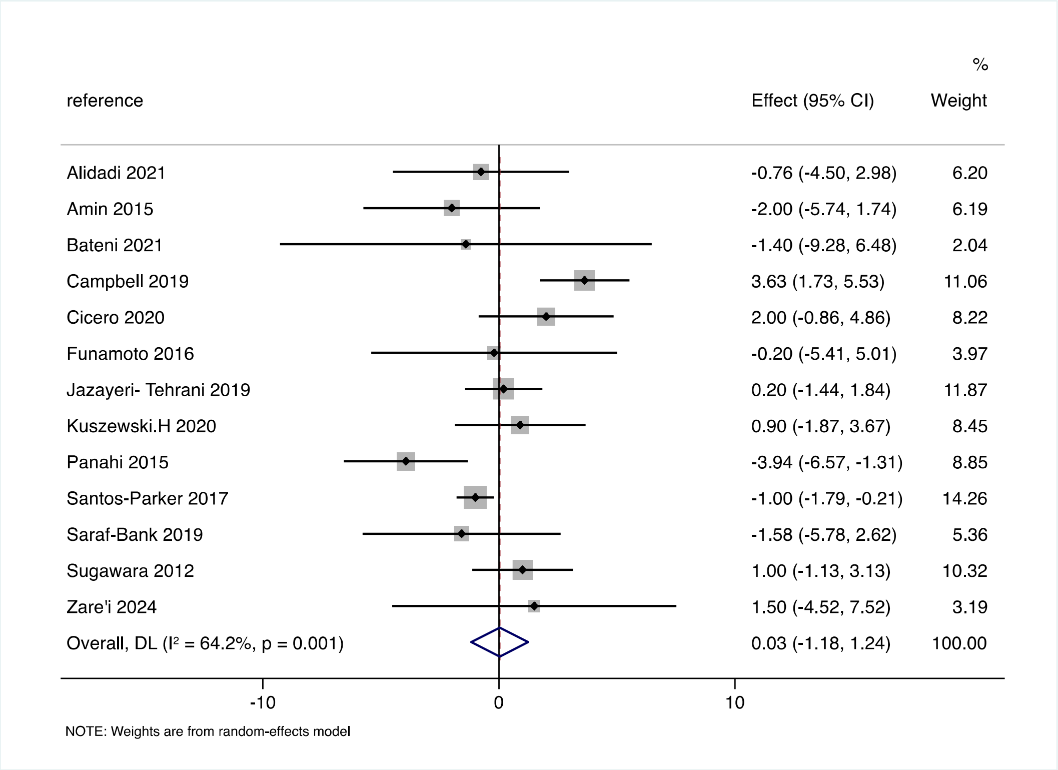

Figure S1-4-2 Forest plot of RCTs investigating the effect of curcumin supplementation on DBP.

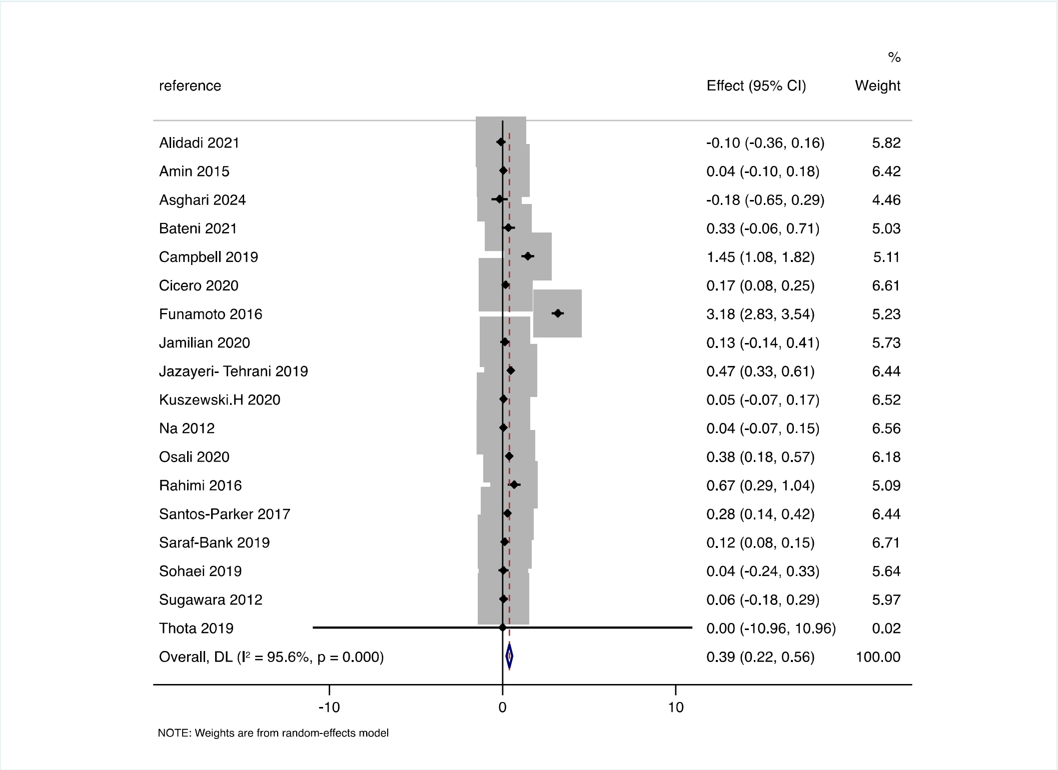

Figure S1-4-3 Forest plot of RCTs investigating the effect of curcumin supplementation on HDL-C.

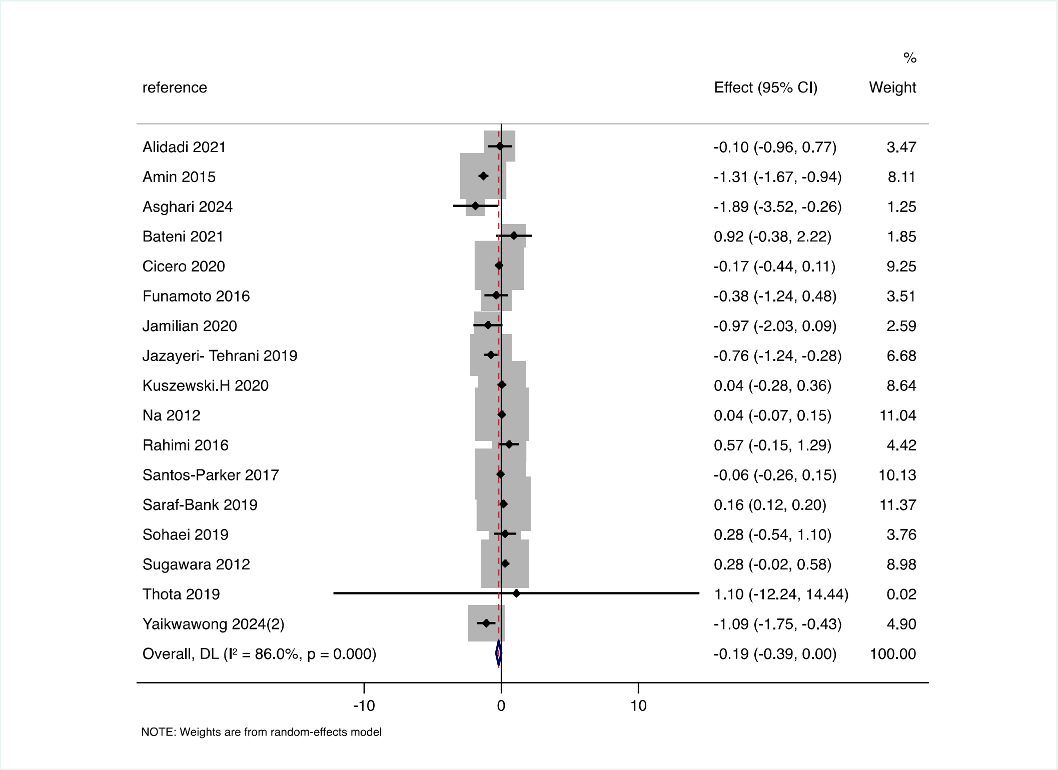

Figure S1-4-4 Forest plot of RCTs investigating the effect of curcumin supplementation on LDL-C.

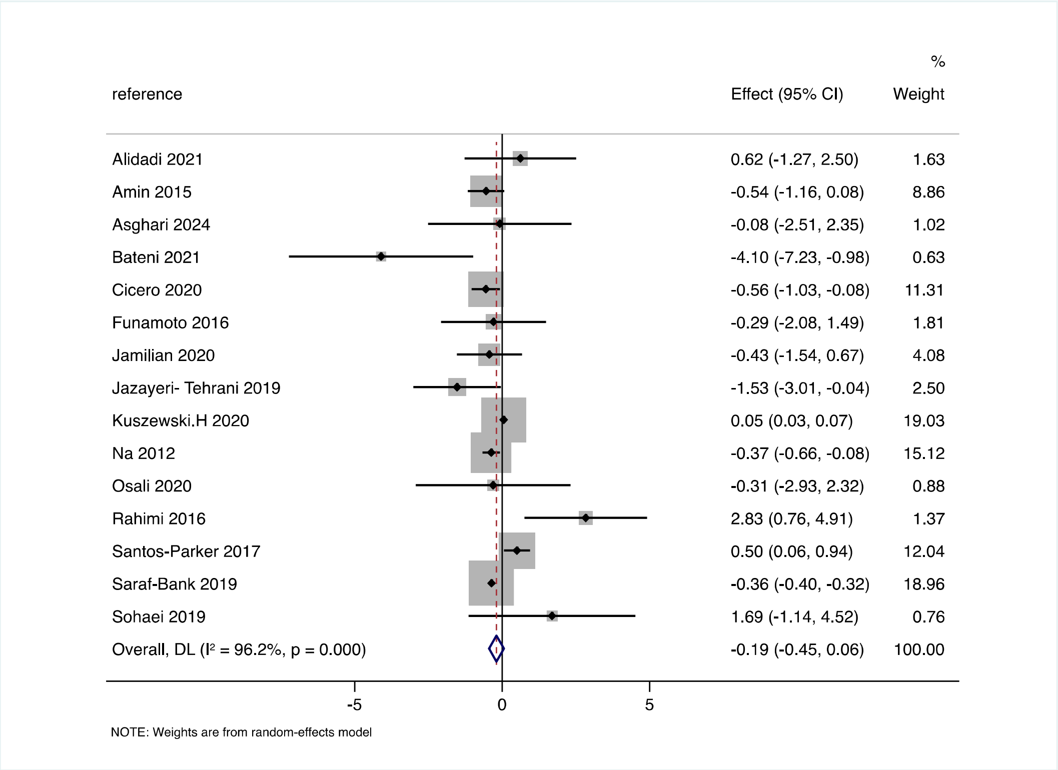

Figure S1-4-5 Forest plot of RCTs investigating the effect of curcumin supplementation on TG

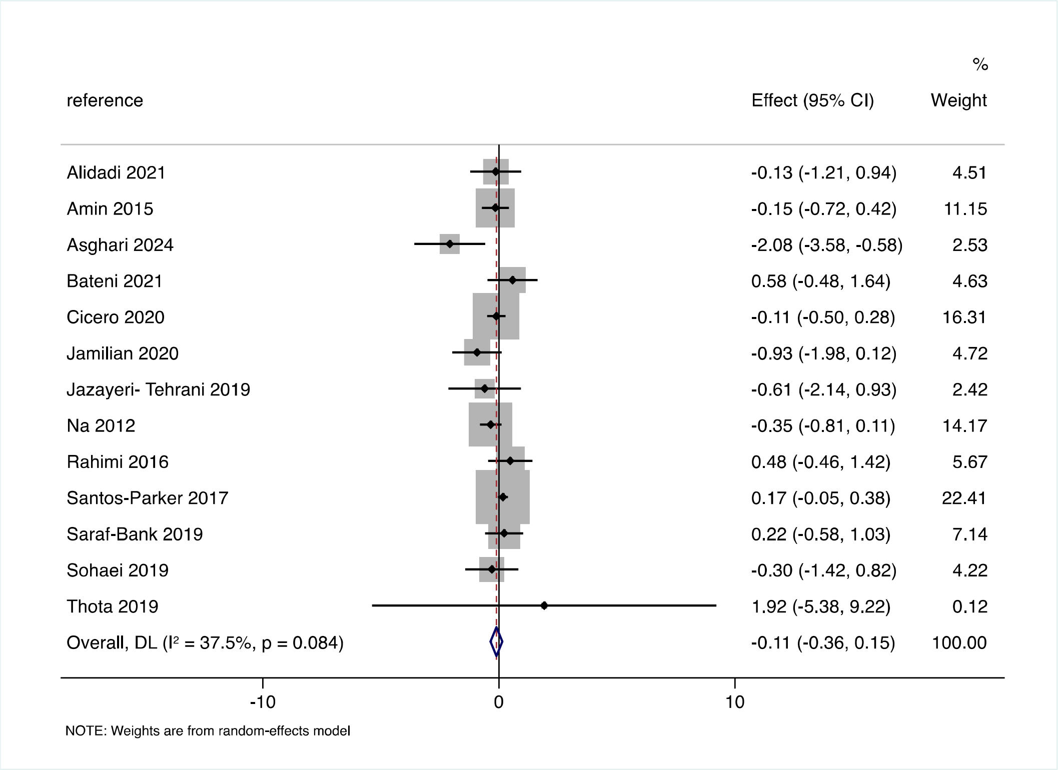

Figure S1-4-6 Forest plot of RCTs investigating the effect of curcumin supplementation on TC.

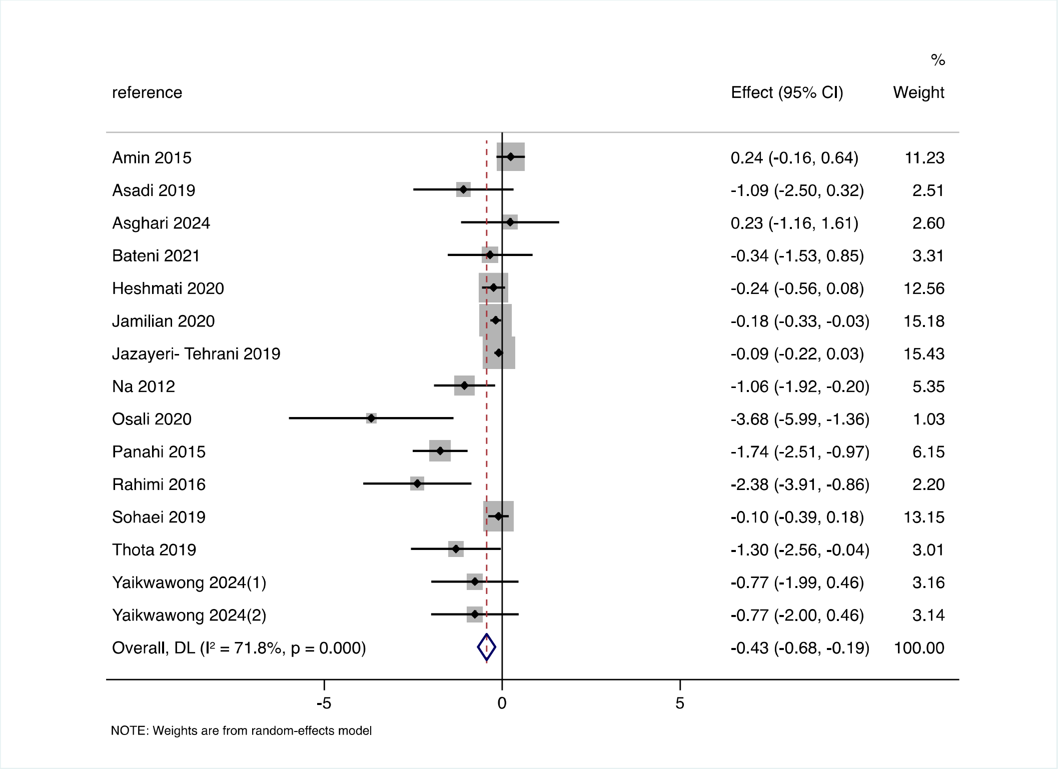

Figure S1-4-7 Forest plot of RCTs investigating the effect of curcumin supplementation on FBG.

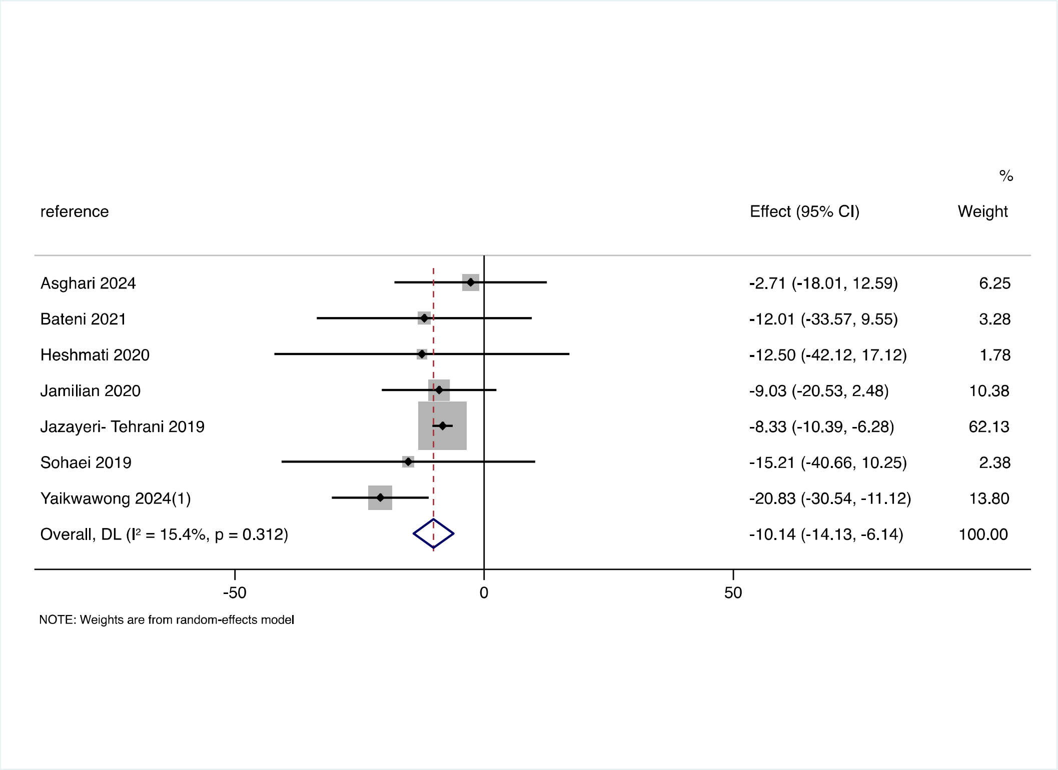

Figure S1-4-8 Forest plot of RCTs investigating the effect of curcumin supplementation on FBI.

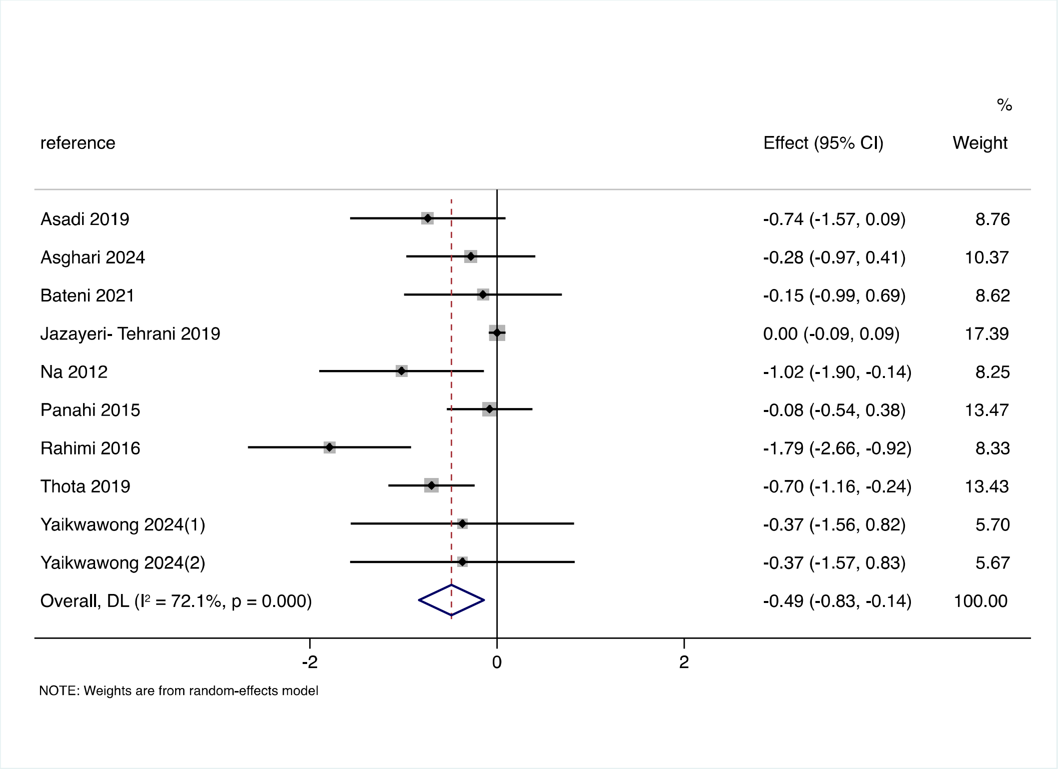

Figure S1-4-9 Forest plot of RCTs investigating the effect of curcumin supplementation on A1C.

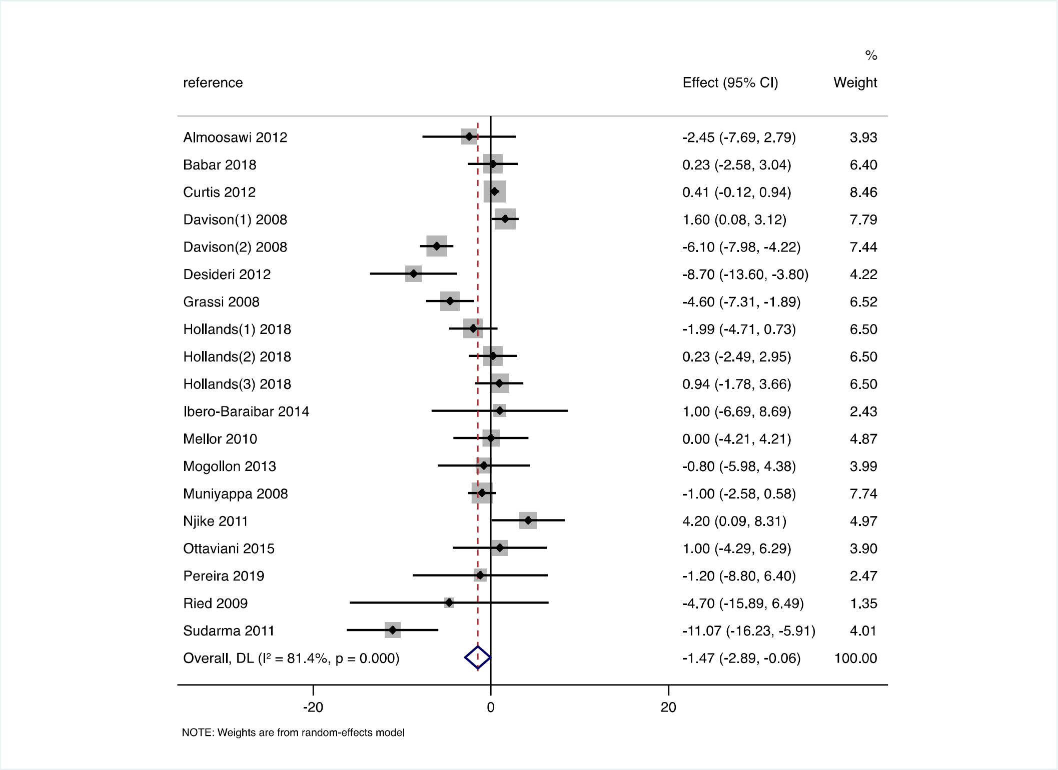

Figure S1-5-1 Forest plot of RCTs investigating the effect of flavanol supplementation on SBP.

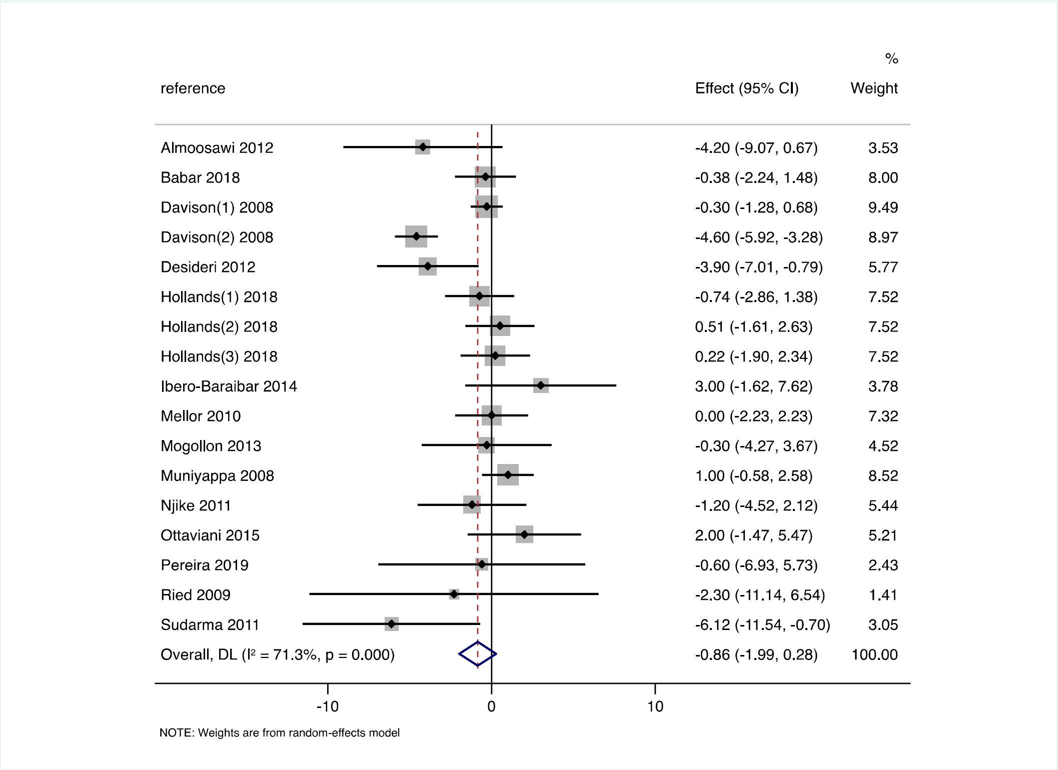

Figure S1-5-2 Forest plot of RCTs investigating the effect of flavanol supplementation on DBP.

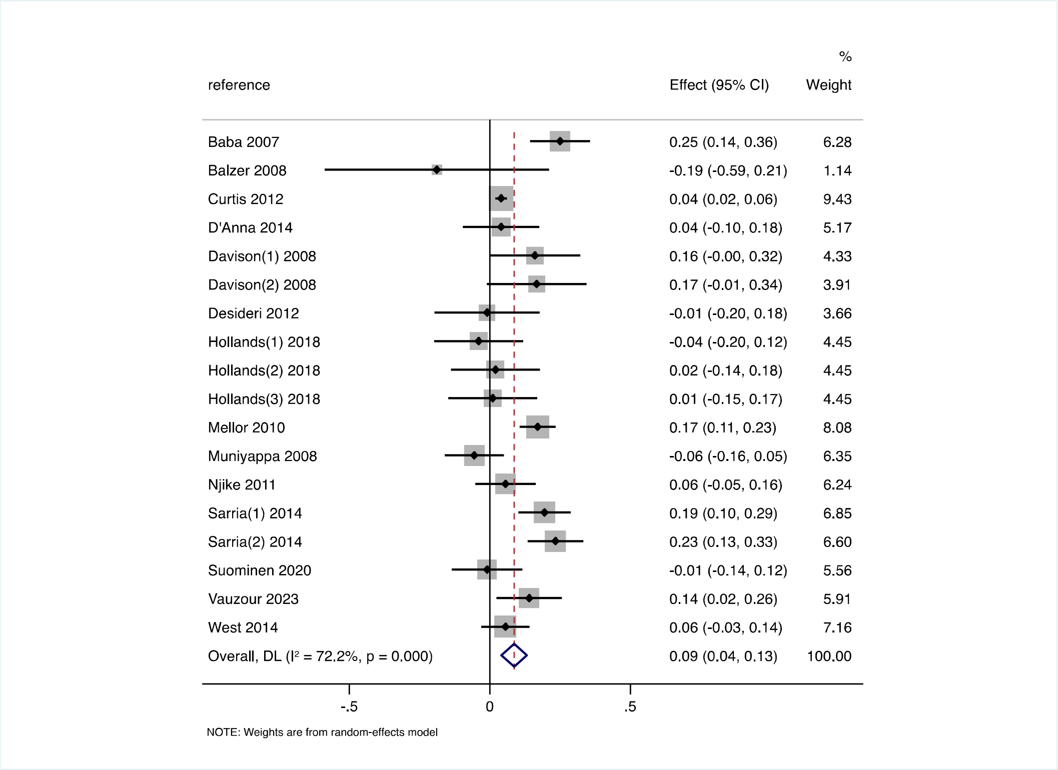

Figure S1-5-3 Forest plot of RCTs investigating the effect of flavanol supplementation on HDL-C.

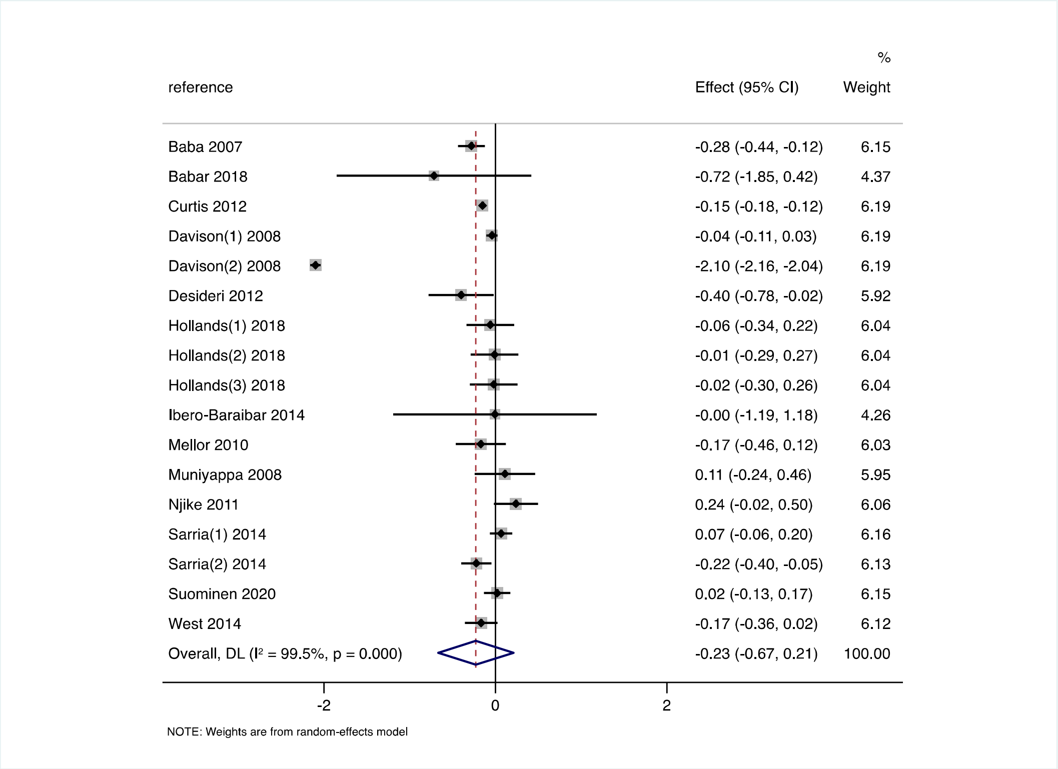

Figure S1-5-4 Forest plot of RCTs investigating the effect of flavanol supplementation on LDL-C.

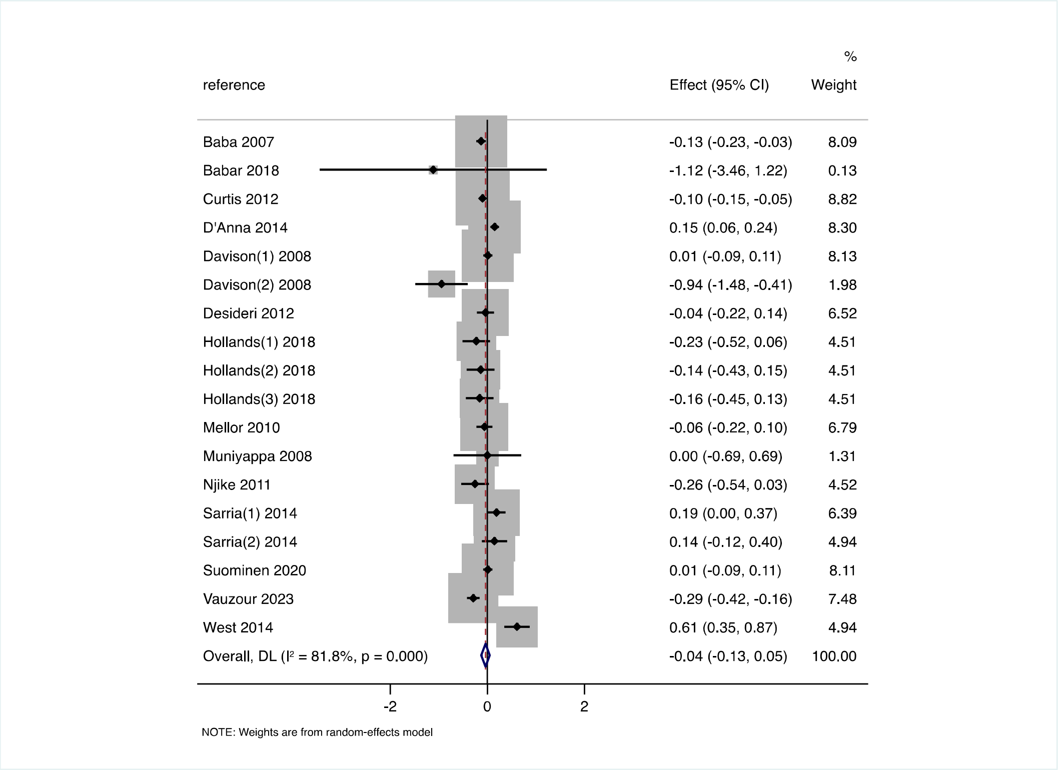

Figure S1-5-5 Forest plot of RCTs investigating the effect of flavanol supplementation on TG

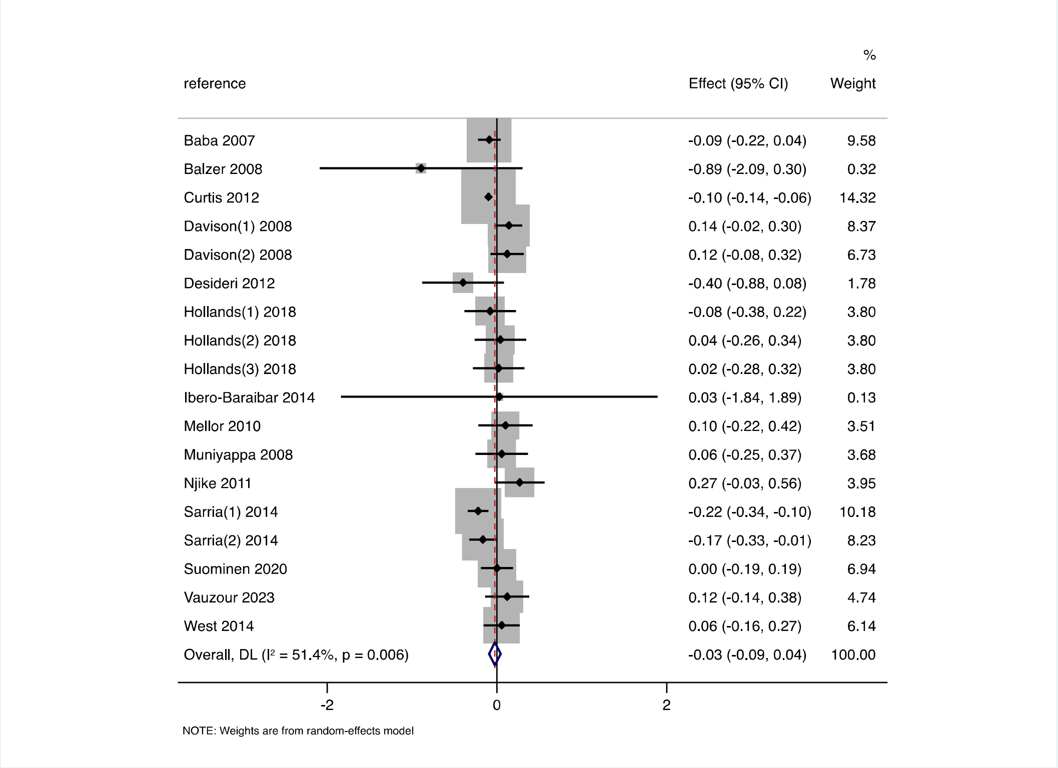

Figure S1-5-6 Forest plot of RCTs investigating the effect of flavanol supplementation on TC.

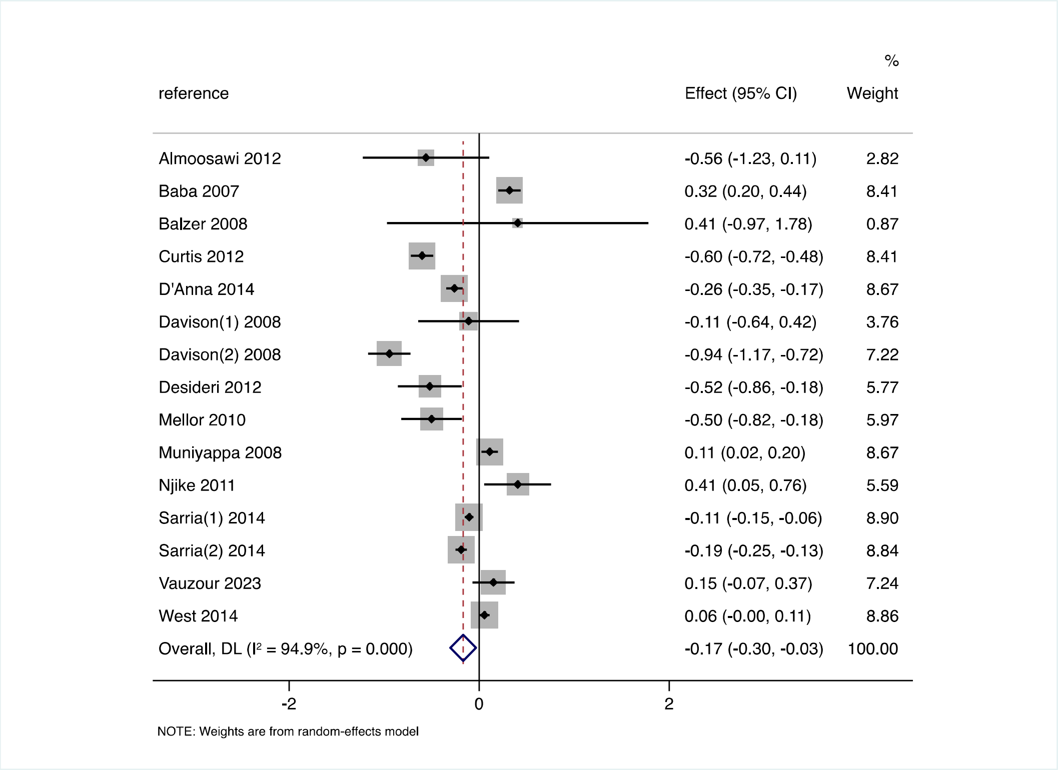

Figure S1-5-7 Forest plot of RCTs investigating the effect of flavanol supplementation on FBG.

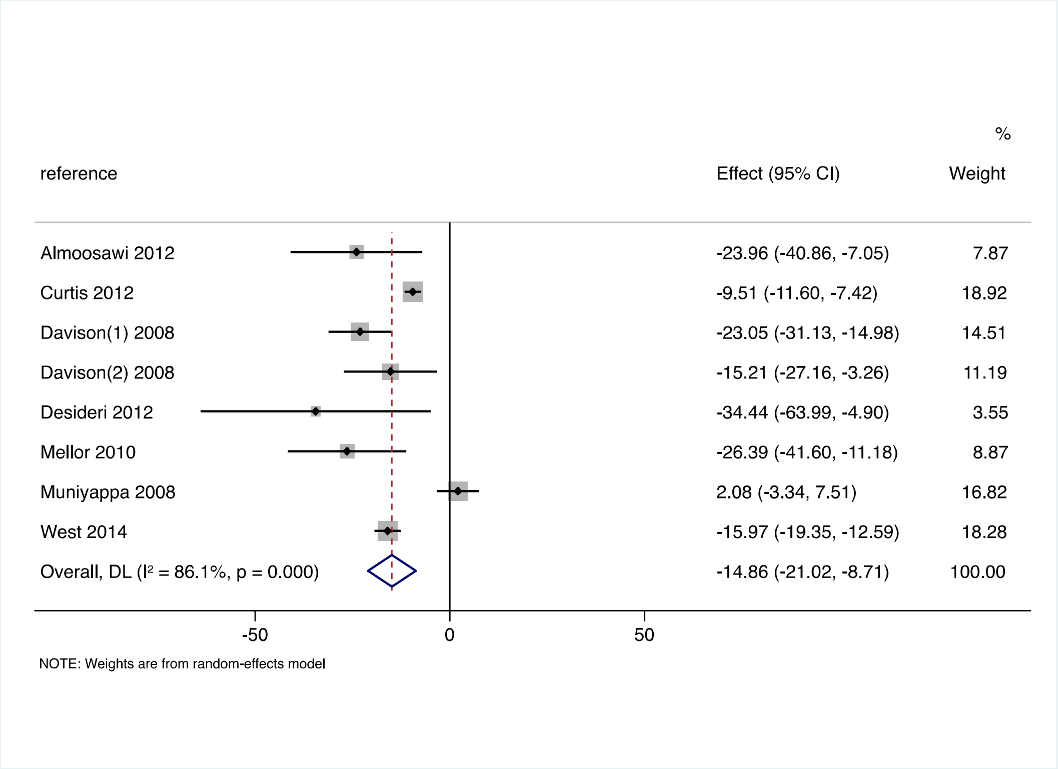

Figure S1-5-8 Forest plot of RCTs investigating the effect of flavanol supplementation on FBI.

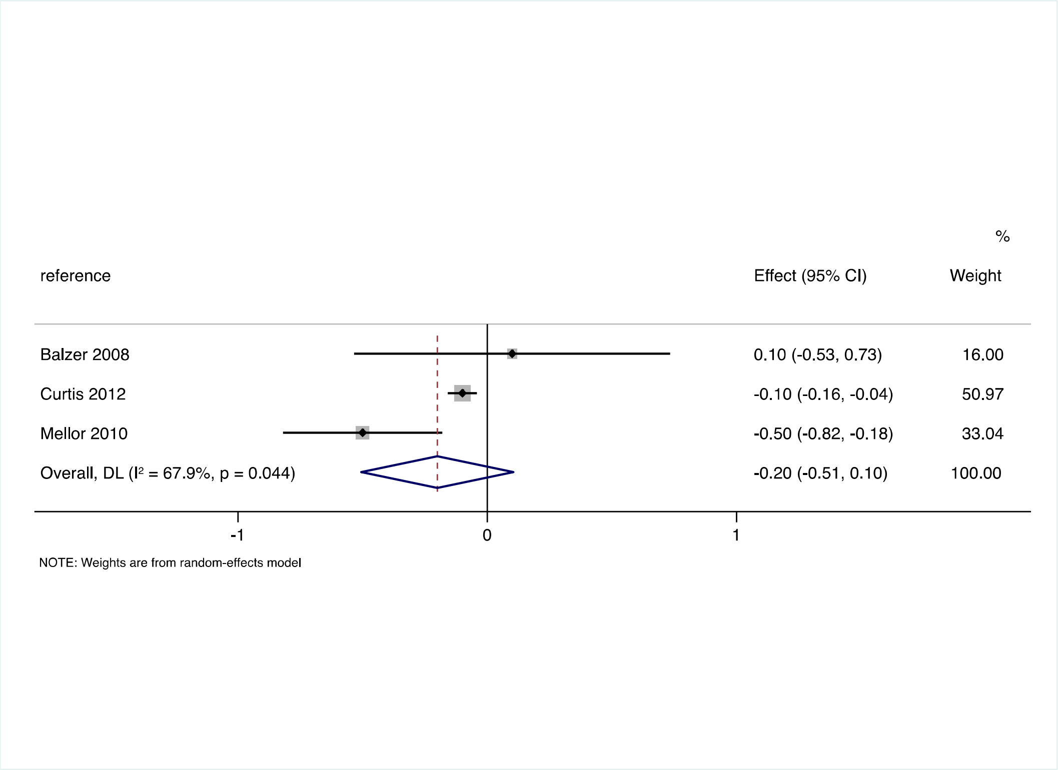

Figure S1-5-9 Forest plot of RCTs investigating the effect of flavanol supplementation on A1C.

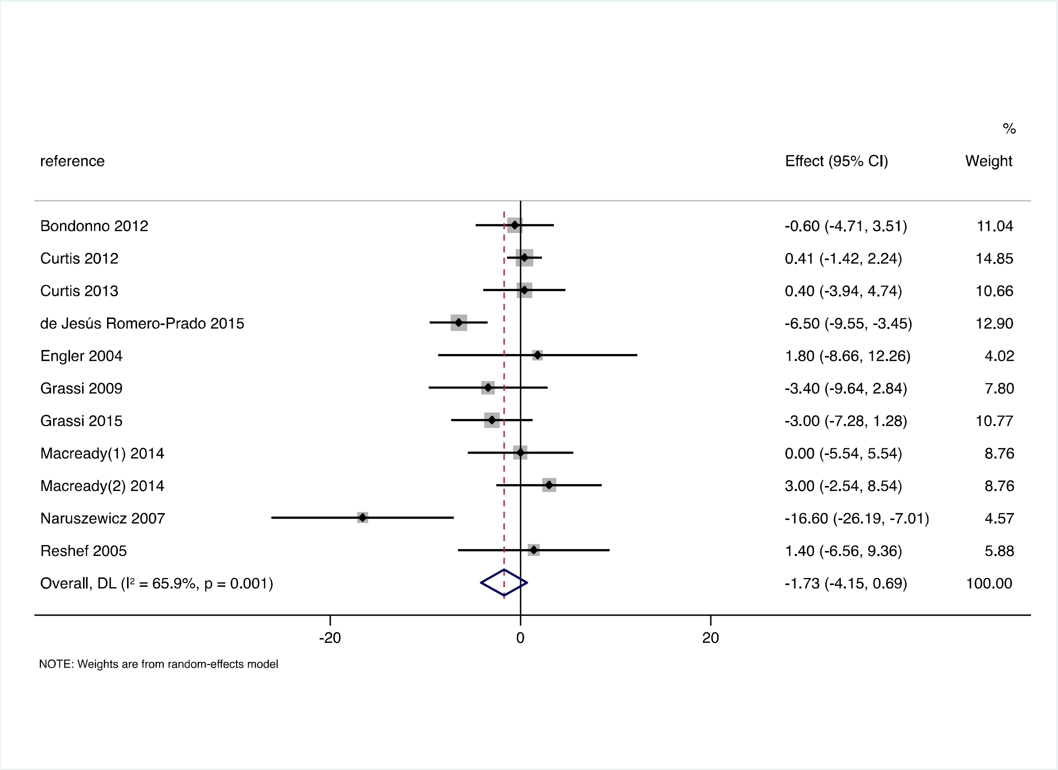

Figure S1-6-1 Forest plot of RCTs investigating the effect of flavonoid supplementation on SBP.

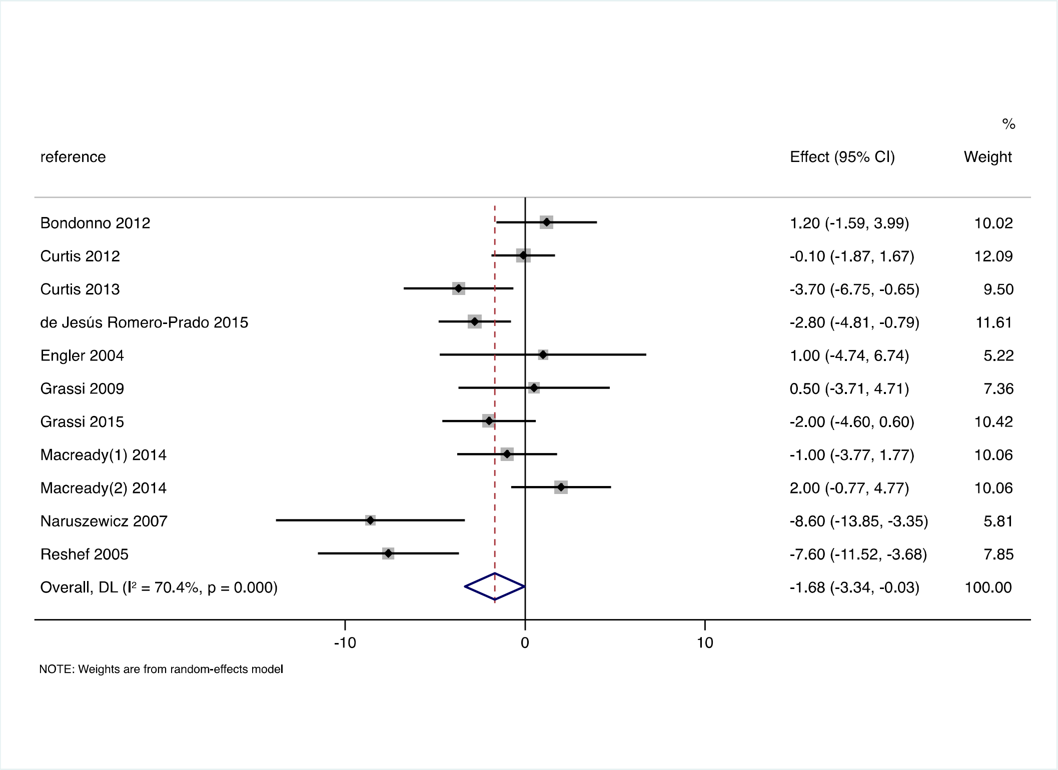

Figure S1-6-2 Forest plot of RCTs investigating the effect of flavonoid supplementation on DBP.

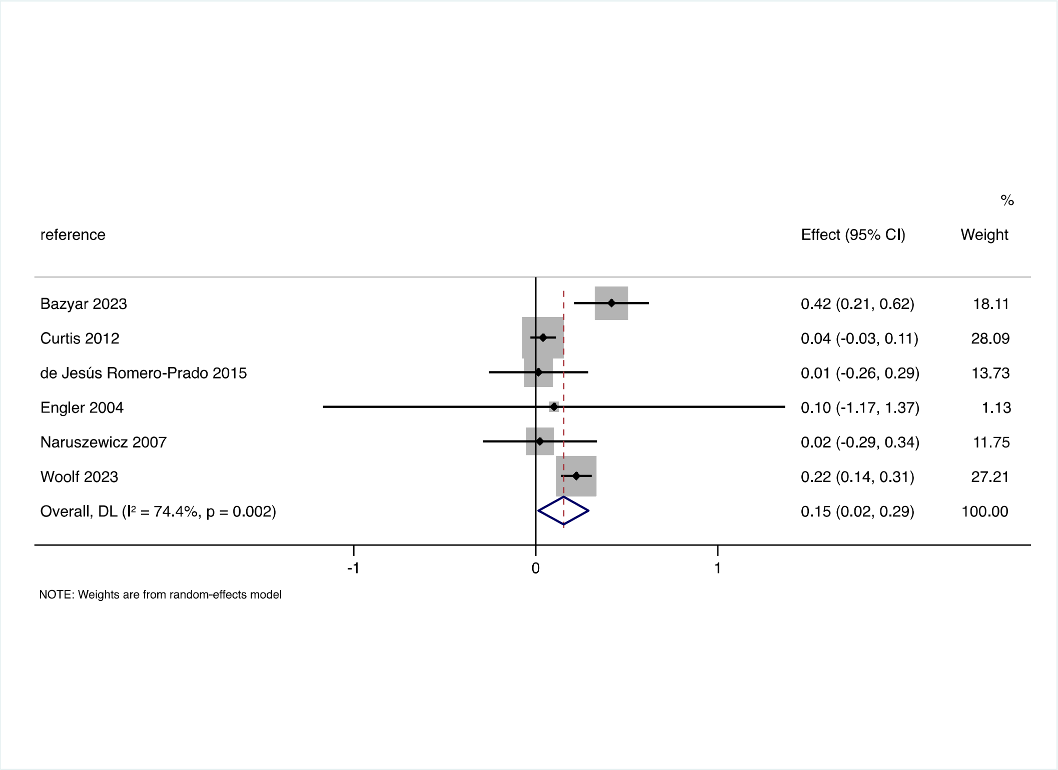

Figure S1-6-3 Forest plot of RCTs investigating the effect of flavonoid supplementation on HDL-C

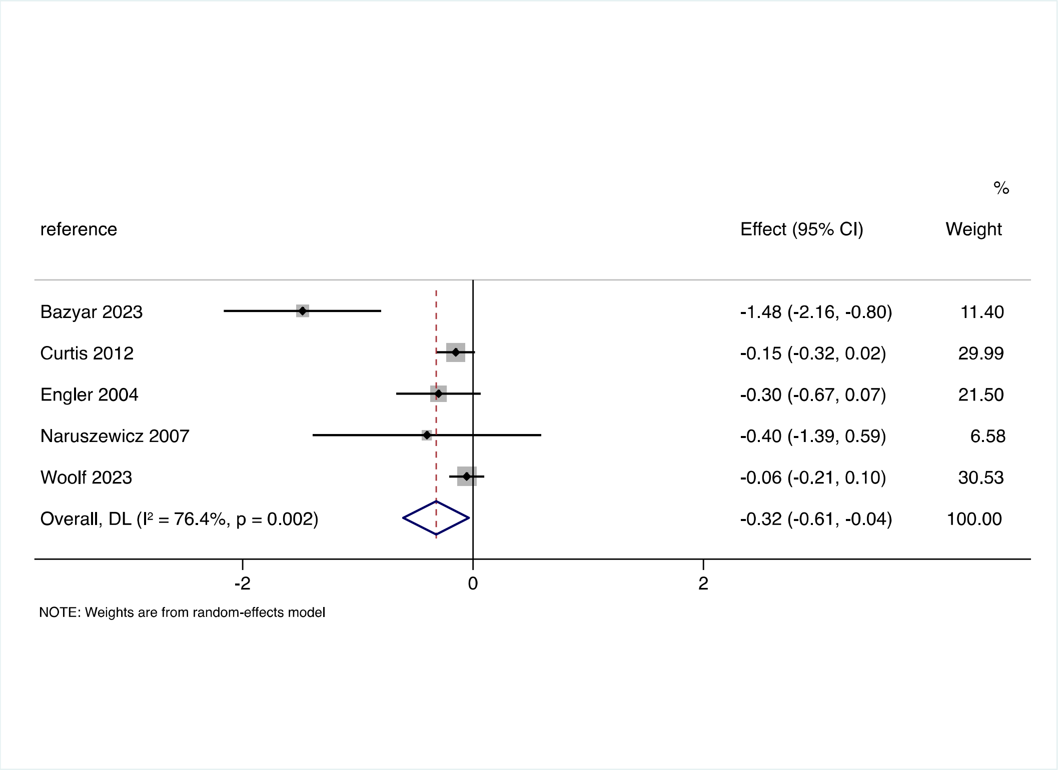

Figure S1-6-4 Forest plot of RCTs investigating the effect of flavonoid supplementation on LDL-C.

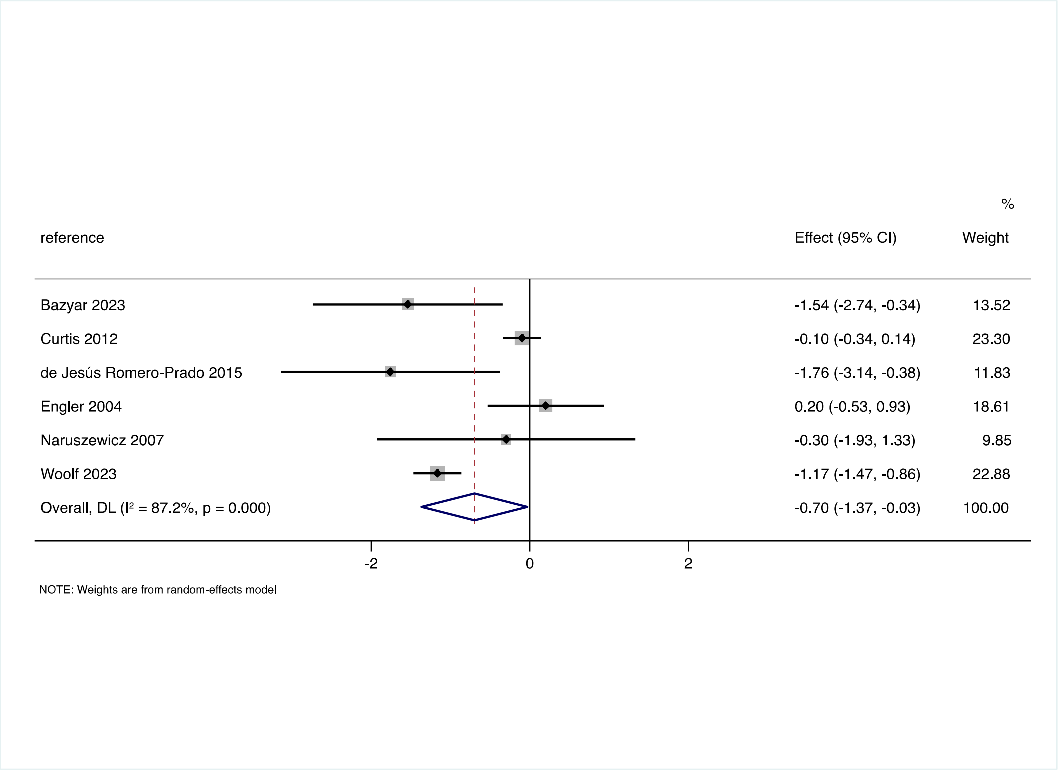

Figure S1-6-5 Forest plot of RCTs investigating the effect of flavonoid supplementation on TG

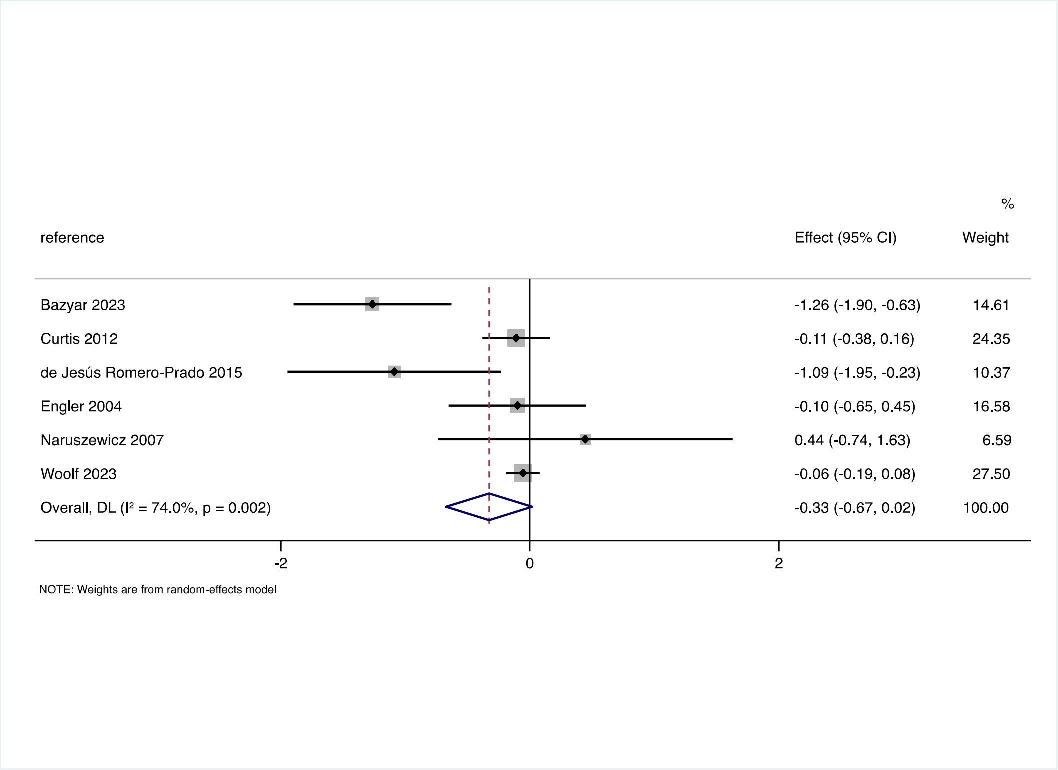

Figure S1-6-6 Forest plot of RCTs investigating the effect of flavonoid supplementation on TC.

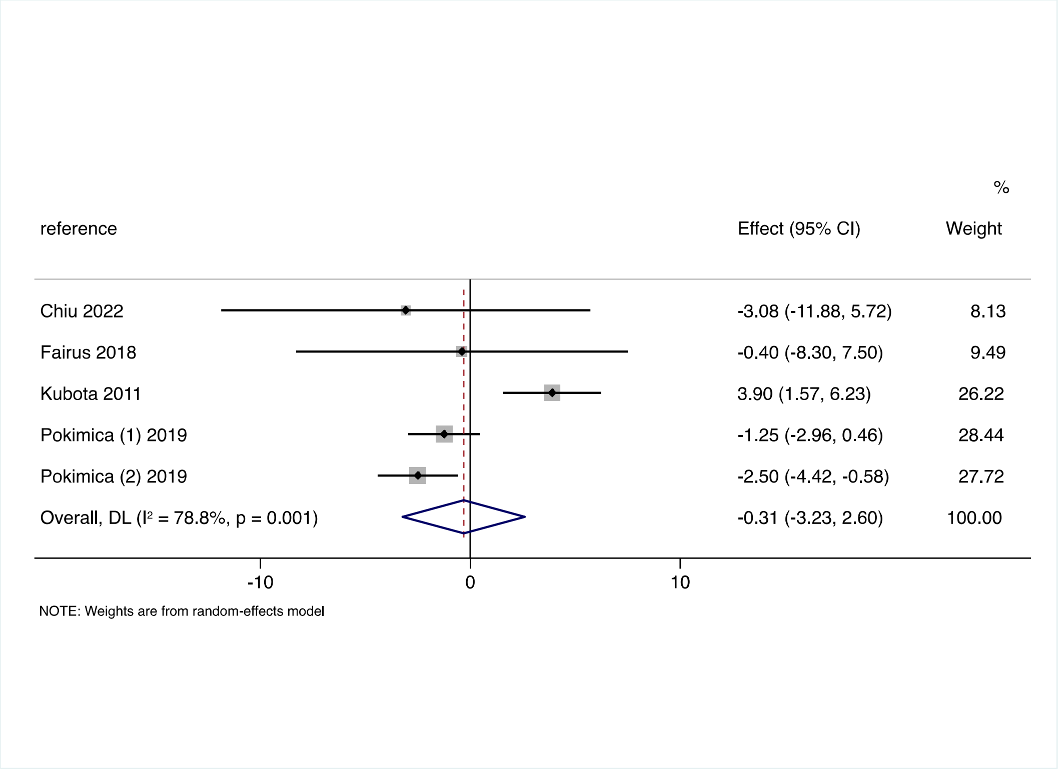

FIgure S1-7-1 Forest plot of RCTs investigating the effect of gallic acid supplementation on SBP.

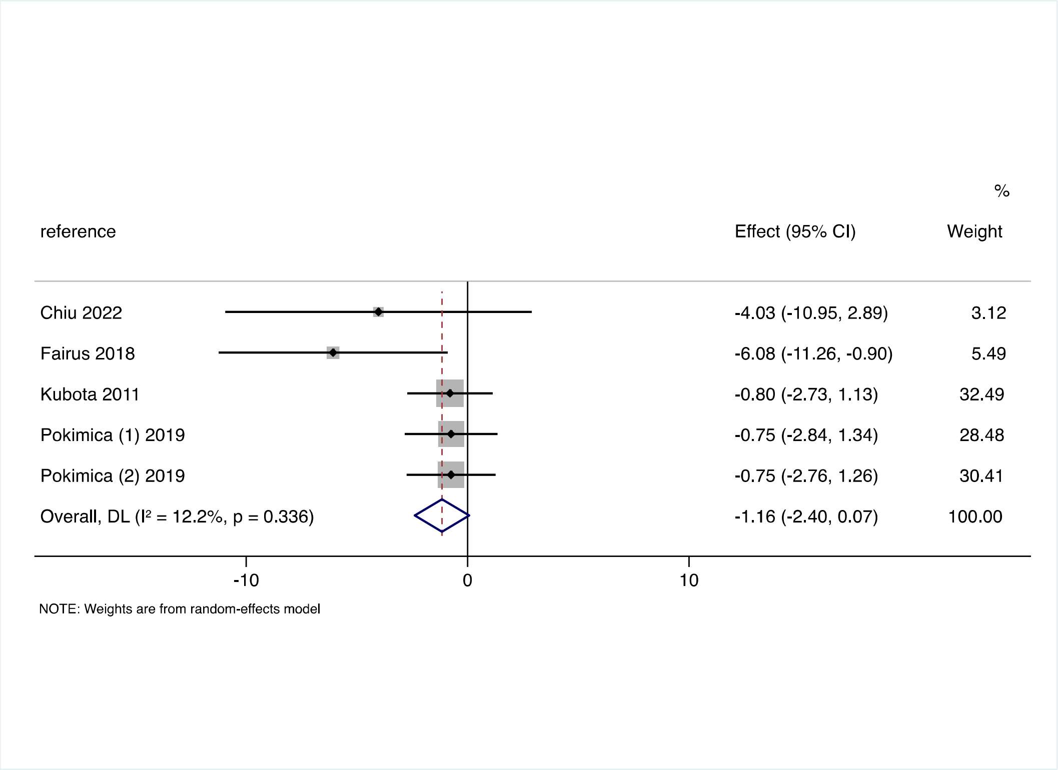

FIgure S1-7-2 Forest plot of RCTs investigating the effect of gallic acid supplementation on DBP.

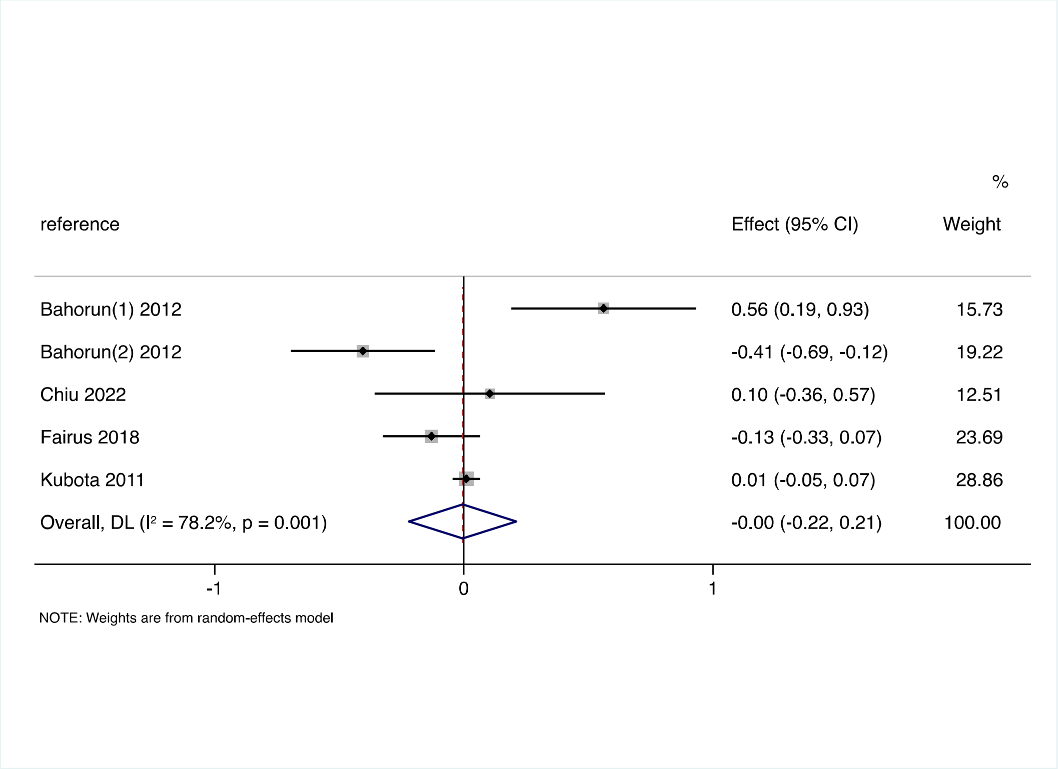

FIgure S1-7-3 Forest plot of RCTs investigating the effect of gallic acid supplementation on HDL-C.

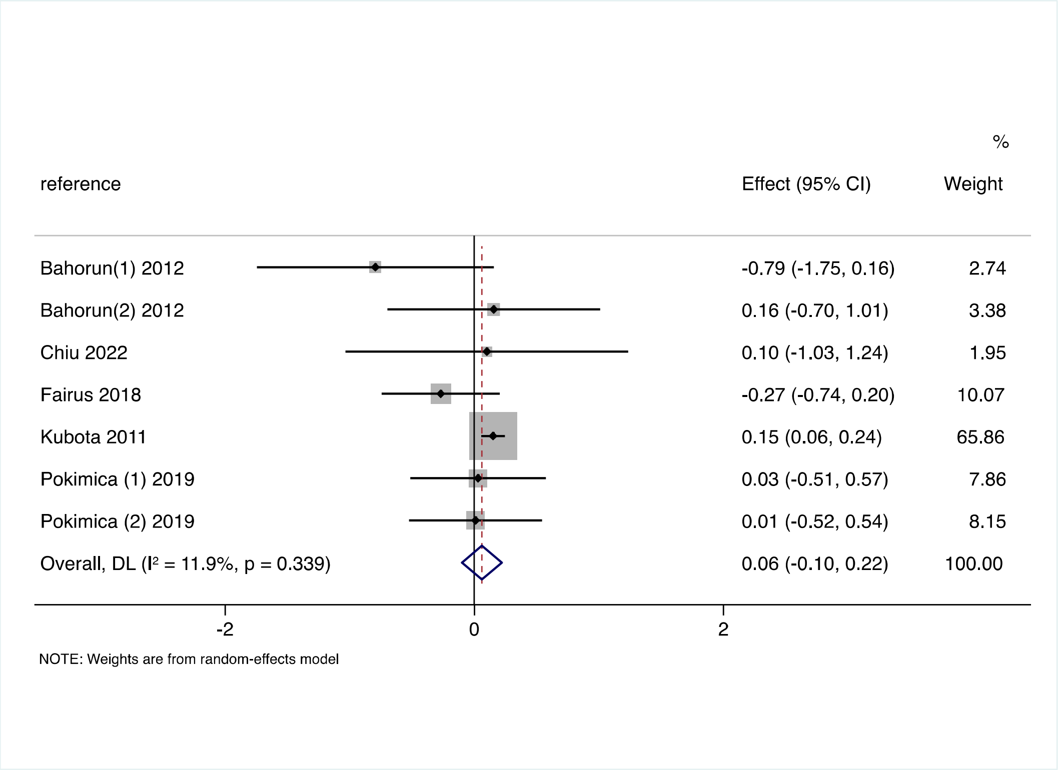

FIgure S1-7-4 Forest plot of RCTs investigating the effect of gallic acid supplementation on LDL-C.

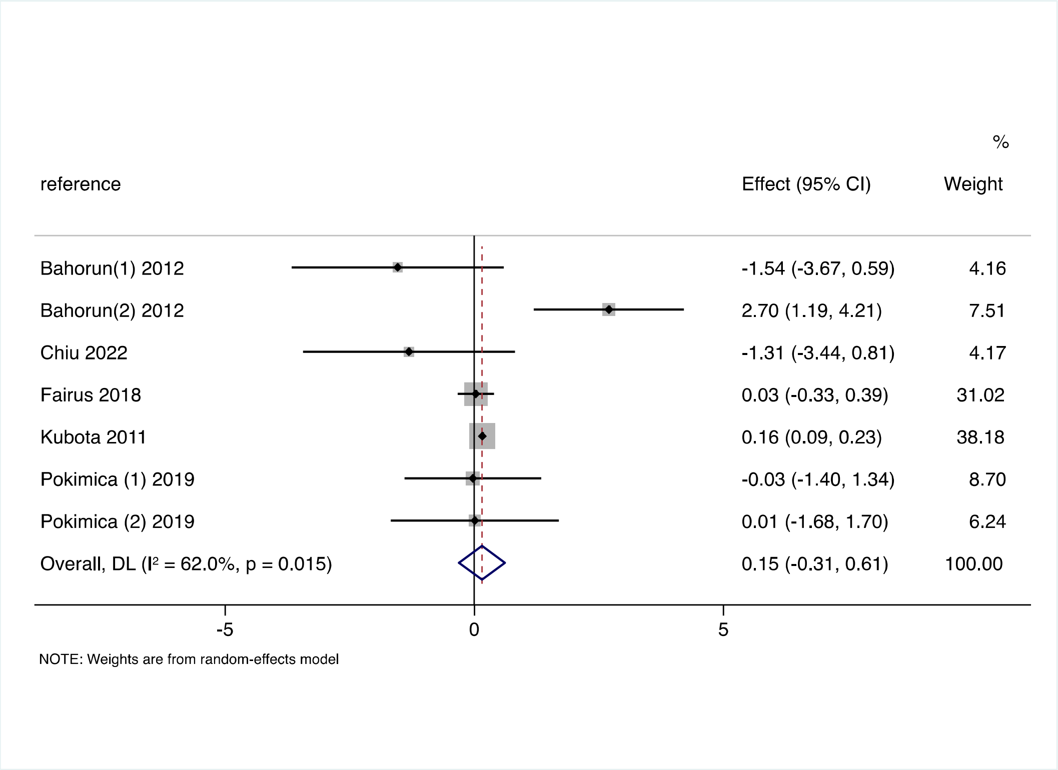

FIgure S1-7-5 Forest plot of RCTs investigating the effect of gallic acid supplementation on TG

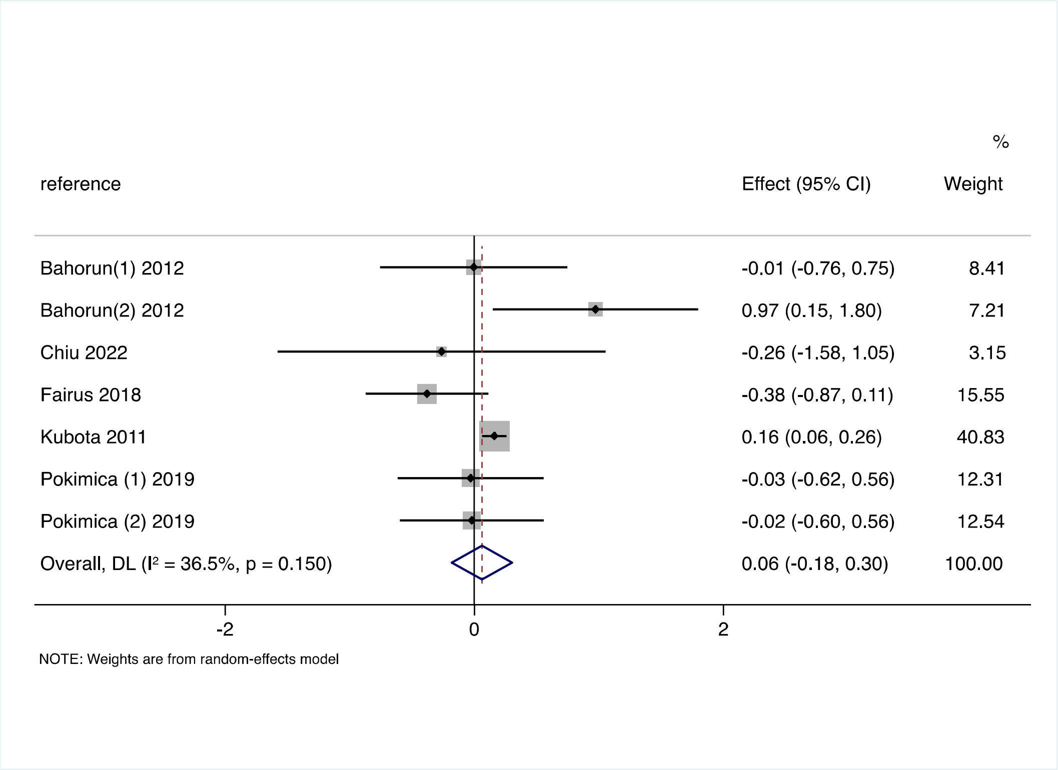

FIgure S1-7-6 Forest plot of RCTs investigating the effect of gallic acid supplementation on TC.

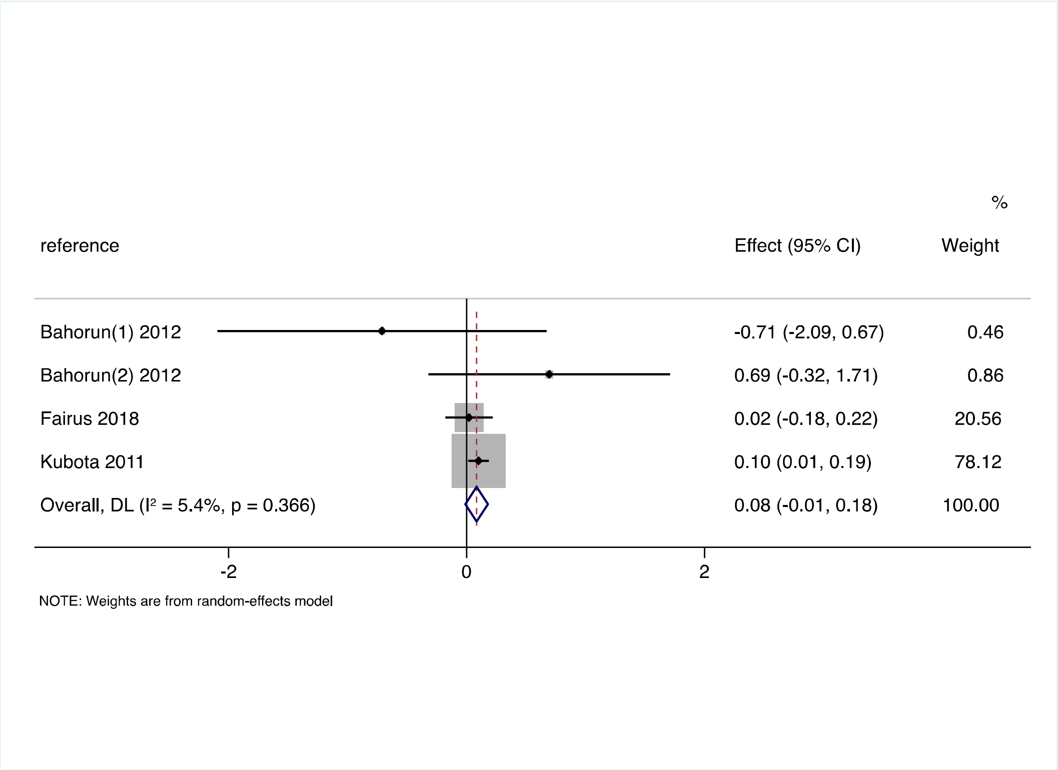

FIgure S1-7-7 Forest plot of RCTs investigating the effect of gallic acid supplementation on FBG.

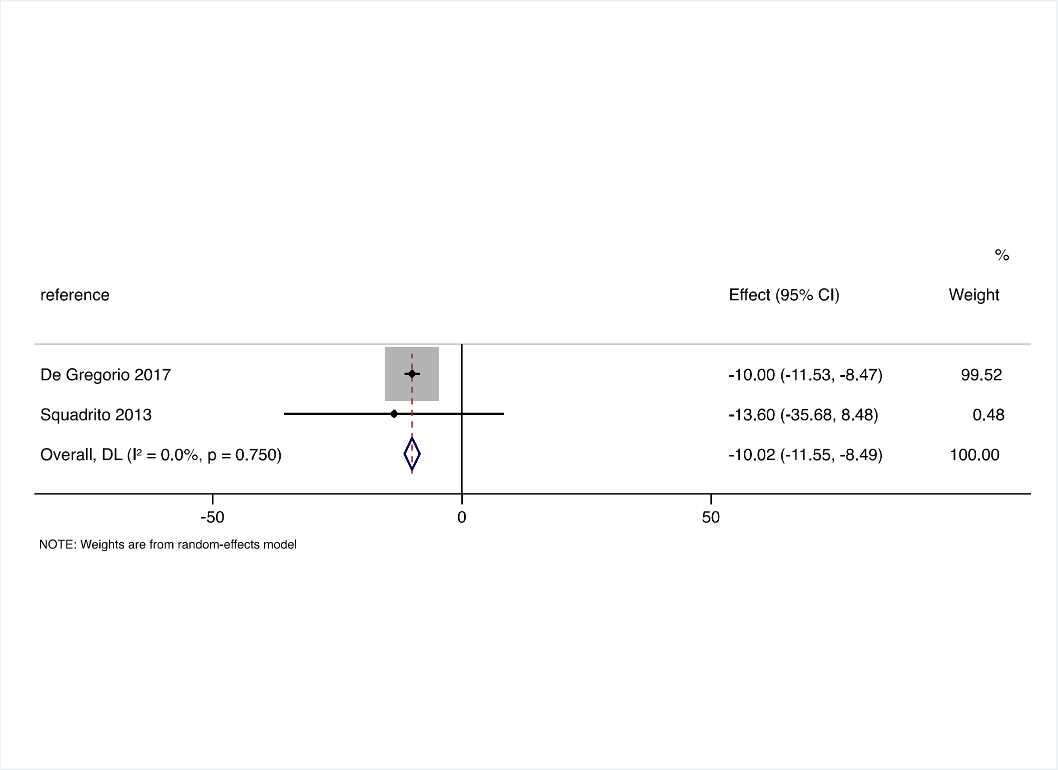

Figure S1-8-1 Forest plot of RCTs investigating the effect of genistein supplementation on SBP.

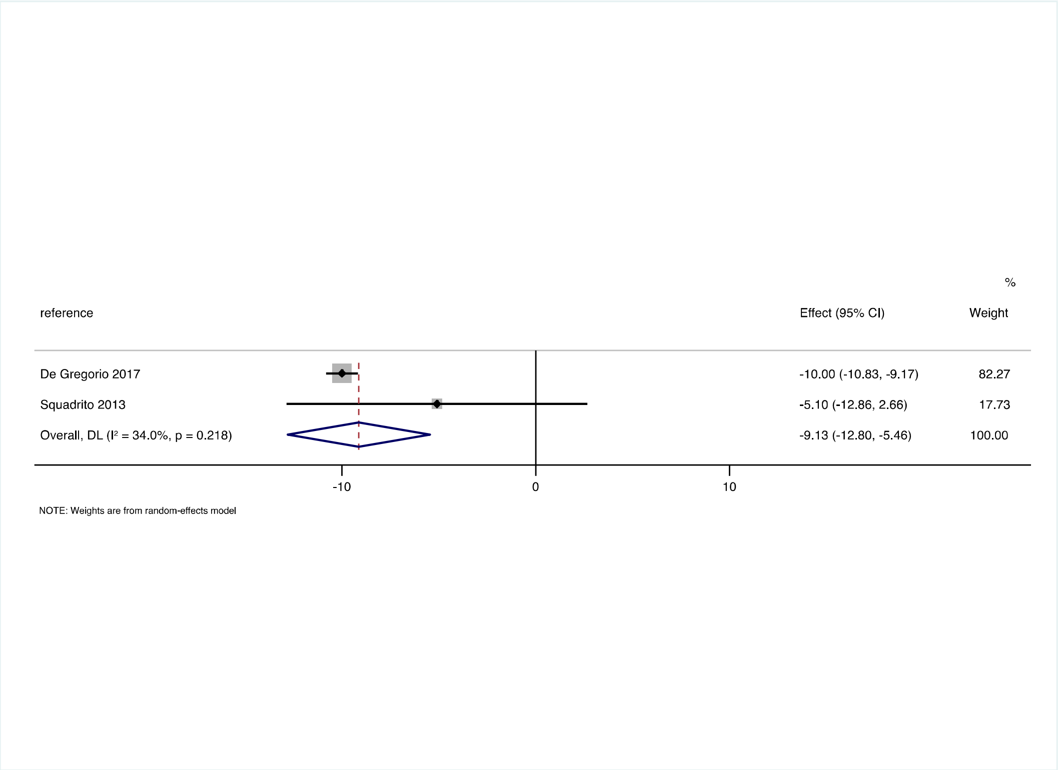

Figure S1-8-2 Forest plot of RCTs investigating the effect of genistein supplementation on DBP.

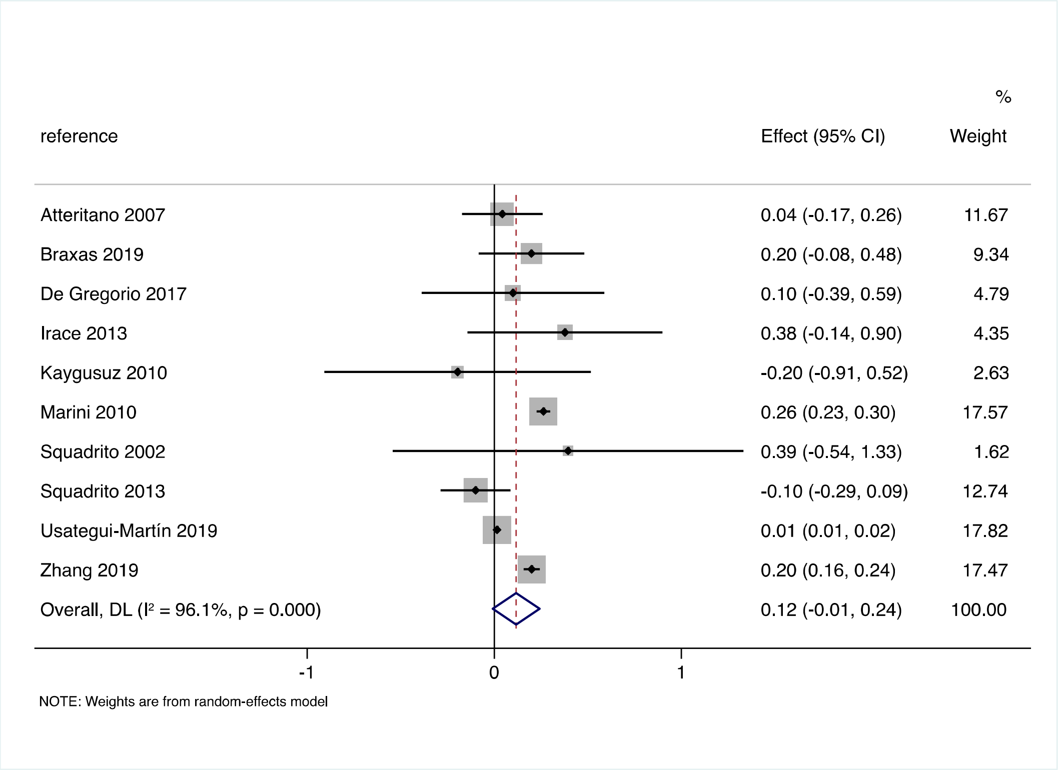

Figure S1-8-3 Forest plot of RCTs investigating the effect of genistein supplementation on HDL-C.

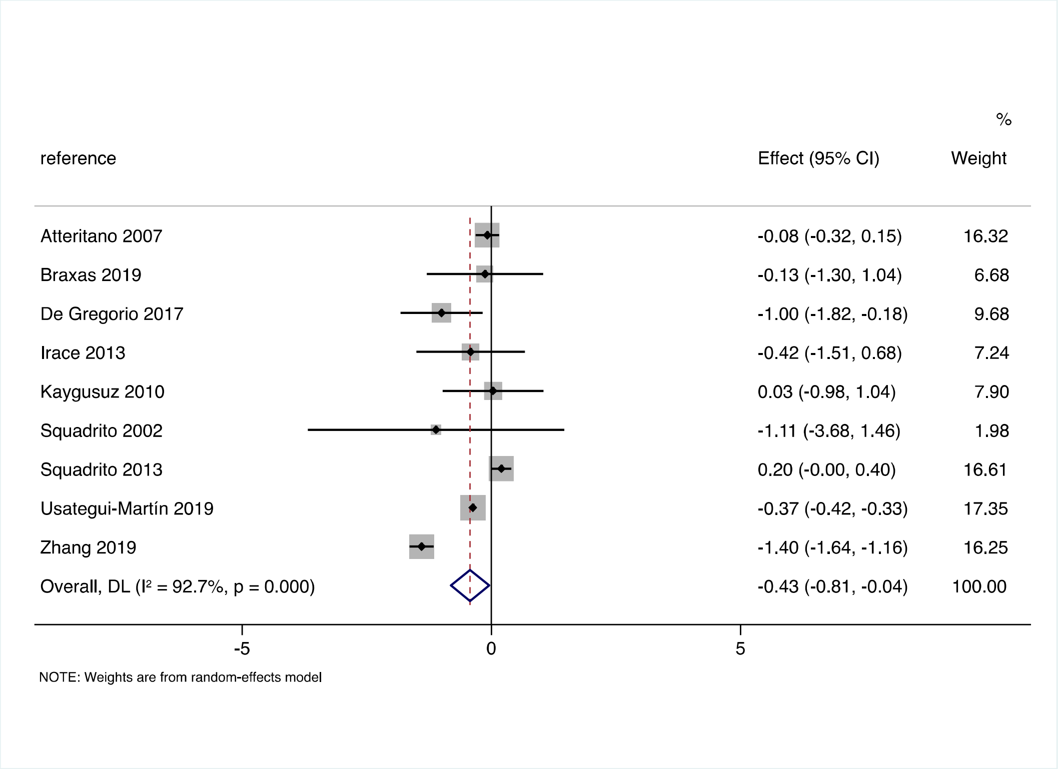

Figure S1-8-4 Forest plot of RCTs investigating the effect of genistein supplementation on LDL-C.

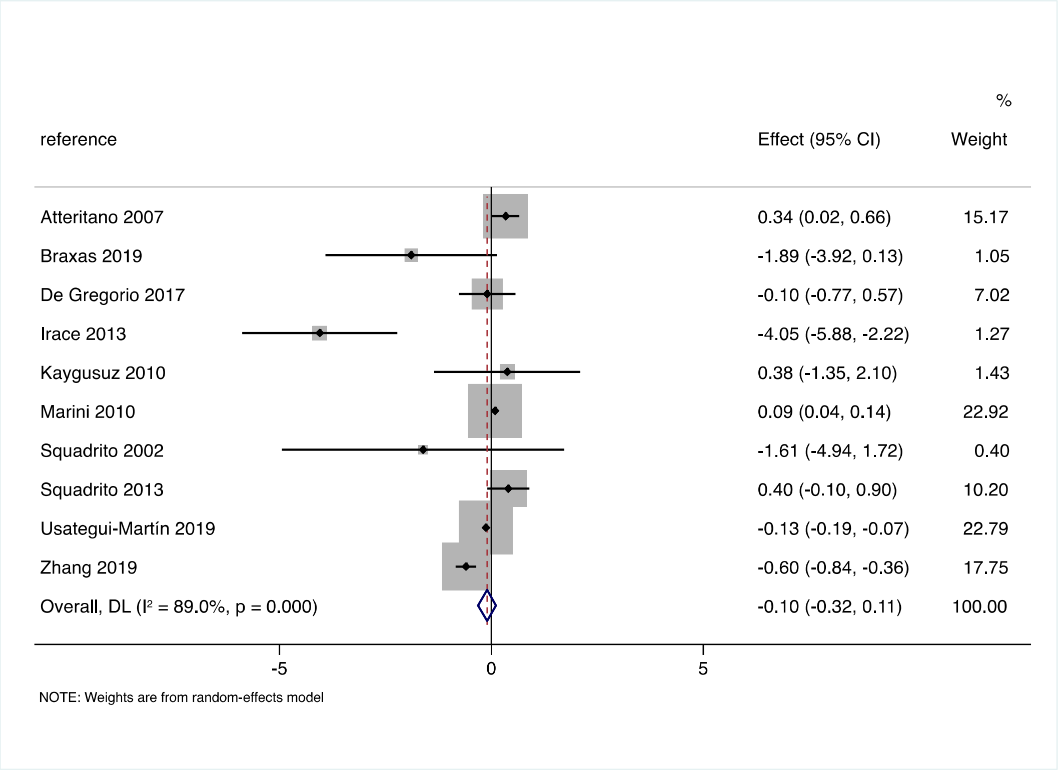

Figure S1-8-5 Forest plot of RCTs investigating the effect of genistein supplementation on TG

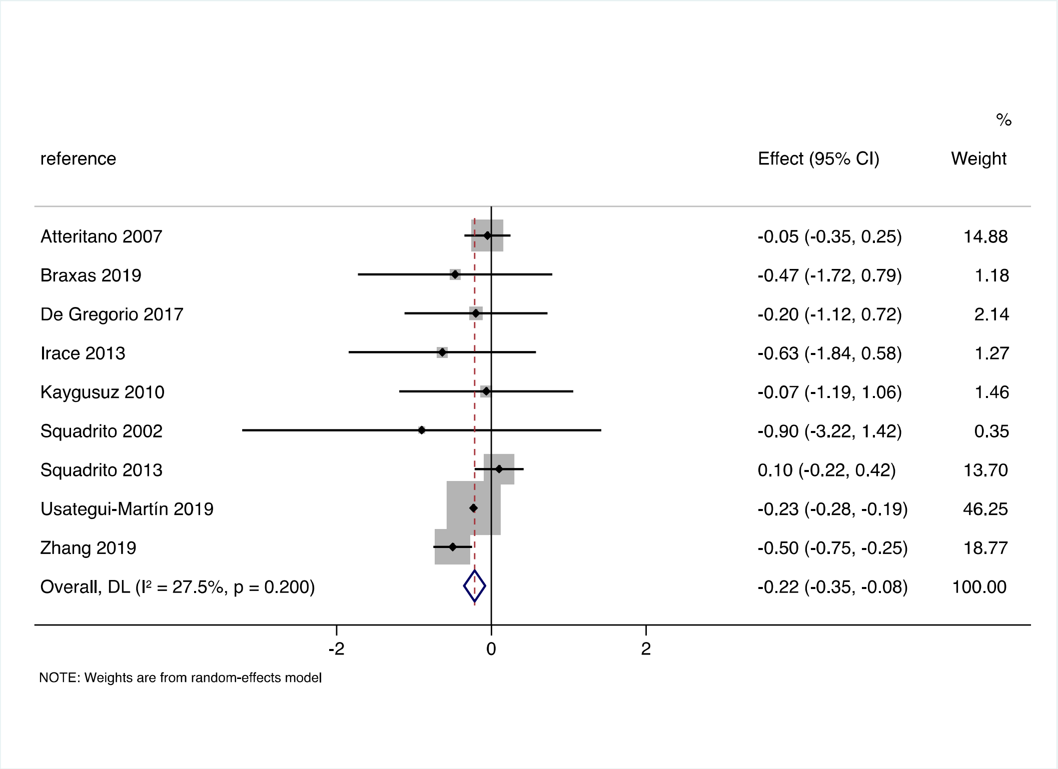

Figure S1-8-6 Forest plot of RCTs investigating the effect of genistein supplementation on TC.

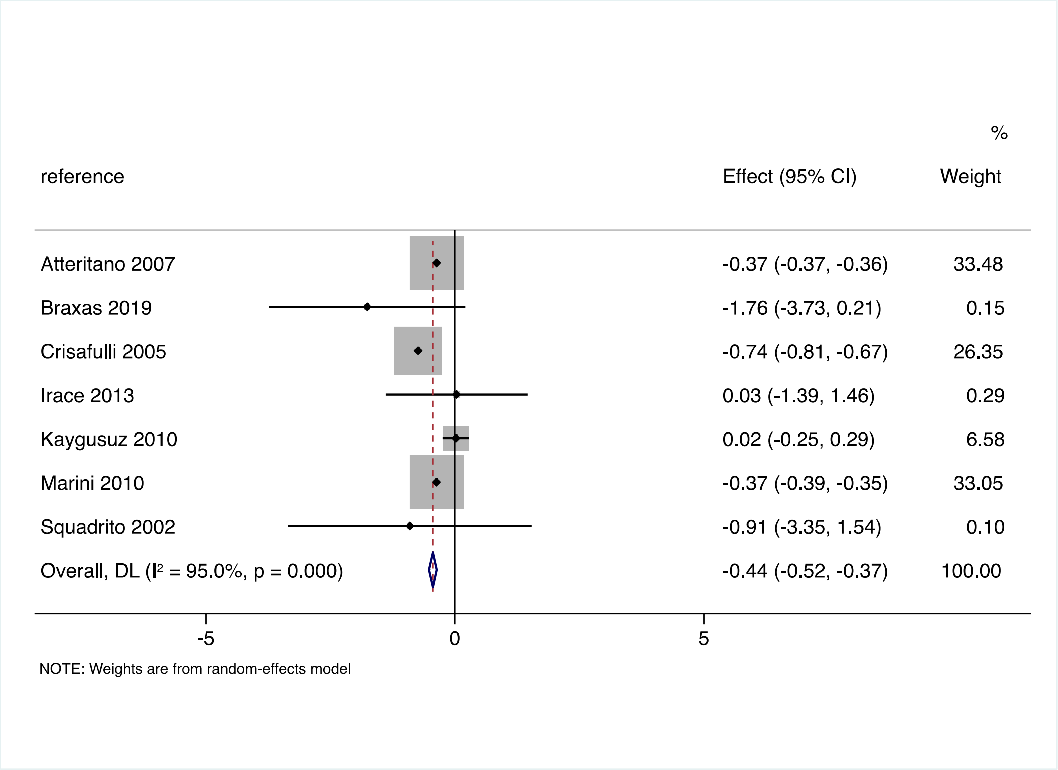

Figure S1-8-7 Forest plot of RCTs investigating the effect of genistein supplementation on FBG.

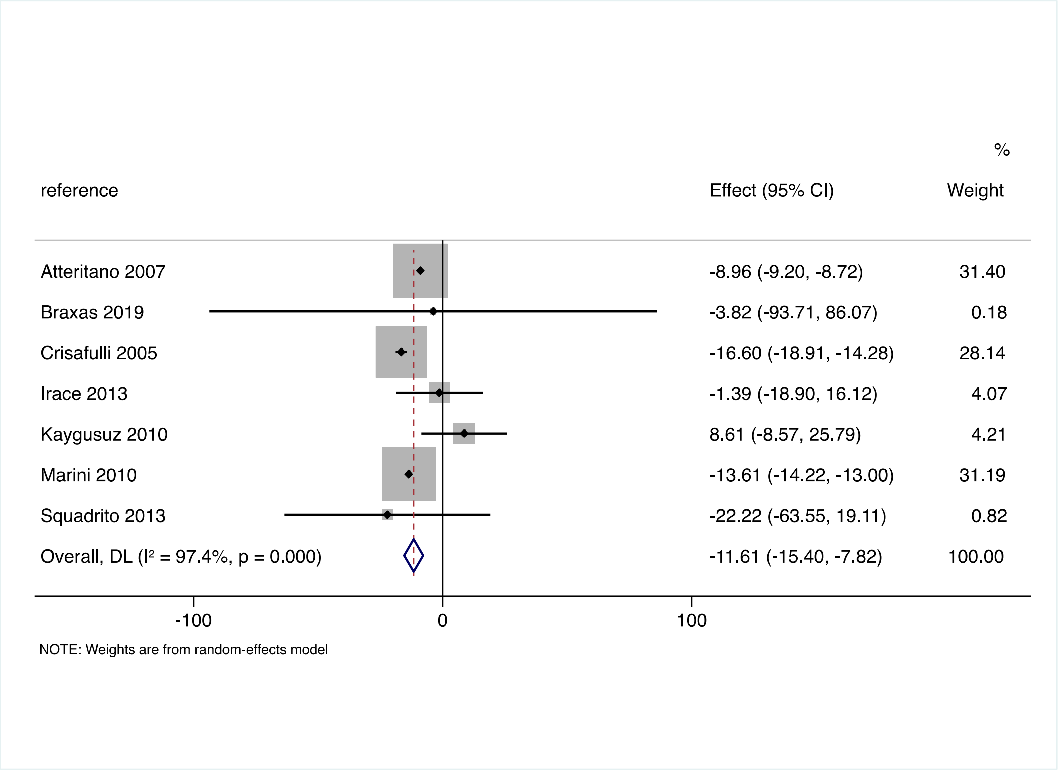

Figure S1-8-8 Forest plot of RCTs investigating the effect of genistein supplementation on FBI.

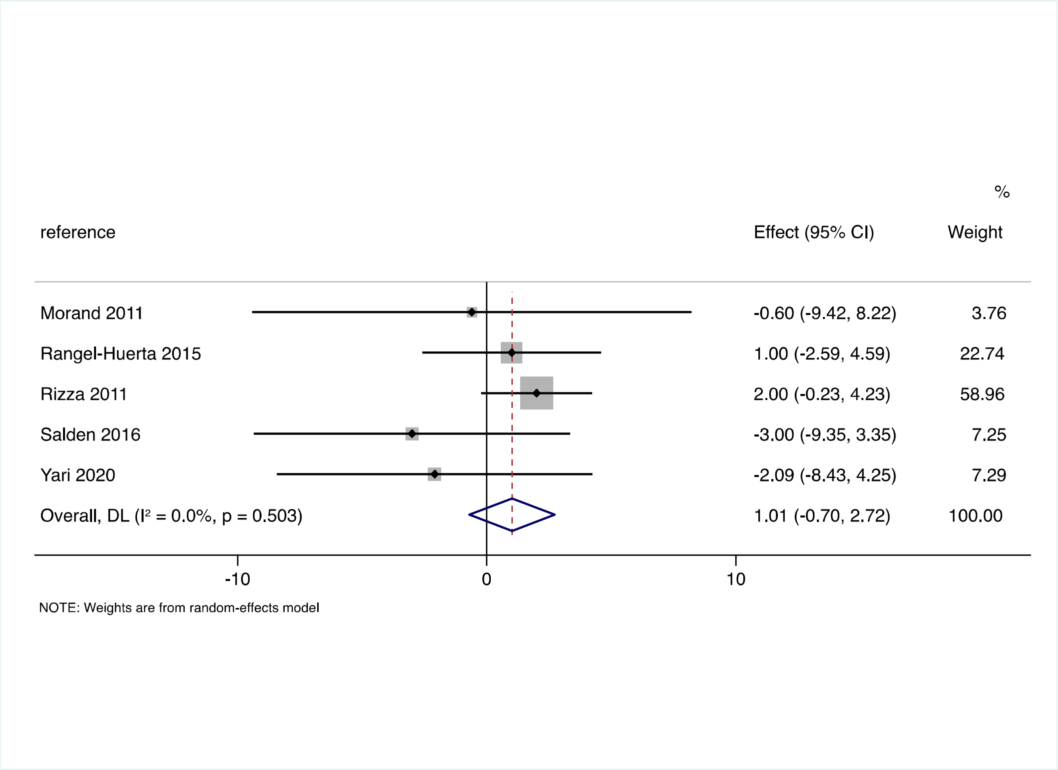

Figure S1-9-1 Forest plot of RCTs investigating the effect of hesperidin supplementation on SBP.

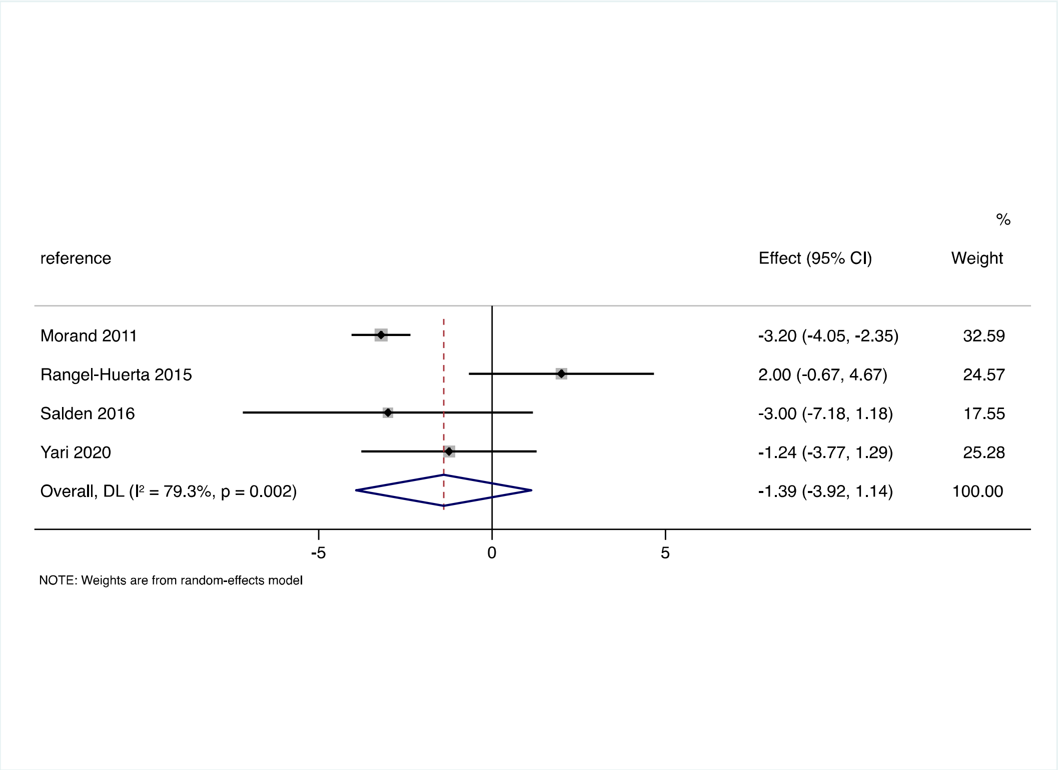

Figure S1-9-2 Forest plot of RCTs investigating the effect of hesperidin supplementation on DBP.

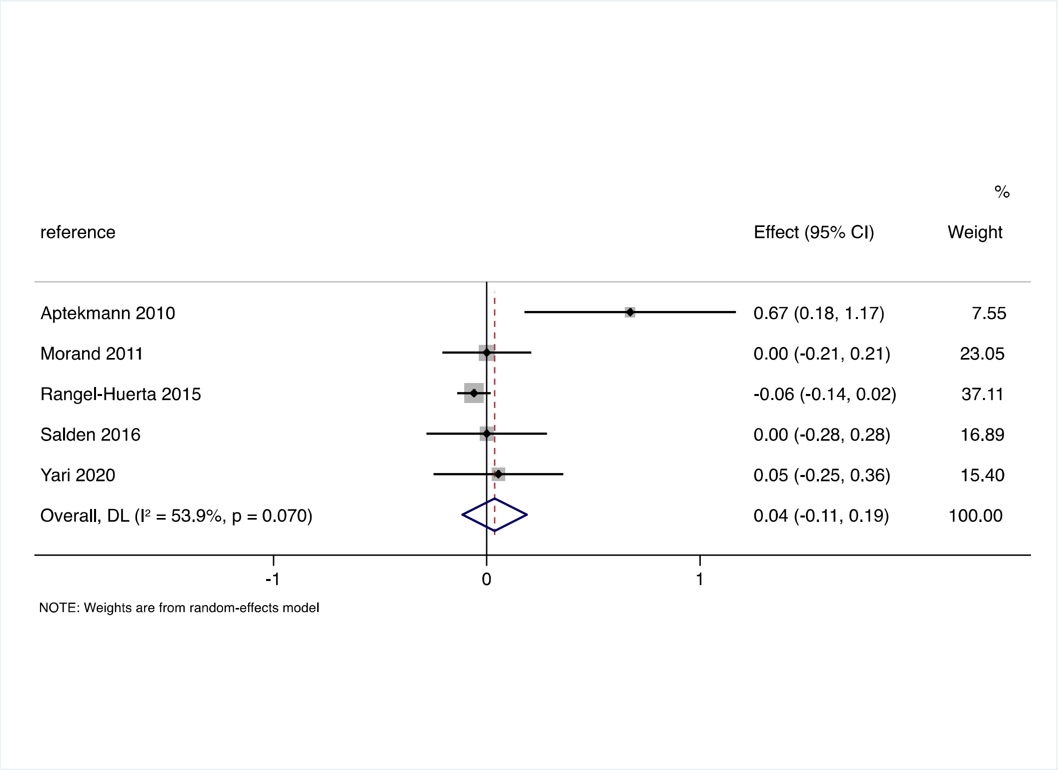

Figure S1-9-3 Forest plot of RCTs investigating the effect of hesperidin supplementation on HDL-C.

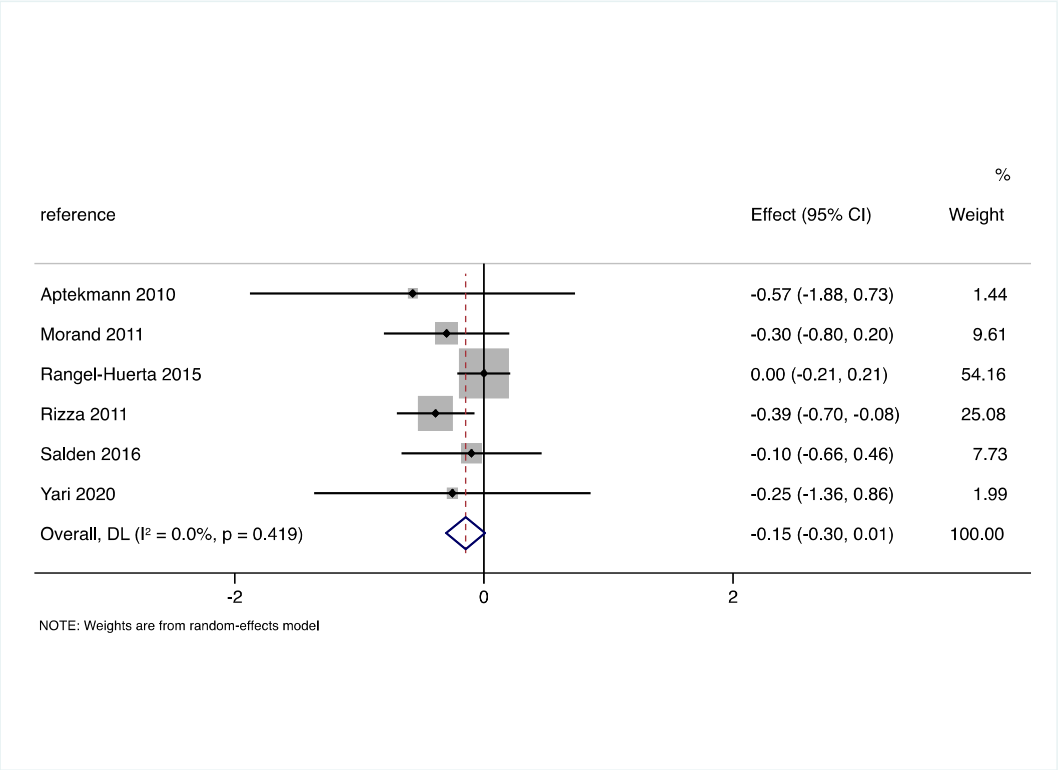

Figure S1-9-4 Forest plot of RCTs investigating the effect of hesperidin supplementation on LDL-C.

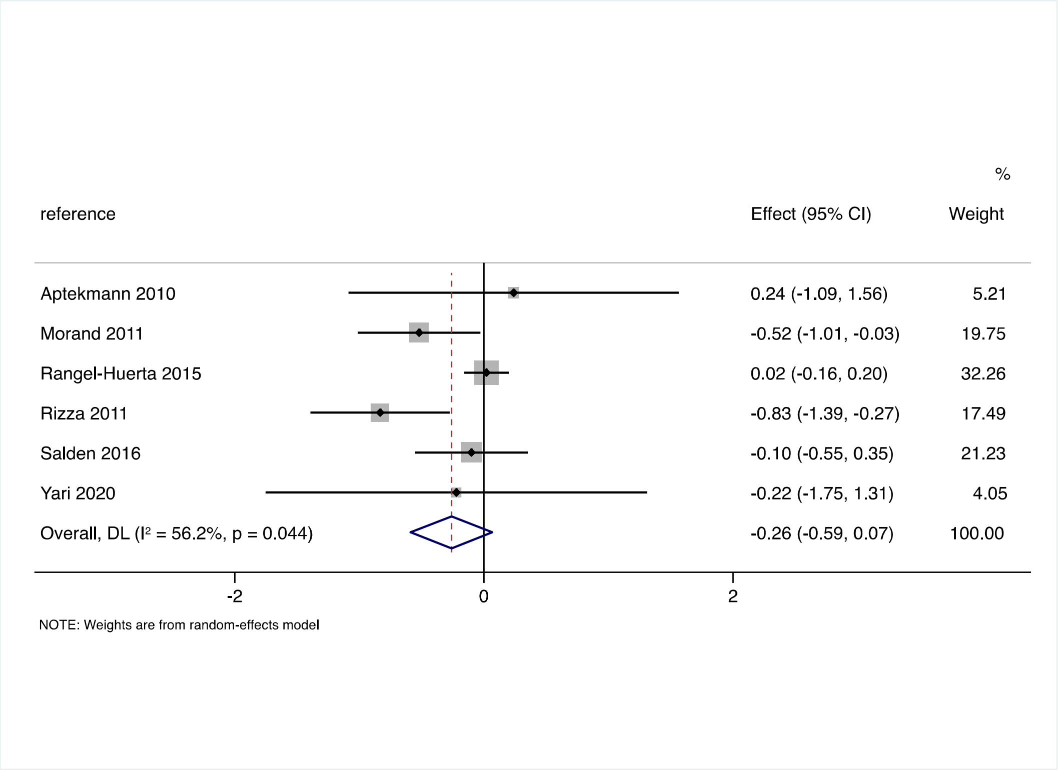

Figure S1-9-5 Forest plot of RCTs investigating the effect of hesperidin supplementation on TG

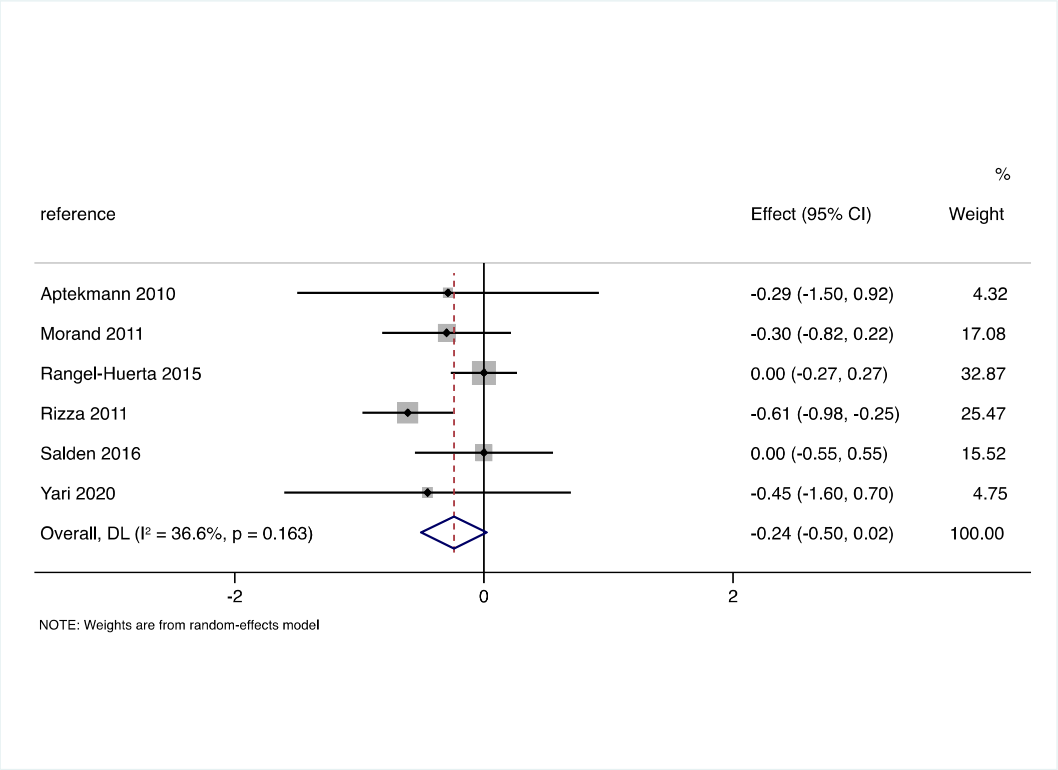

Figure S1-9-6 Forest plot of RCTs investigating the effect of hesperidin supplementation on TC.

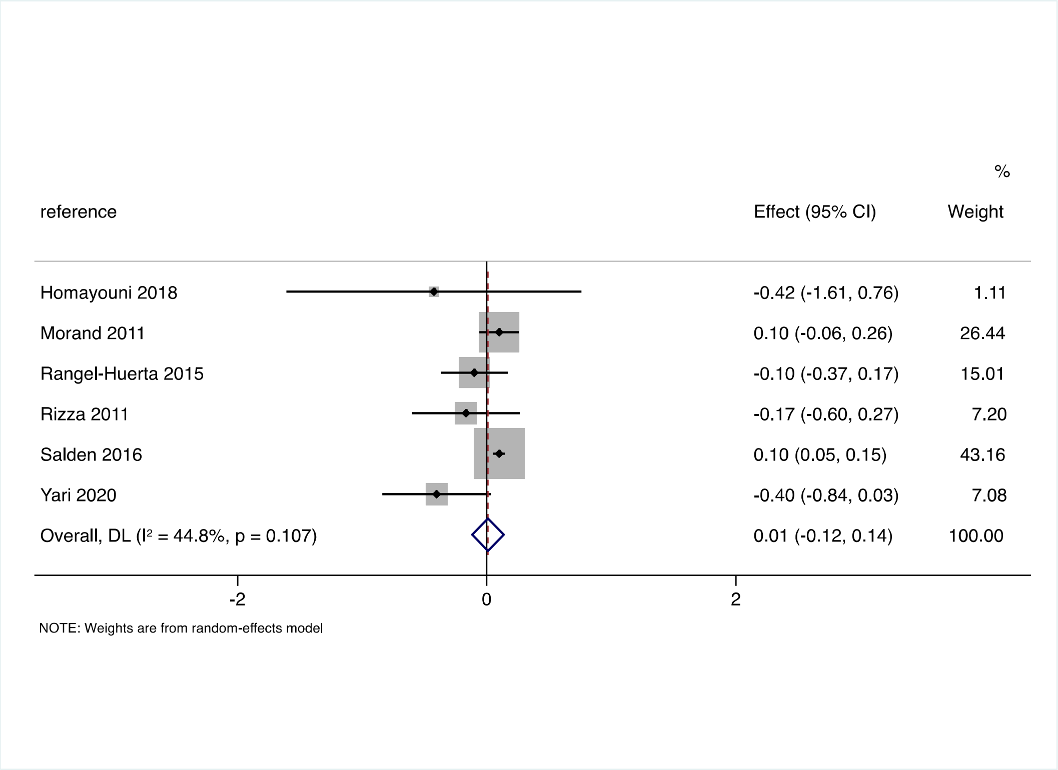

Figure S1-9-7 Forest plot of RCTs investigating the effect of hesperidin supplementation on FBG.

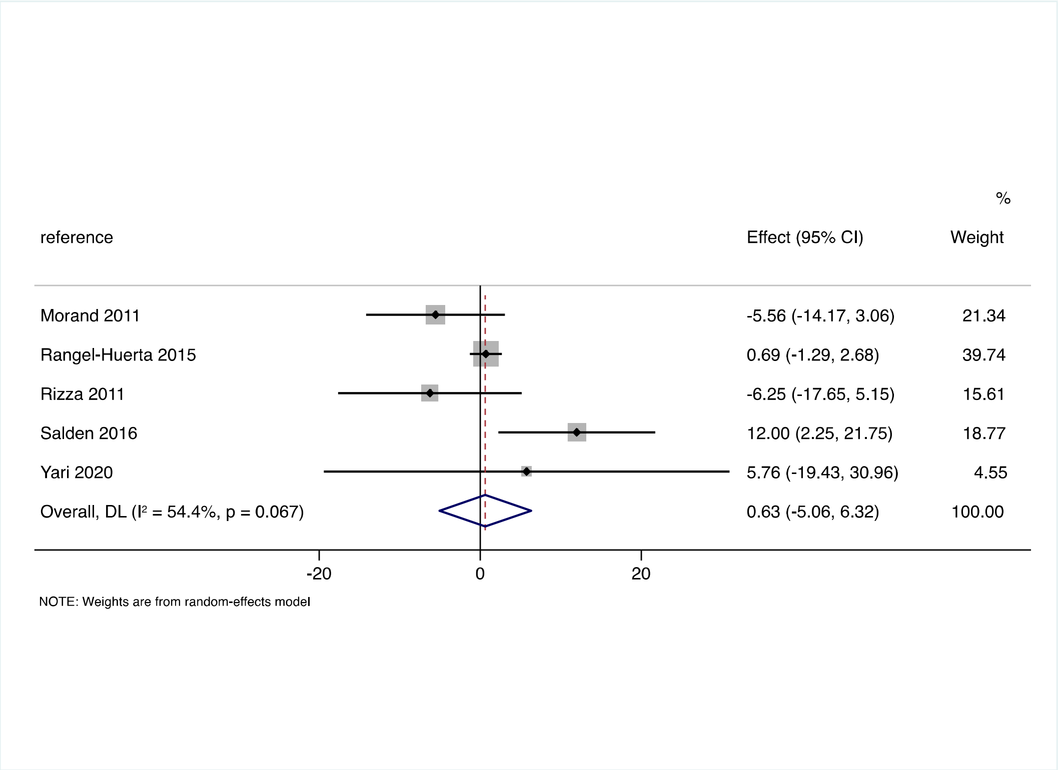

Figure S1-9-8 Forest plot of RCTs investigating the effect of hesperidin supplementation on FBI.

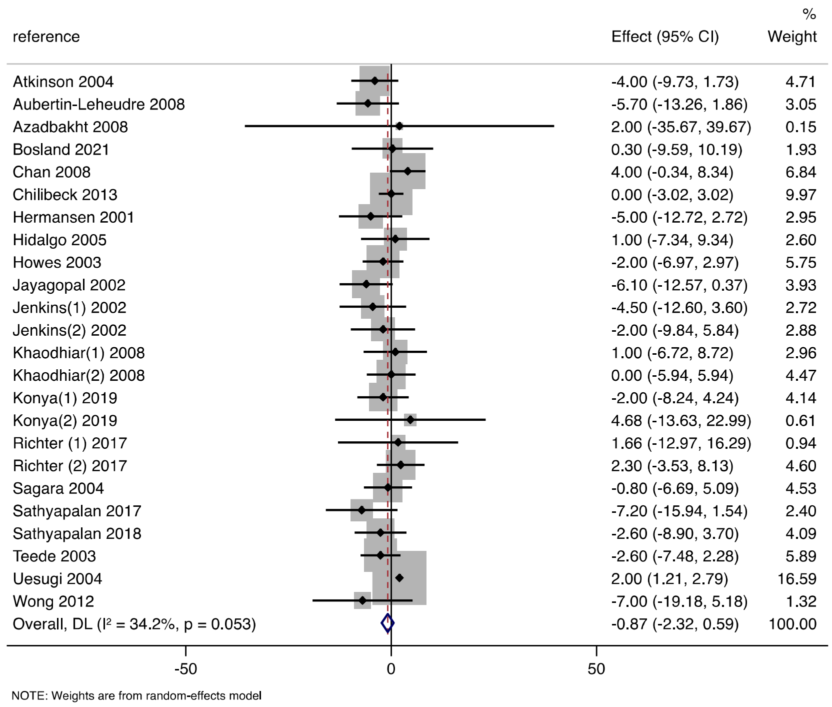

Figure S1-10-1 Forest plot of RCTs investigating the effect of isoflavone supplementation on SBP.

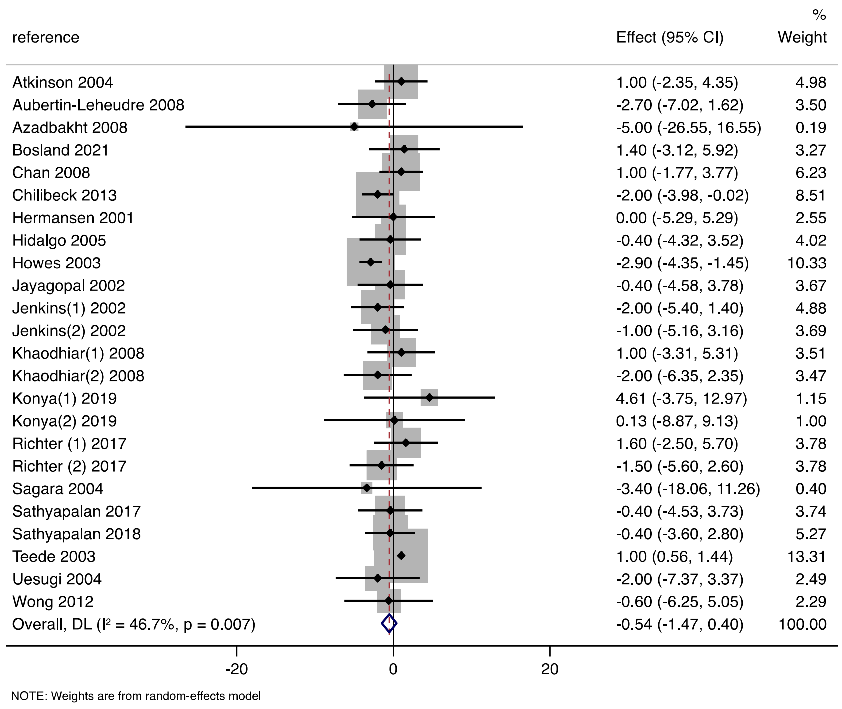

Figure S1-10-2 Forest plot of RCTs investigating the effect of isoflavone supplementation on DBP.

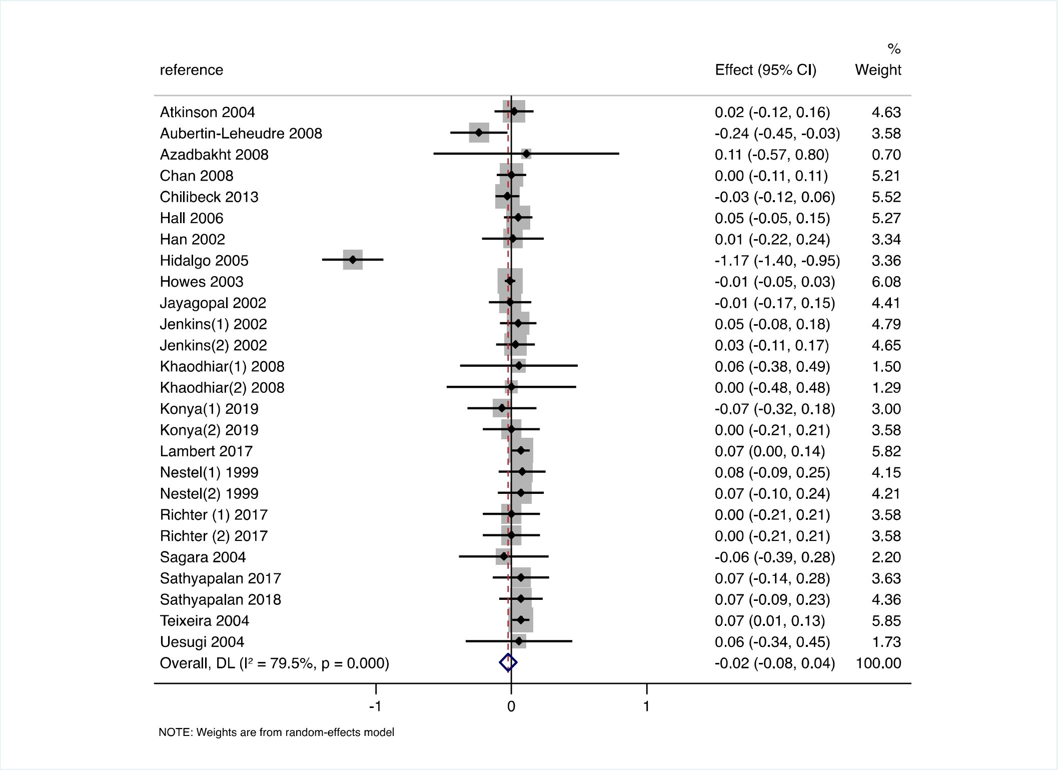

Figure S1-10-3 Forest plot of RCTs investigating the effect of isoflavone supplementation on HDL-C.

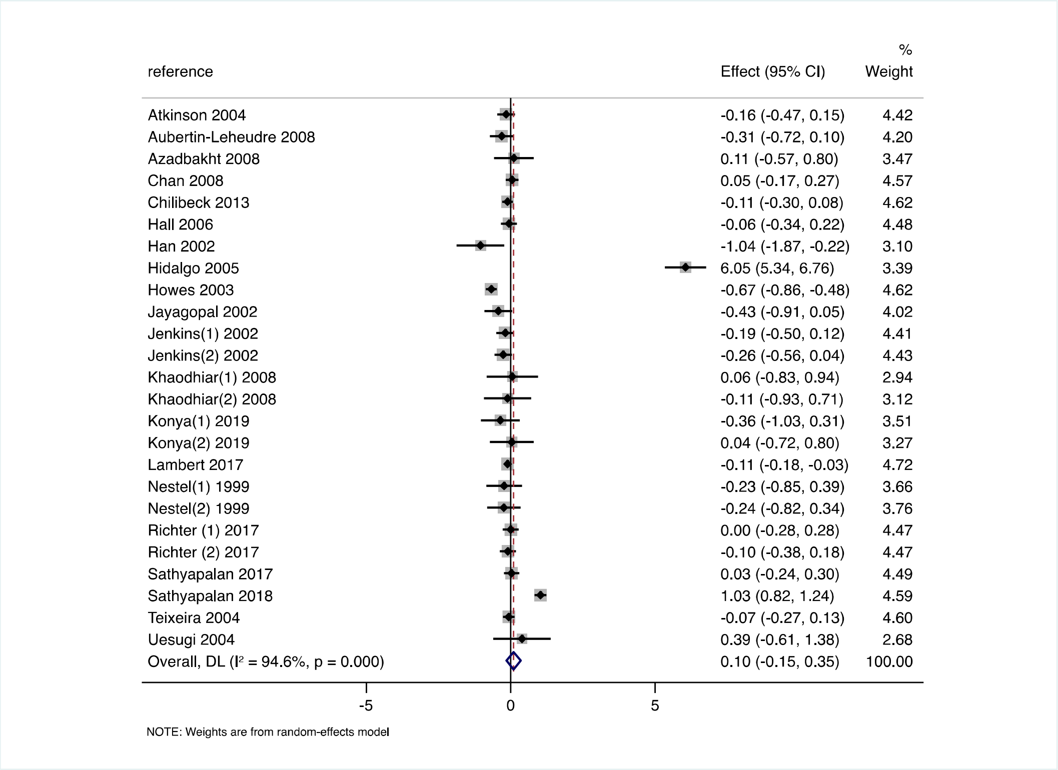

Figure S1-10-4 Forest plot of RCTs investigating the effect of isoflavone supplementation on

LDL-C.

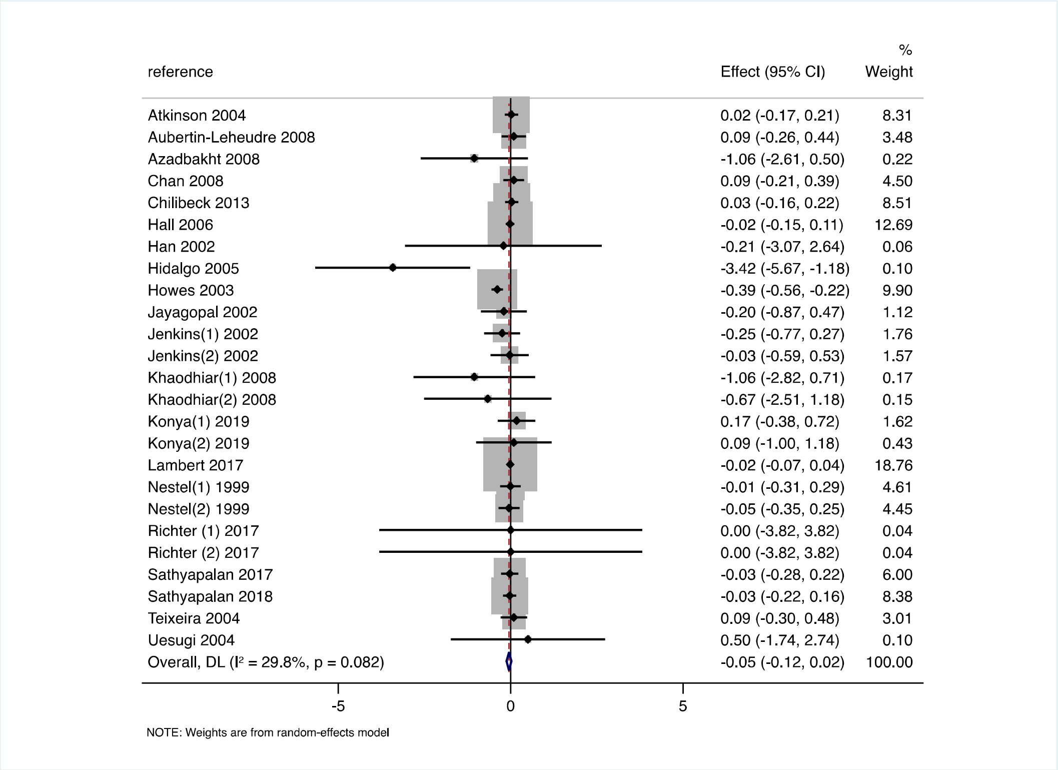

Figure S1-10-5 Forest plot of RCTs investigating the effect of isoflavone supplementation on TG

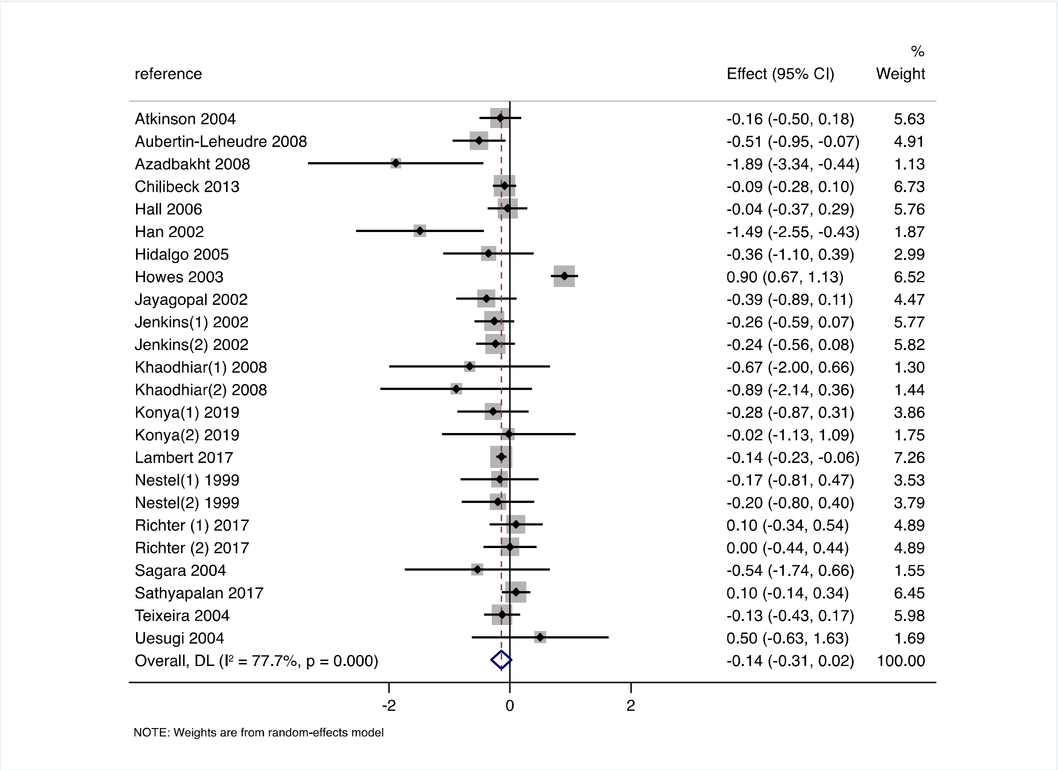

Figure S1-10-6 Forest plot of RCTs investigating the effect of isoflavone supplementation on TC.

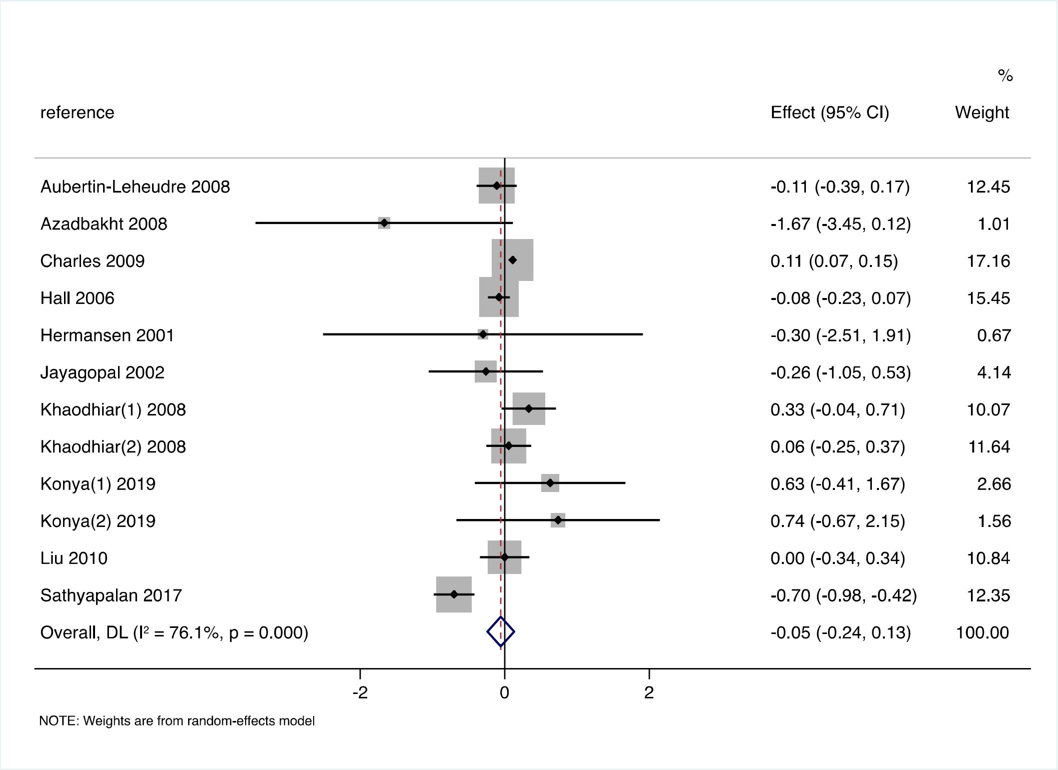

Figure S1-10-7 Forest plot of RCTs investigating the effect of isoflavone supplementation on FBG.

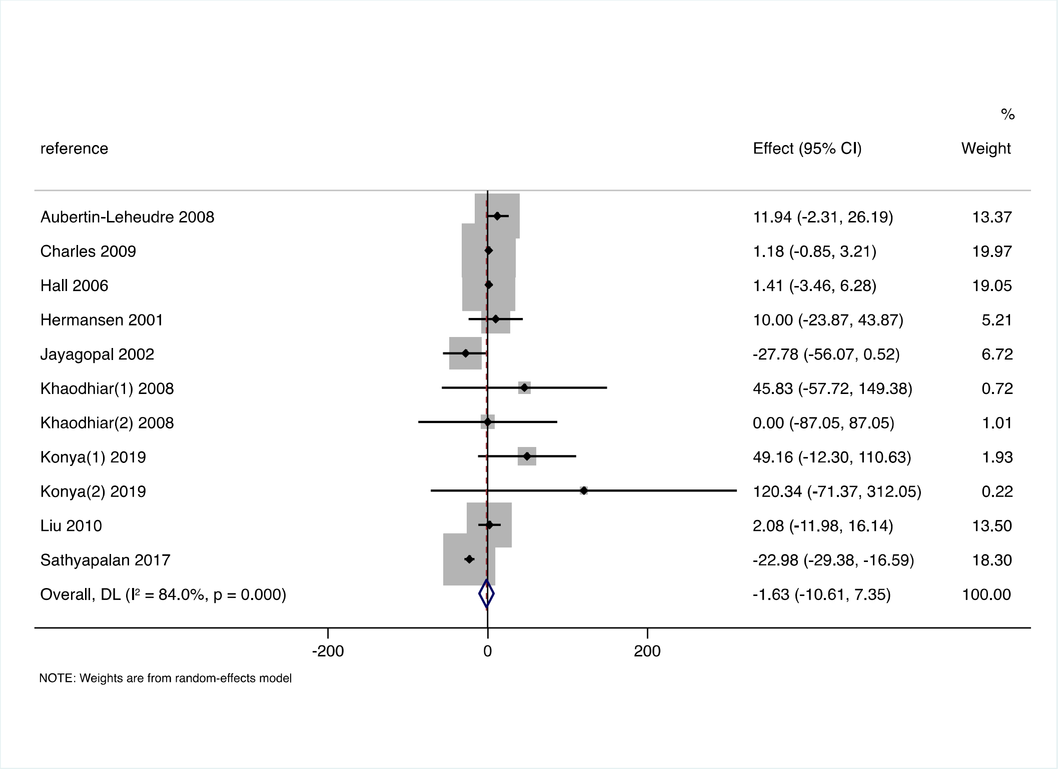

Figure S1-10-8 Forest plot of RCTs investigating the effect of isoflavone supplementation on FBI.

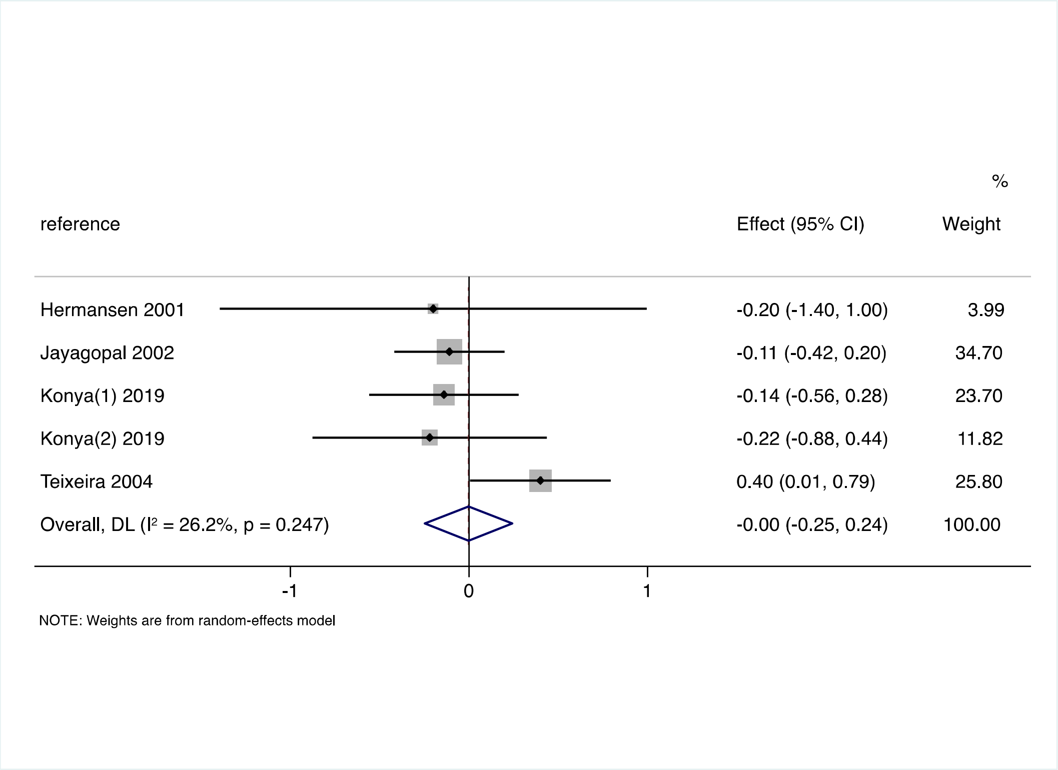

Figure S1-10-9 Forest plot of RCTs investigating the effect of isoflavone supplementation on A1C.

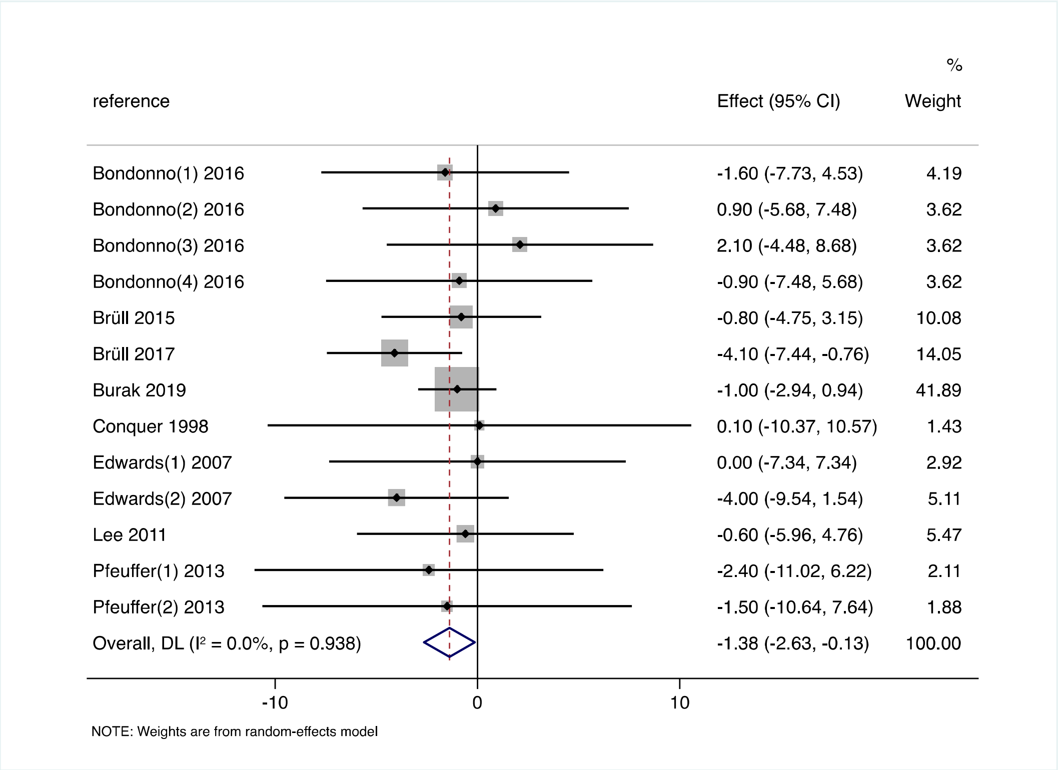

Figure S1-11-1 Forest plot of RCTs investigating the effect of quercetin supplementation on SBP.

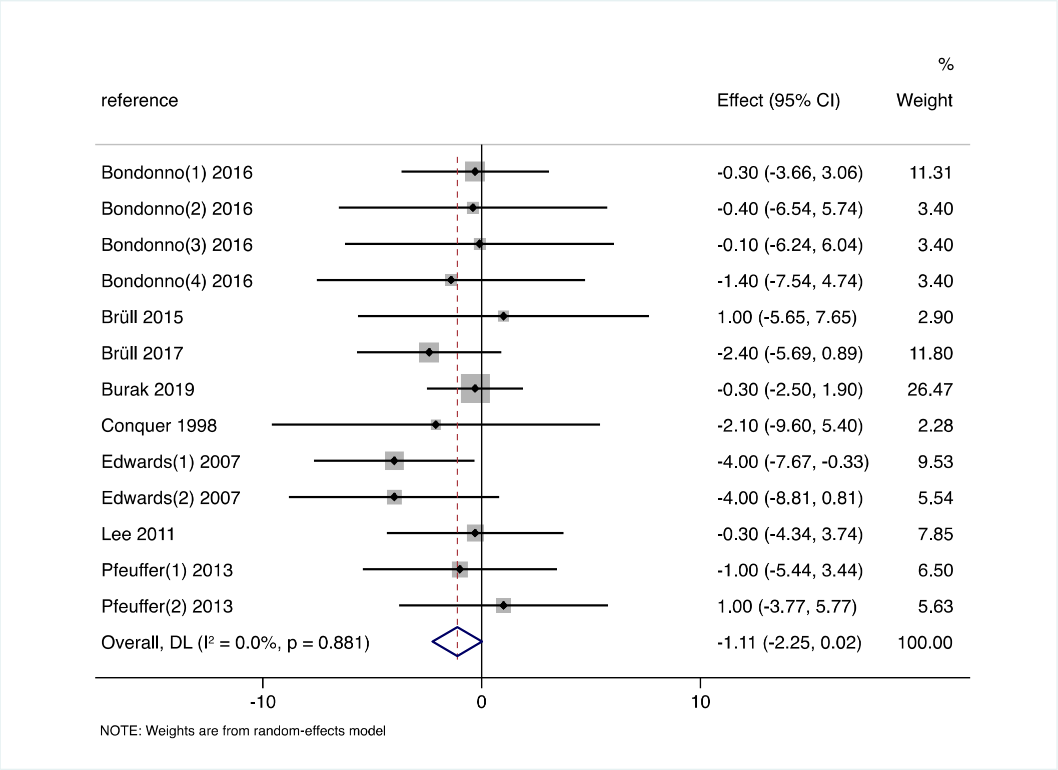

Figure S1-11-2 Forest plot of RCTs investigating the effect of quercetin supplementation on DBP.

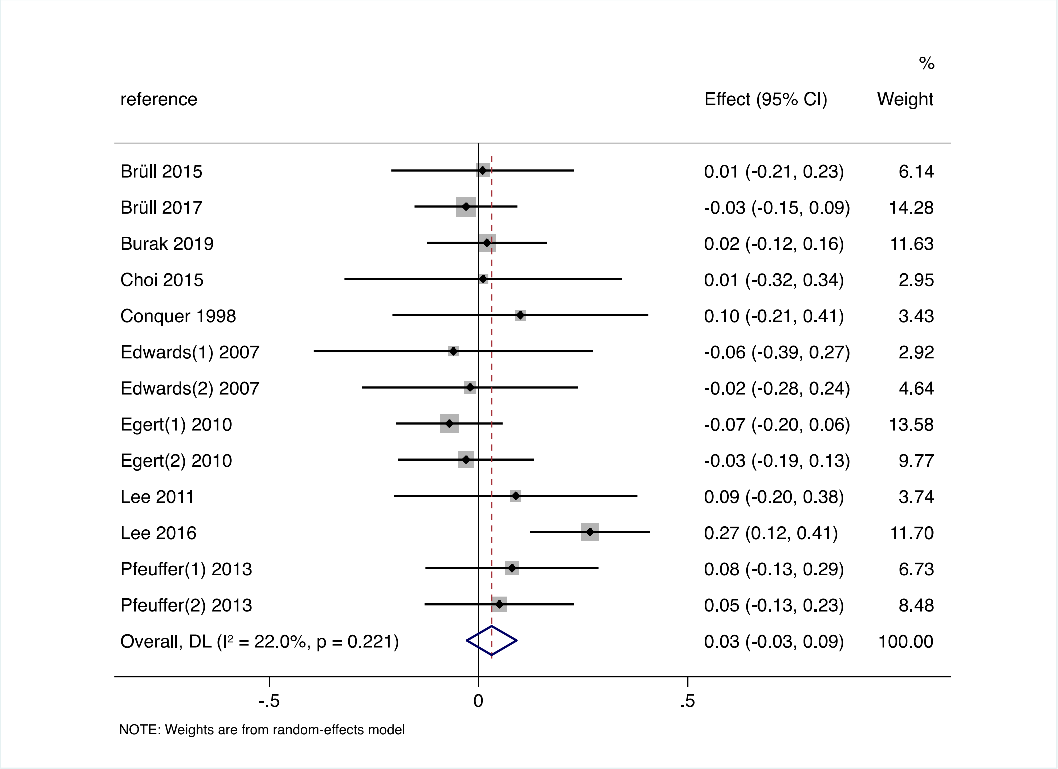

Figure S1-11-3 Forest plot of RCTs investigating the effect of quercetin supplementation on HDL-C.

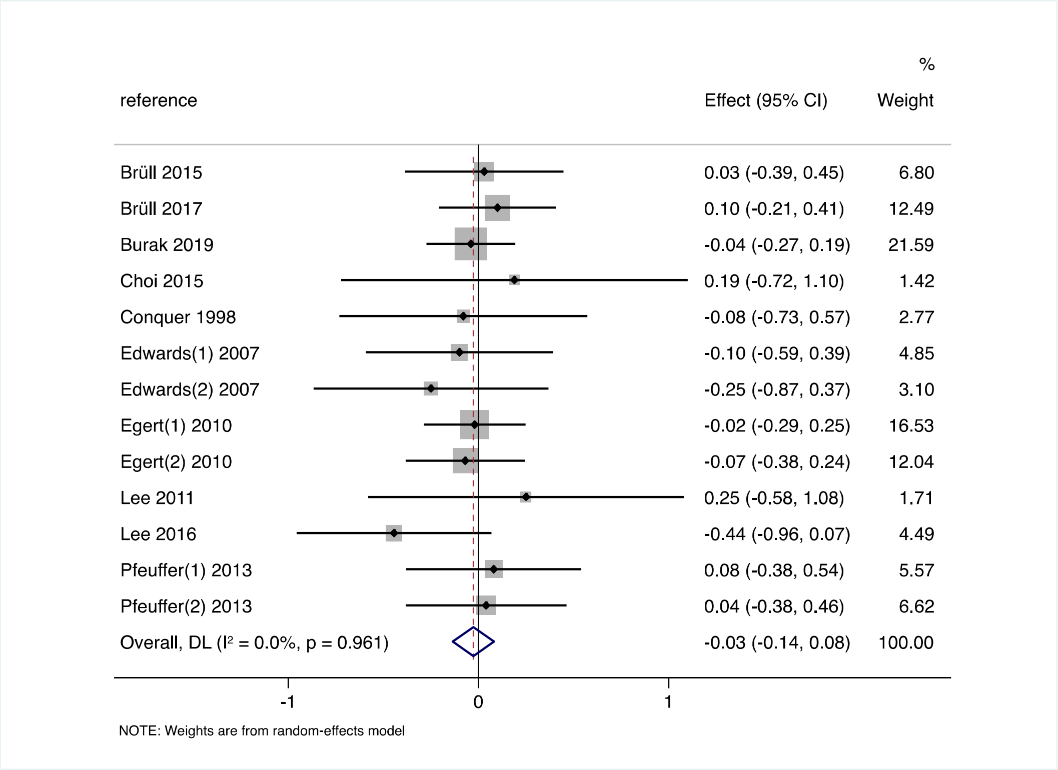

Figure S1-11-4 Forest plot of RCTs investigating the effect of quercetin supplementation on LDL-C.

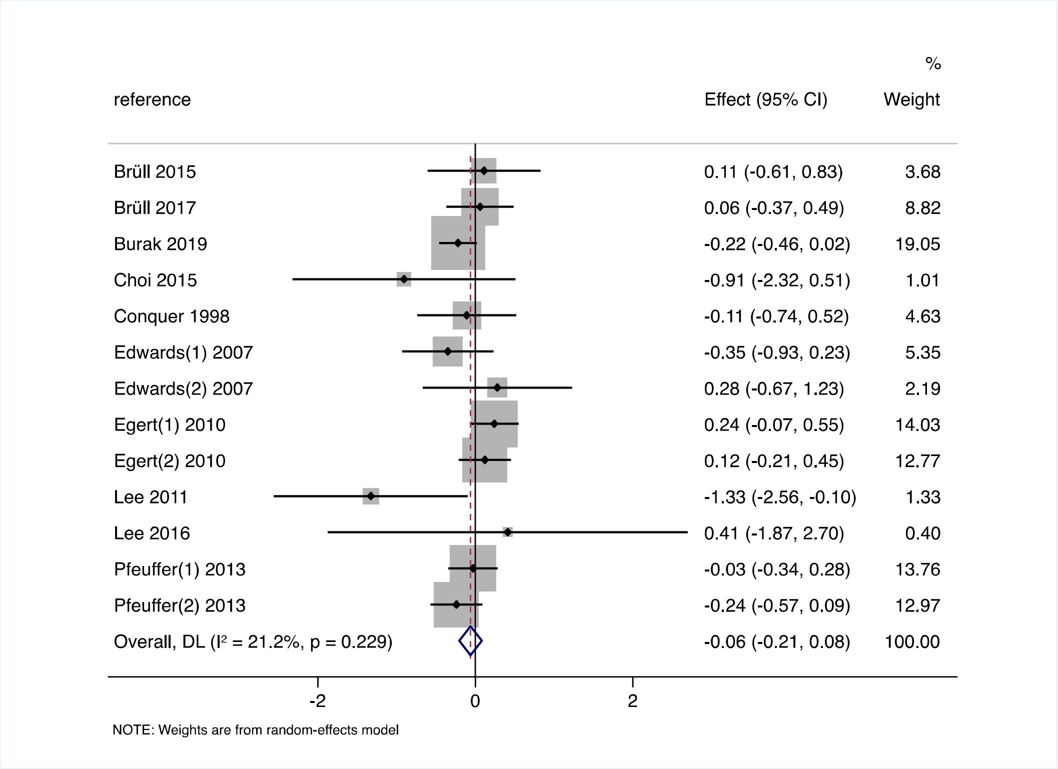

Figure S1-11-5 Forest plot of RCTs investigating the effect of quercetin supplementation on TG

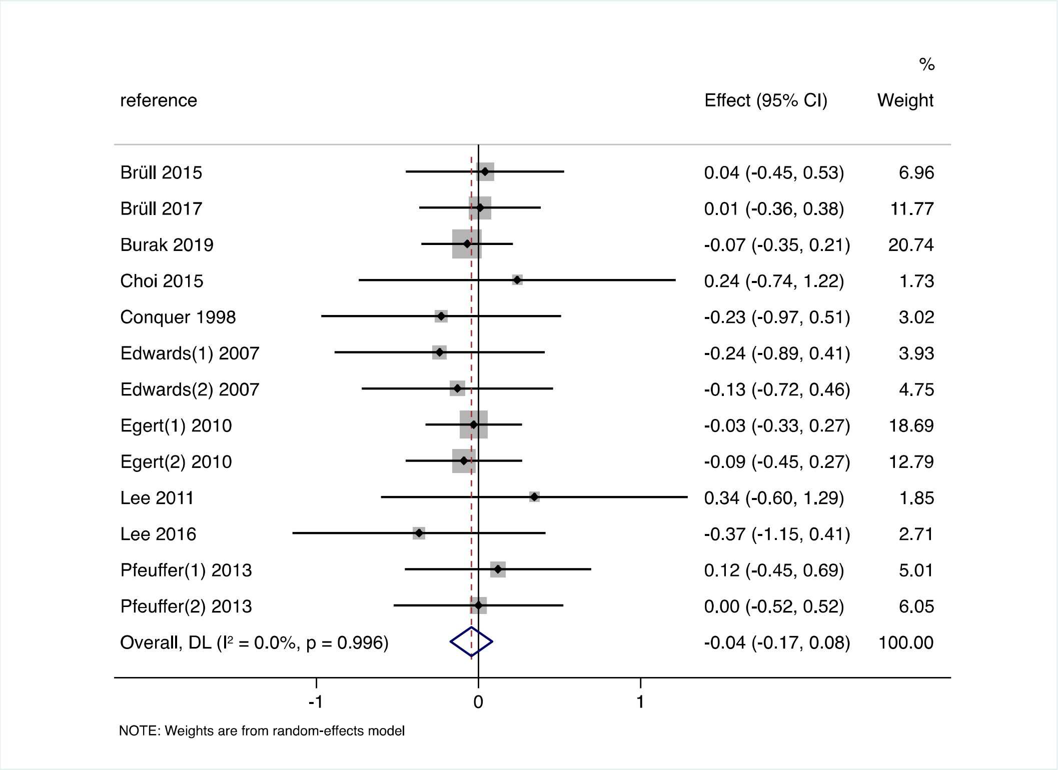

Figure S1-11-6 Forest plot of RCTs investigating the effect of quercetin supplementation on TC.

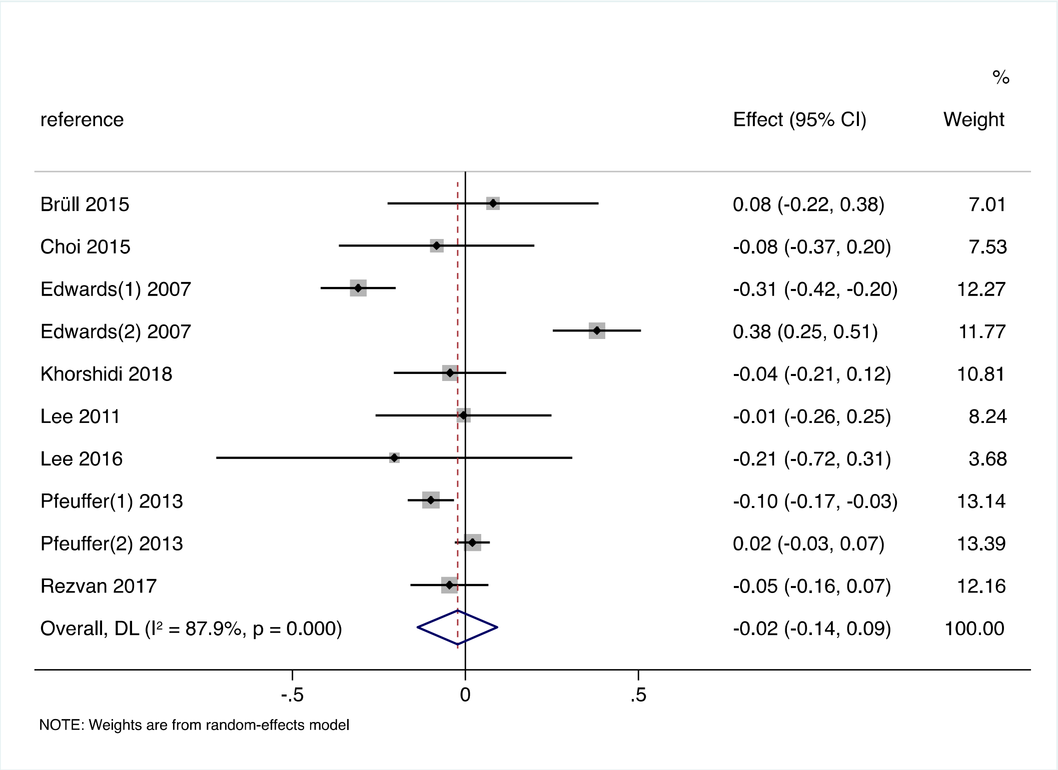

Figure S1-11-7 Forest plot of RCTs investigating the effect of quercetin supplementation on FBG.

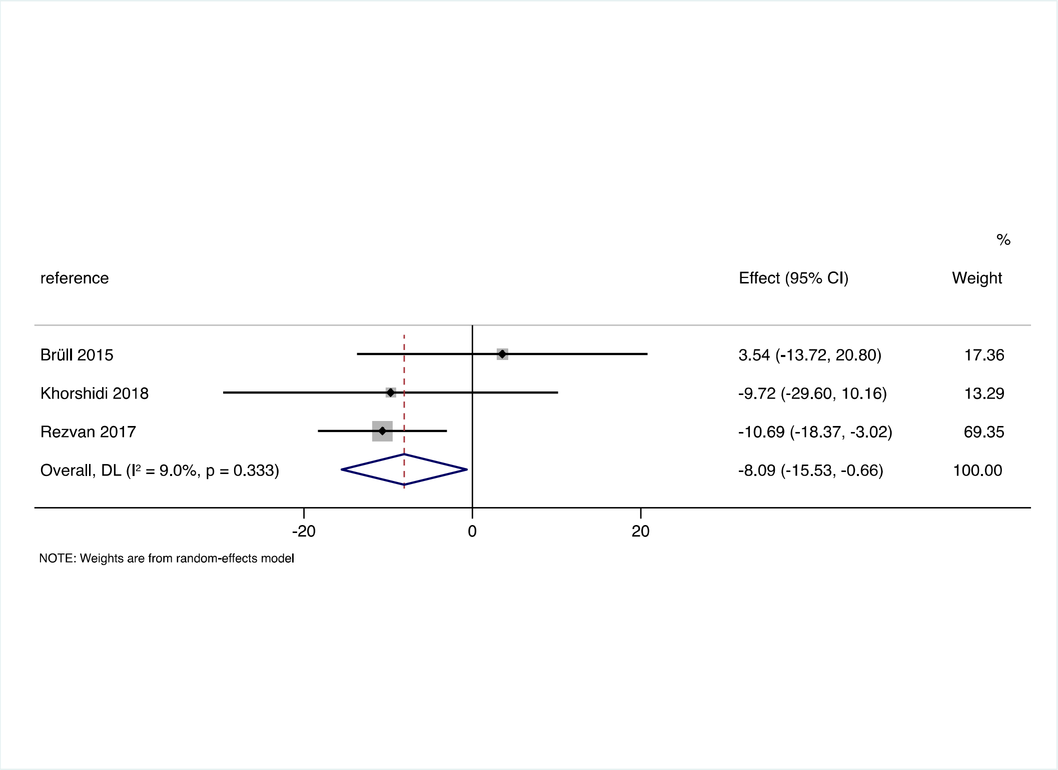

Figure S1-11-8 Forest plot of RCTs investigating the effect of quercetin supplementation on FBI.

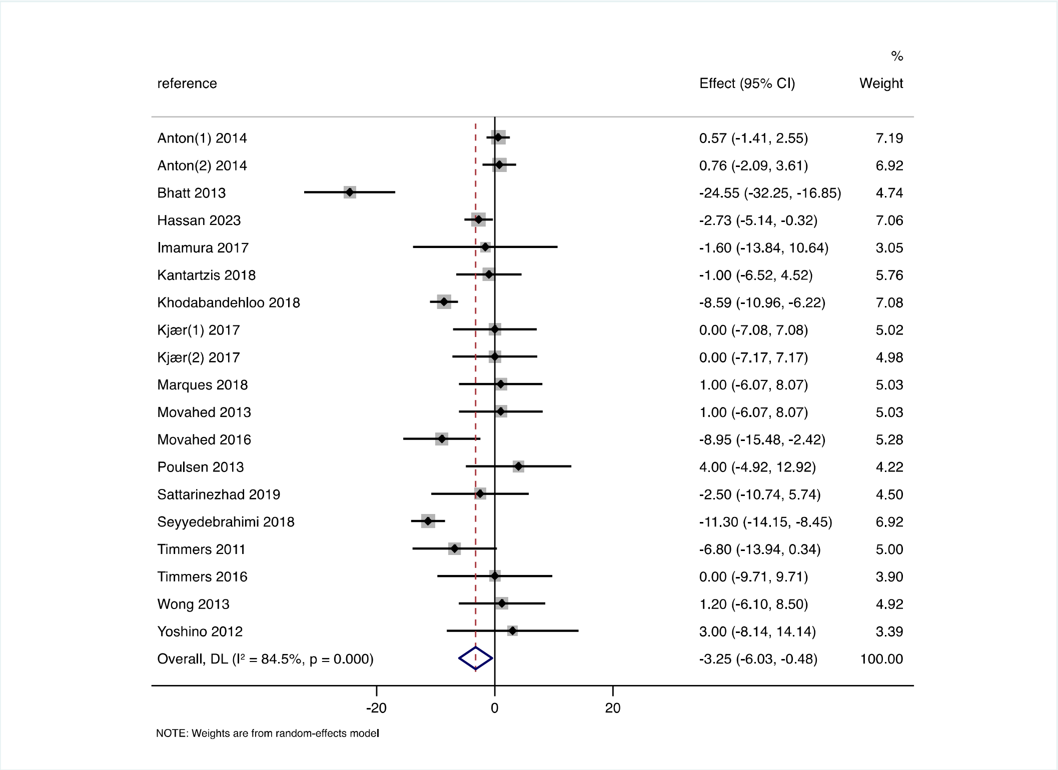

Figure S1-12-1 Forest plot of RCTs investigating the effect of resveratrol supplementation on SBP.

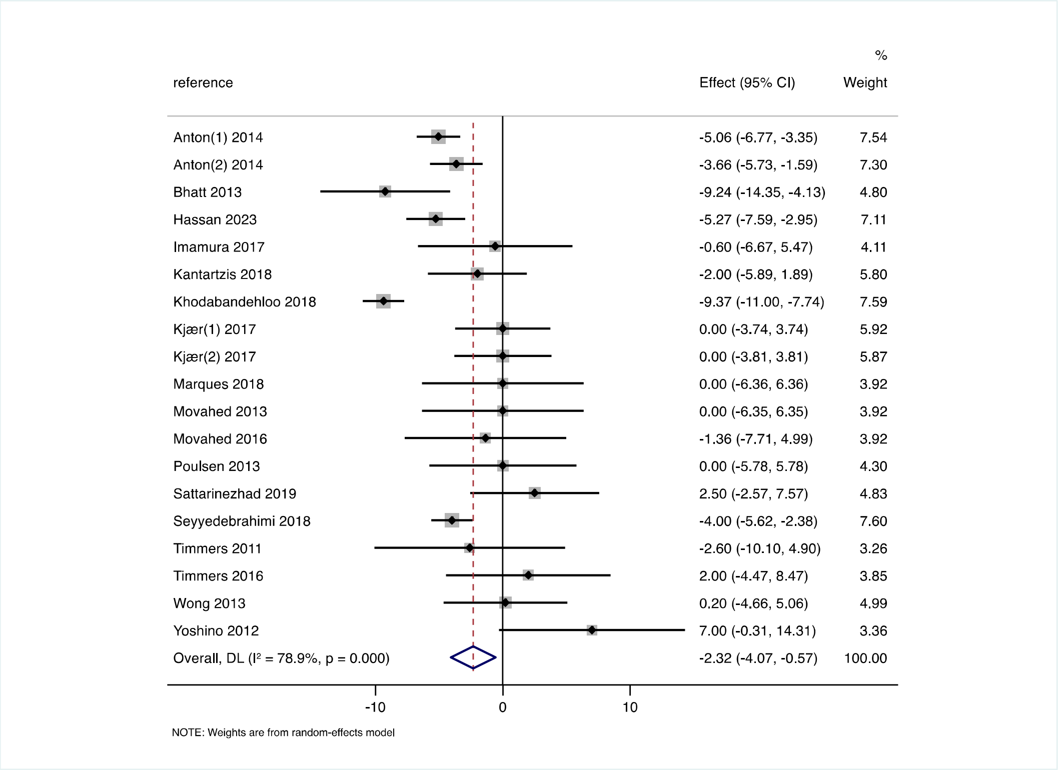

Figure S1-12-2 Forest plot of RCTs investigating the effect of resveratrol supplementation on DBP.

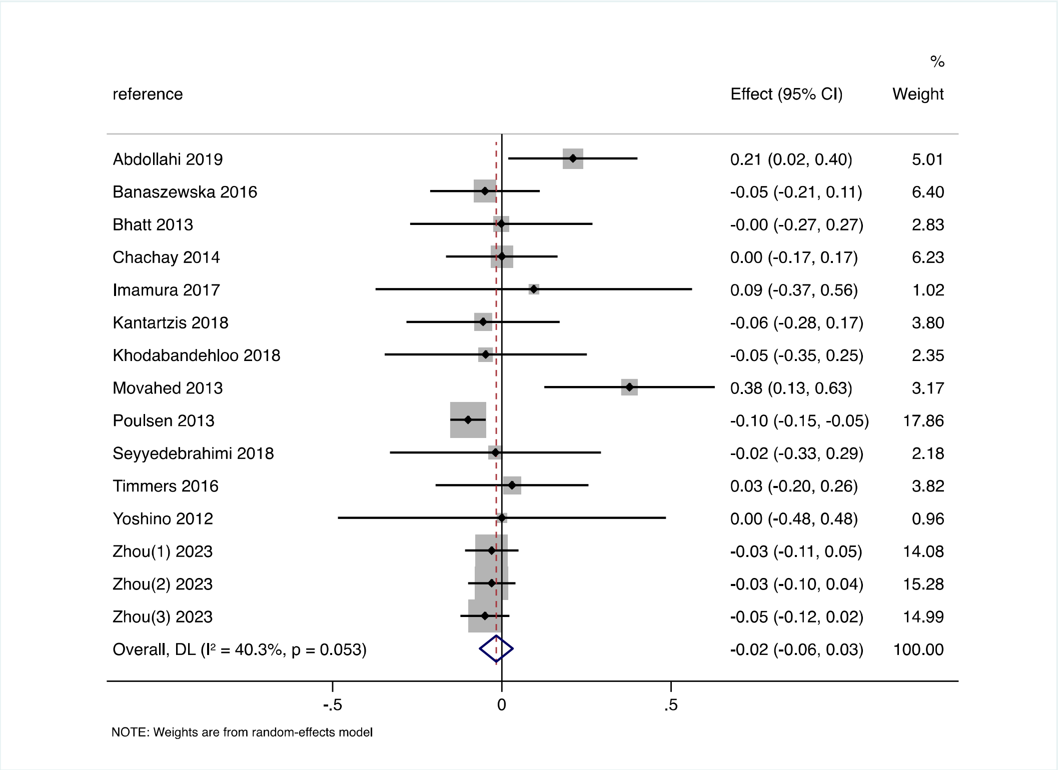

Figure S1-12-3 Forest plot of RCTs investigating the effect of resveratrol supplementation on HDL-C

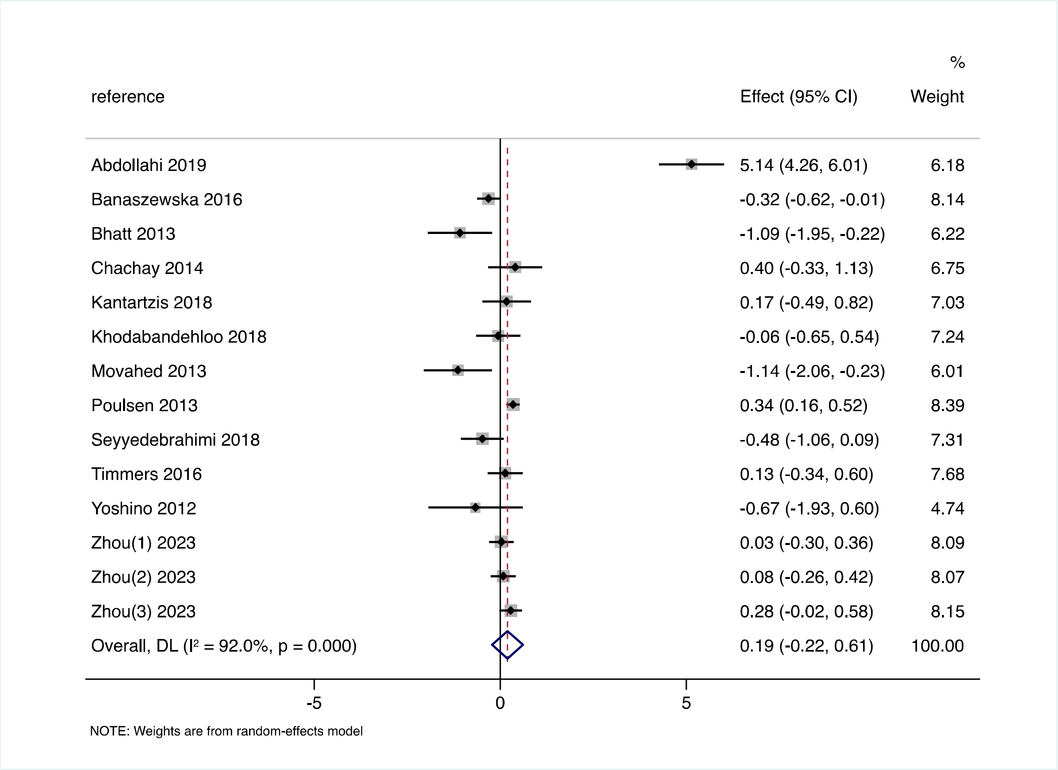

Figure S1-12-4 Forest plot of RCTs investigating the effect of resveratrol supplementation on LDL-C.

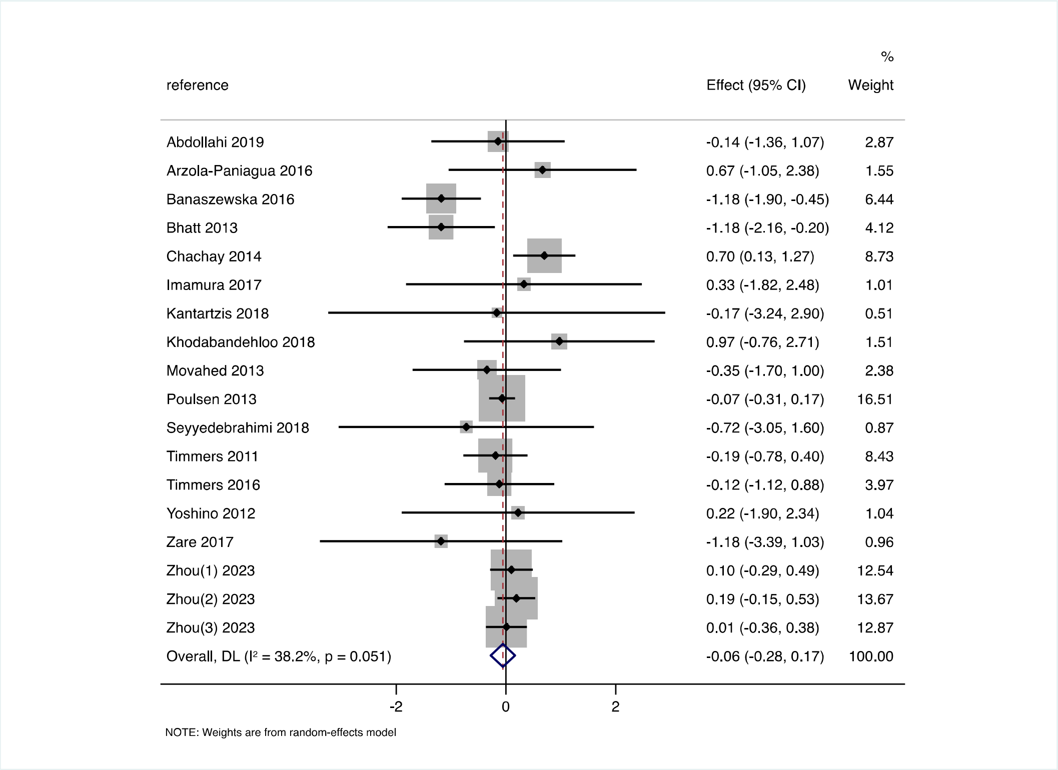

Figure S1-12-5 Forest plot of RCTs investigating the effect of resveratrol supplementation on TG

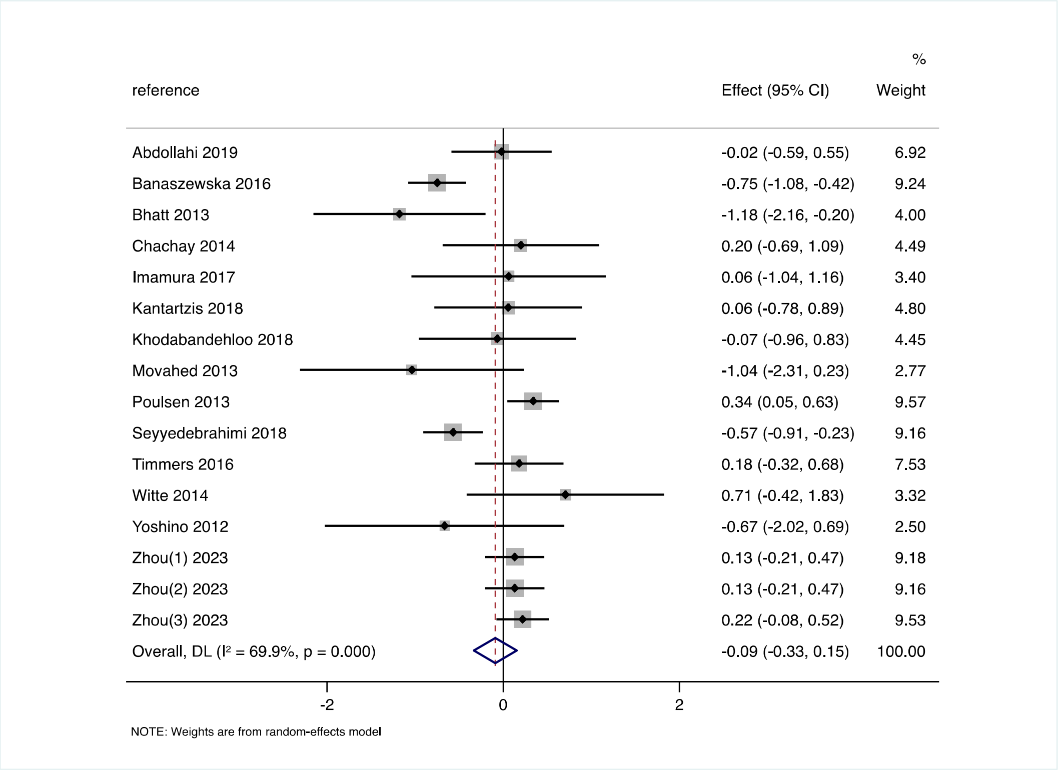

Figure S1-12-6 Forest plot of RCTs investigating the effect of resveratrol supplementation on TC.

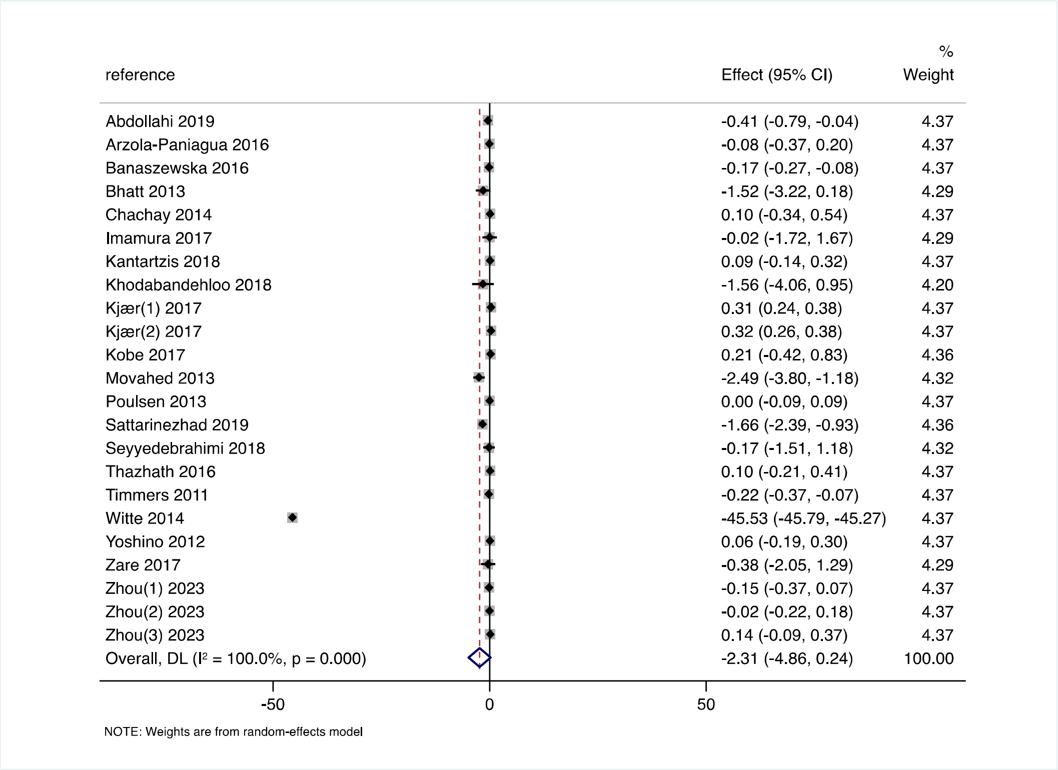

Figure S1-12-7 Forest plot of RCTs investigating the effect of resveratrol supplementation on FBG.

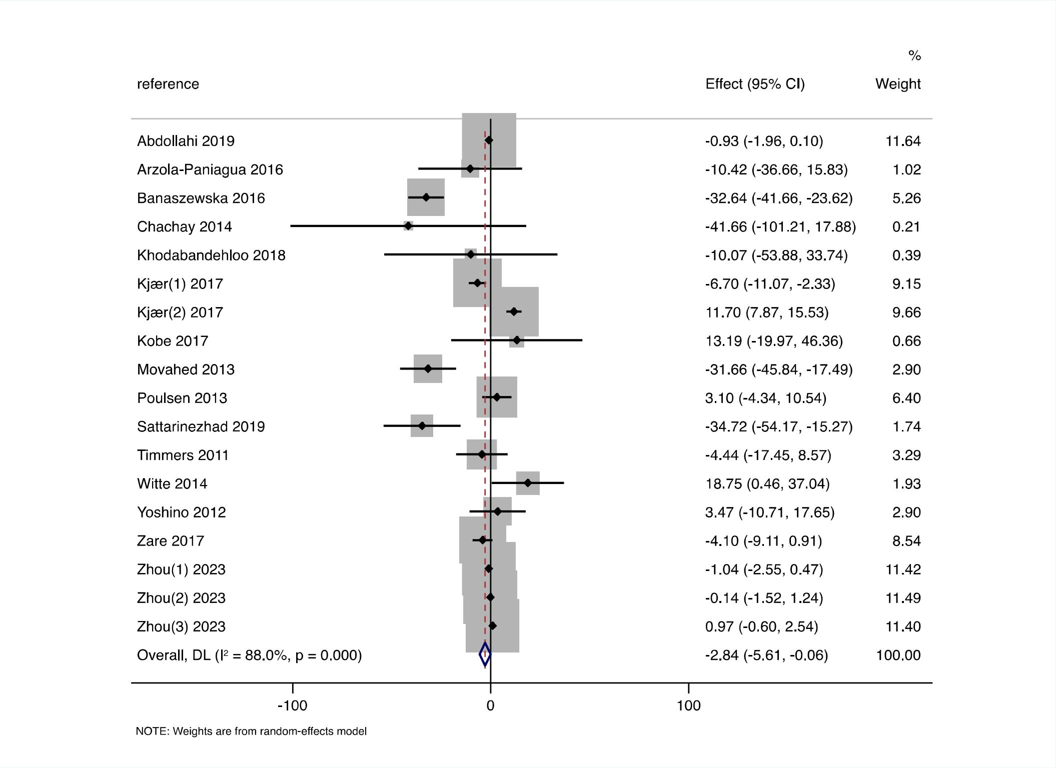

Figure S1-12-8 Forest plot of RCTs investigating the effect of resveratrol supplementation on FBI.

Appendix 3- Risk of bias

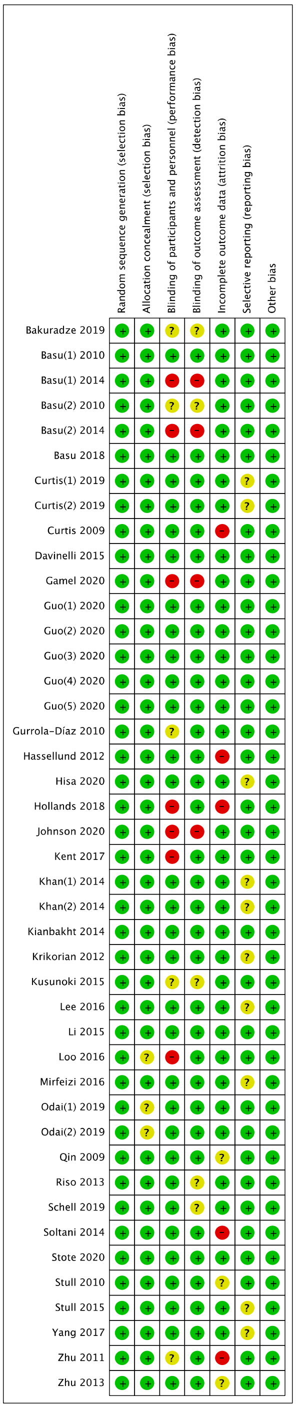

Figure S2-1 Risk of bias graph: the distribution of risk of bias judgments (Low risk of bias, Green; High risk of bias, Red; Unclear, Yellow) for each study (anthocyanin).

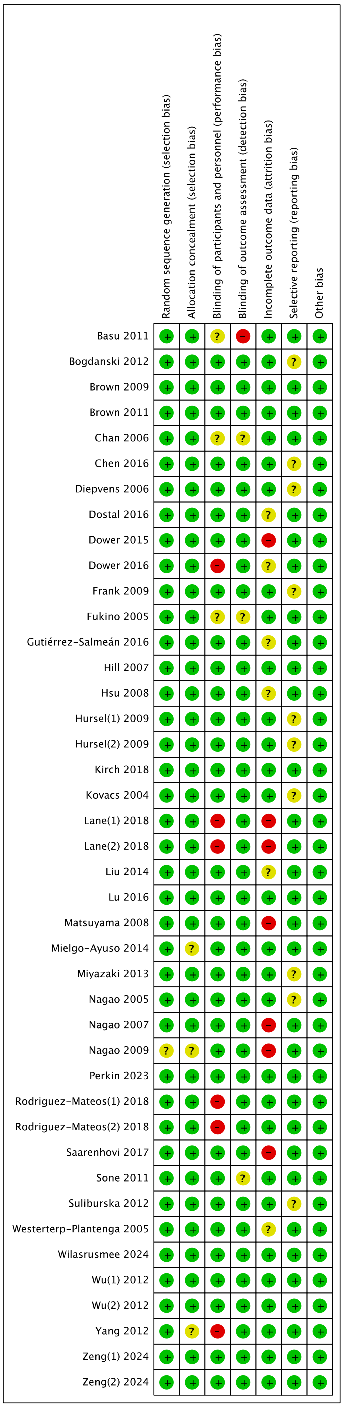

Figure S2-2 Risk of bias graph: the distribution of risk of bias judgments (Low risk of bias, Green; High risk of bias, Red; Unclear, Yellow) for each study (catechin).

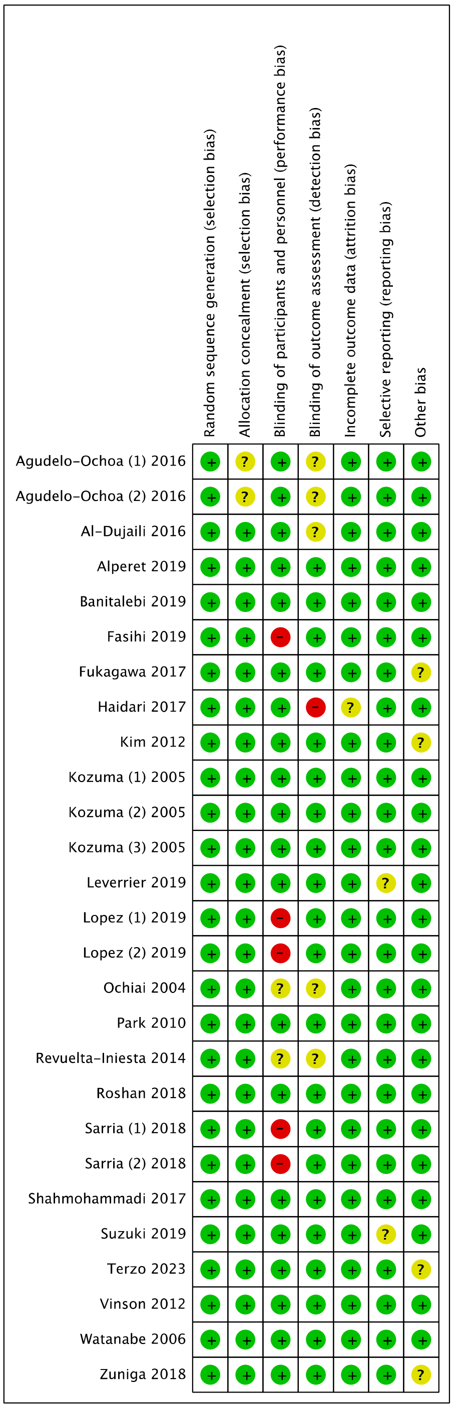

Figure S2-3 Risk of bias graph: the distribution of risk of bias judgments (Low risk of bias, Green; High risk of bias, Red; Unclear, Yellow) for each study (chlorogenic acid).

	Random sequence generation (selection bias)	Allocation concealment (selection bias)	Blinding of participants and personnel (performance bias)	Blinding of outcome assessment (detection bias)	Incomplete outcome data (attrition bias)	Selective reporting (reporting bias)	Other bias
Alidadi 2021	+	+	+	+	−	+	+
Amin 2015	+	+	+	+	−	+	+
Asadi 2019	+	+	+	+	+	?	+
Asghari 2024	+	+	+	+	+	+	+
Bateni 2021	+	+	+	+	−	+	+
Campbell 2019	+	?	+	+	−	+	+
Cicero 2020	+	+	+	+	+	+	+
Funamoto 2016	+	?	+	+	?	+	+
Heshmati 2020	+	+	+	+	+	+	+
Jamilian 2020	+	+	+	+	+	?	+
Jazayeri- Tehrani 2019	+	+	+	+	+	+	+
Kuszewski.H 2020	+	−	+	+	−	+	+
Na 2012	+	+	+	+	+	?	+
Osali 2020	+	+	+	+	?	+	+
Panahi 2015	+	?	+	+	+	+	+
Rahimi 2016	+	+	+	+	+	?	+
Santos-Parker 2017	+	?	−	+	−	+	+
Saraf-Bank 2019	+	+	+	+	−	+	+
Sohaei 2019	+	+	+	+	?	?	+
Sugawara 2012	+	+	+	+	−	+	+
Thota 2019	+	+	+	+	+	+	+
Yaikwawong 2024(1)	+	+	+	+	+	?	+
Yaikwawong 2024(2)	+	+	+	+	+	?	+
Zare'i 2024	+	+	+	+	+	+	+

Figure S2-4 Risk of bias graph: the distribution of risk of bias judgments (Low risk of bias, Green; High risk of bias, Red; Unclear, Yellow) for each study (curcumin).

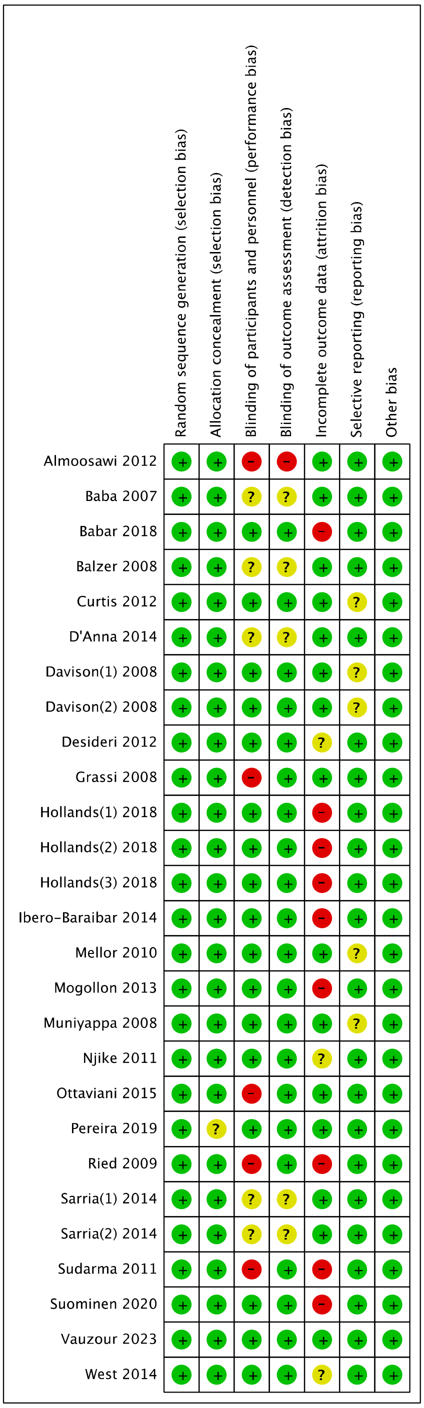

Figure S2-5 Risk of bias graph: the distribution of risk of bias judgments (Low risk of bias, Green; High risk of bias, Red; Unclear, Yellow) for each study (flavanol).

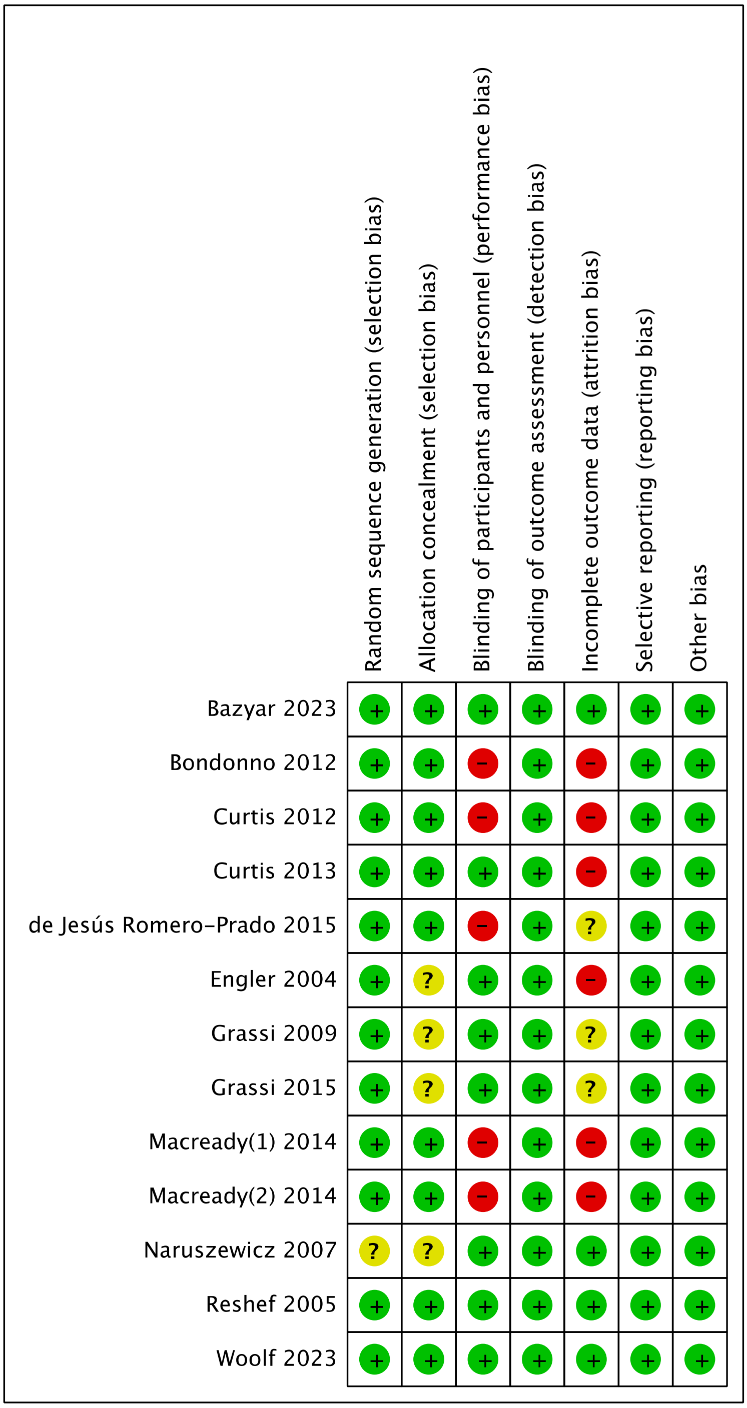

Figure S2-6 Risk of bias graph: the distribution of risk of bias judgments (Low risk of bias, Green; High risk of bias, Red; Unclear, Yellow) for each study (flavonoid).

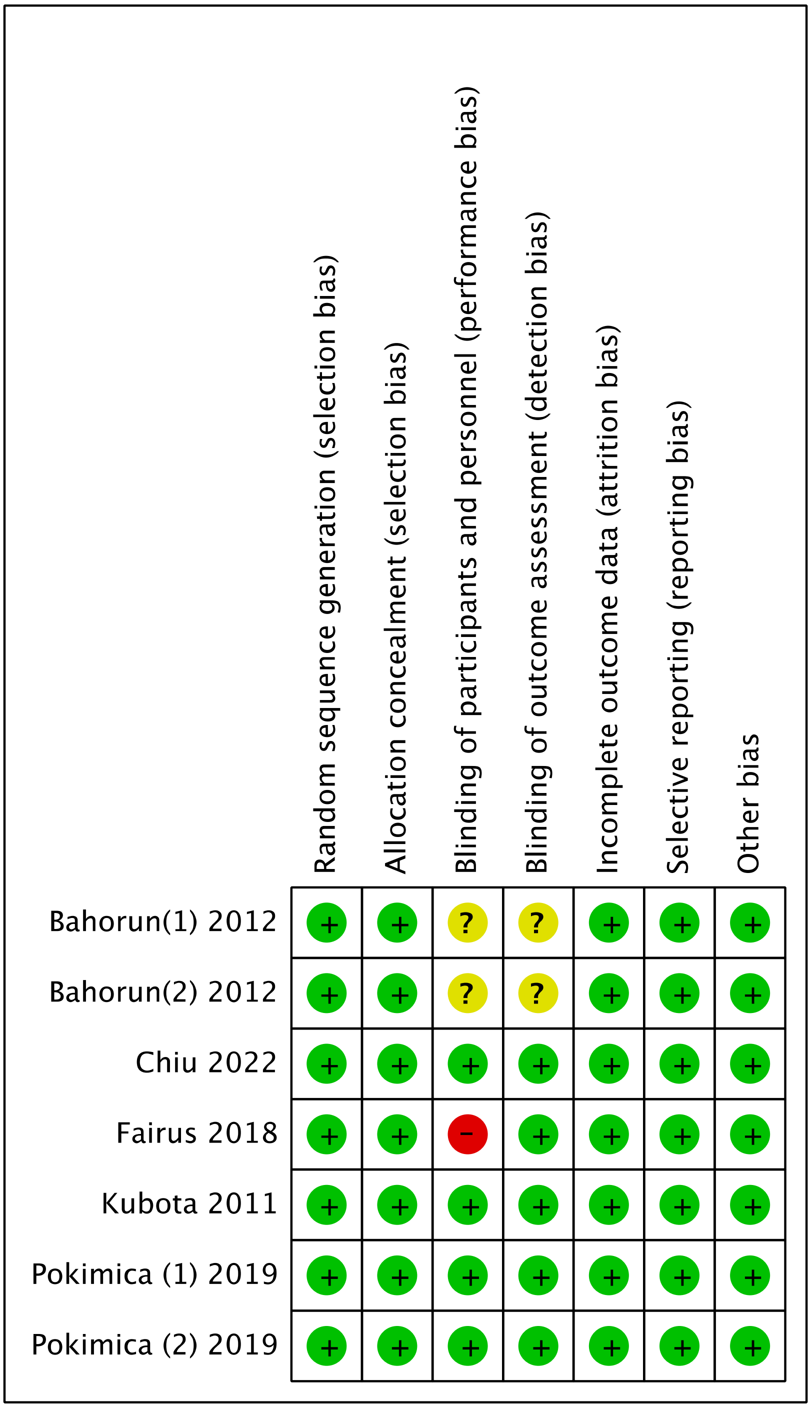

Figure S2-7 Risk of bias graph: the distribution of risk of bias judgments (Low risk of bias, Green; High risk of bias, Red; Unclear, Yellow) for each study (gallic acid).

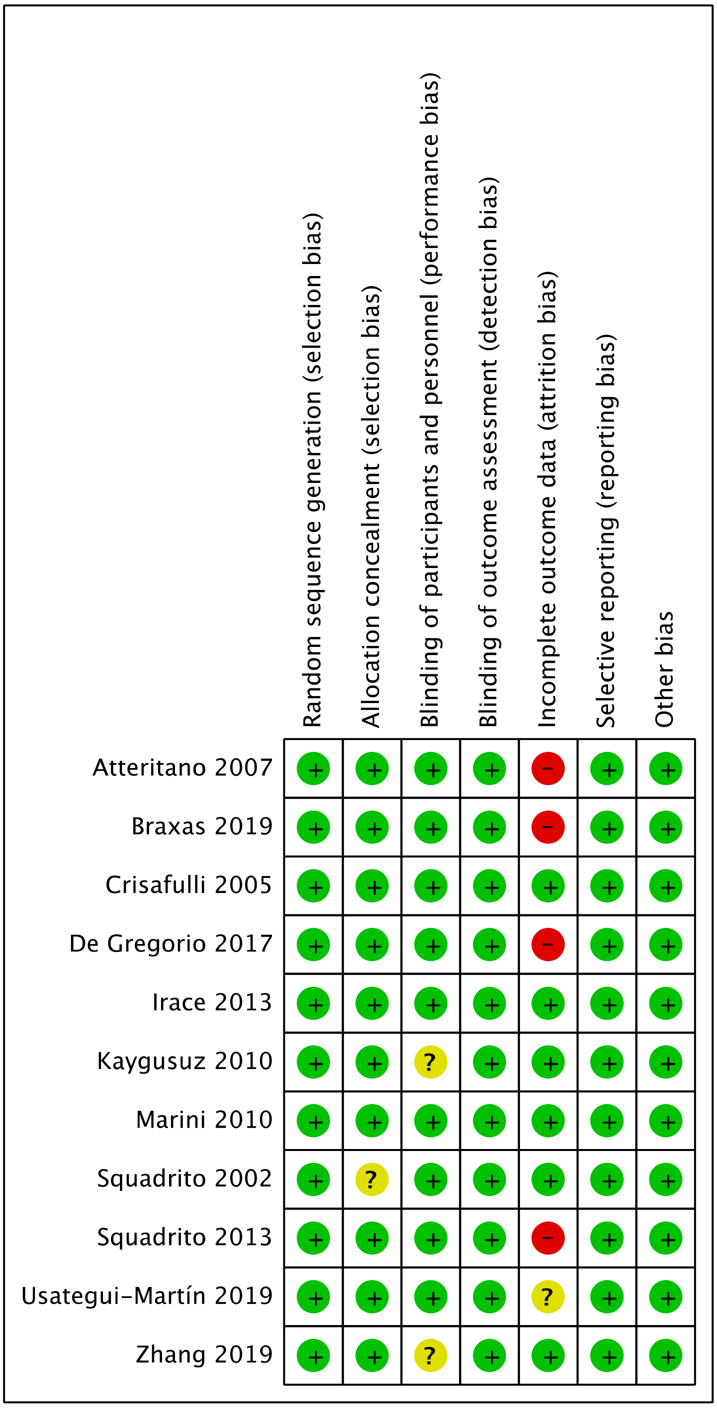

Figure S2-8 Risk of bias graph: the distribution of risk of bias judgments (Low risk of bias, Green; High risk of bias, Red; Unclear, Yellow) for each study (genistein).

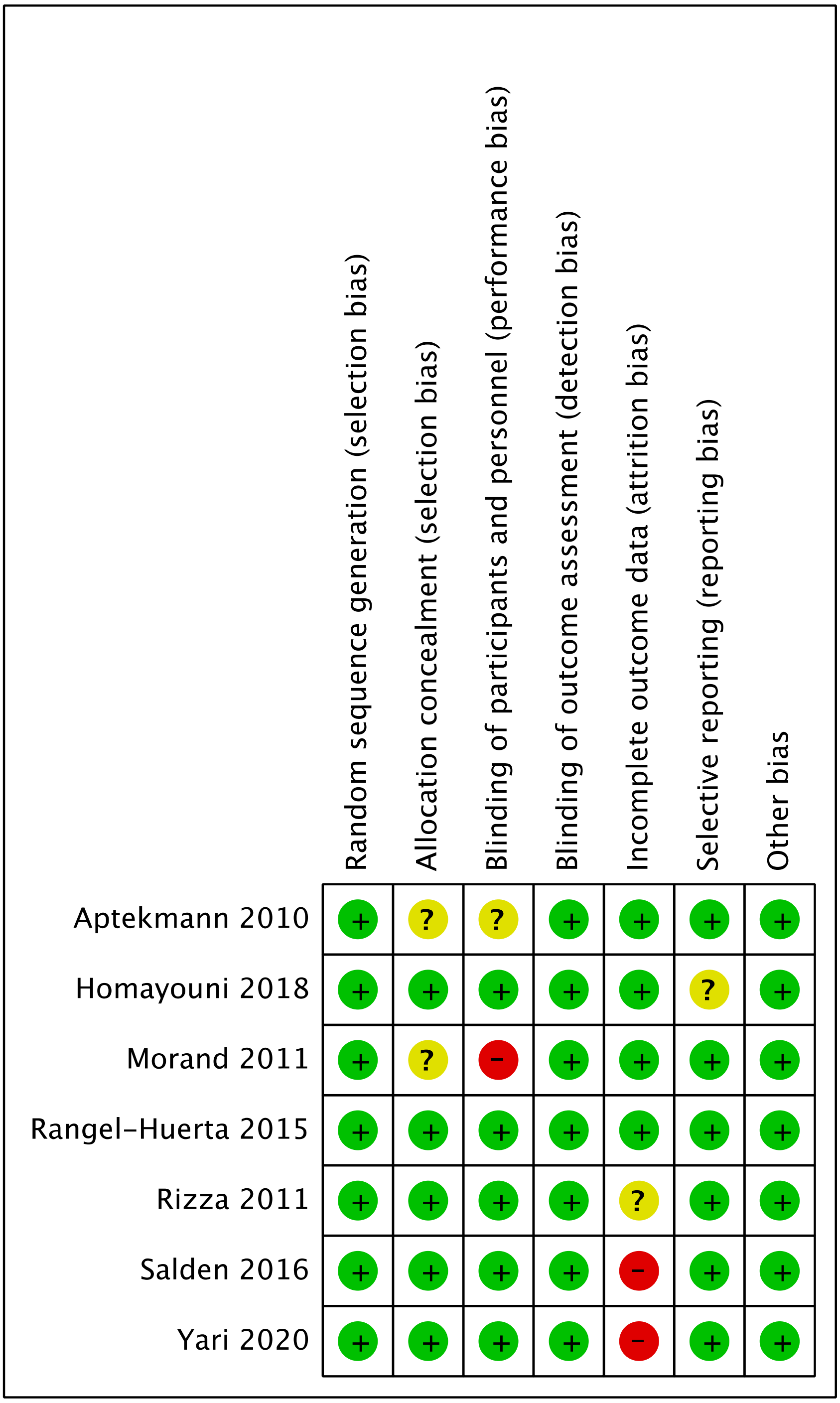

Figure S2-9 Risk of bias graph: the distribution of risk of bias judgments (Low risk of bias, Green; High risk of bias, Red; Unclear, Yellow) for each study (hesperidin).

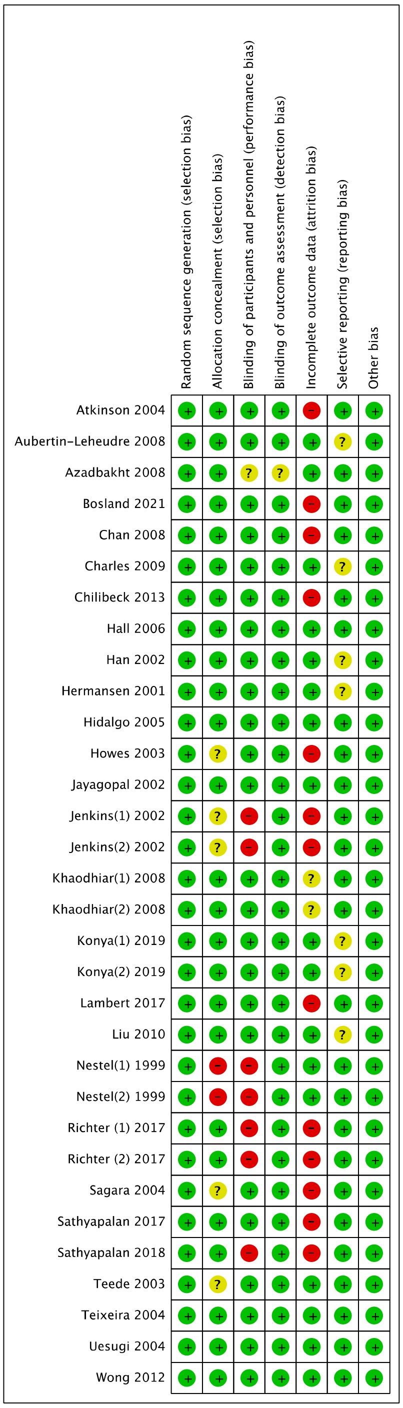

Figure S2-10 Risk of bias graph: the distribution of risk of bias judgments (Low risk of bias, Green; High risk of bias, Red; Unclear, Yellow) for each study (isoflavone).

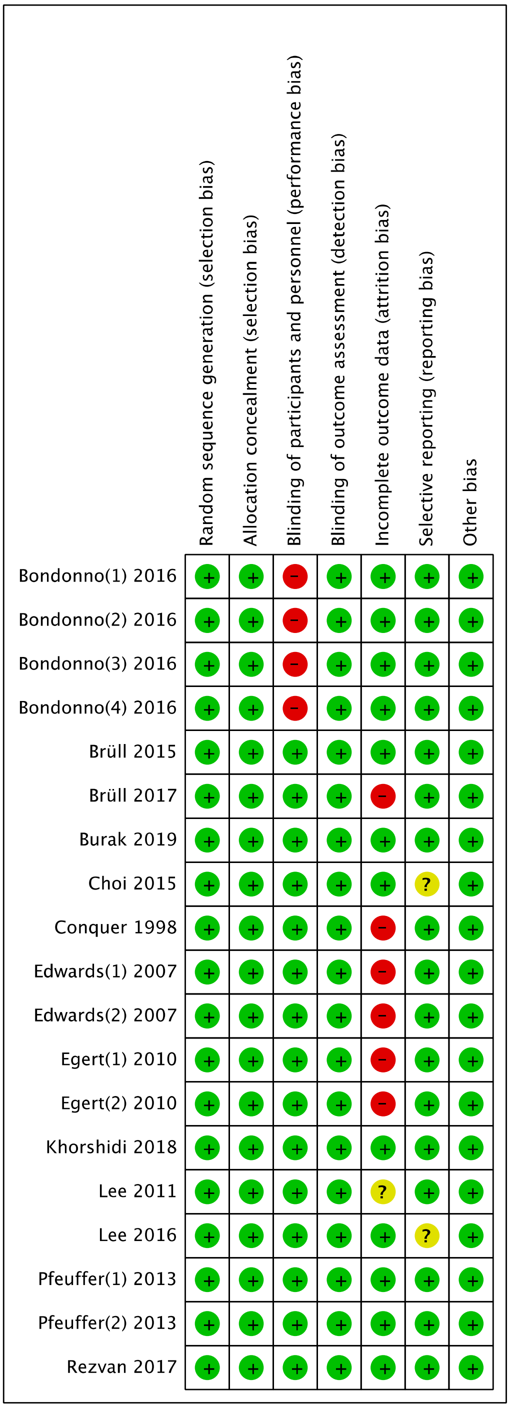

Figure S2-11 Risk of bias graph: the distribution of risk of bias judgments (Low risk of bias, Green; High risk of bias, Red; Unclear, Yellow) for each study (quercetin).

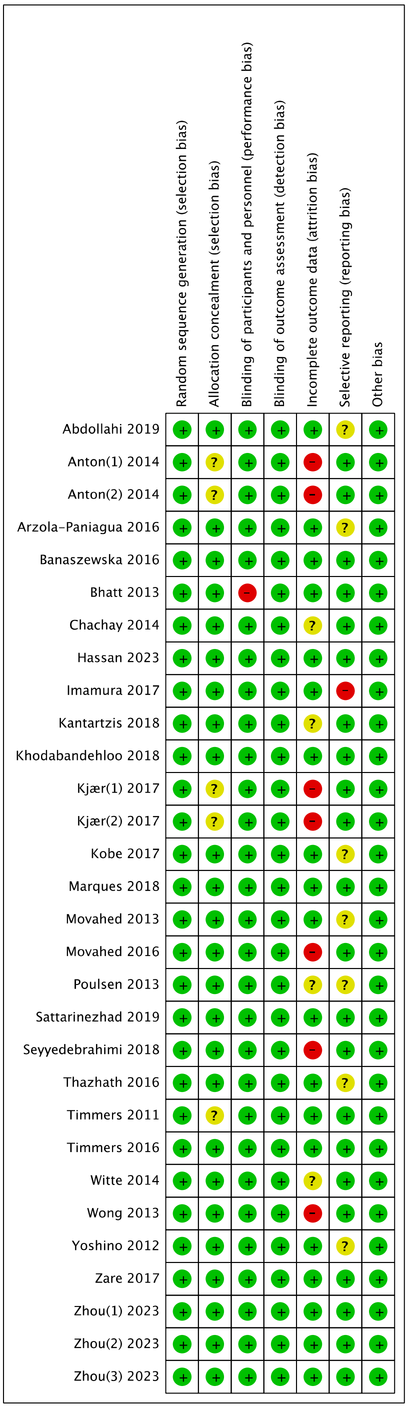

Figure S2-12 Risk of bias graph: the distribution of risk of bias judgments (Low risk of bias, Green; High risk of bias, Red; Unclear, Yellow) for each study (resveratrol).

Appendix 4 - Funnel plot

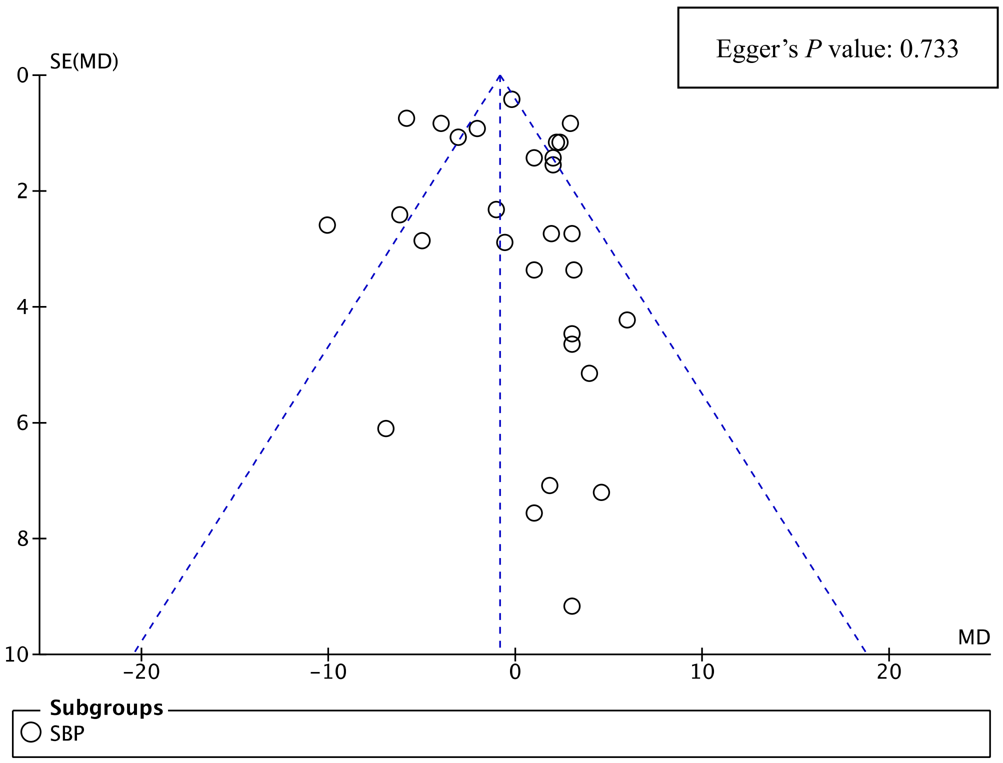

Figure S3-1-1 Funnel plot of RCTs investigating the effect of anthocyanin supplementation on SBP.

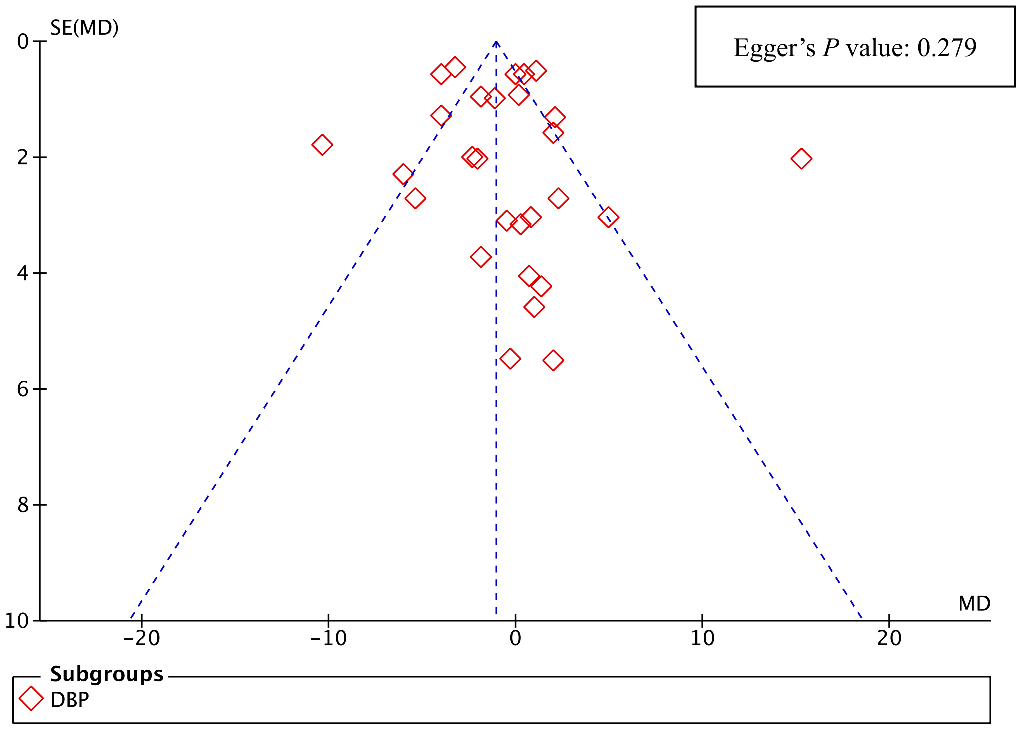

Figure S3-1-2 Funnel plot of RCTs investigating the effect of anthocyanin supplementation on DBP.

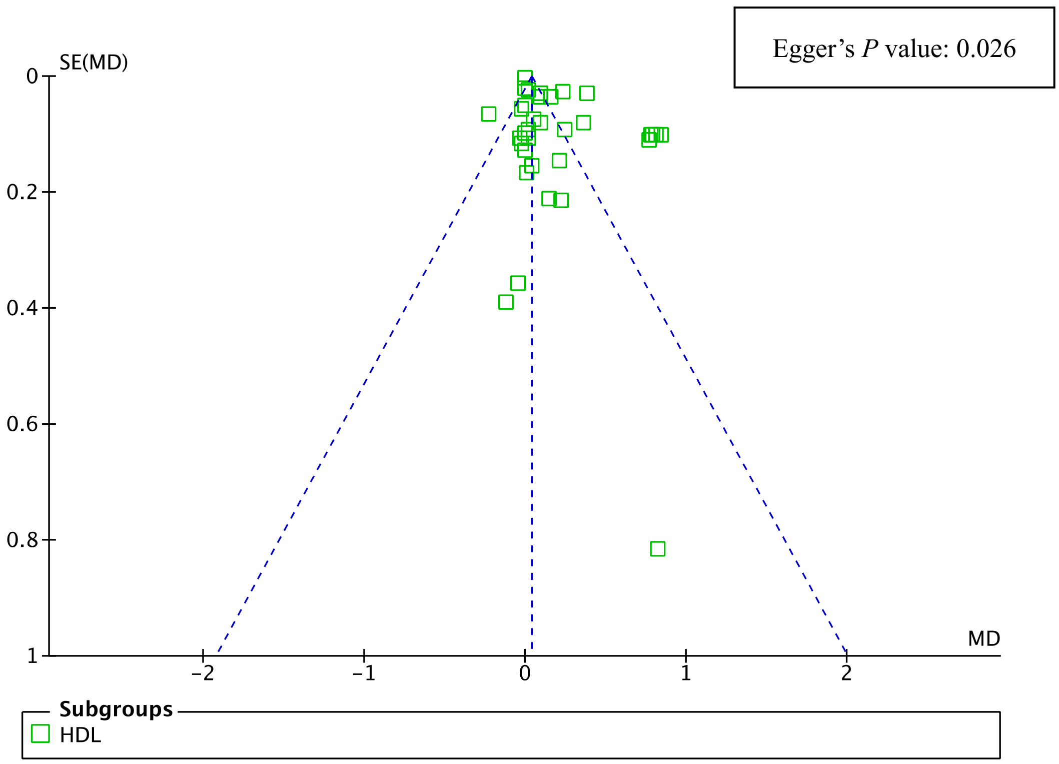

Figure S3-1-3 Funnel plot of RCTs investigating the effect of anthocyanin supplementation on HDL-C.

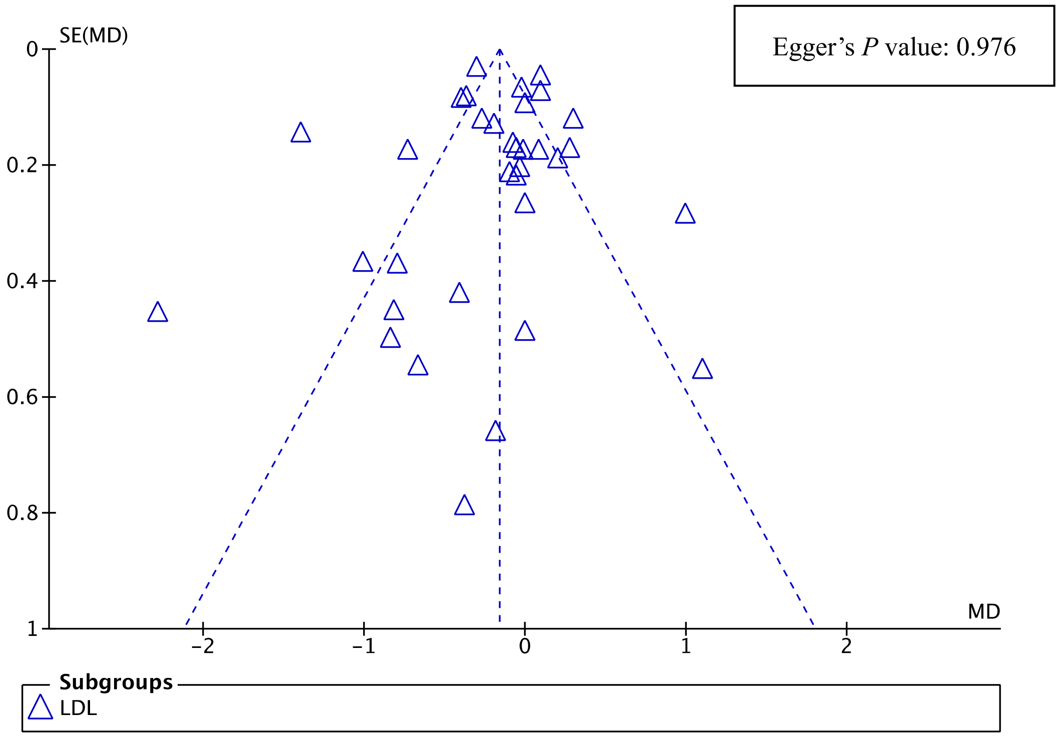

Figure S3-1-4 Funnel plot of RCTs investigating the effect of anthocyanin supplementation on LDL-C.

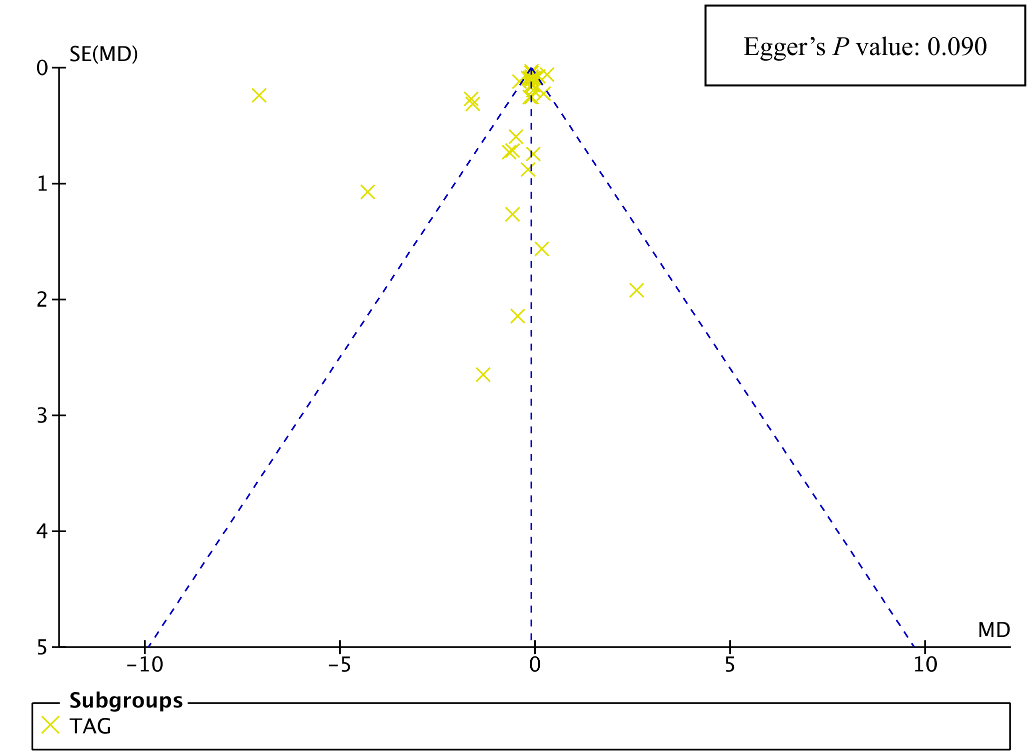

Figure S3-1-5 Funnel plot of RCTs investigating the effect of anthocyanin supplementation on TG

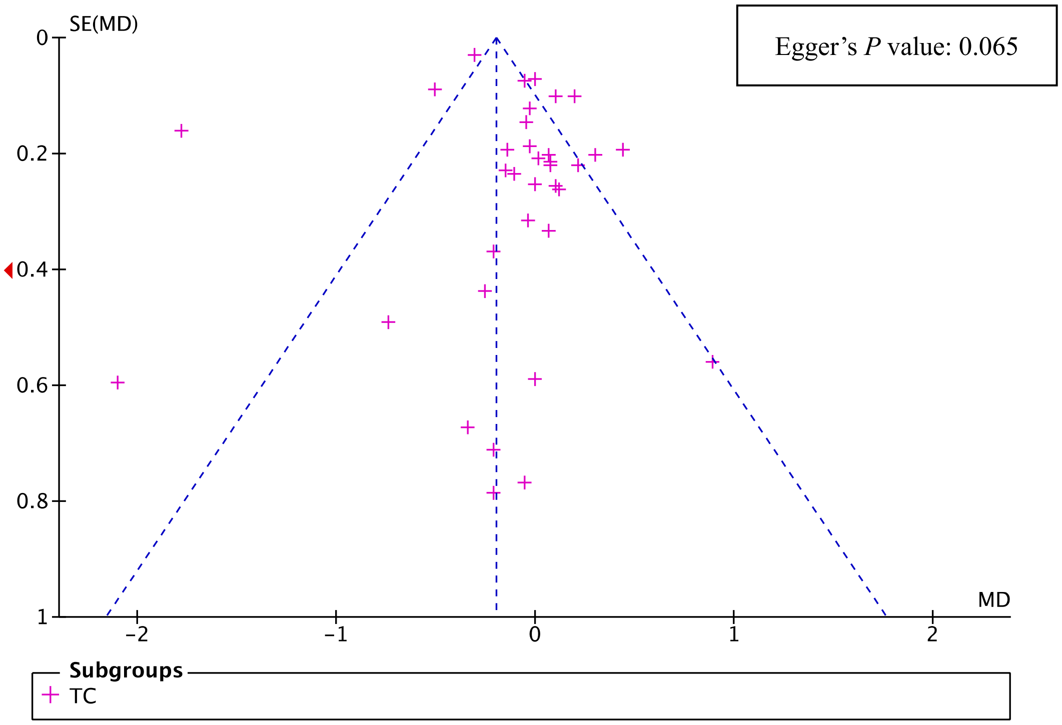

Figure S3-1-6 Funnel plot of RCTs investigating the effect of anthocyanin supplementation on TC.

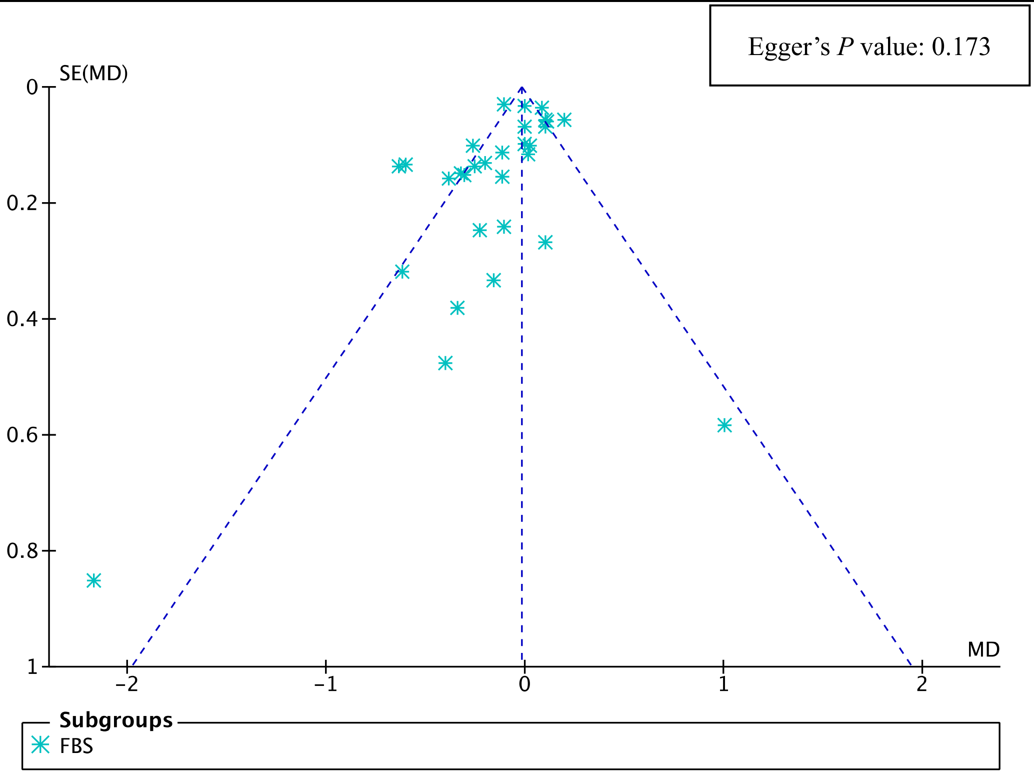

Figure S3-1-7 Funnel plot of RCTs investigating the effect of anthocyanin supplementation on FBG.

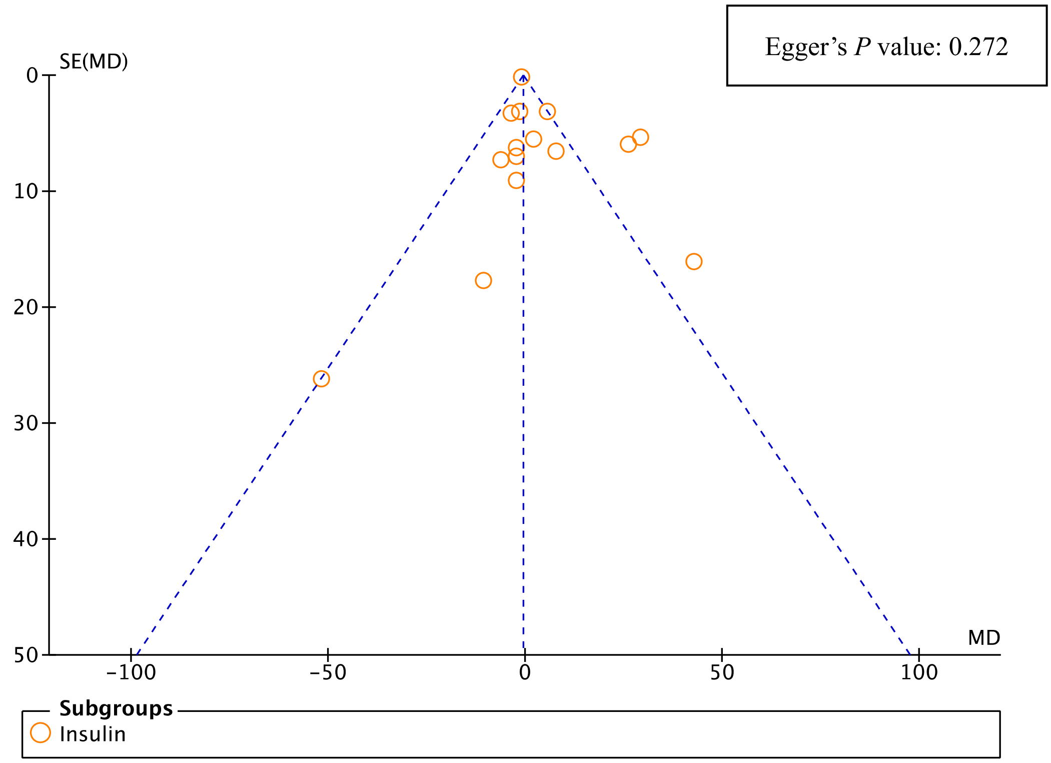

Figure S3-1-8 Funnel plot of RCTs investigating the effect of anthocyanin supplementation on FBI.

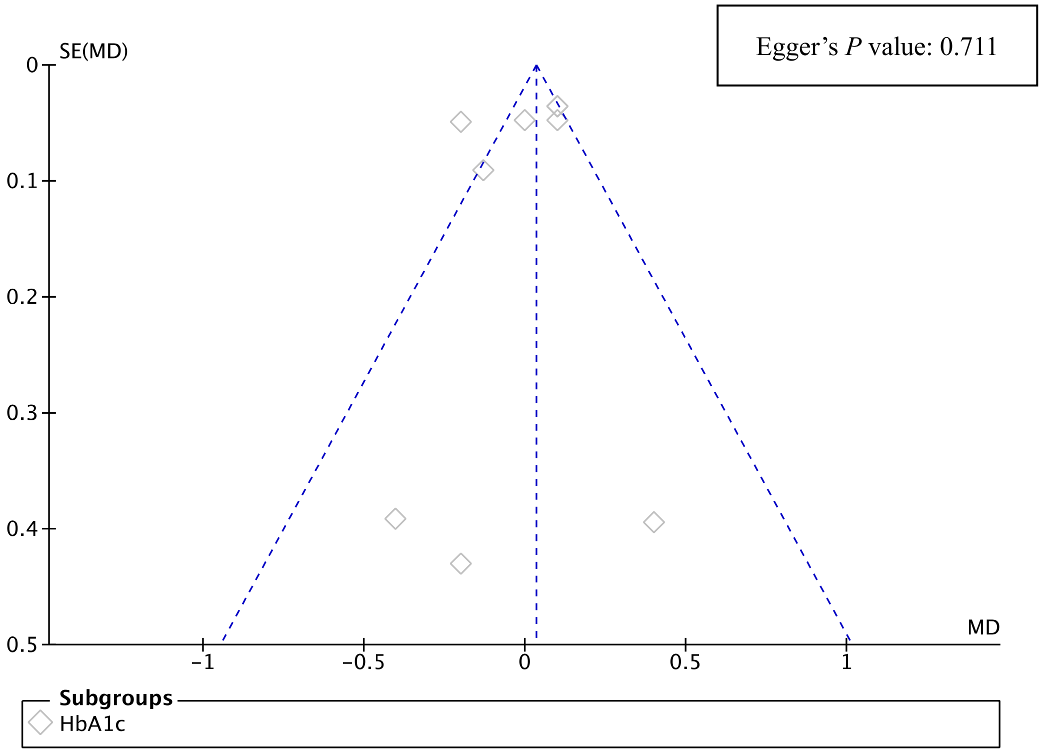

Figure S3-1-9 Funnel plot of RCTs investigating the effect of anthocyanin supplementation on A1C.

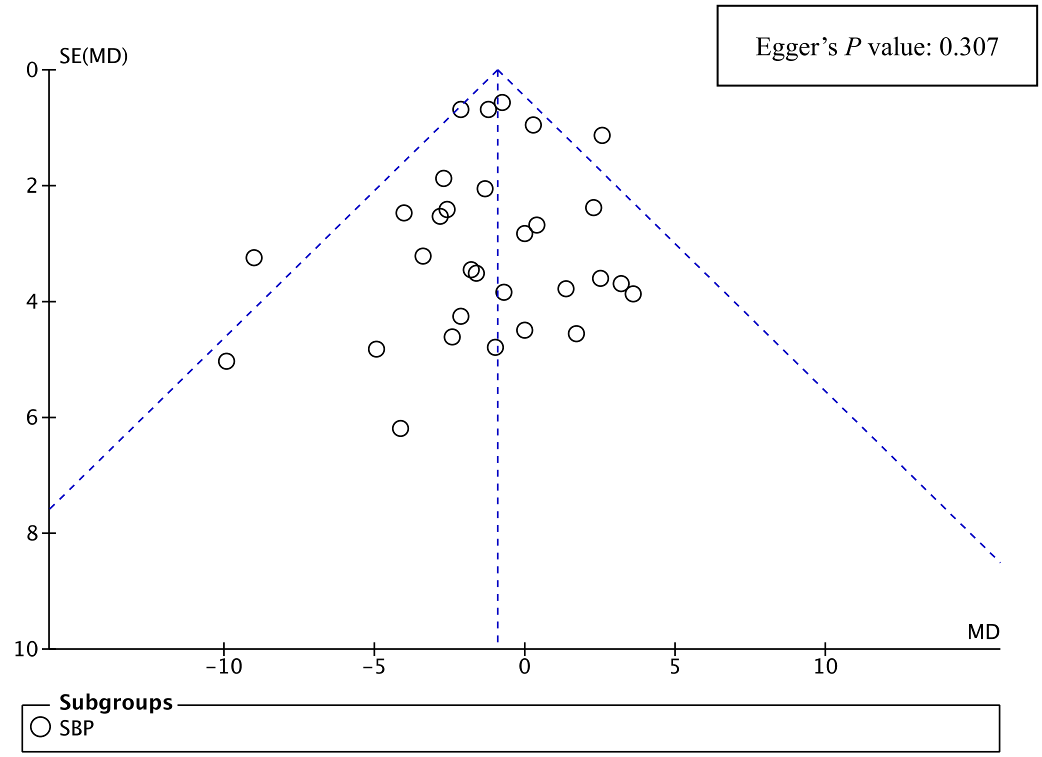

Figure S3-2-1 Funnel plot of RCTs investigating the effect of catechin supplementation on SBP.

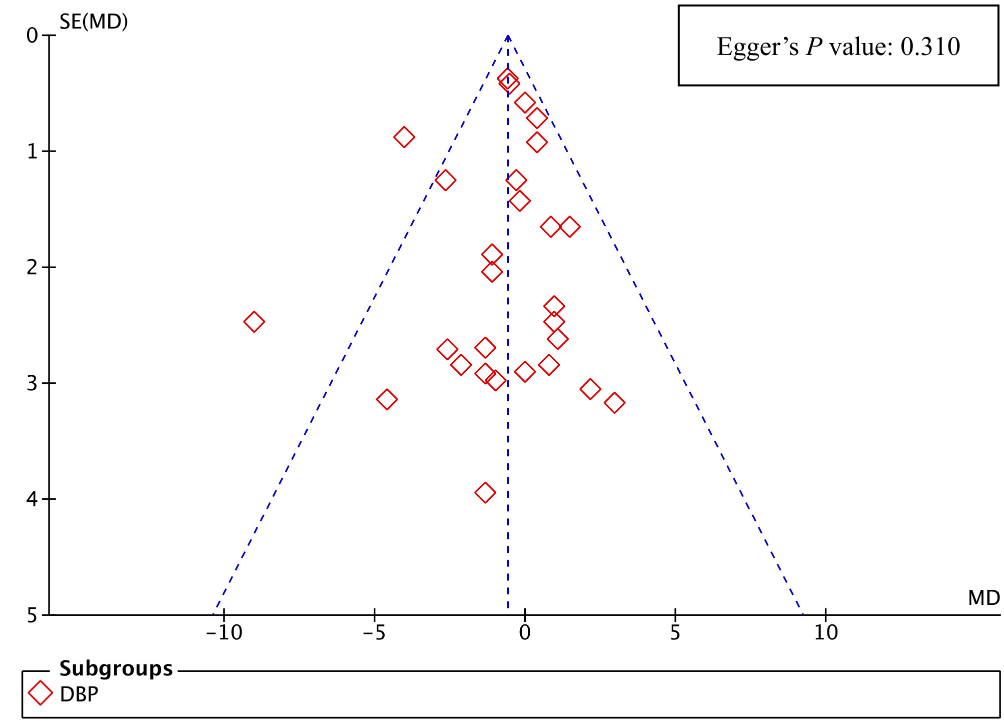

Figure S3-2-2 Funnel plot of RCTs investigating the effect of catechin supplementation on DBP.

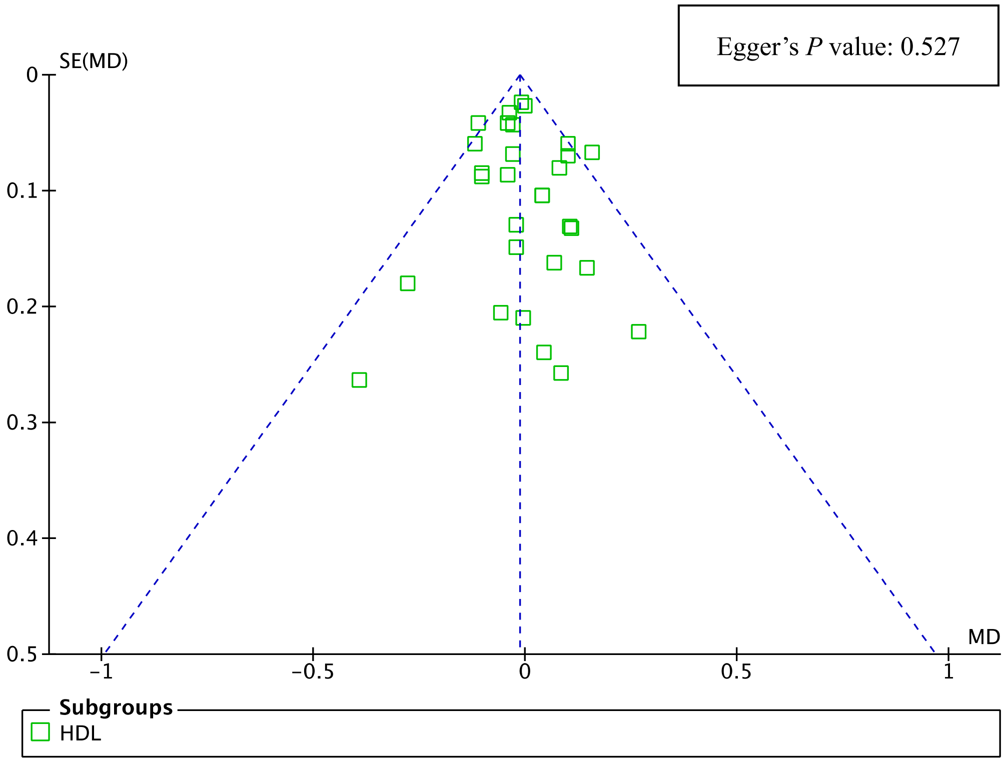

Figure S3-2-3 Funnel plot of RCTs investigating the effect of catechin supplementation on HDL-C.

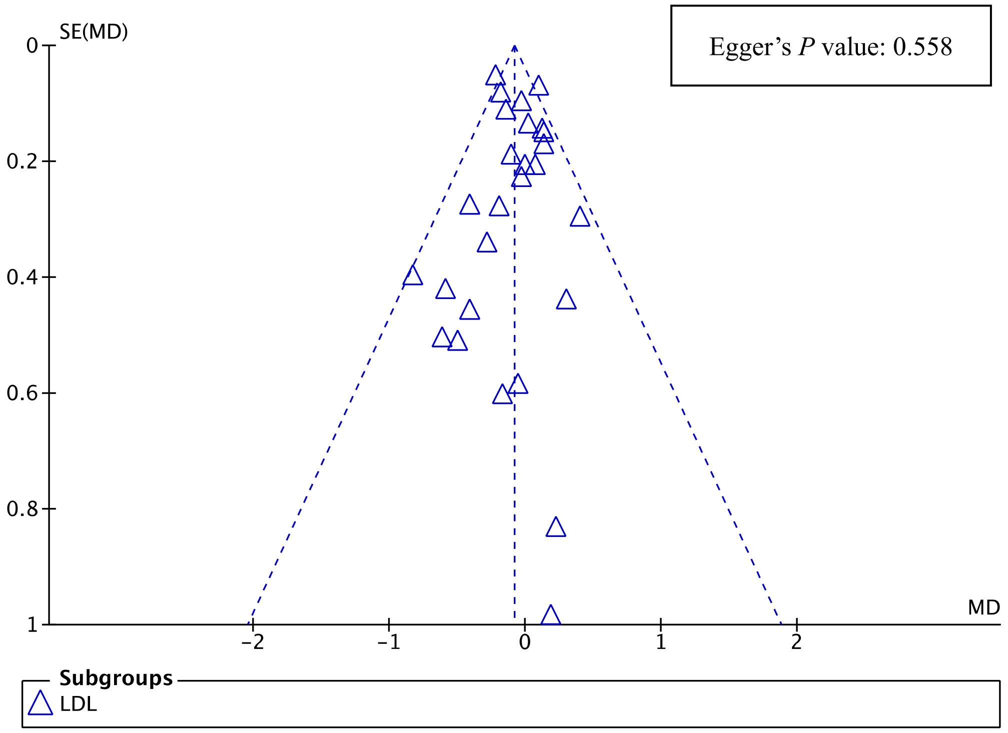

Figure S3-2-4 Funnel plot of RCTs investigating the effect of catechin supplementation on LDL-C.

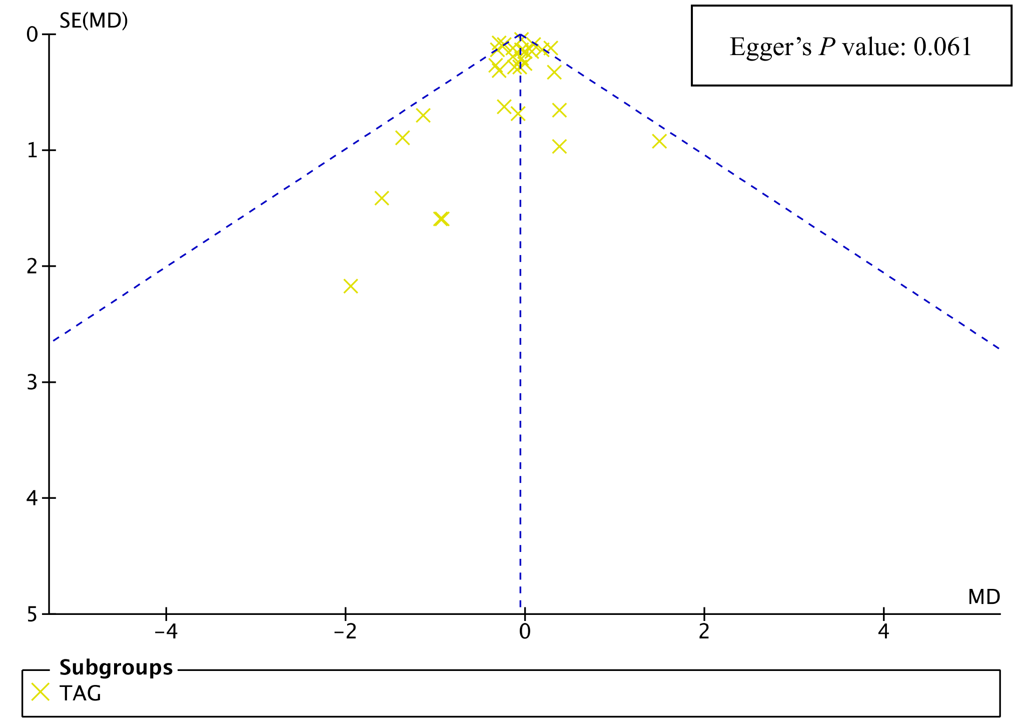

Figure S3-2-5 Funnel plot of RCTs investigating the effect of catechin supplementation on TG

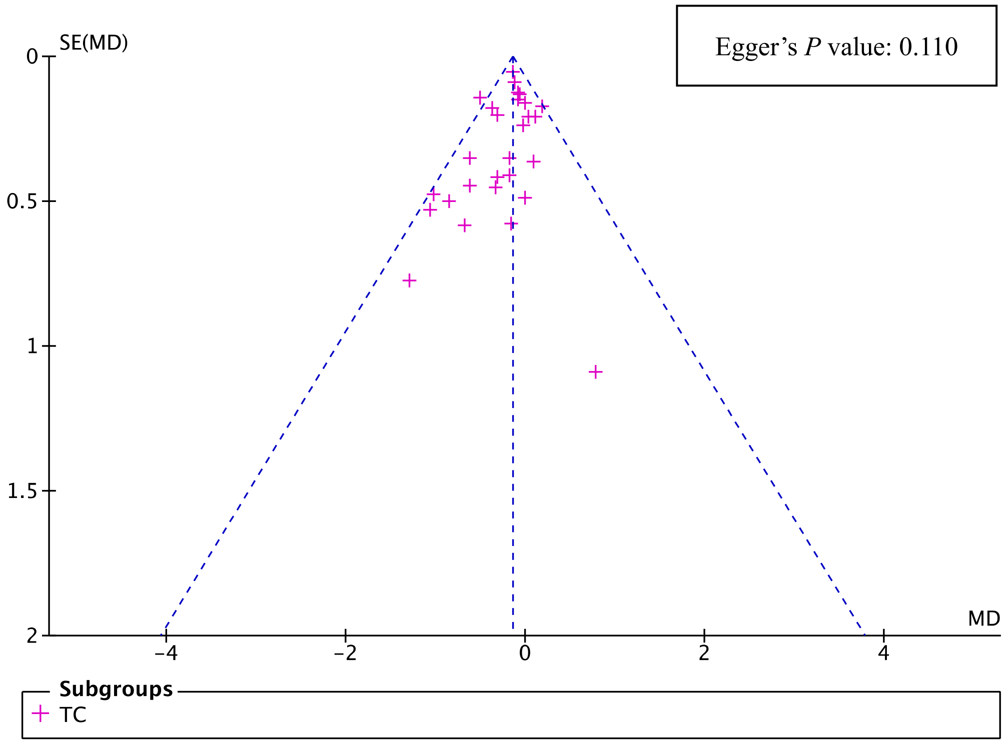

Figure S3-2-6 Funnel plot of RCTs investigating the effect of catechin supplementation on TC.

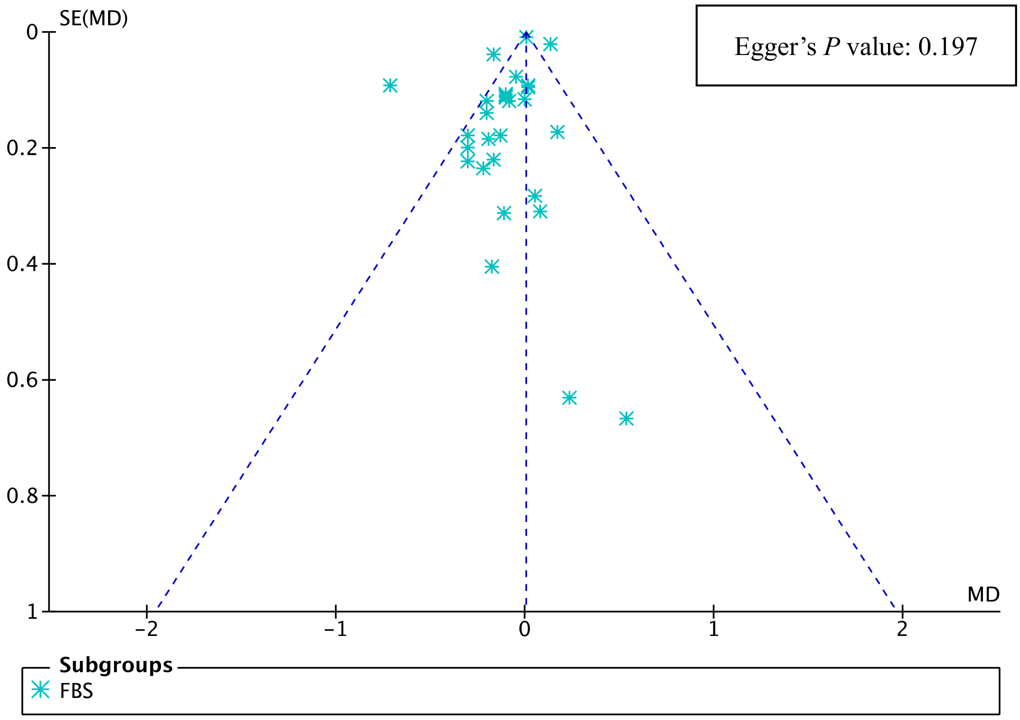

Figure S3-2-7 Funnel plot of RCTs investigating the effect of catechin supplementation on FBG.

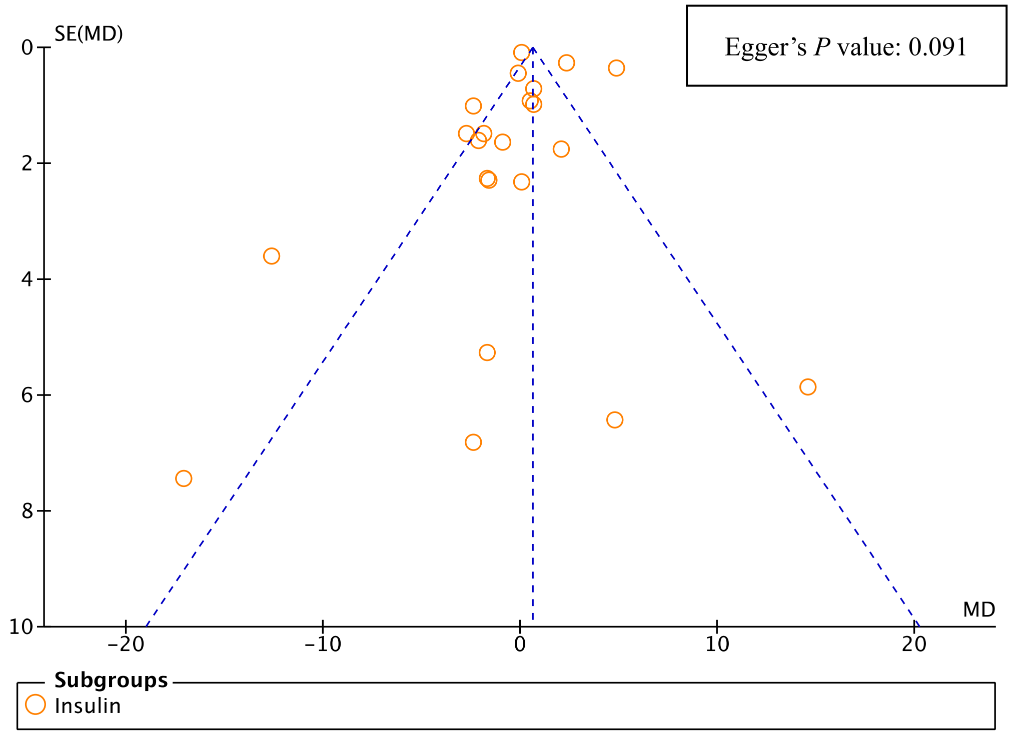

Figure S3-2-8 Funnel plot of RCTs investigating the effect of catechin supplementation on FBI.

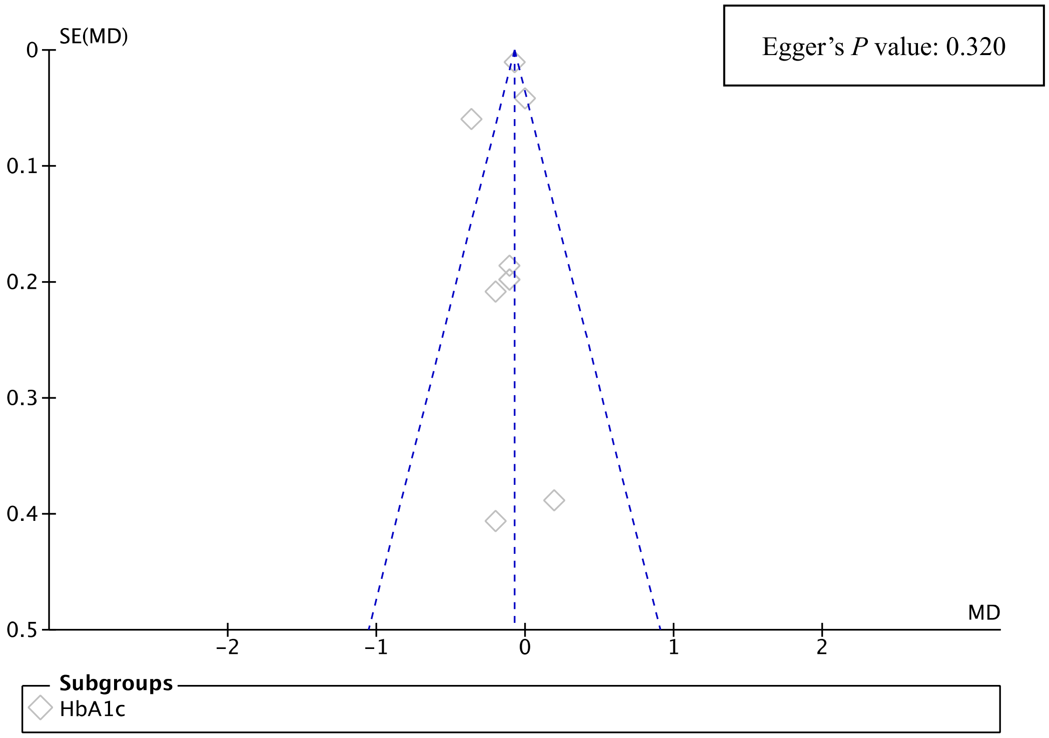

Figure S3-2-9 Funnel plot of RCTs investigating the effect of catechin supplementation on A1C.

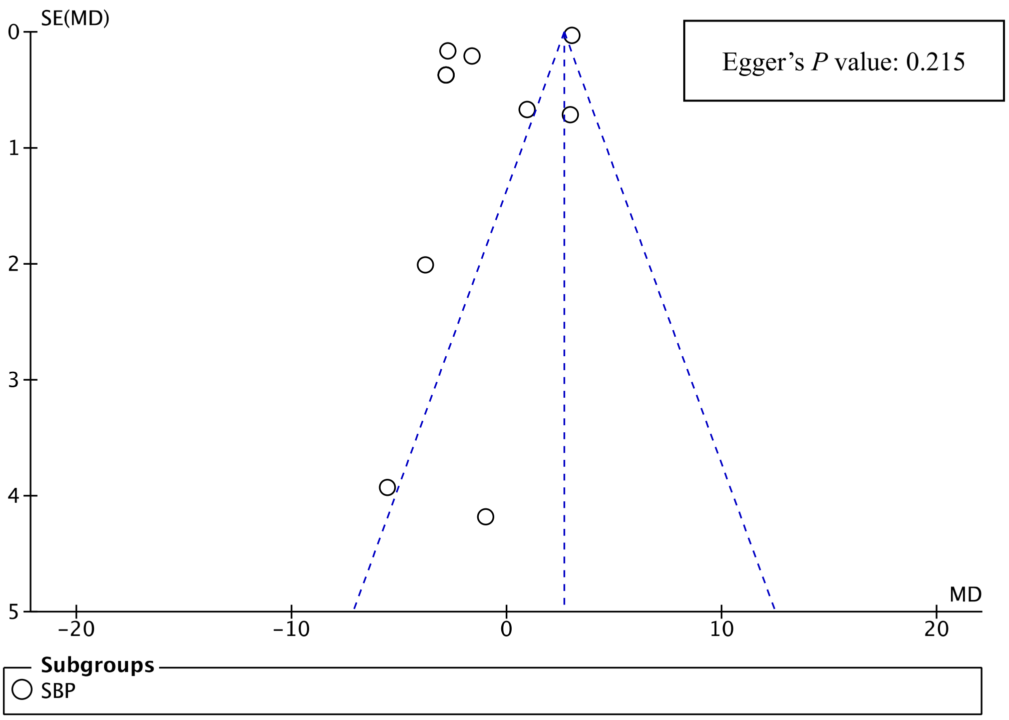

Figure S3-3-1 Funnel plot of RCTs investigating the effect of chlorogenic acid supplementation on SBP.

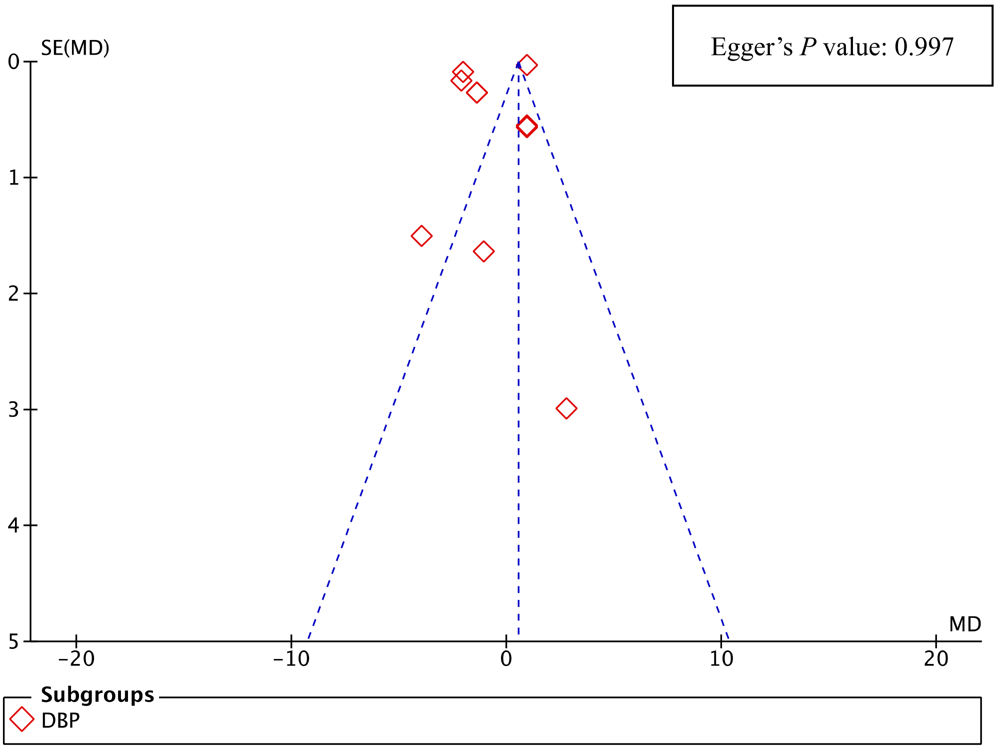

Figure S3-3-2 Funnel plot of RCTs investigating the effect of chlorogenic acid supplementation on DBP.

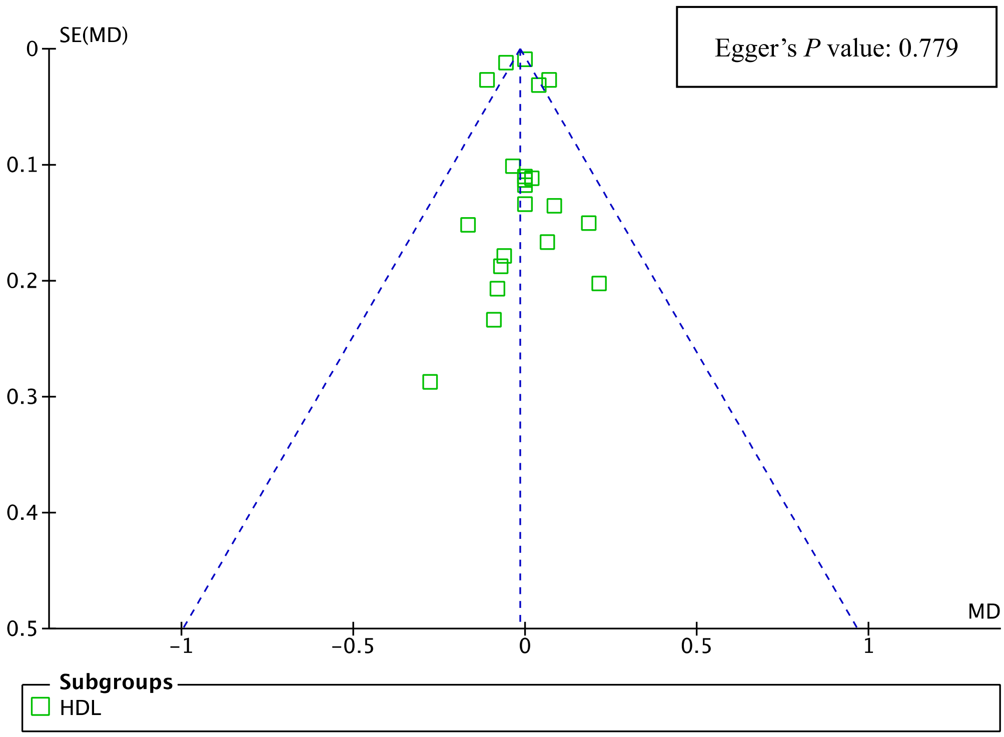

Figure S3-3-3 Funnel plot of RCTs investigating the effect of chlorogenic acid supplementation on HDL-C.

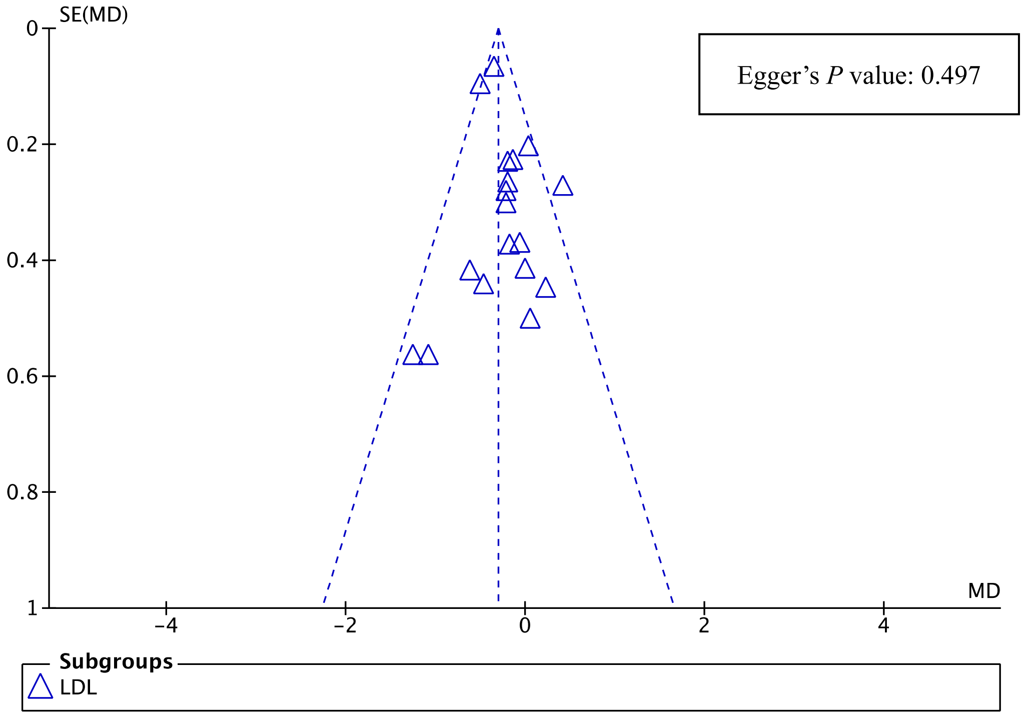

Figure S3-3-4 Funnel plot of RCTs investigating the effect of chlorogenic acid supplementation on LDL-C.

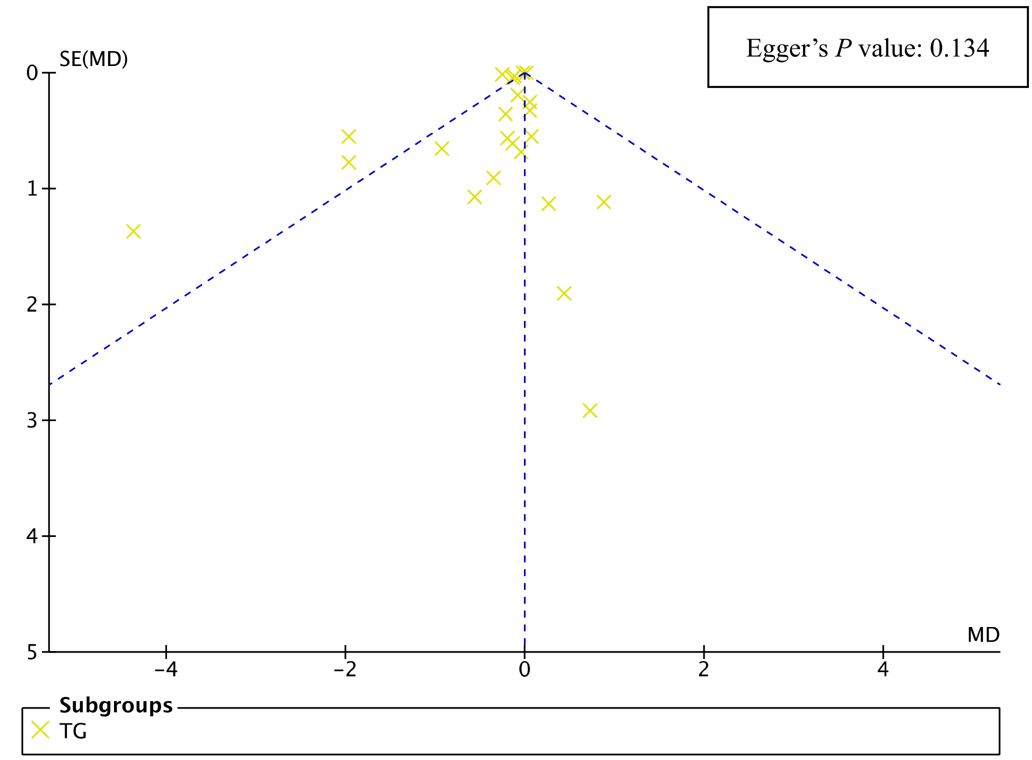

Figure S3-3-5 Funnel plot of RCTs investigating the effect of chlorogenic acid supplementation on TG

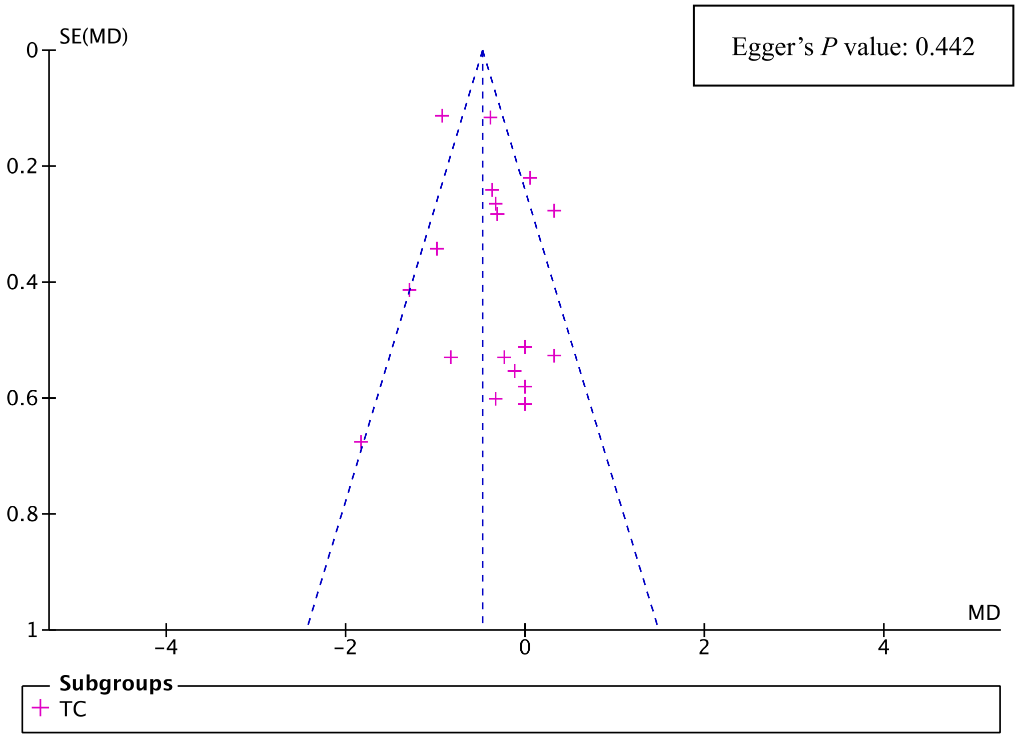

Figure S3-3-6 Funnel plot of RCTs investigating the effect of chlorogenic acid supplementation on TC.

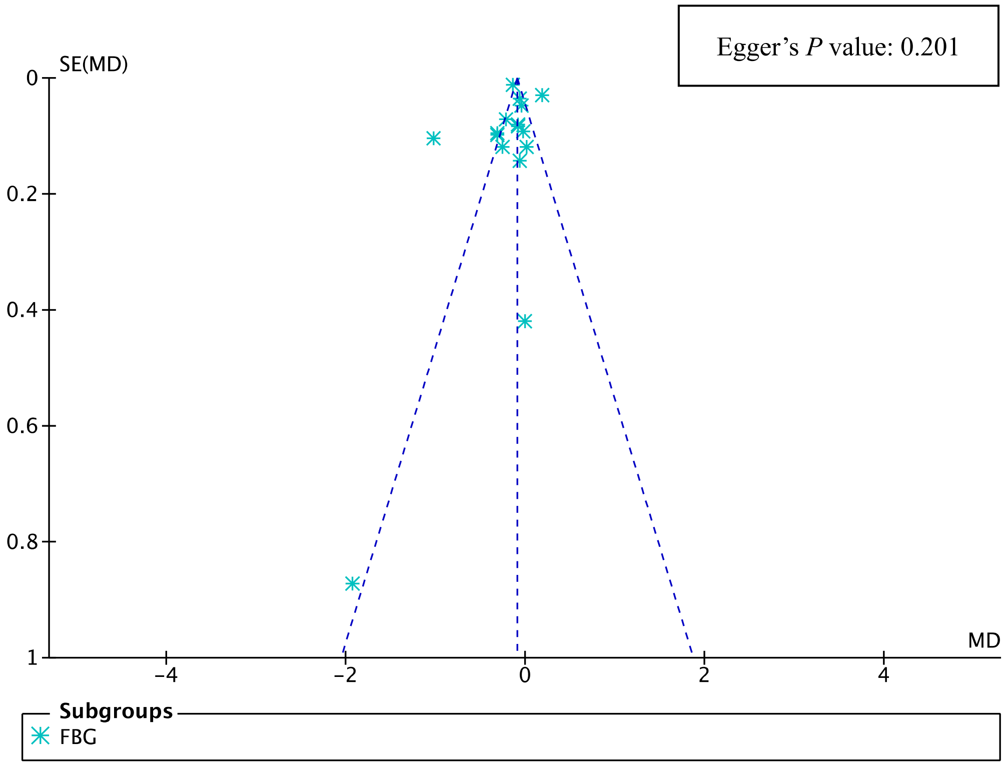

Figure S3-3-7 Funnel plot of RCTs investigating the effect of chlorogenic acid supplementation on FBG.

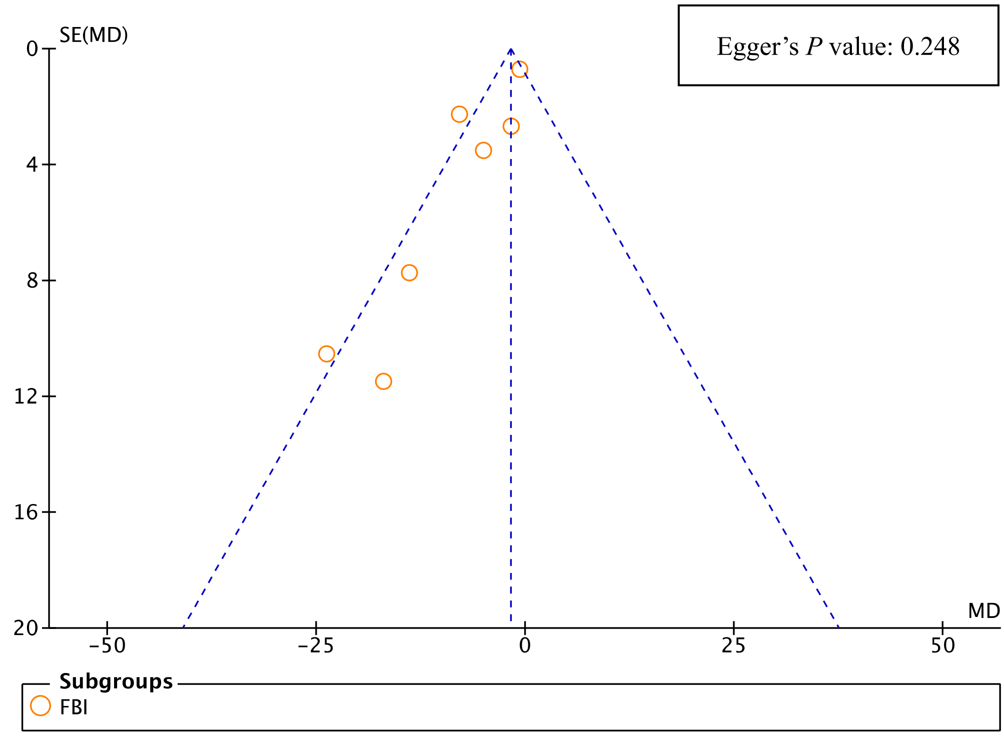

Figure S3-3-8 Funnel plot of RCTs investigating the effect of chlorogenic acid supplementation on FBI.

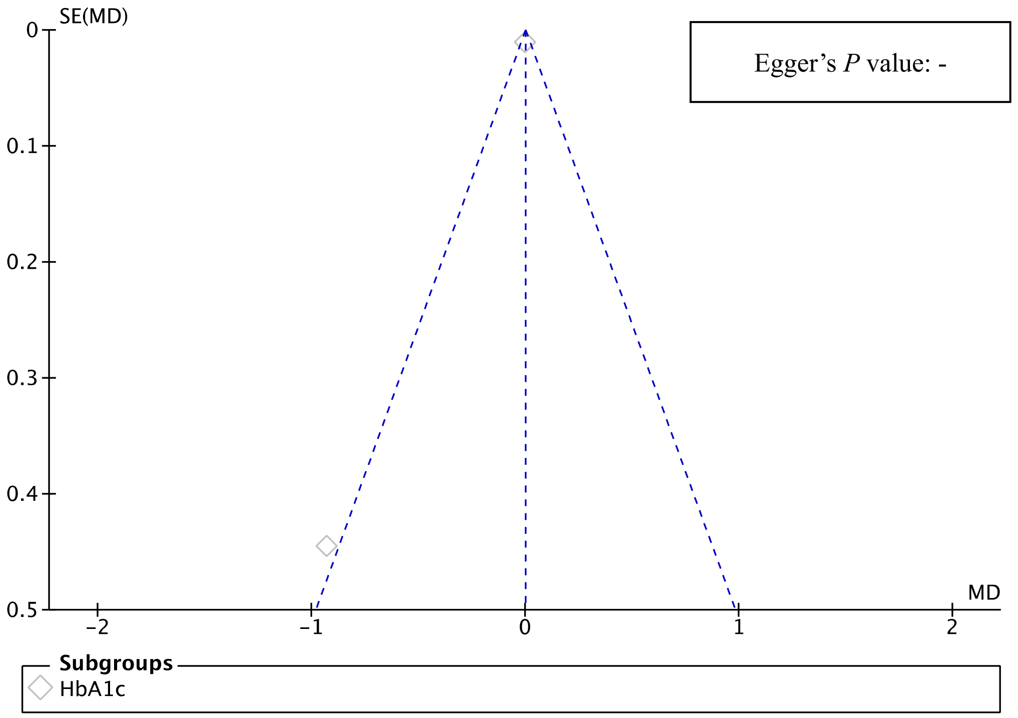

Figure S3-3-9 Funnel plot of RCTs investigating the effect of chlorogenic acid supplementation on A1C.

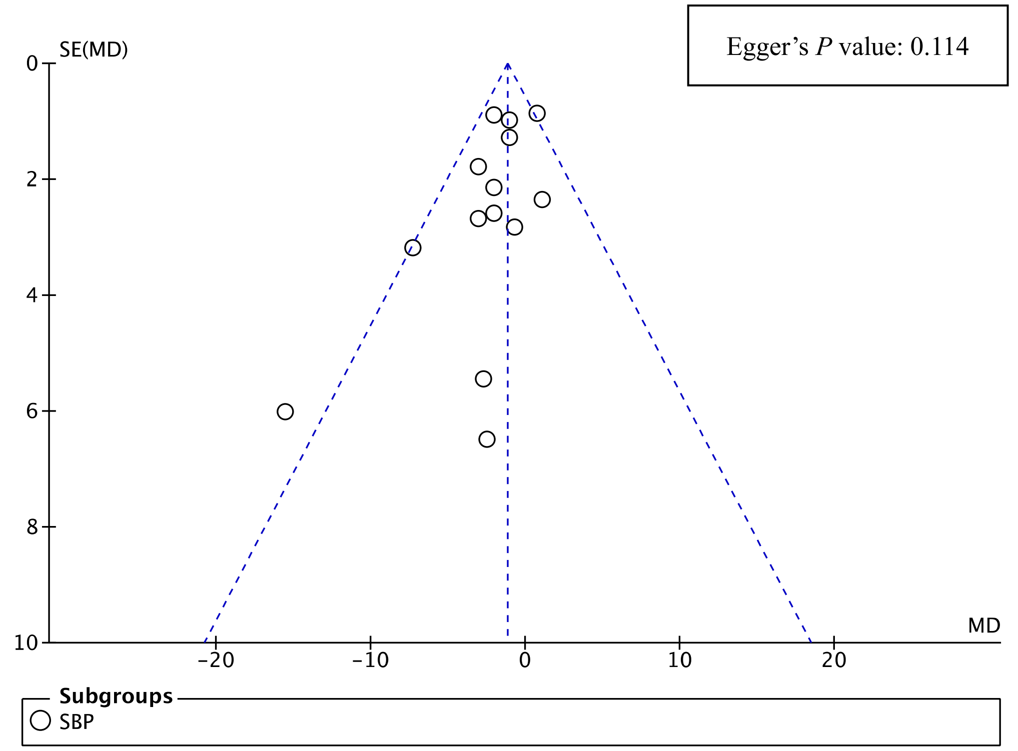

Figure S3-4-1 Funnel plot of RCTs investigating the effect of curcumin supplementation on SBP.

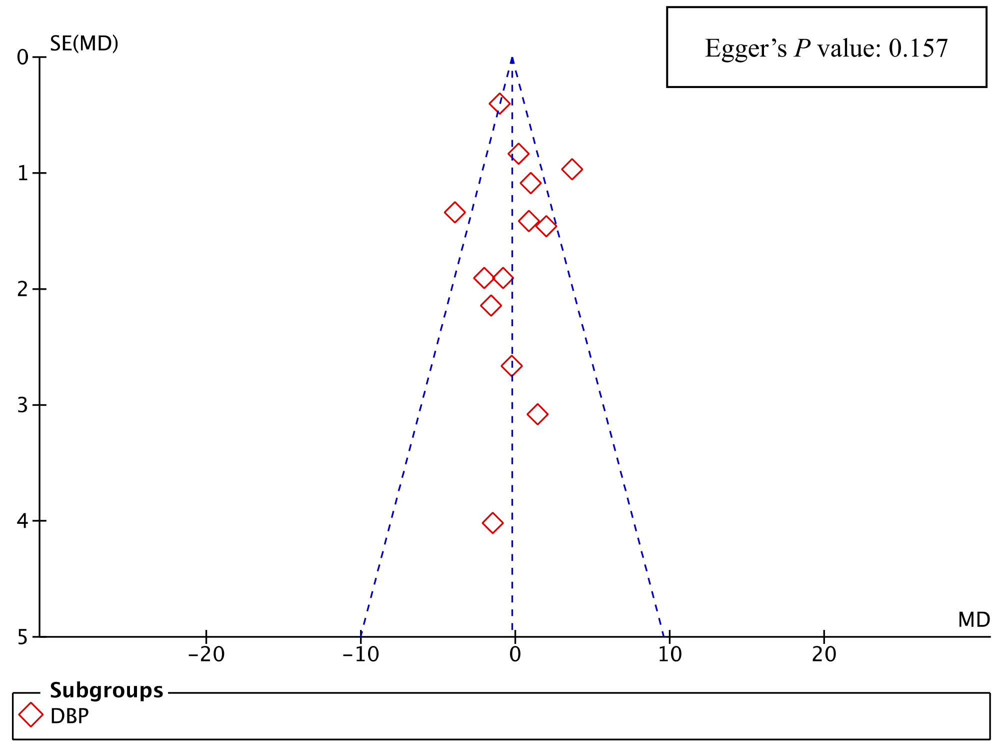

Figure S3-4-2 Funnel plot of RCTs investigating the effect of curcumin supplementation on DBP.

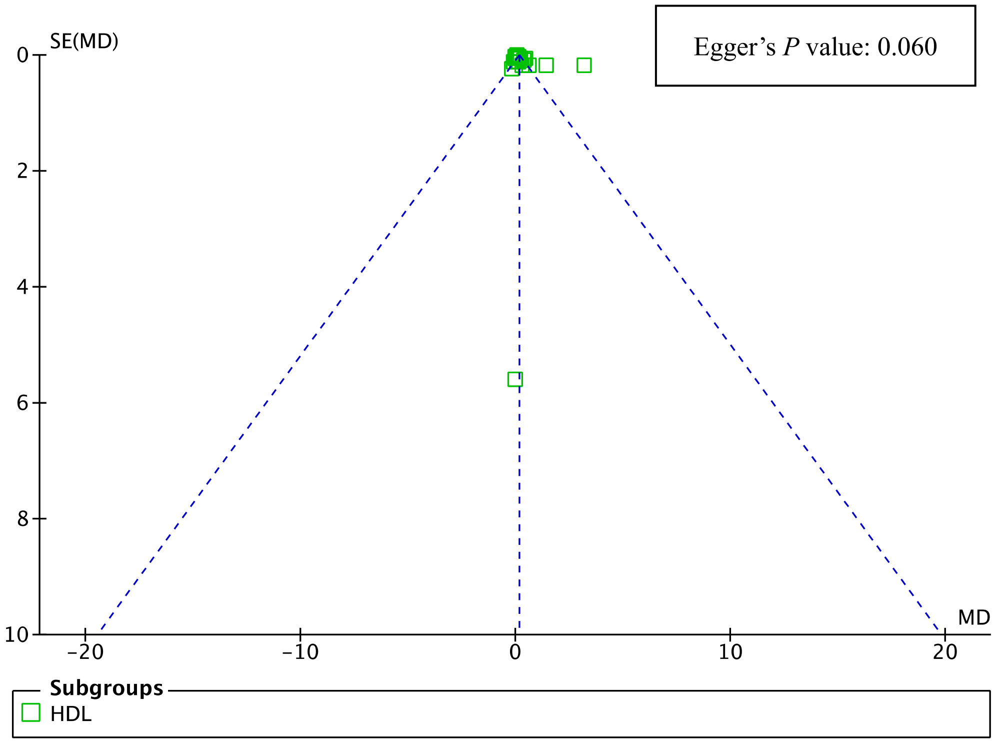

Figure S3-4-3 Funnel plot of RCTs investigating the effect of curcumin supplementation on HDL-C.

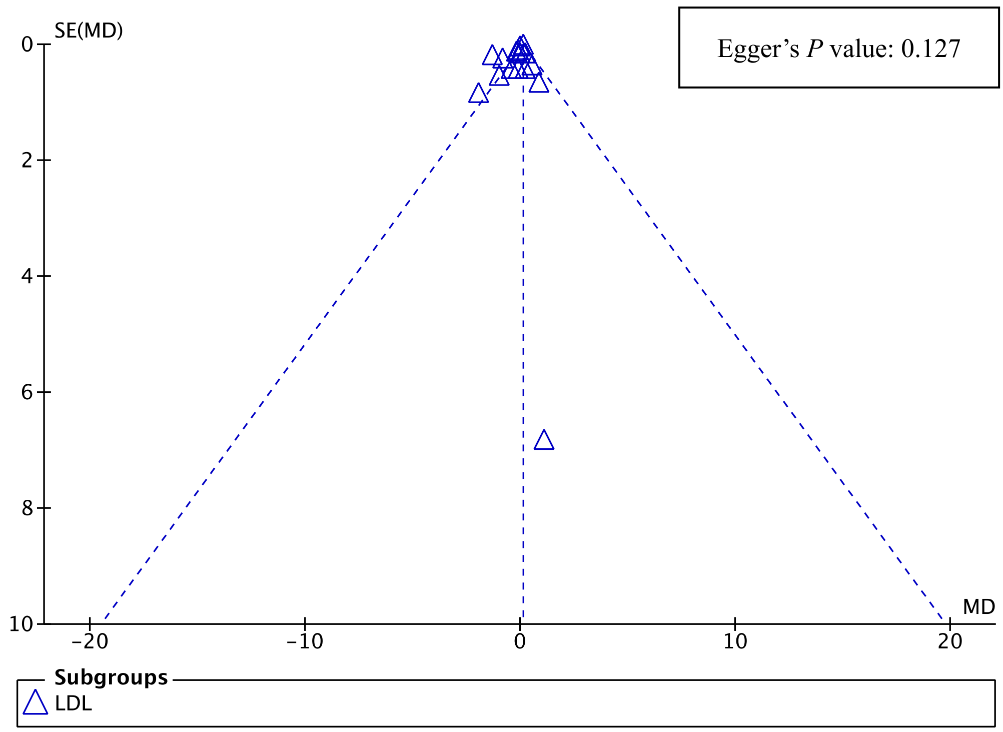

Figure S3-4-4 Funnel plot of RCTs investigating the effect of curcumin supplementation on LDL-C.

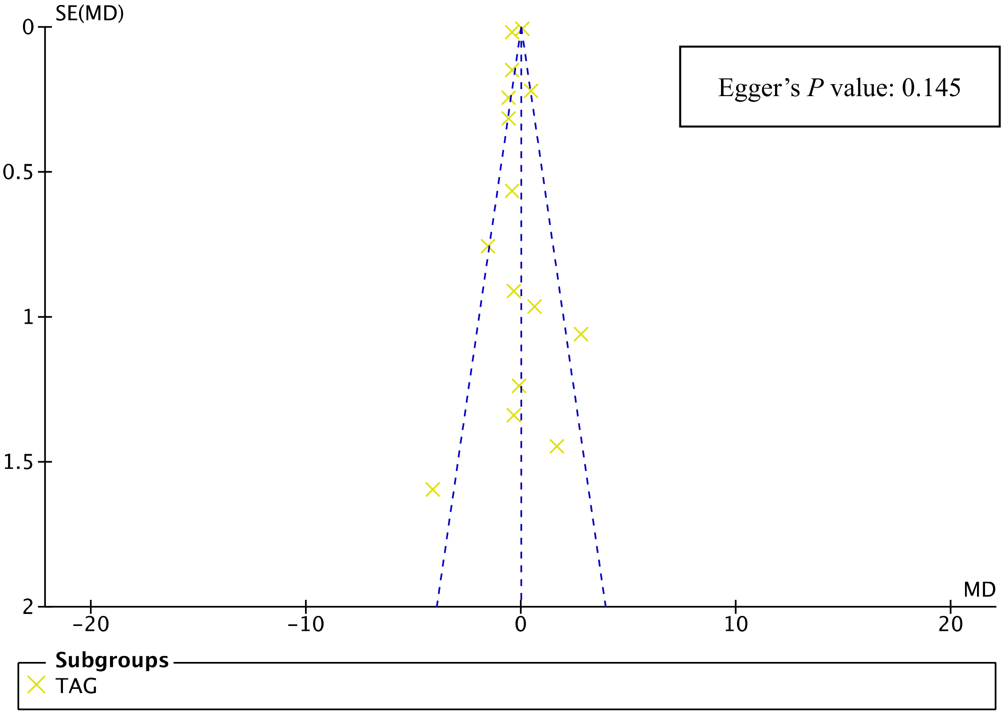

Figure S3-4-5 Funnel plot of RCTs investigating the effect of curcumin supplementation on TG

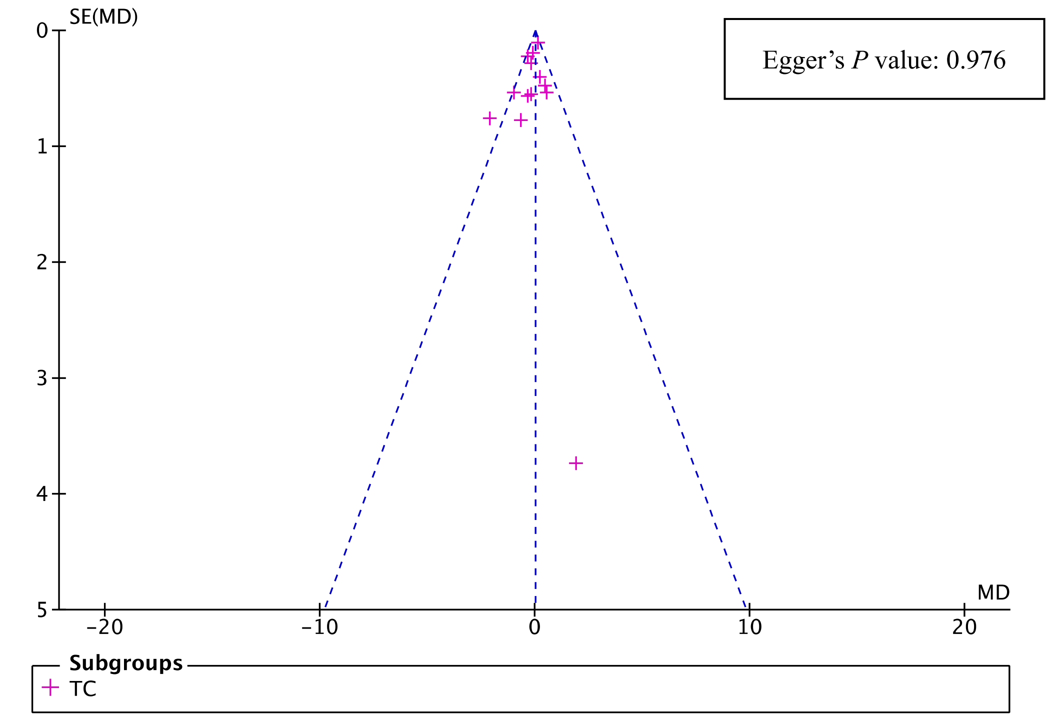

Figure S3-4-6 Funnel plot of RCTs investigating the effect of curcumin supplementation on TC.

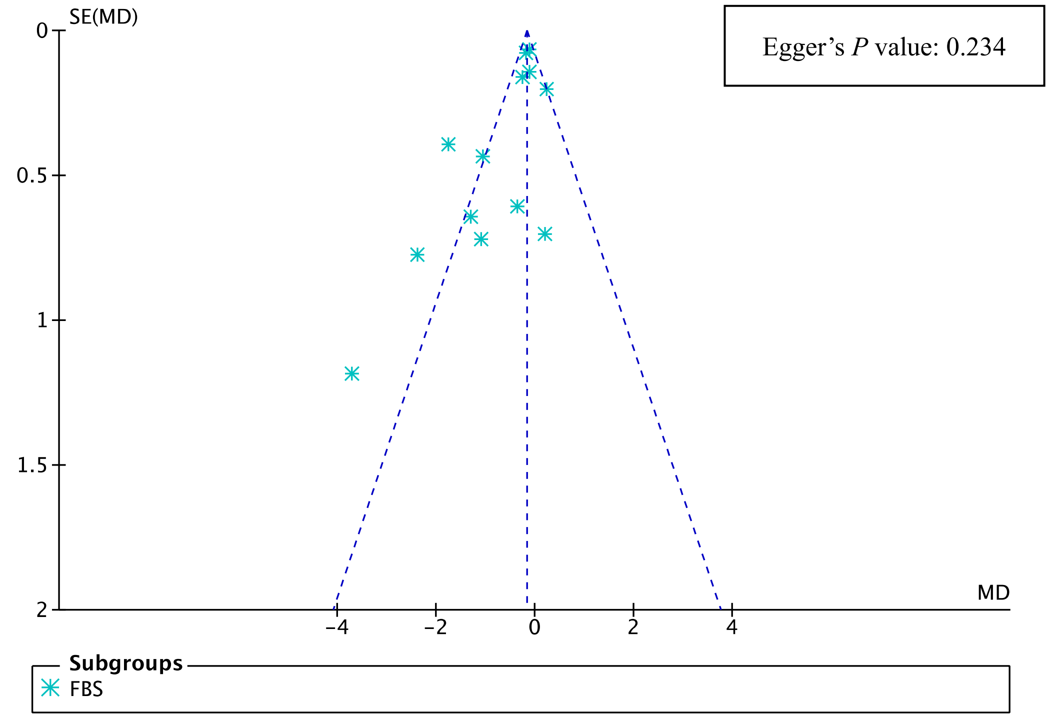

Figure S3-4-7 Funnel plot of RCTs investigating the effect of curcumin supplementation on FBG.

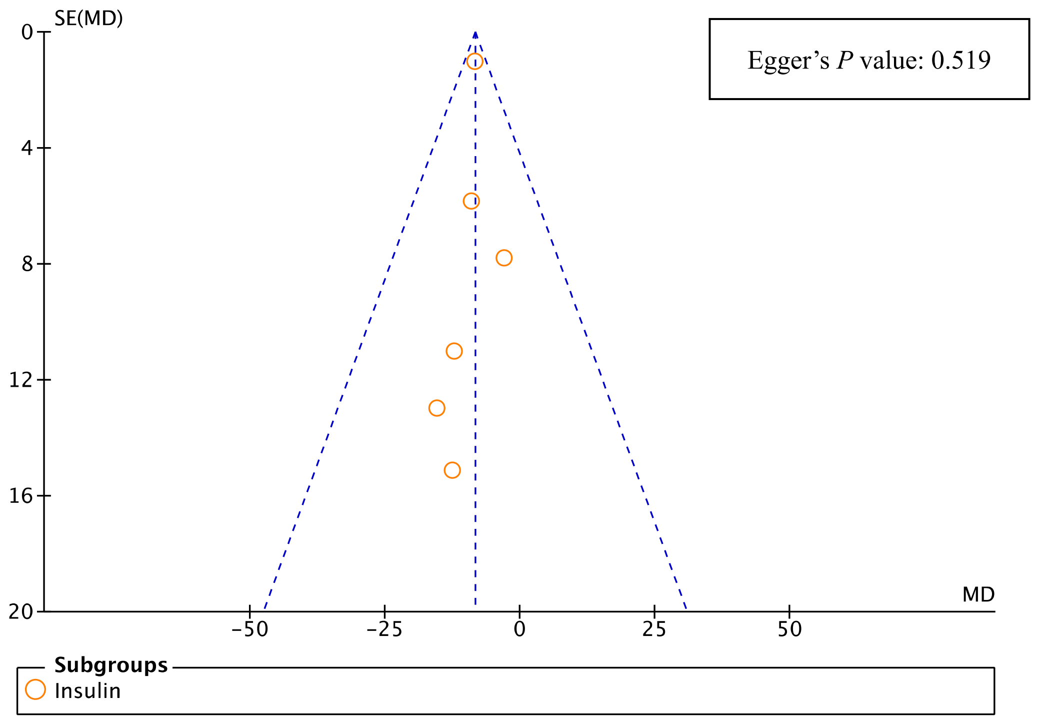

Figure S3-4-8 Funnel plot of RCTs investigating the effect of curcumin supplementation on FBI.

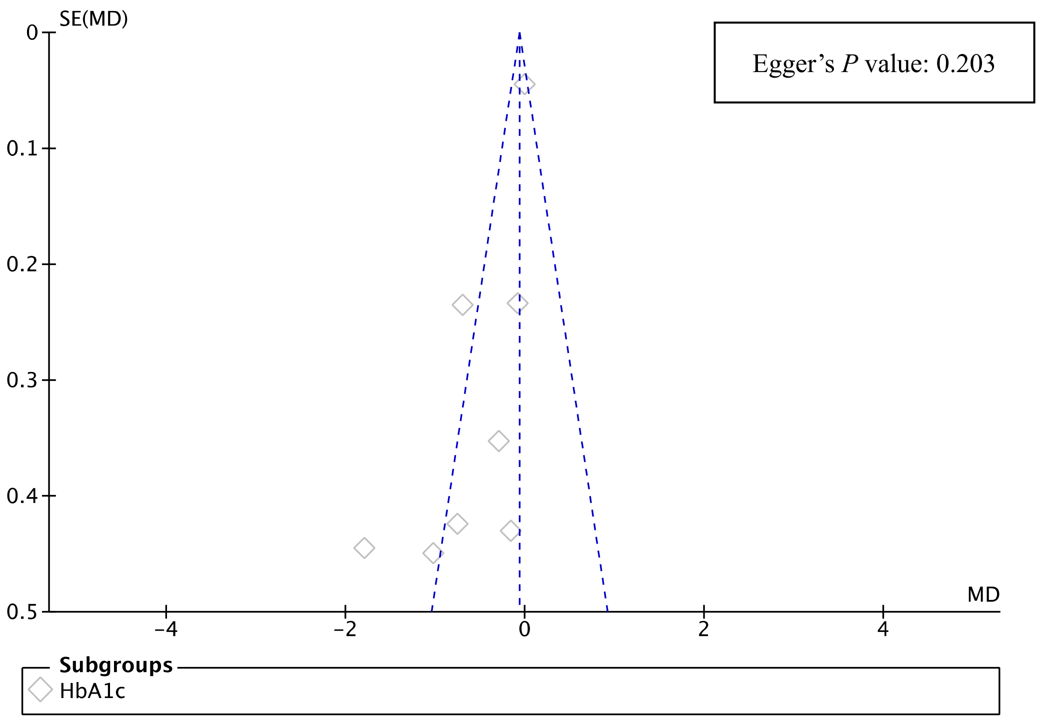

Figure S3-4-9 Funnel plot of RCTs investigating the effect of curcumin supplementation on A1C.

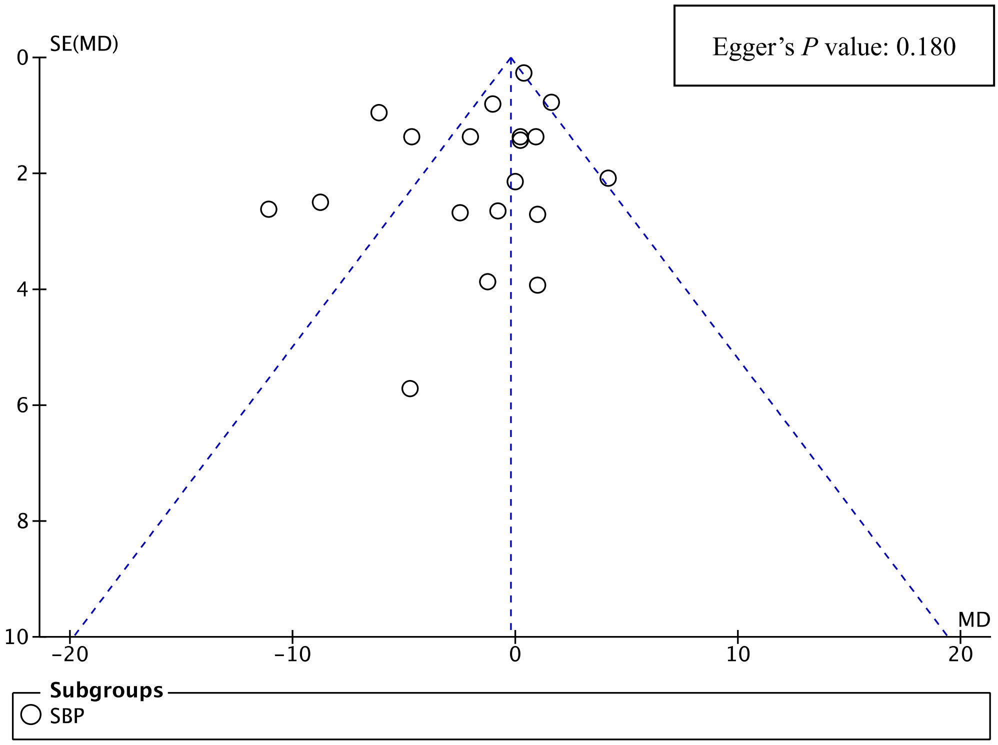

Figure S3-5-1 Funnel plot of RCTs investigating the effect of flavanol supplementation on SBP.

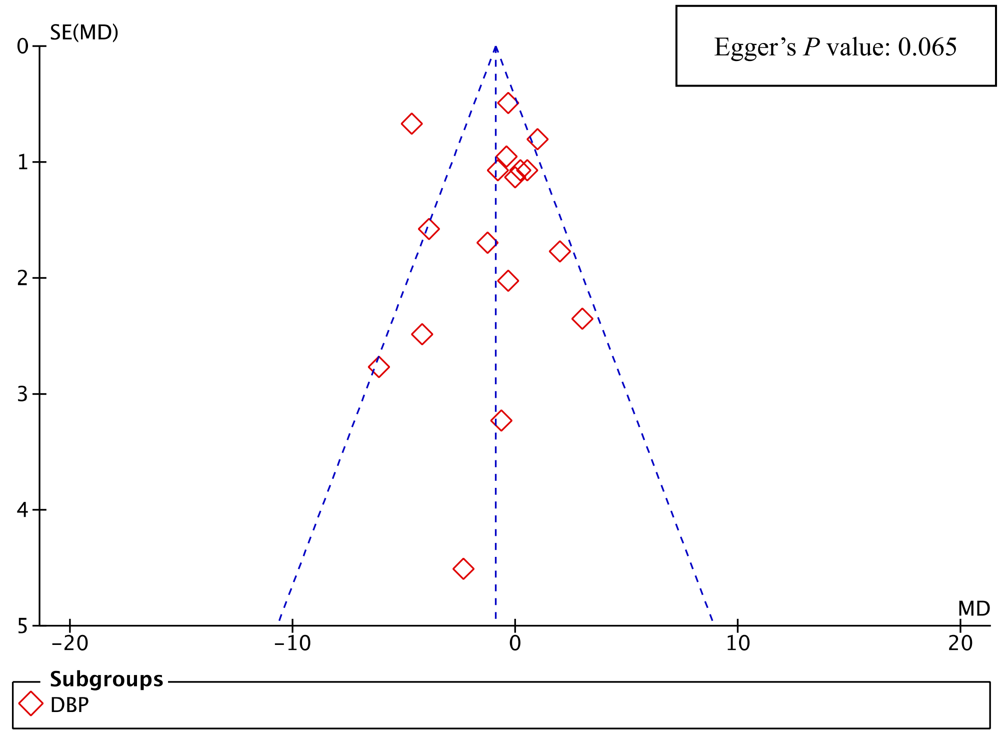

Figure S3-5-2 Funnel plot of RCTs investigating the effect of flavanol supplementation on DBP.

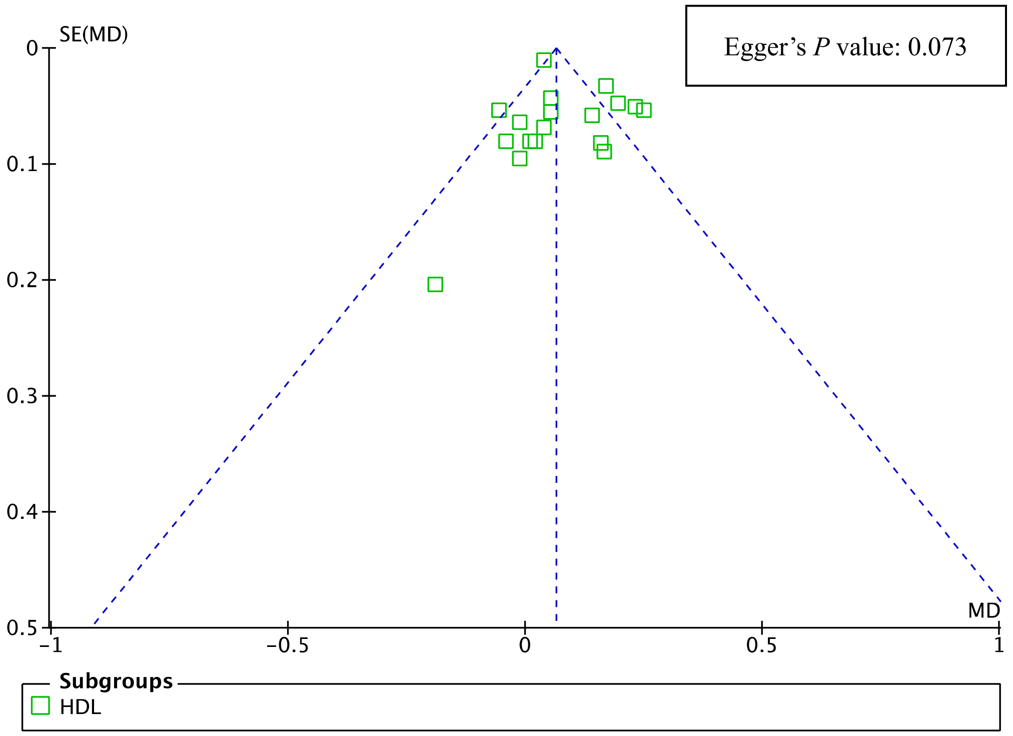

Figure S3-5-3 Funnel plot of RCTs investigating the effect of flavanol supplementation on HDL-C.

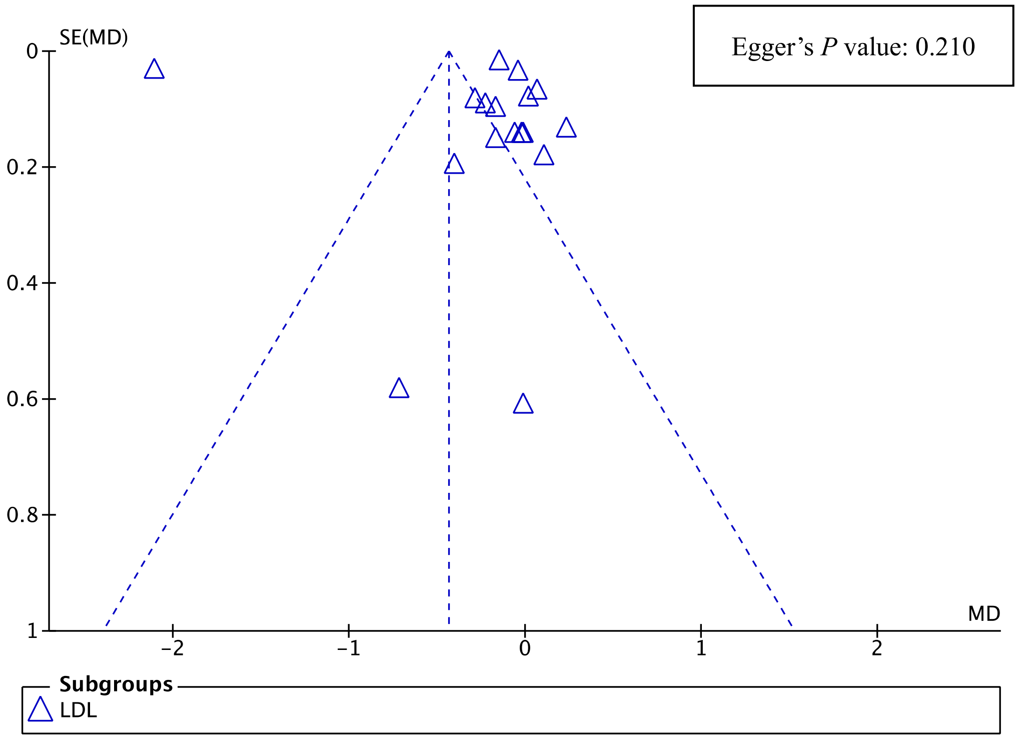

Figure S3-5-4 Funnel plot of RCTs investigating the effect of flavanol supplementation on LDL-C.

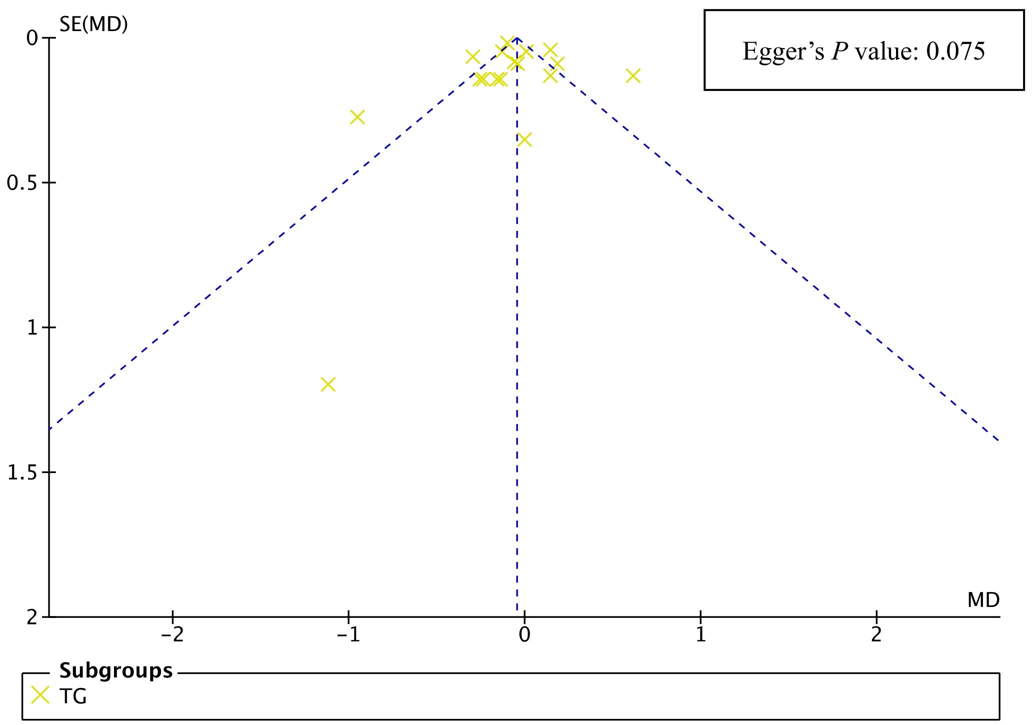

Figure S3-5-5 Funnel plot of RCTs investigating the effect of flavanol supplementation on TG

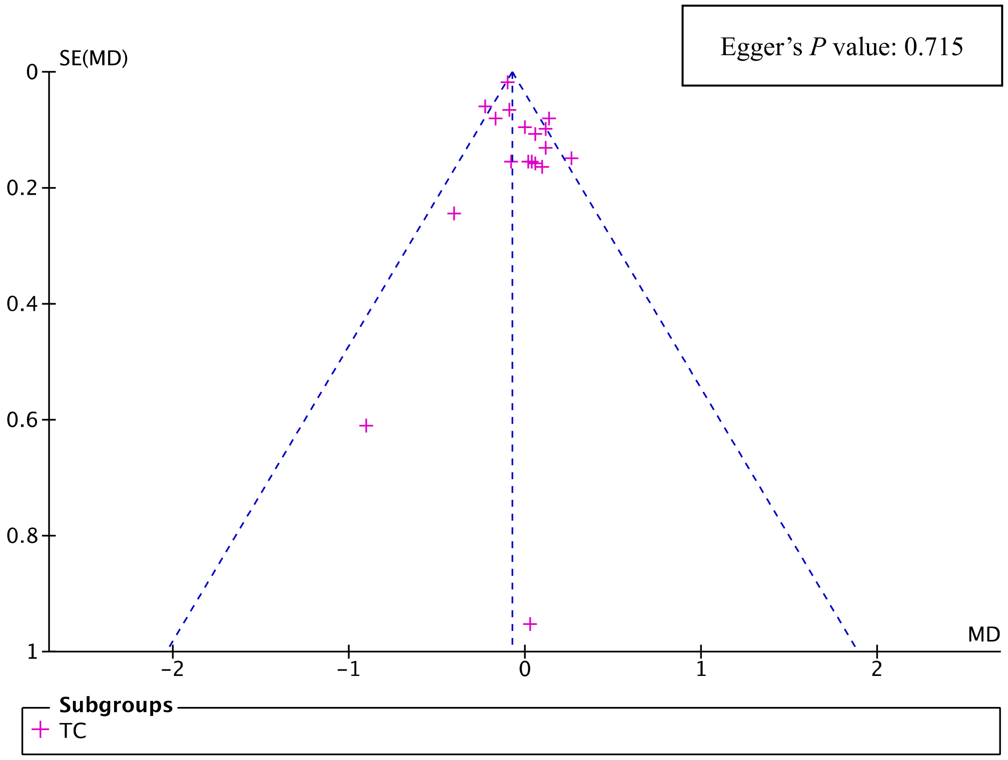

Figure S3-5-6 Funnel plot of RCTs investigating the effect of flavanol supplementation on TC.

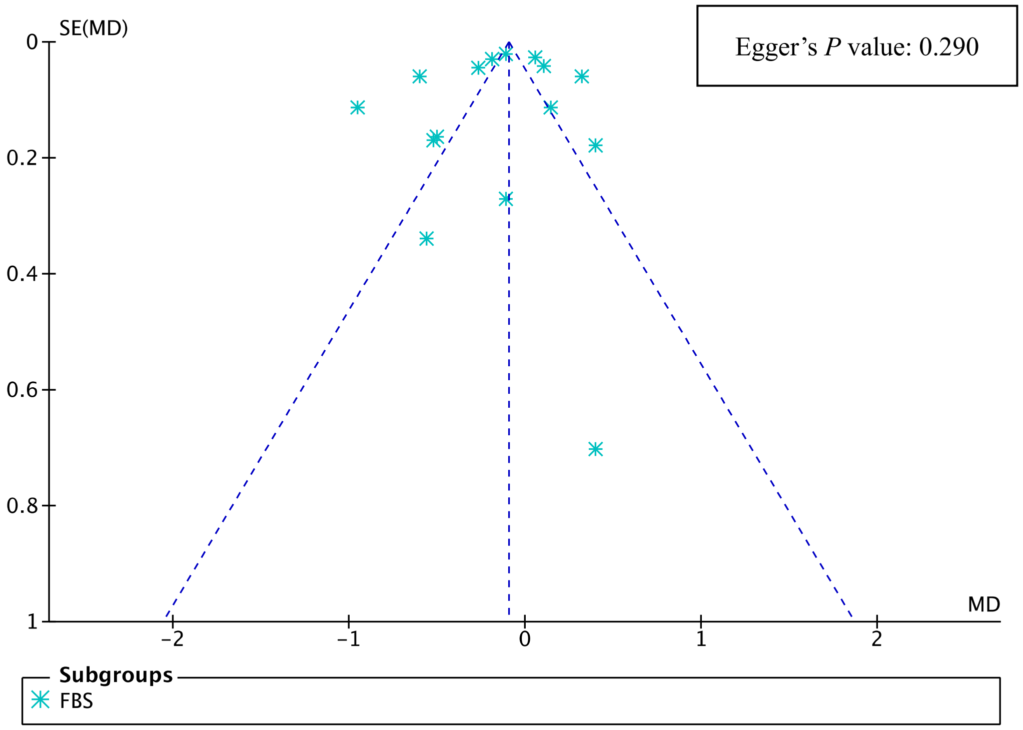

Figure S3-5-7 Funnel plot of RCTs investigating the effect of flavanol supplementation on FBG.

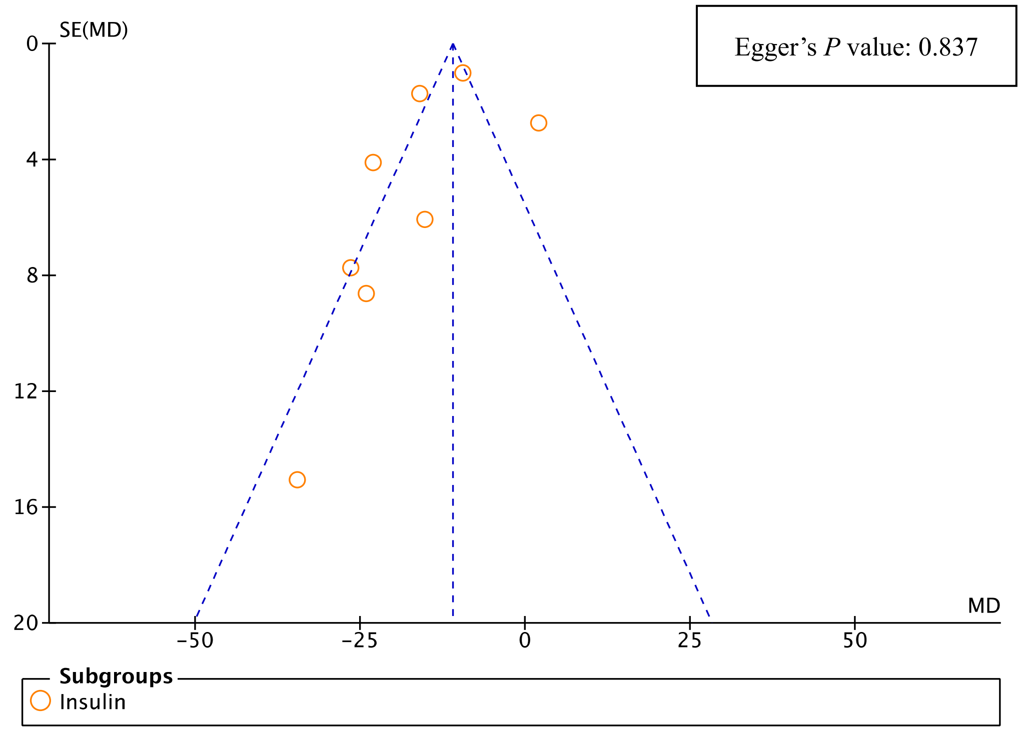

Figure S3-5-8 Funnel plot of RCTs investigating the effect of flavanol supplementation on FBI.

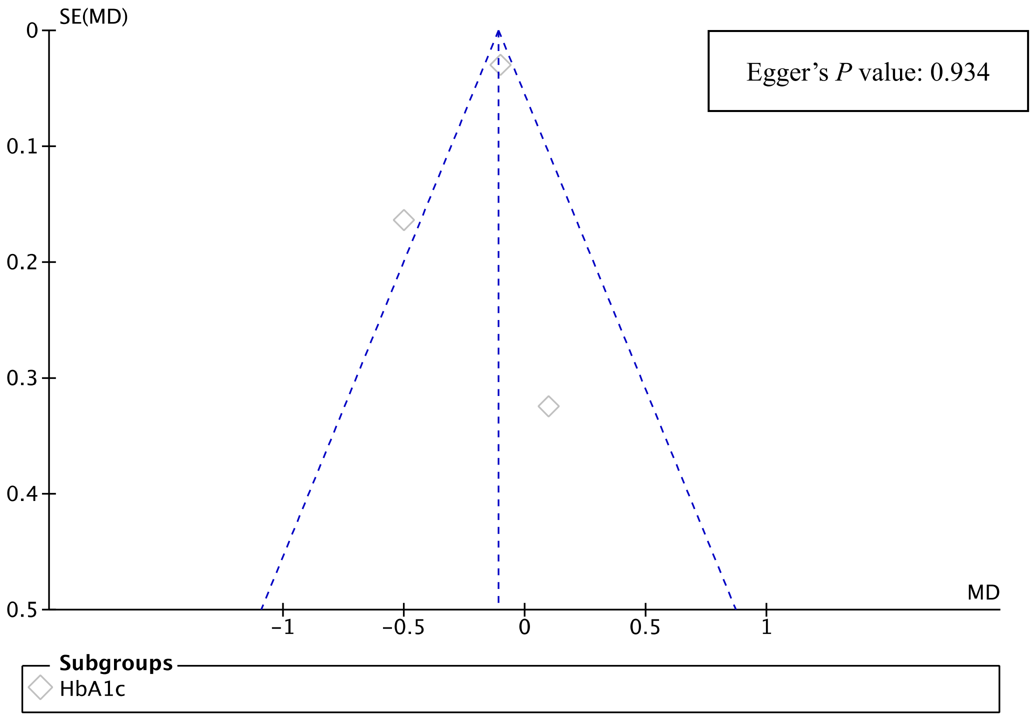

Figure S3-5-9 Funnel plot of RCTs investigating the effect of flavanol supplementation on A1C.

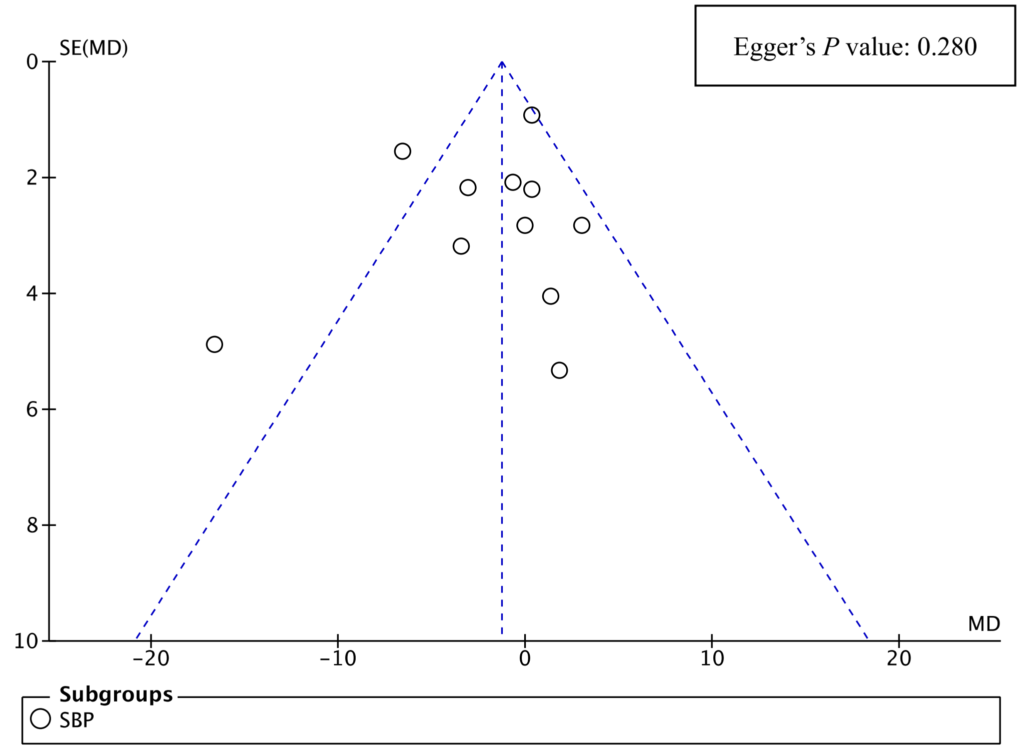

Figure S3-6-1 Funnel plot of RCTs investigating the effect of flavonoid supplementation on SBP.

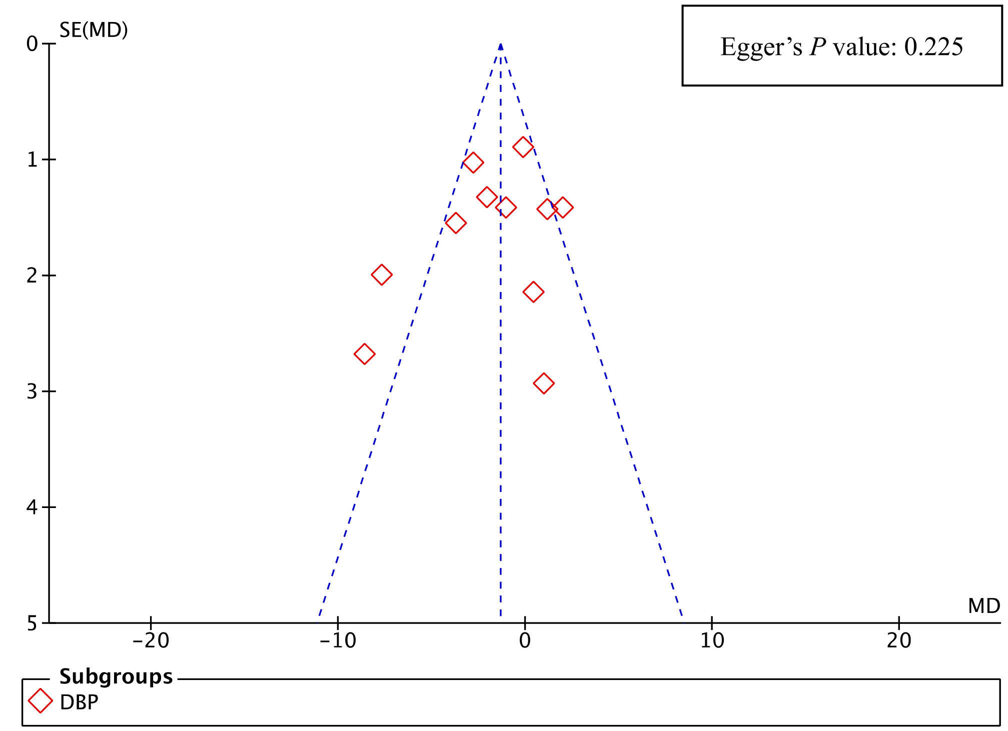

Figure S3-6-2 Funnel plot of RCTs investigating the effect of flavonoid supplementation on DBP.

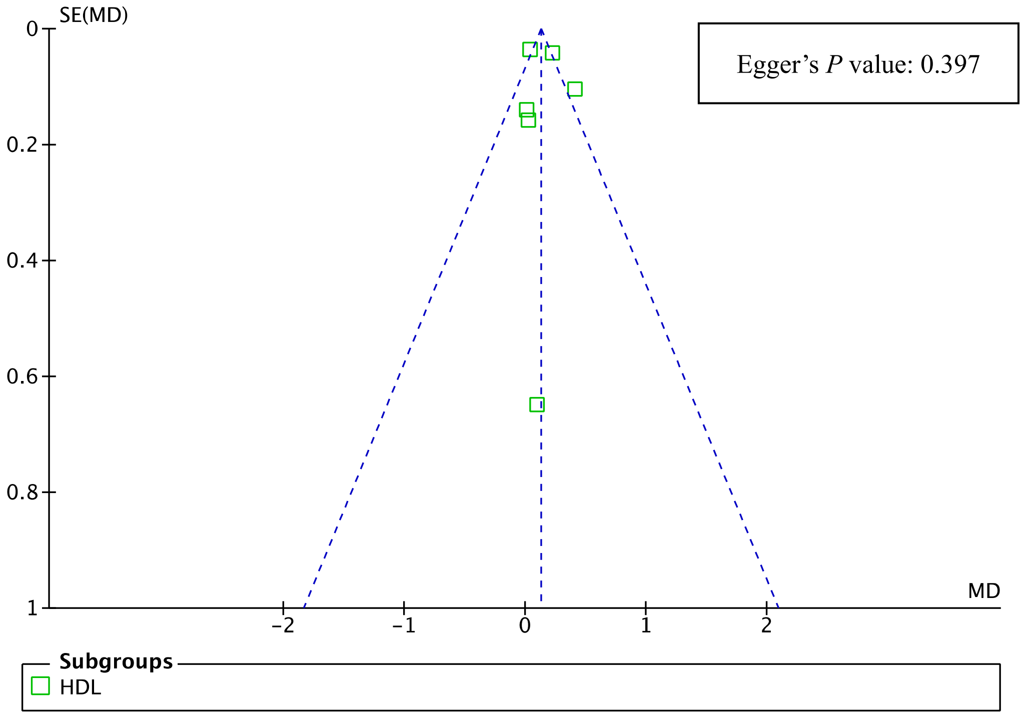

Figure S3-6-3 Funnel plot of RCTs investigating the effect of flavonoid supplementation on HDL-C.

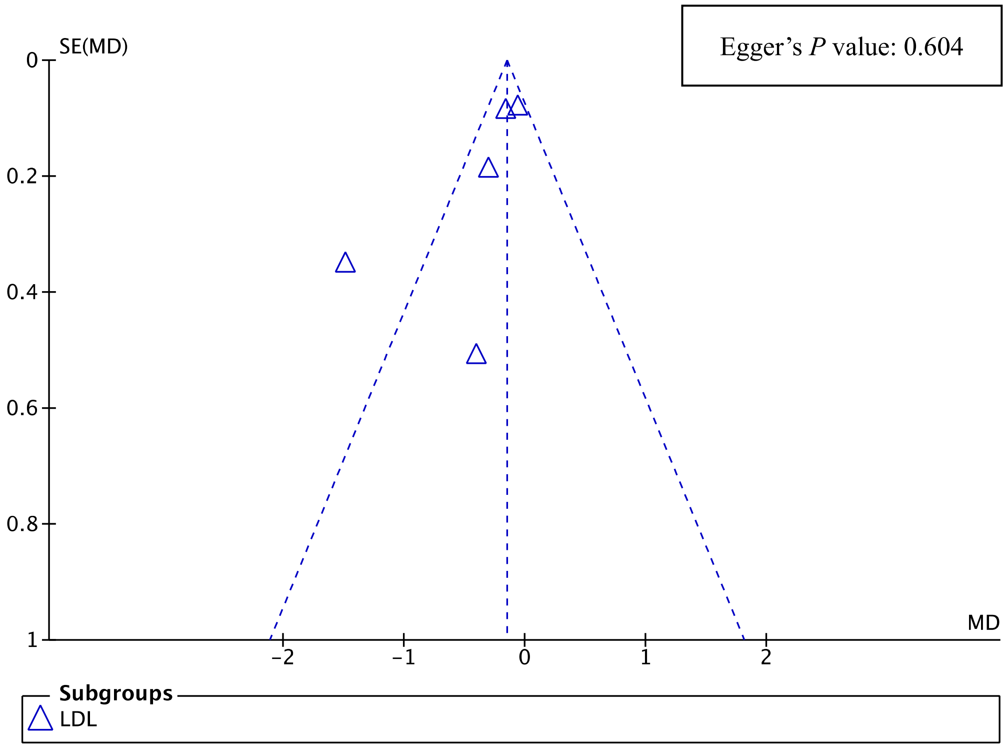

Figure S3-6-4 Funnel plot of RCTs investigating the effect of flavonoid supplementation on LDL-C.

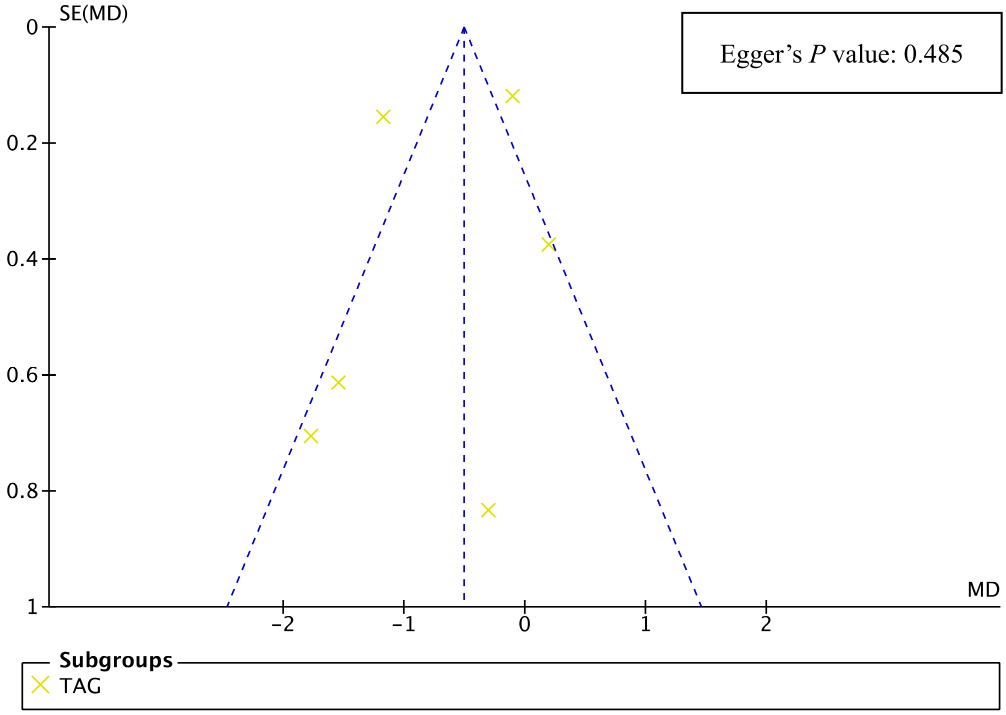

Figure S3-6-5 Funnel plot of RCTs investigating the effect of flavonoid supplementation on TG

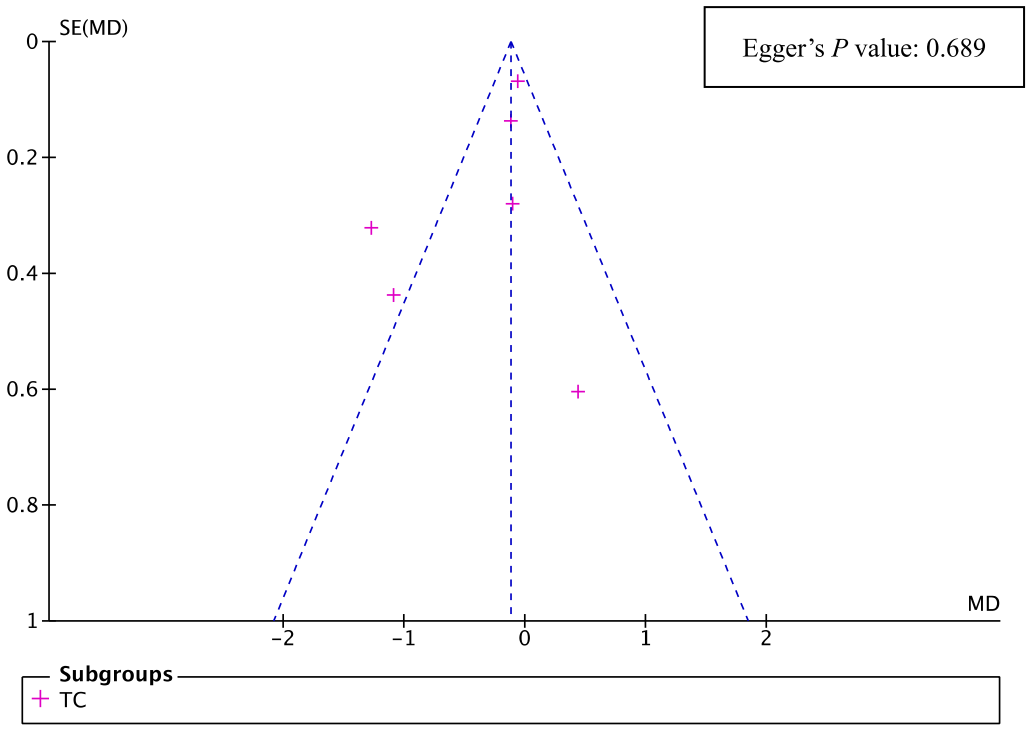

Figure S3-6-6 Funnel plot of RCTs investigating the effect of flavonoid supplementation on TC.

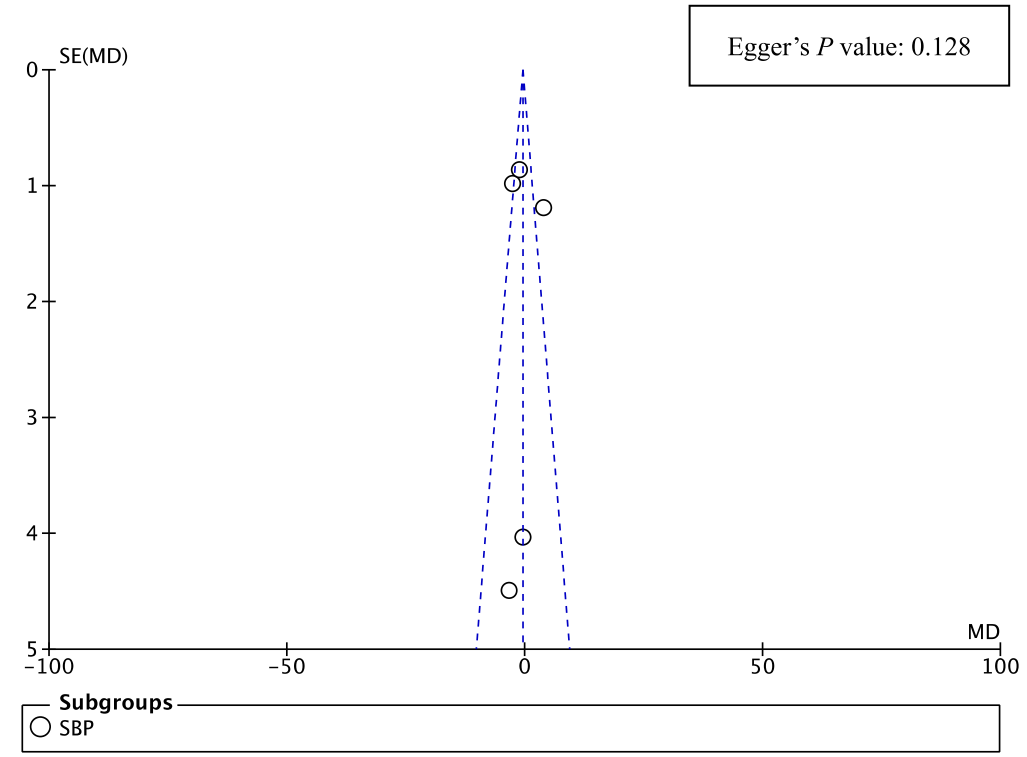

Figure S3-7-1 Funnel plot of RCTs investigating the effect of gallic acid supplementation on SBP.

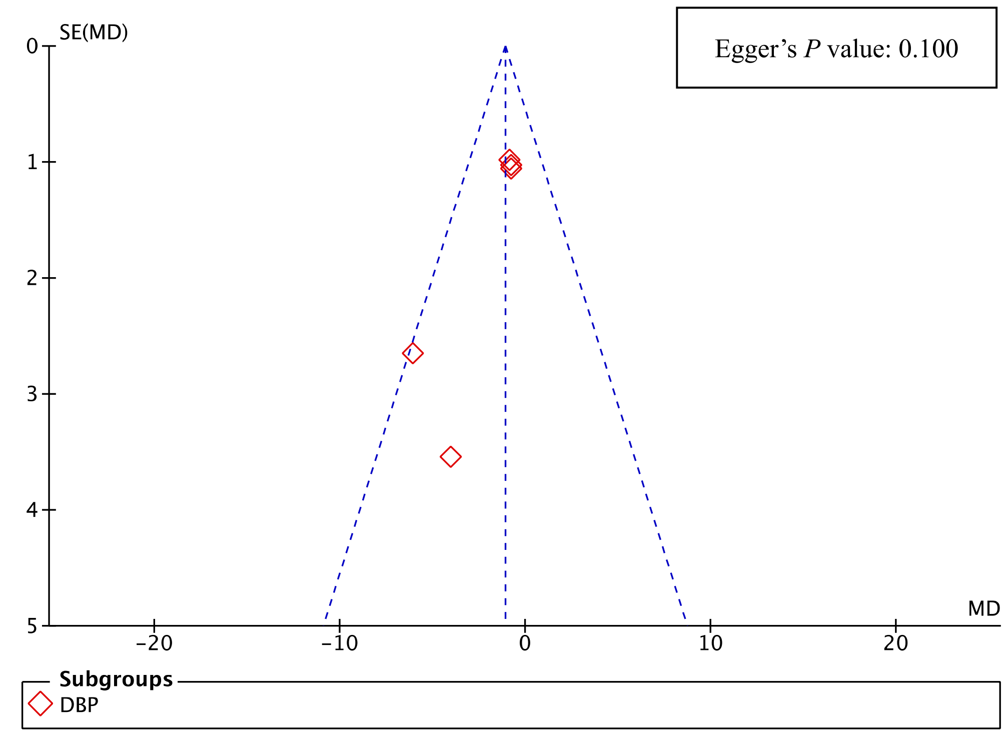

Figure S3-7-2 Funnel plot of RCTs investigating the effect of gallic acid supplementation on DBP.

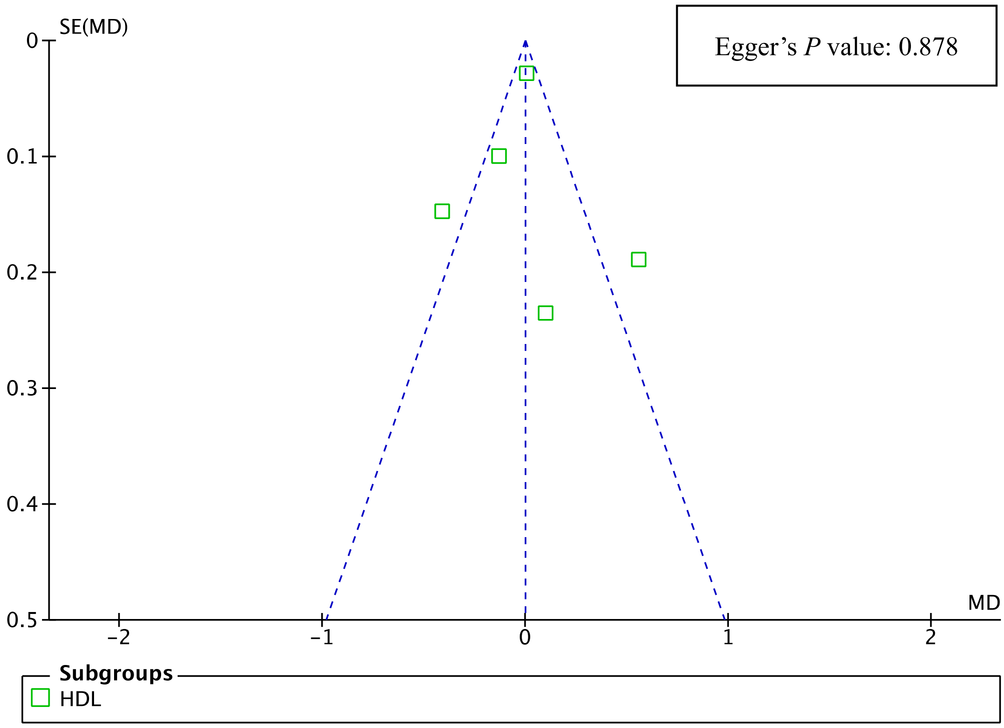

Figure S3-7-3 Funnel plot of RCTs investigating the effect of gallic acid supplementation on HDL.

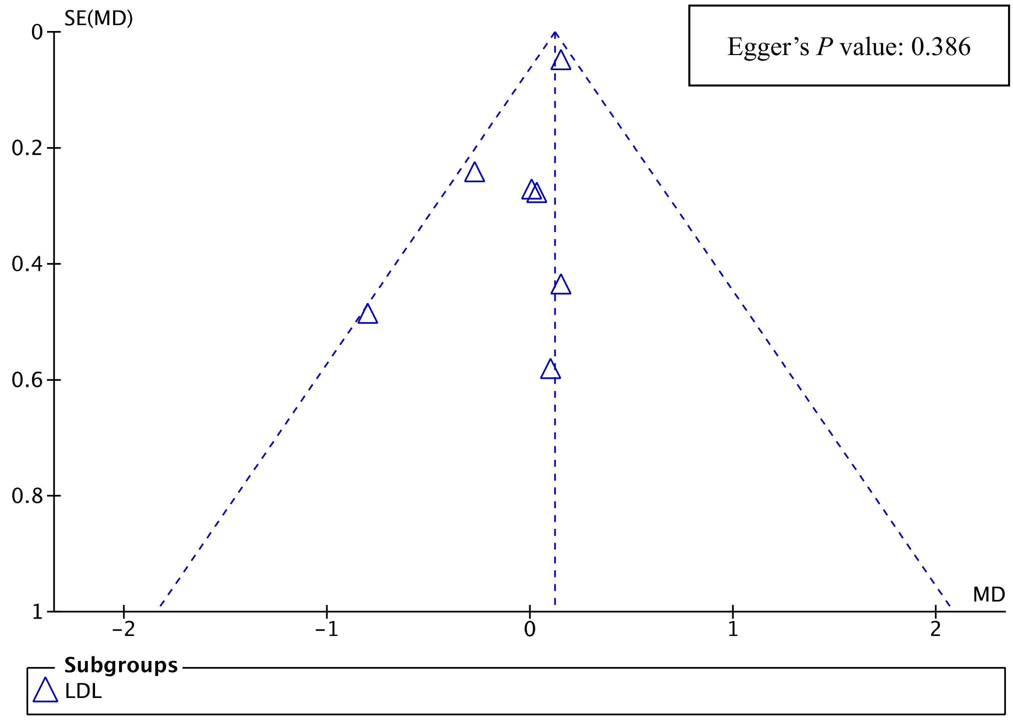

Figure S3-7-4 Funnel plot of RCTs investigating the effect of gallic acid supplementation on LDL.

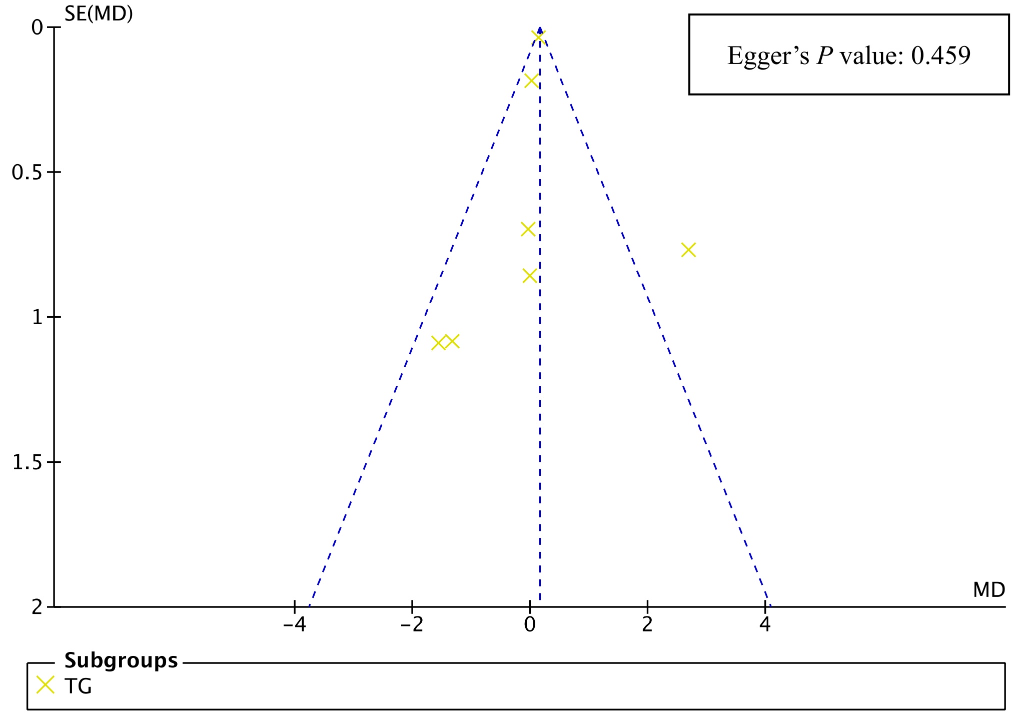

Figure S3-7-5 Funnel plot of RCTs investigating the effect of gallic acid supplementation on TG.

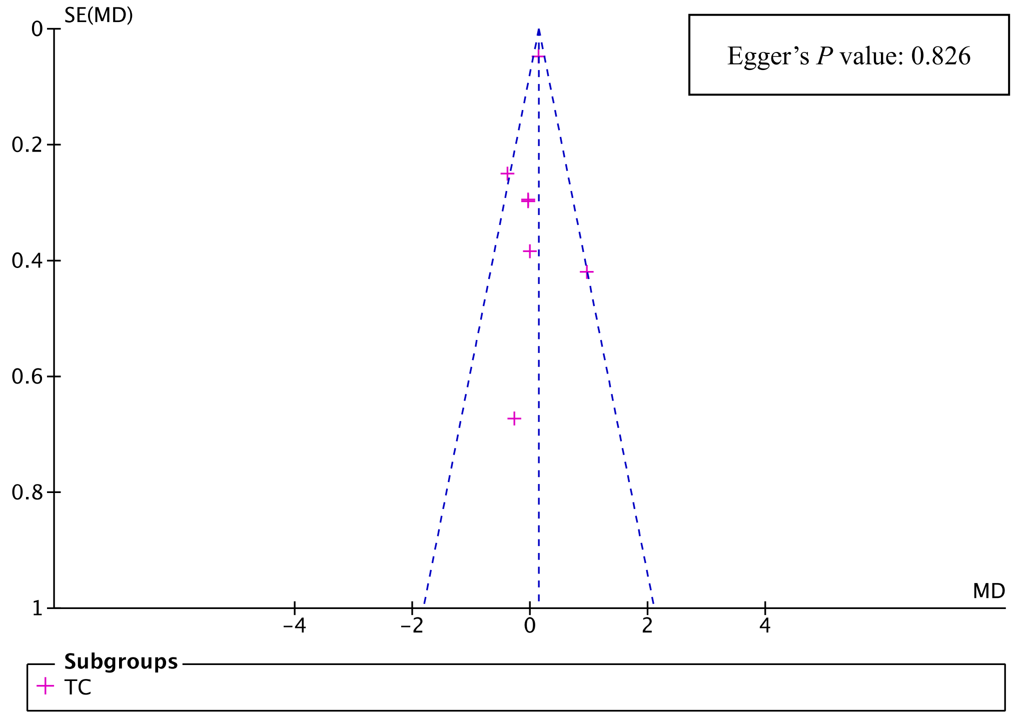

Figure S3-7-6 Funnel plot of RCTs investigating the effect of gallic aciid supplementation on TC.

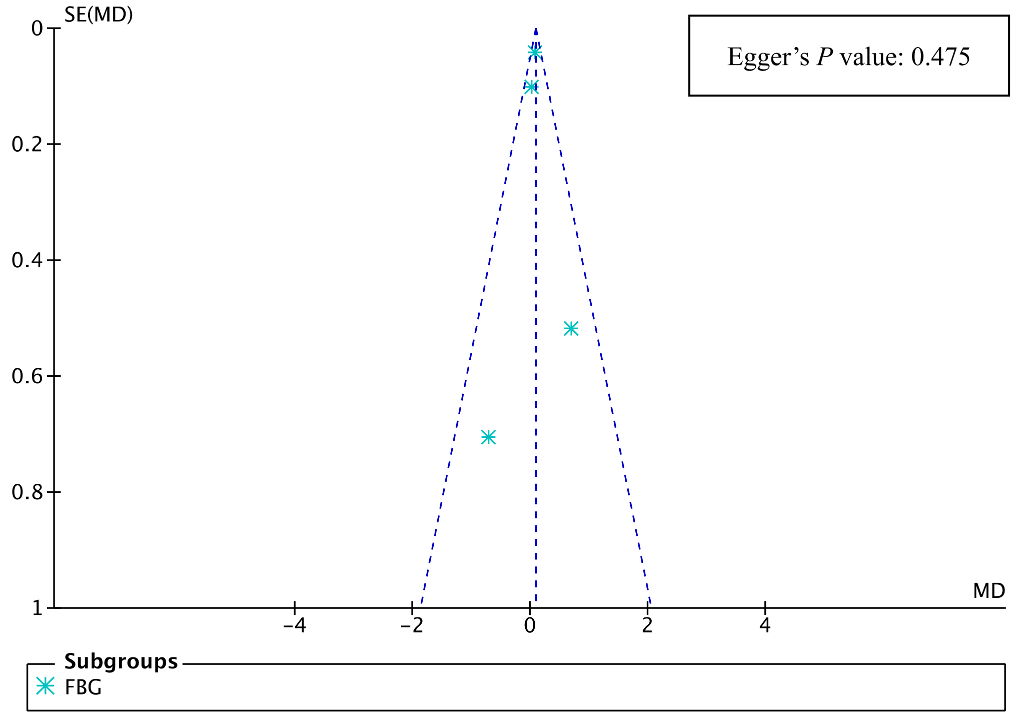

Figure S3-7-7 Funnel plot of RCTs investigating the effect of gallic acids supplementation on FBG.

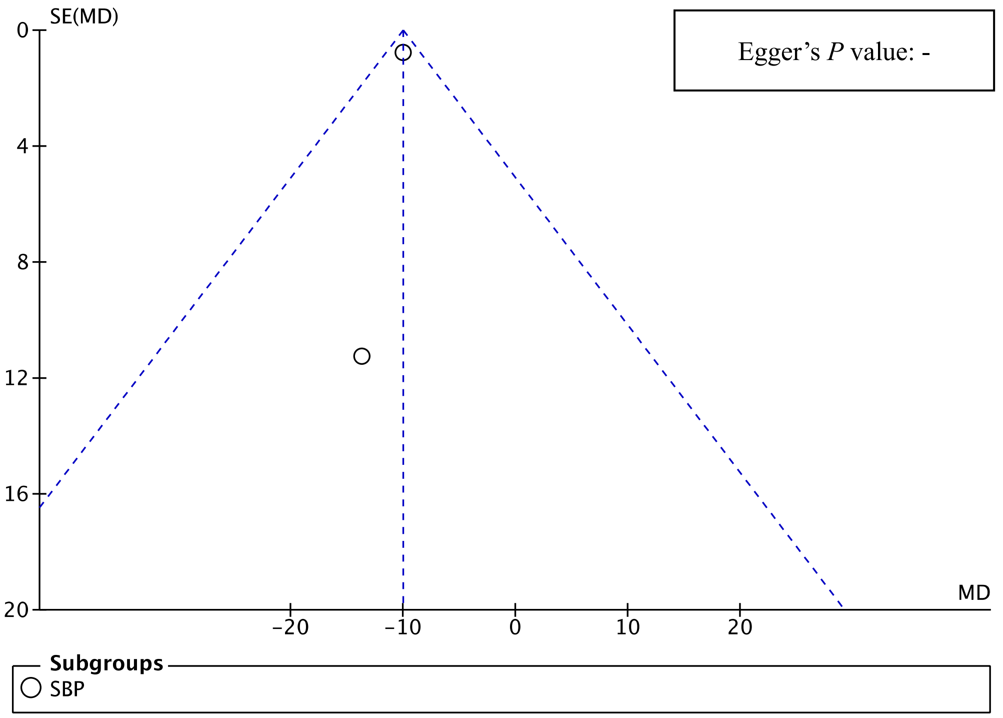

Figure S3-8-1 Funnel plot of RCTs investigating the effect of genistein supplementation on SBP.

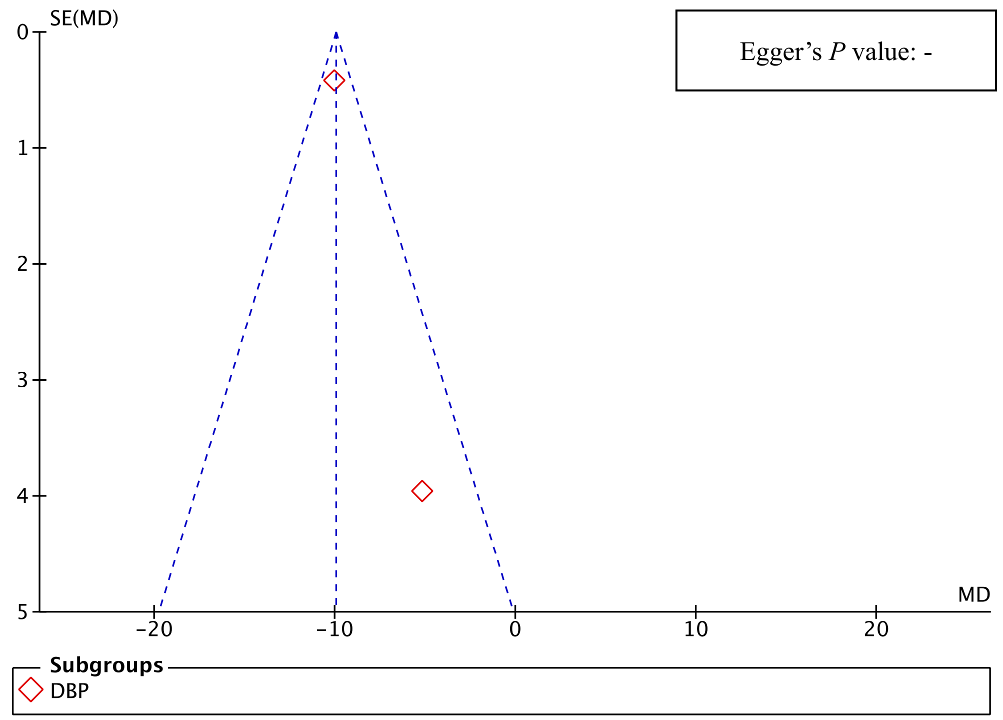

Figure S3-8-2 Funnel plot of RCTs investigating the effect of genistein supplementation on DBP.

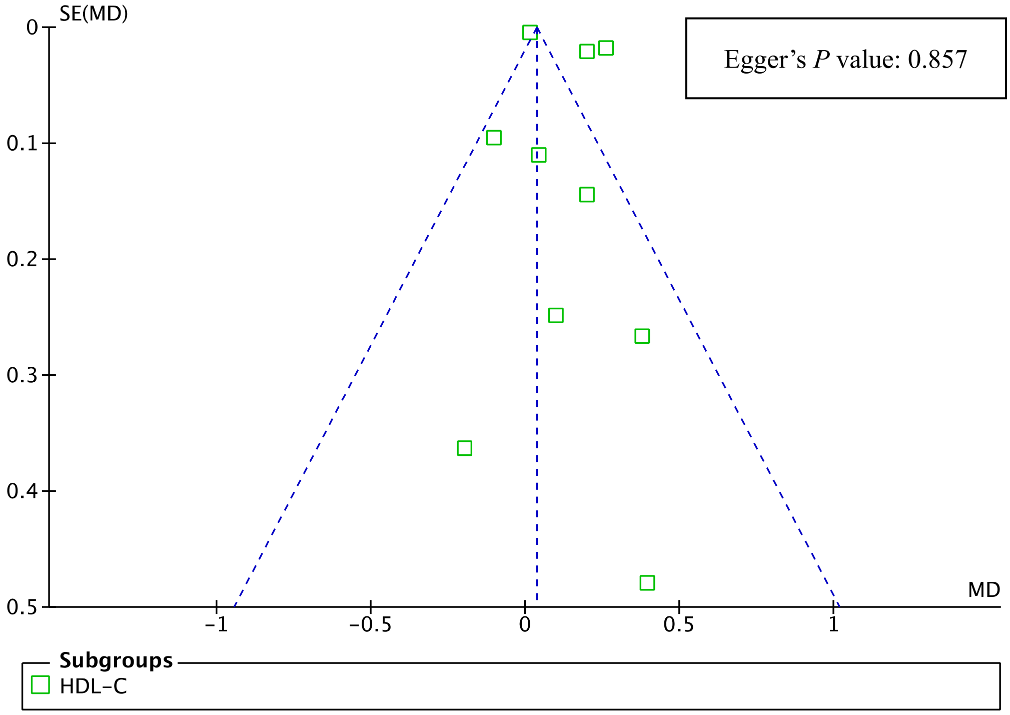

Figure S3-8-3 Funnel plot of RCTs investigating the effect of genistein supplementation on HDL.

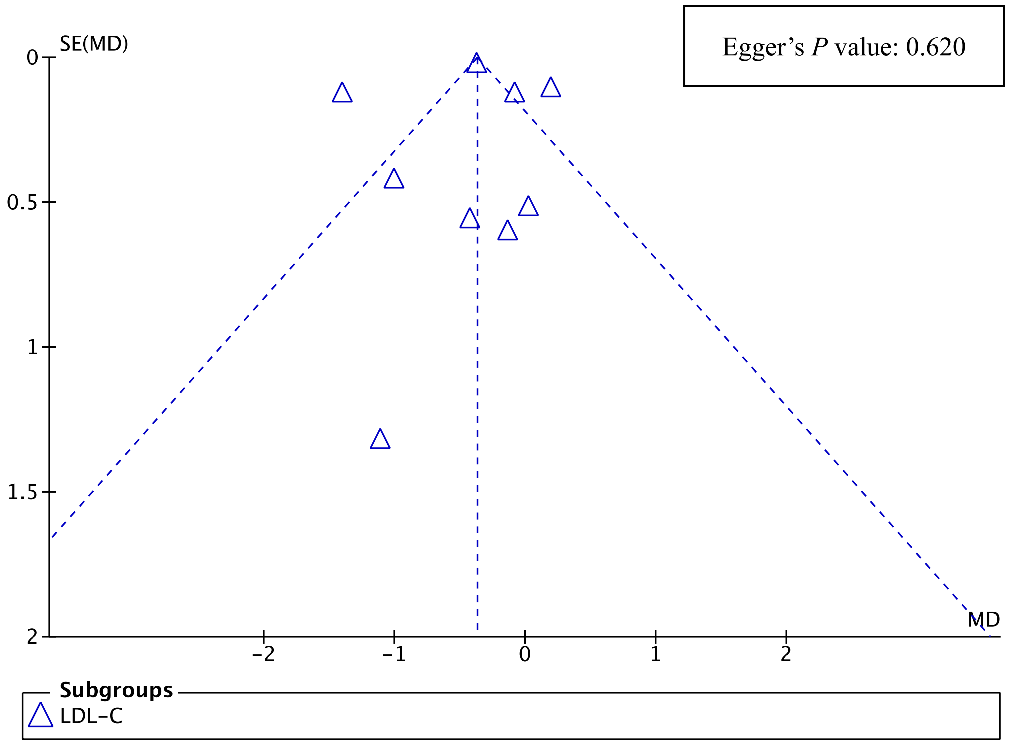

Figure S3-8-4 Funnel plot of RCTs investigating the effect of genistein supplementation on LDL.

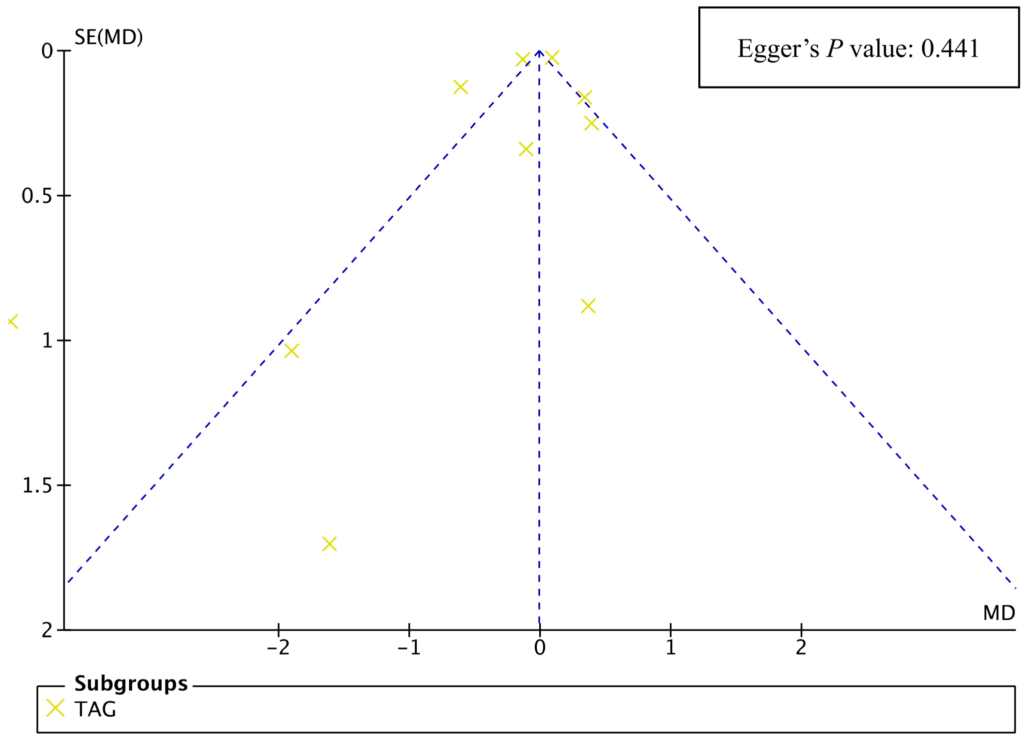

Figure S3-8-5 Funnel plot of RCTs investigating the effect of genistein supplementation on TG.

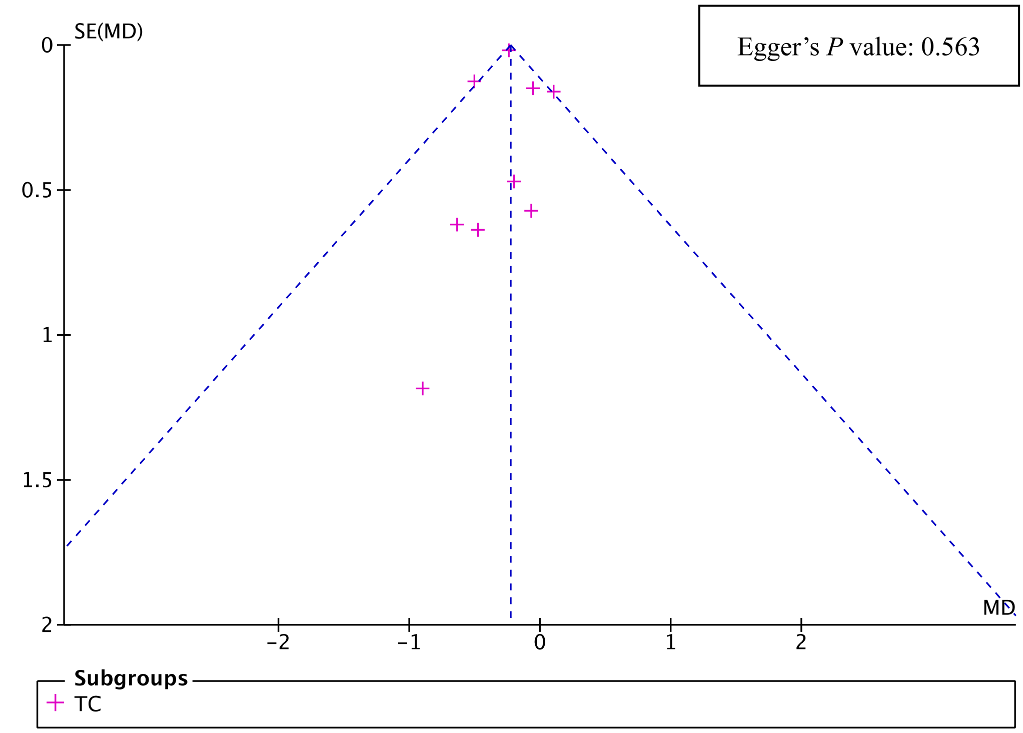

Figure S3-8-6 Funnel plot of RCTs investigating the effect of genistein supplementation on TC.

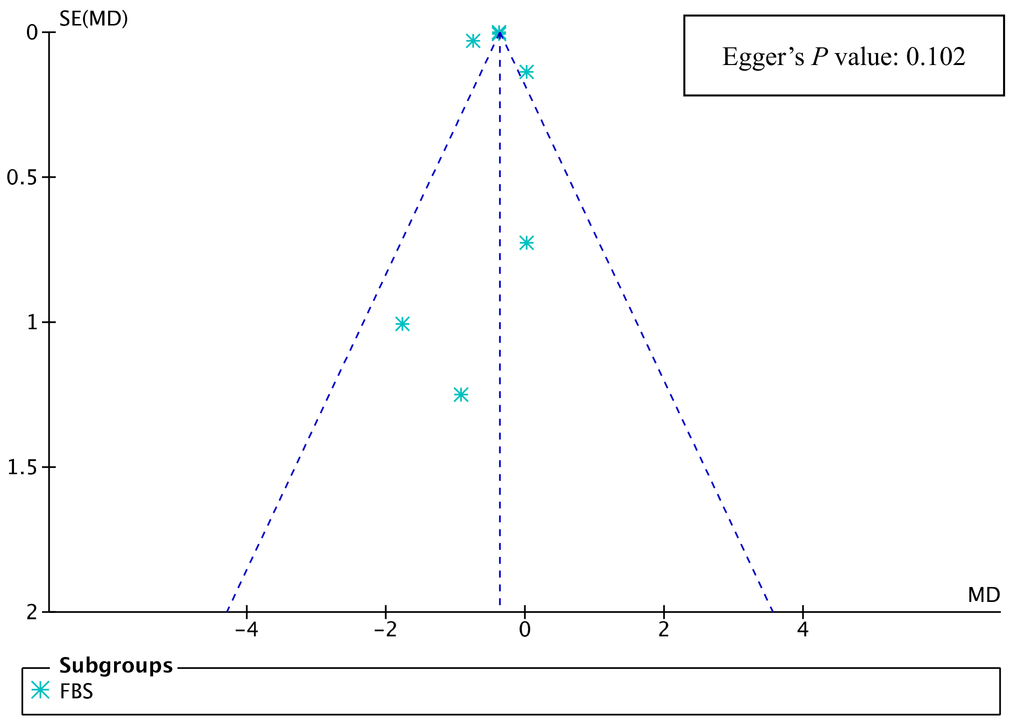

Figure S3-8-7 Funnel plot of RCTs investigating the effect of genistein supplementation on FBG.

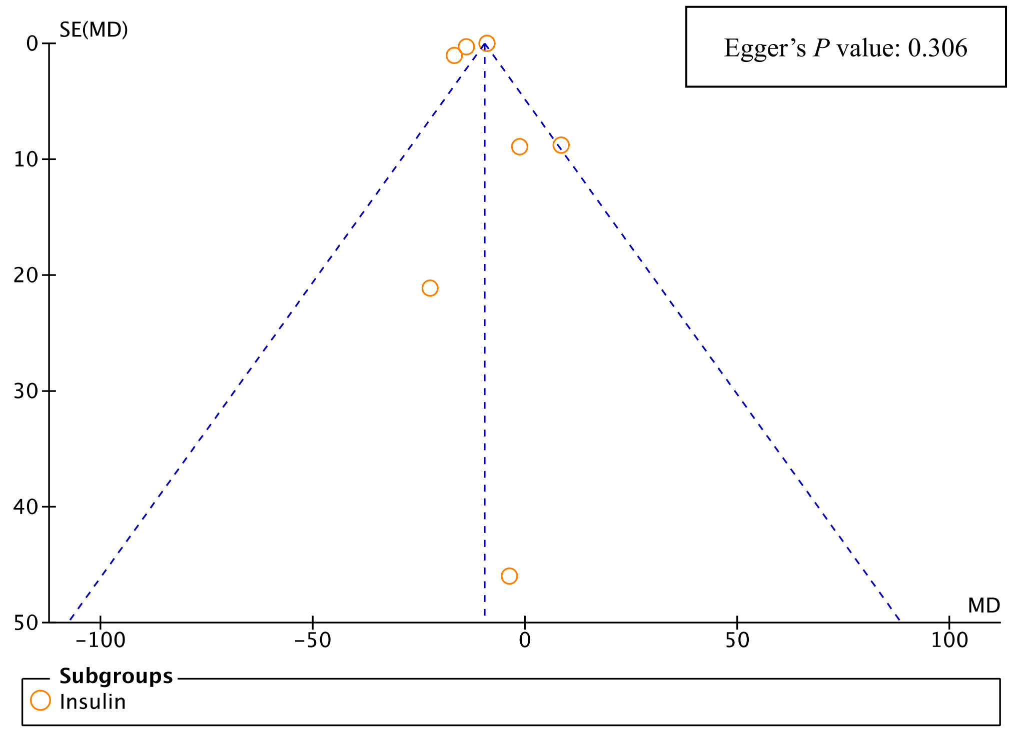

Figure S3-8-8 Funnel plot of RCTs investigating the effect of genistein supplementation on FBI.

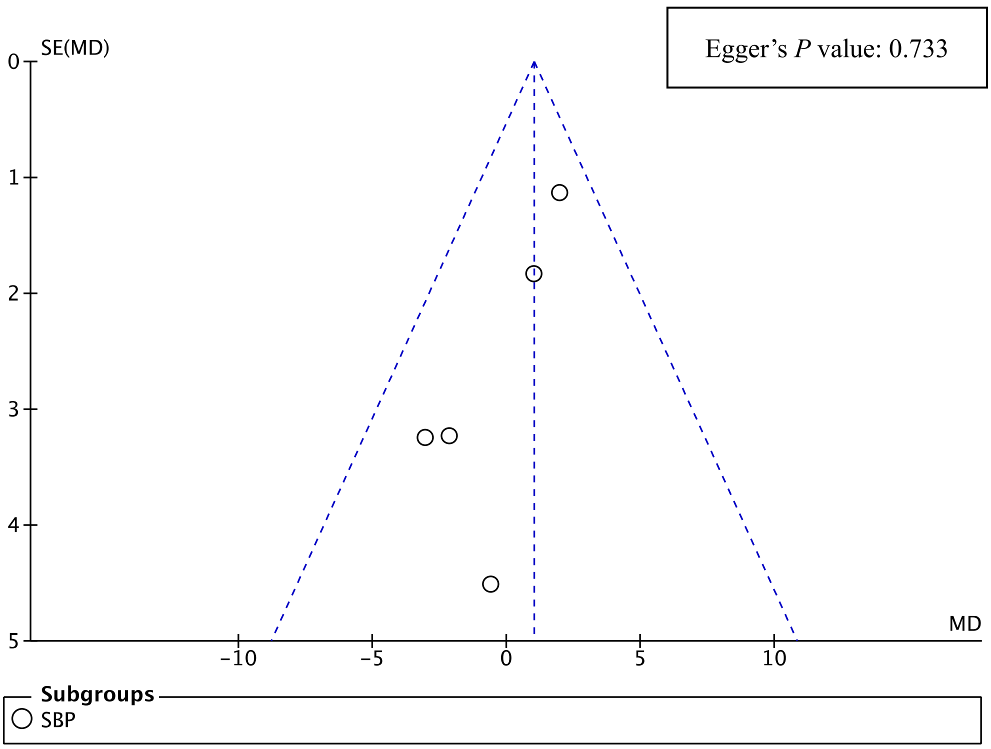

Figure S3-9-1 Funnel plot of RCTs investigating the effect of hesperidin supplementation on SBP.

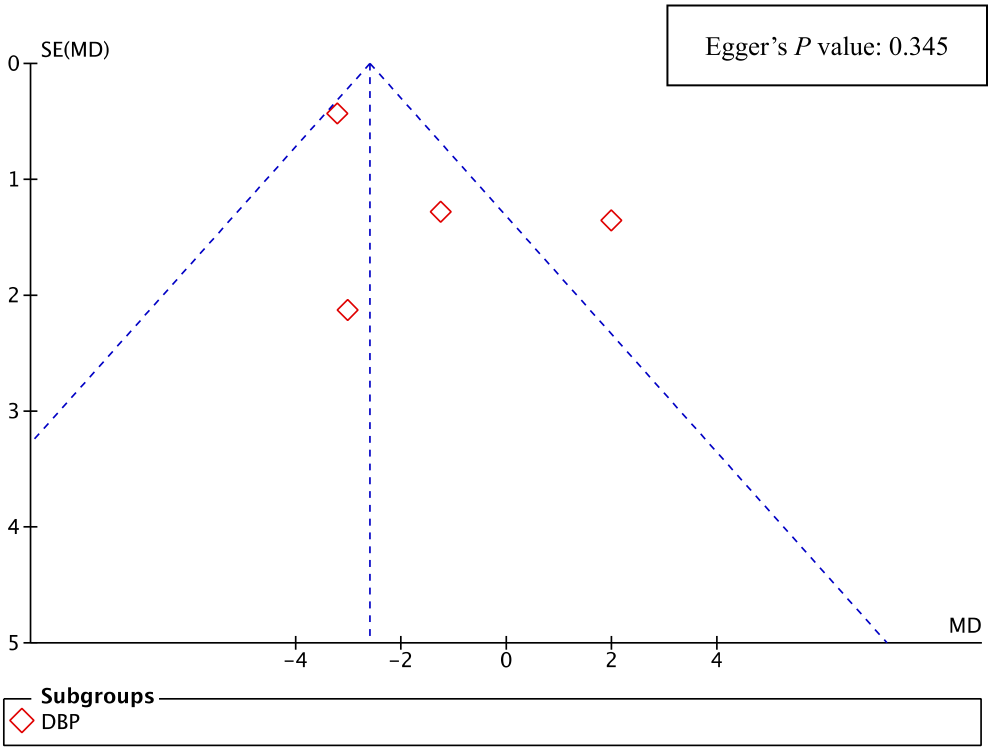

Figure S3-9-2 Funnel plot of RCTs investigating the effect of hesperidin supplementation on DBP.

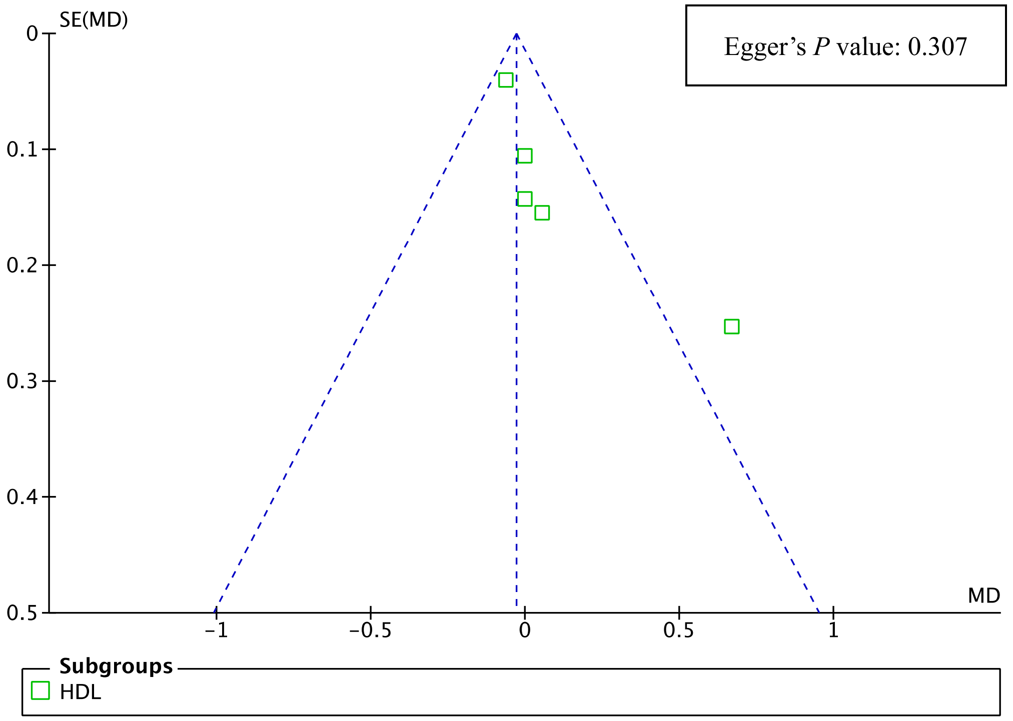

Figure S3-9-3 Funnel plot of RCTs investigating the effect of hesperidin supplementation on HDL.

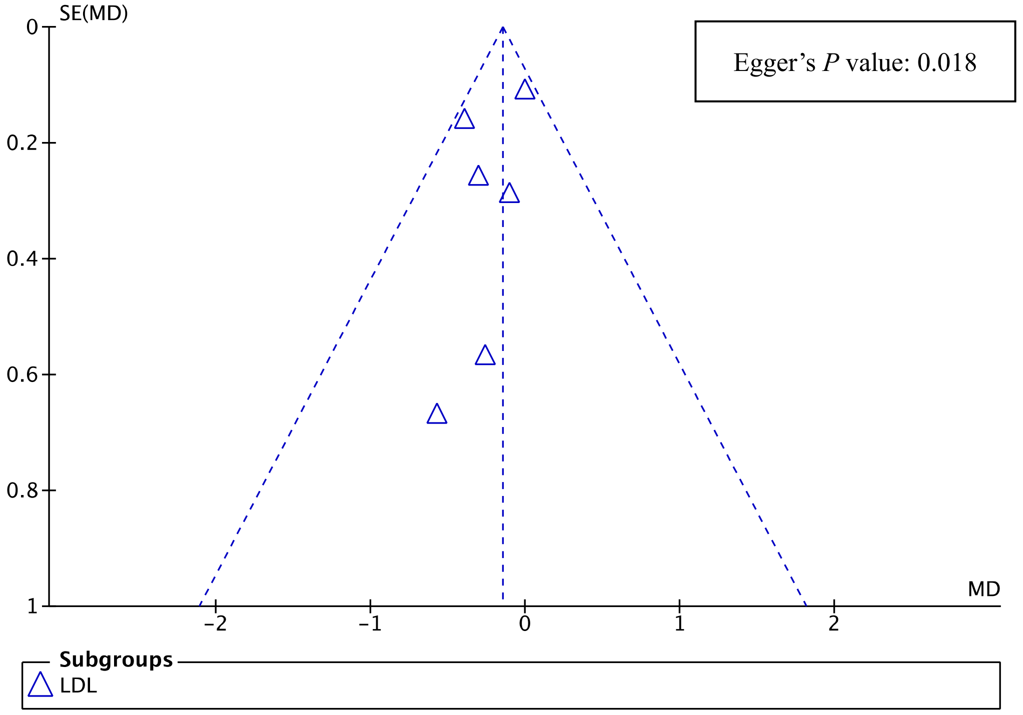

Figure S3-9-4 Funnel plot of RCTs investigating the effect of hesperidin supplementation on LDL.

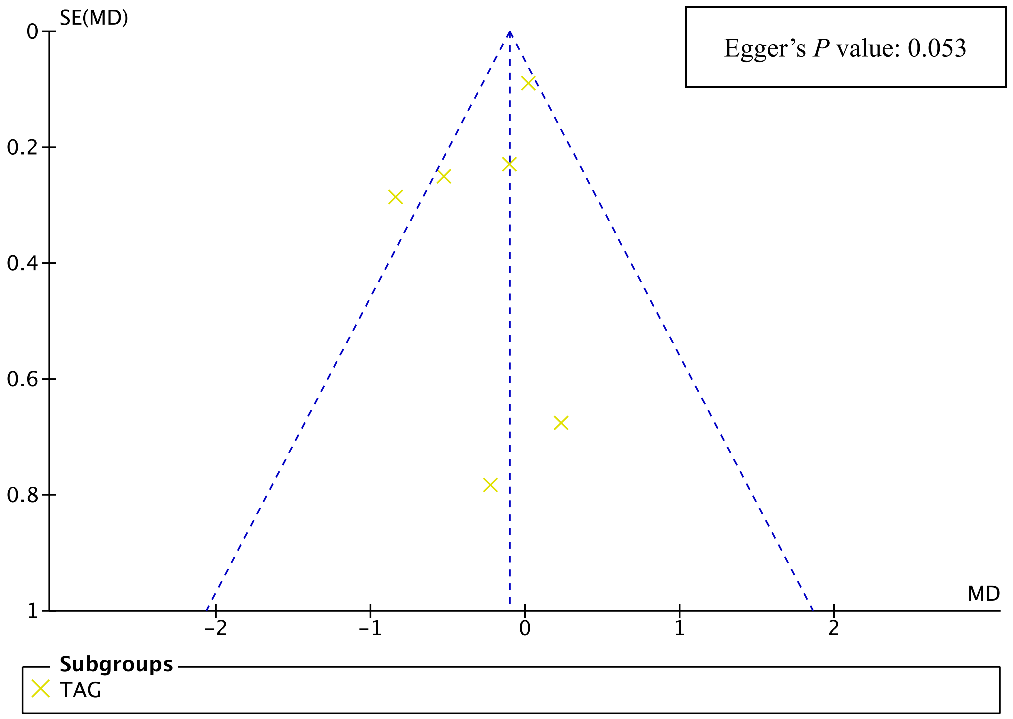

Figure S3-9-5 Funnel plot of RCTs investigating the effect of hesperidin supplementation on TG.

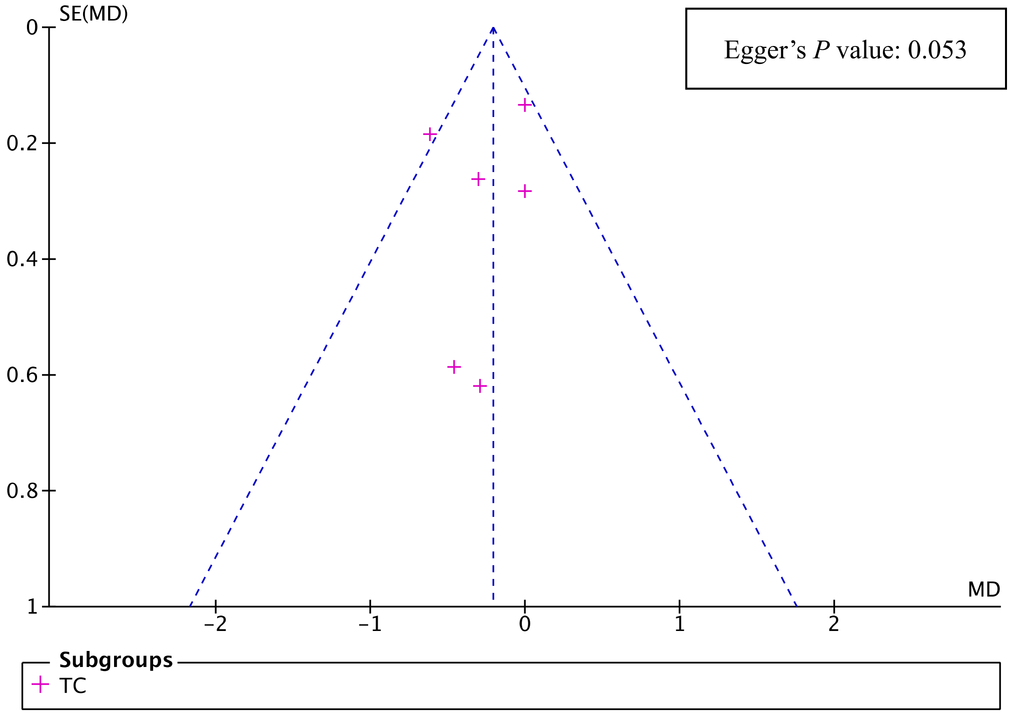

Figure S3-9-6 Funnel plot of RCTs investigating the effect of hesperidin supplementation on TC.

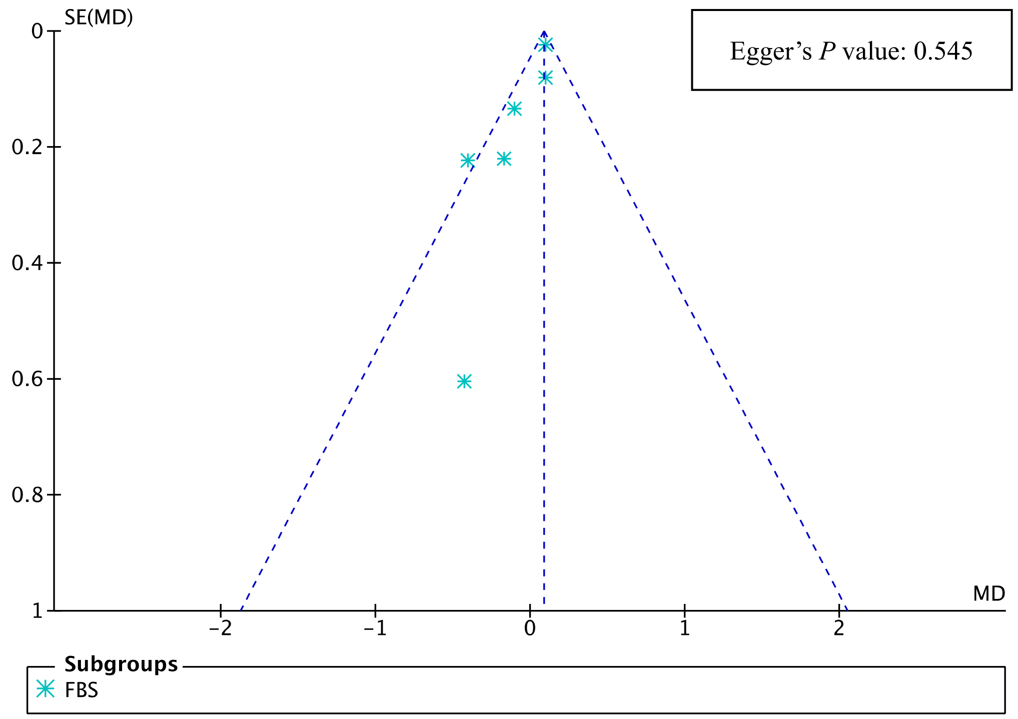

Figure S3-9-7 Funnel plot of RCTs investigating the effect of hesperidin supplementation on FBG.

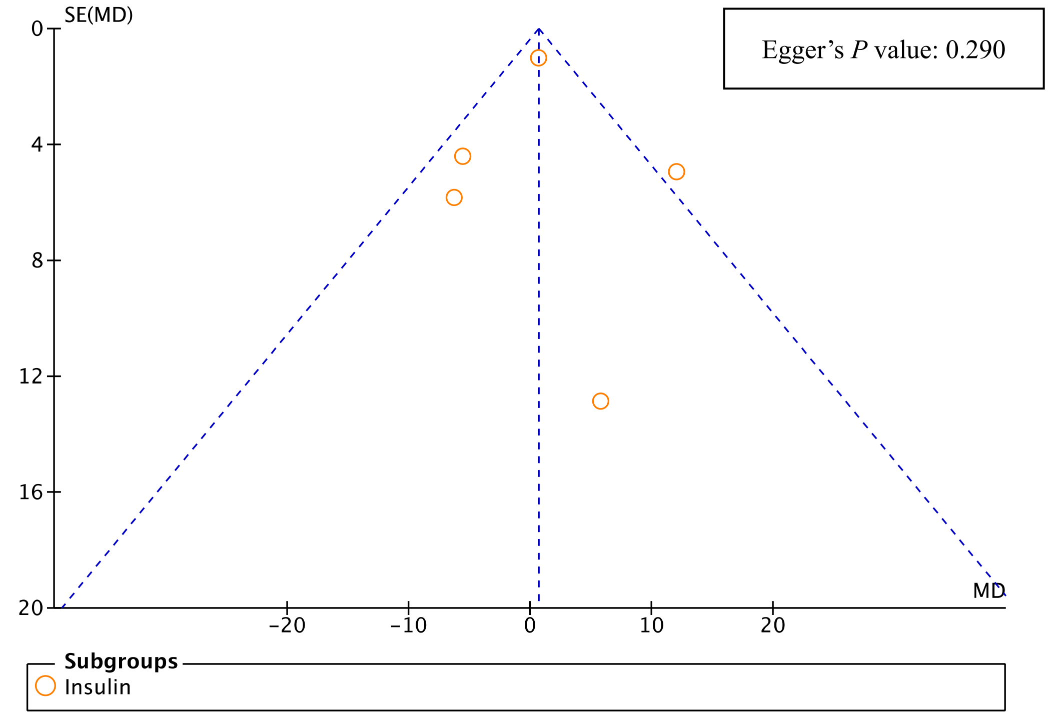

Figure S3-9-8 Funnel plot of RCTs investigating the effect of hesperidin supplementation on FBI.

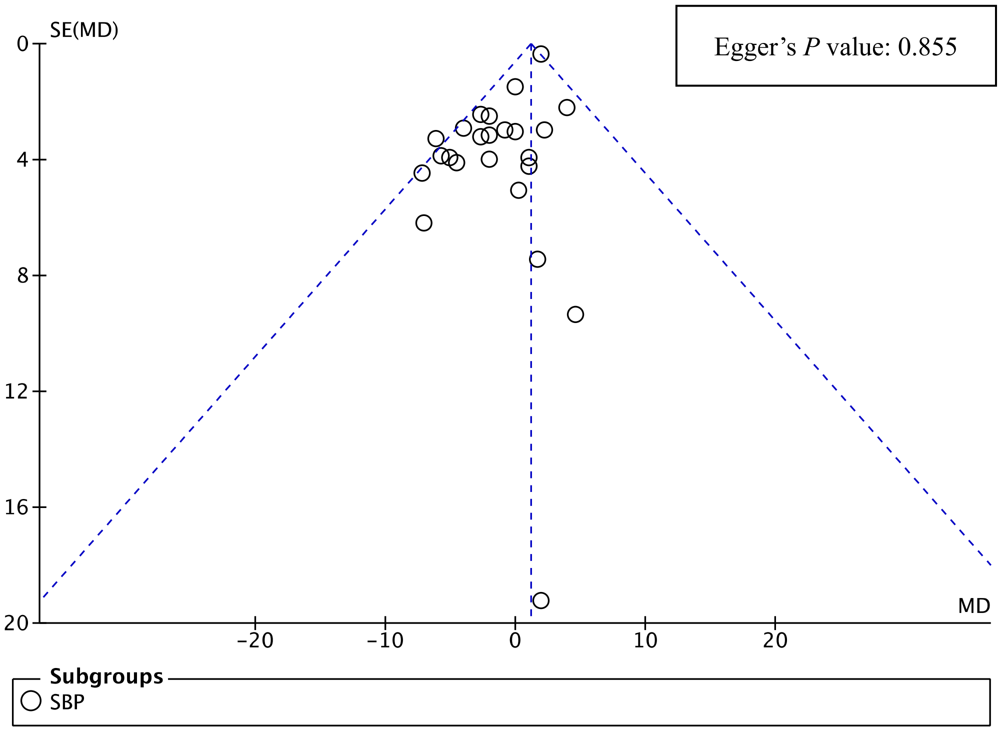

Figure S3-10-1 Funnel plot of RCTs investigating the effect of isoflavone supplementation on SBP.

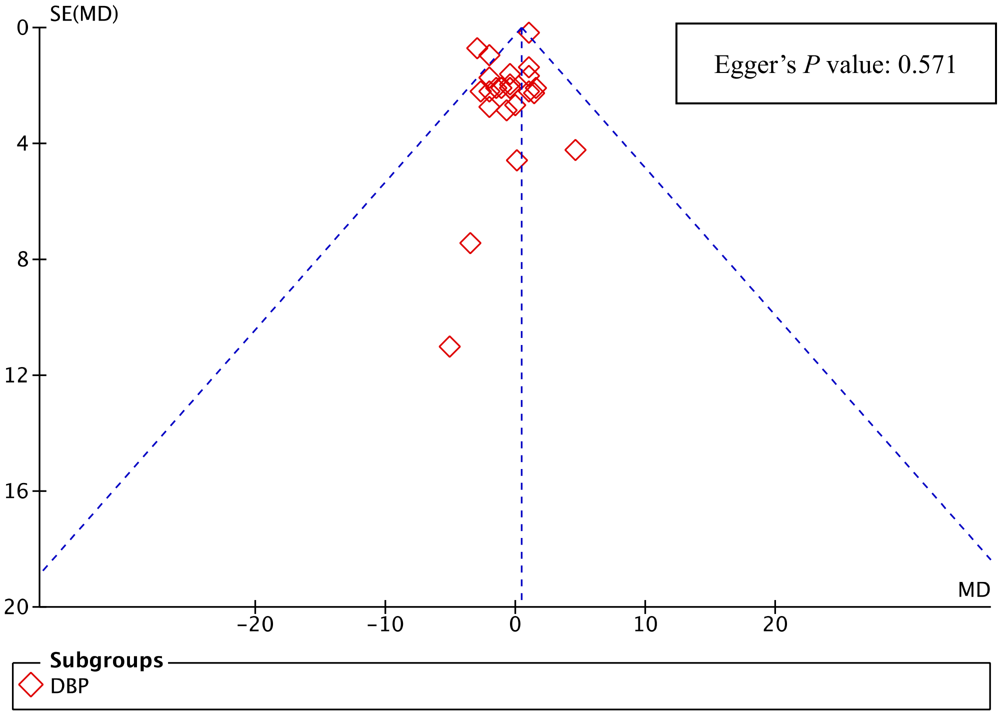

Figure S3-10-2 Funnel plot of RCTs investigating the effect of isoflavone supplementation on DBP.

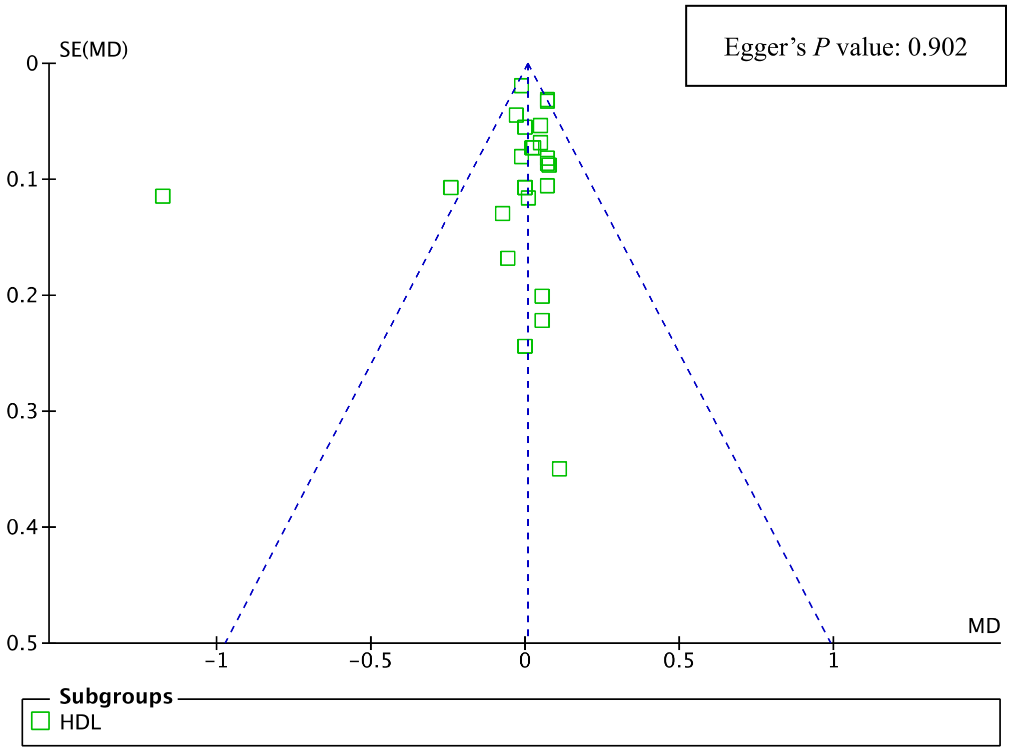

Figure S3-10-3 Funnel plot of RCTs investigating the effect of isoflavone supplementation on HDL.

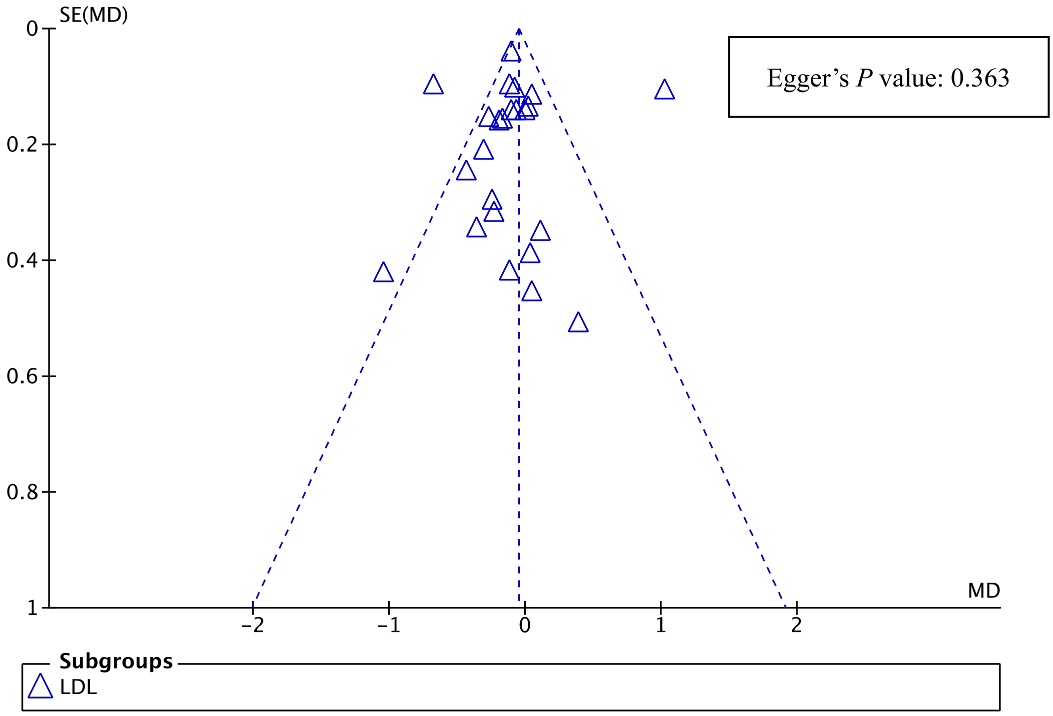

Figure S3-10-4 Funnel plot of RCTs investigating the effect of isoflavone supplementation on LDL.

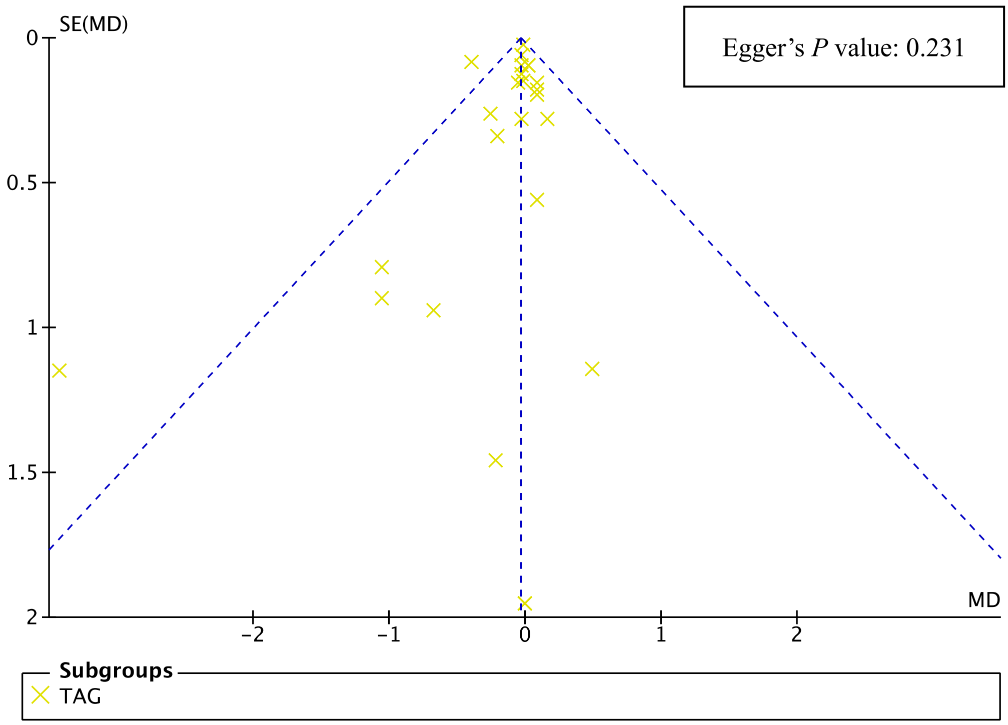

Figure S3-10-5 Funnel plot of RCTs investigating the effect of isoflavone supplementation on TG.

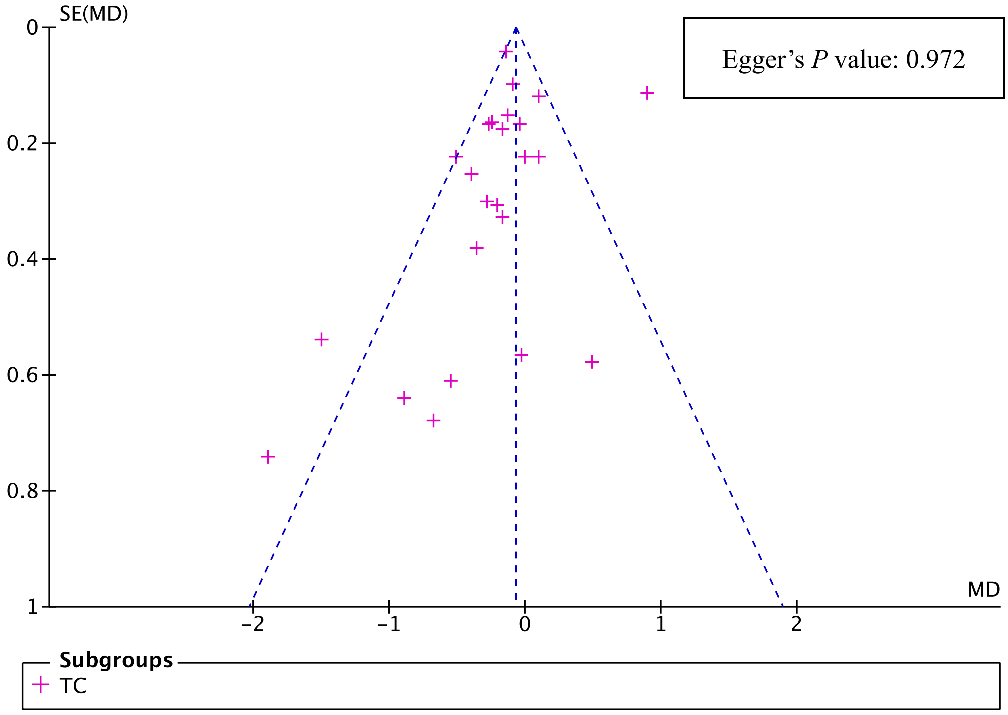

Figure S3-10-6 Funnel plot of RCTs investigating the effect of isoflavone supplementation on TC.

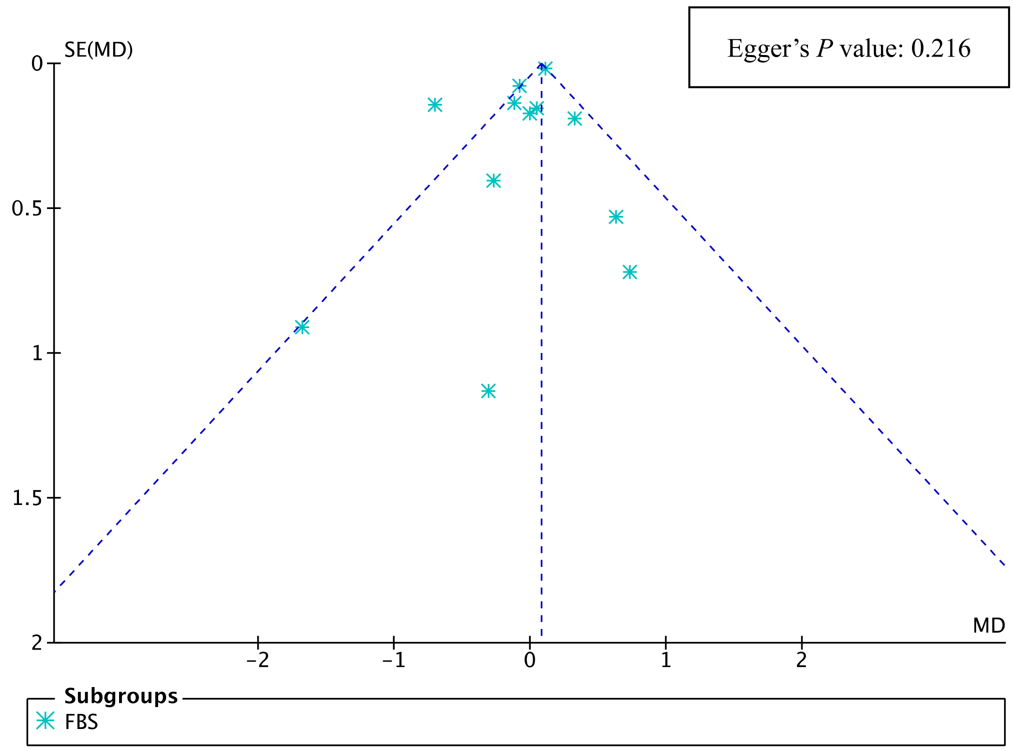

Figure S3-10-7 Funnel plot of RCTs investigating the effect of isoflavone supplementation on FBG.

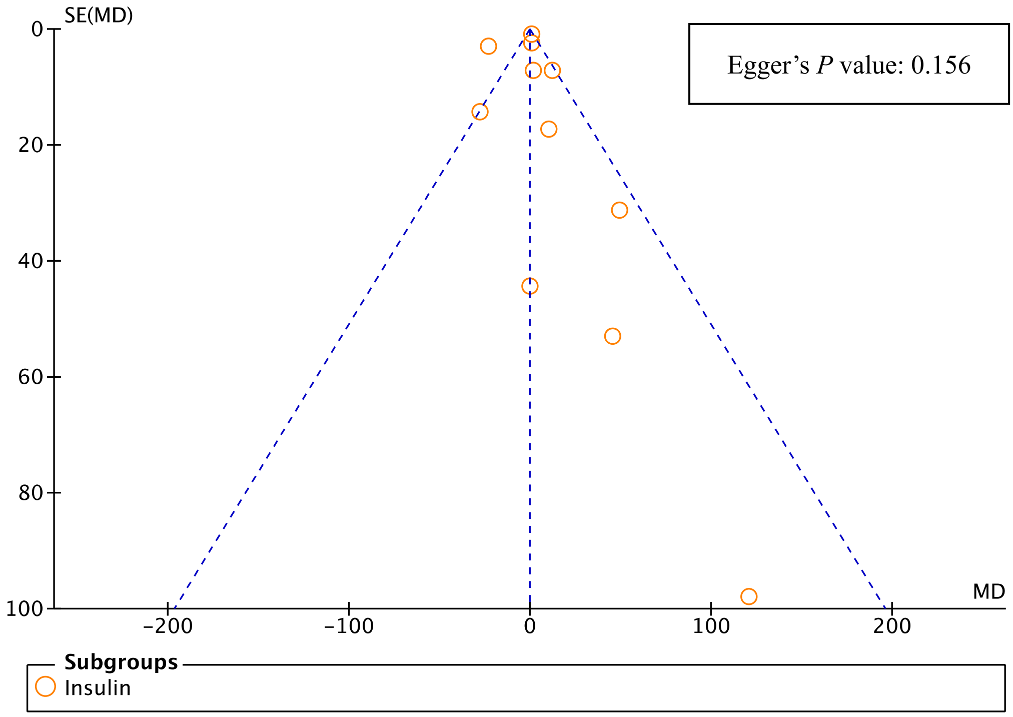

Figure S3-10-8 Funnel plot of RCTs investigating the effect of isoflavone supplementation on FBI.

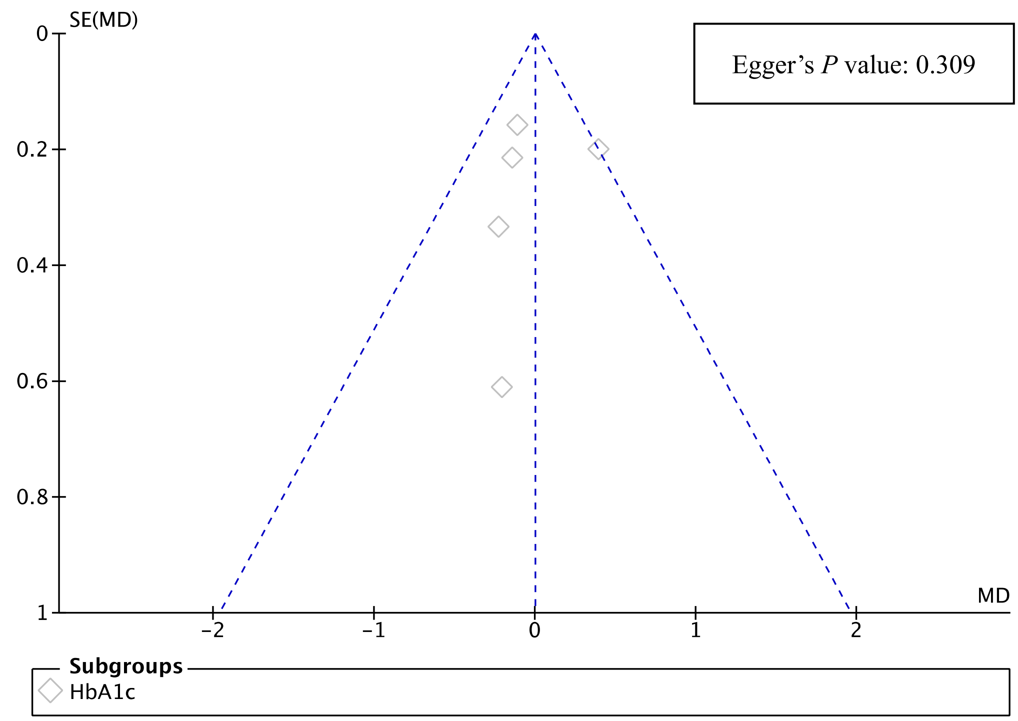

Figure S3-10-9 Funnel plot of RCTs investigating the effect of isoflavone supplementation on A1C.

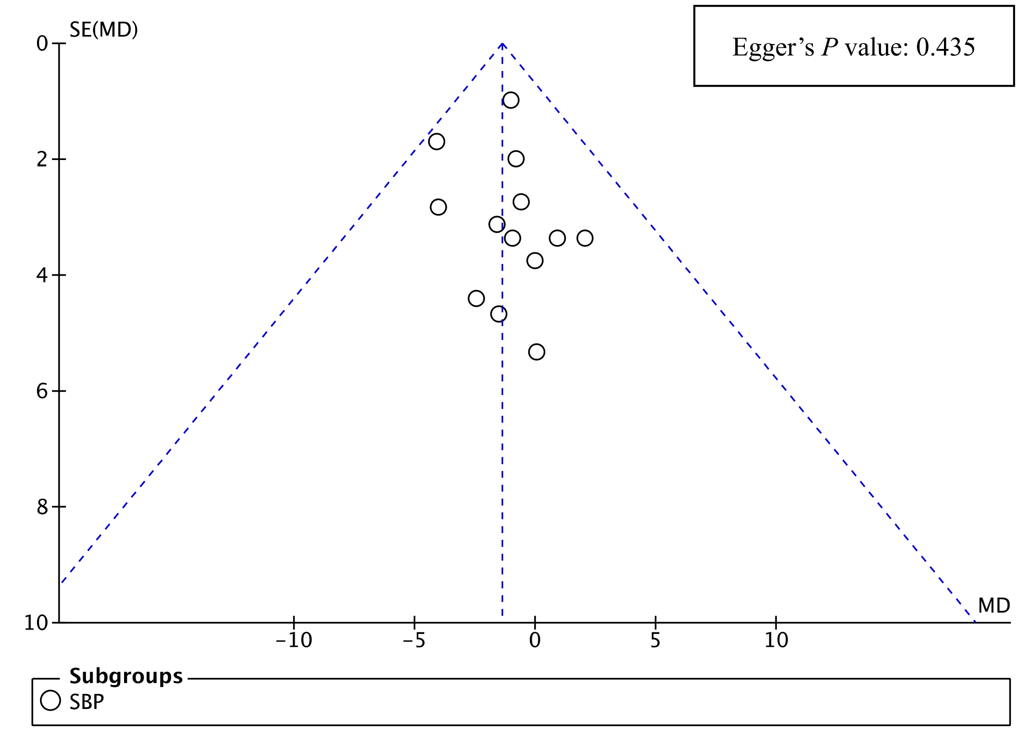

Figure S3-11-1 Funnel plot of RCTs investigating the effect of antioxidant phenolics supplementation on SBP in population with obesity.

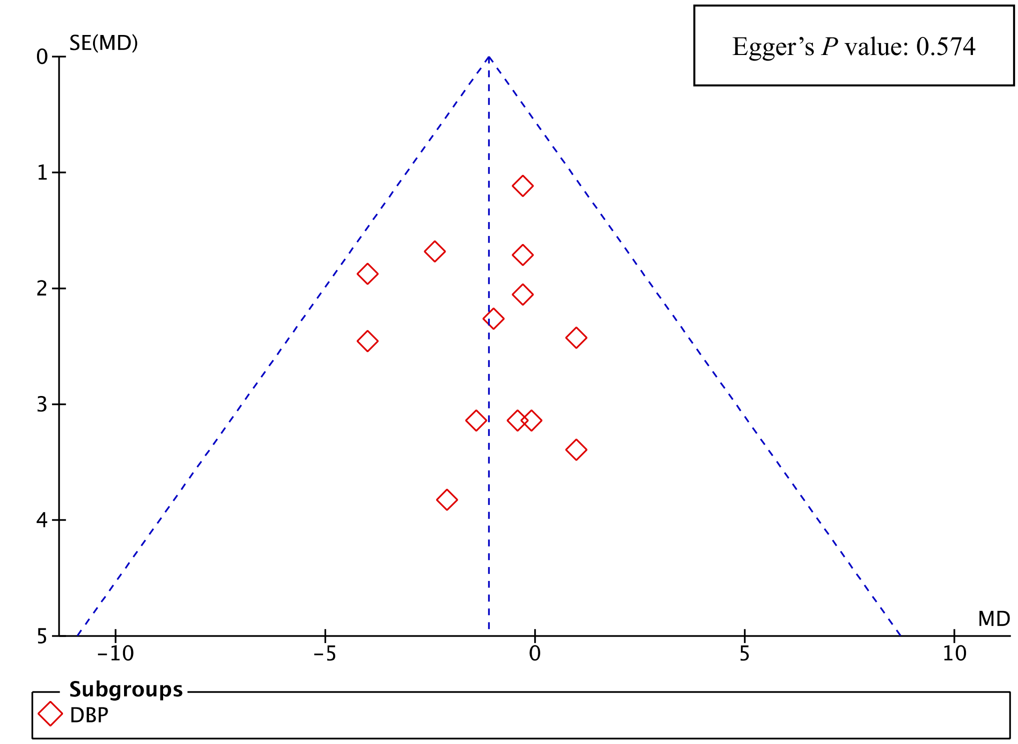

Figure S3-11-2 Funnel plot of RCTs investigating the effect of antioxidant phenolics supplementation on DBP in population with obesity.

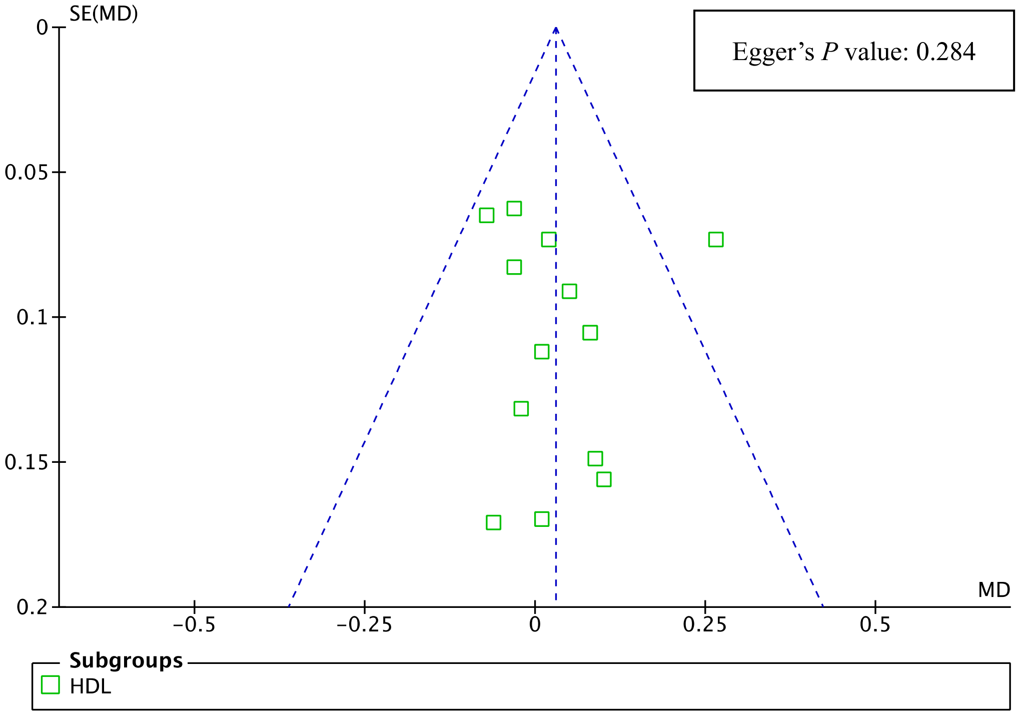

Figure S3-11-3 Funnel plot of RCTs investigating the effect of antioxidant phenolics supplementation on HDL in population with obesity.

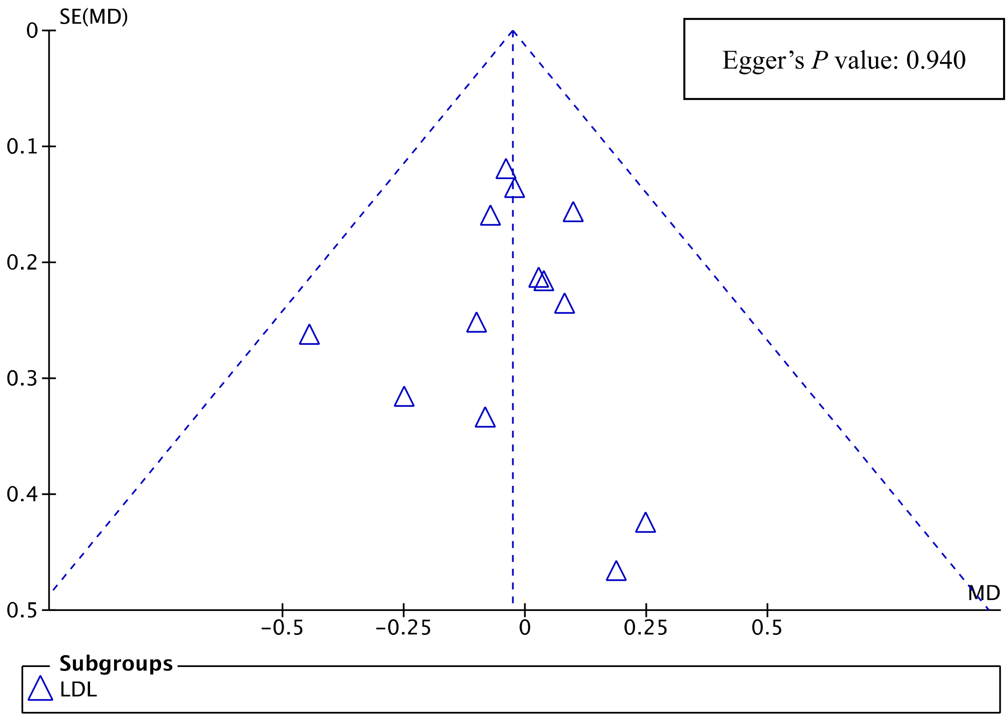

Figure S3-11-4 Funnel plot of RCTs investigating the effect of antioxidant phenolics supplementation on LDL in population with obesity.

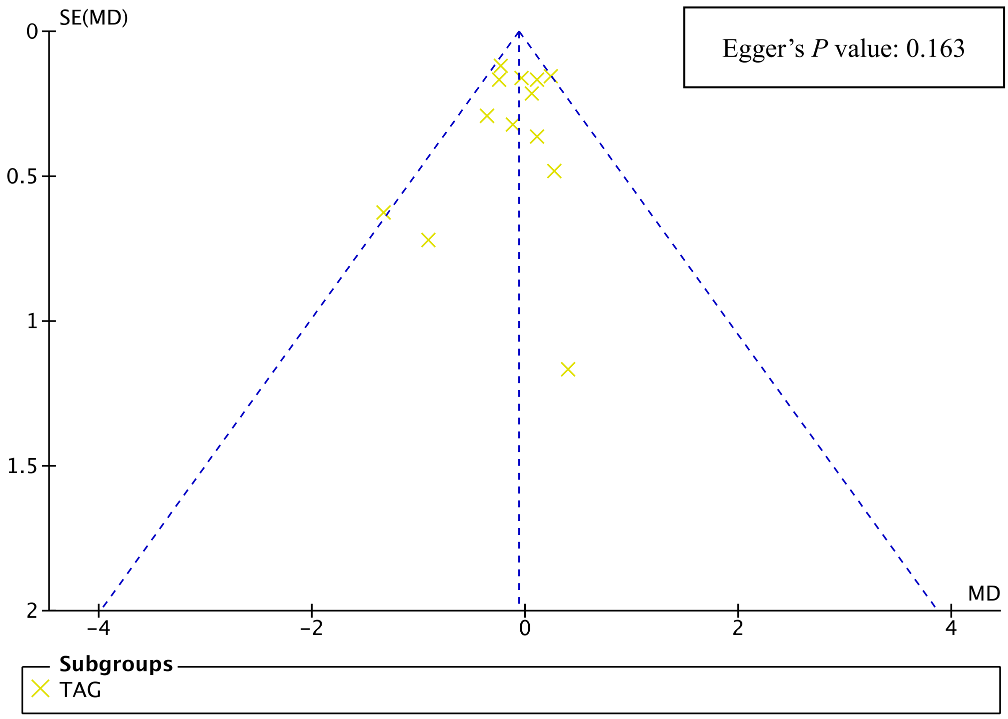

Figure S3-11-5 Funnel plot of RCTs investigating the effect of antioxidant phenolics supplementation on TG in population with obesity.

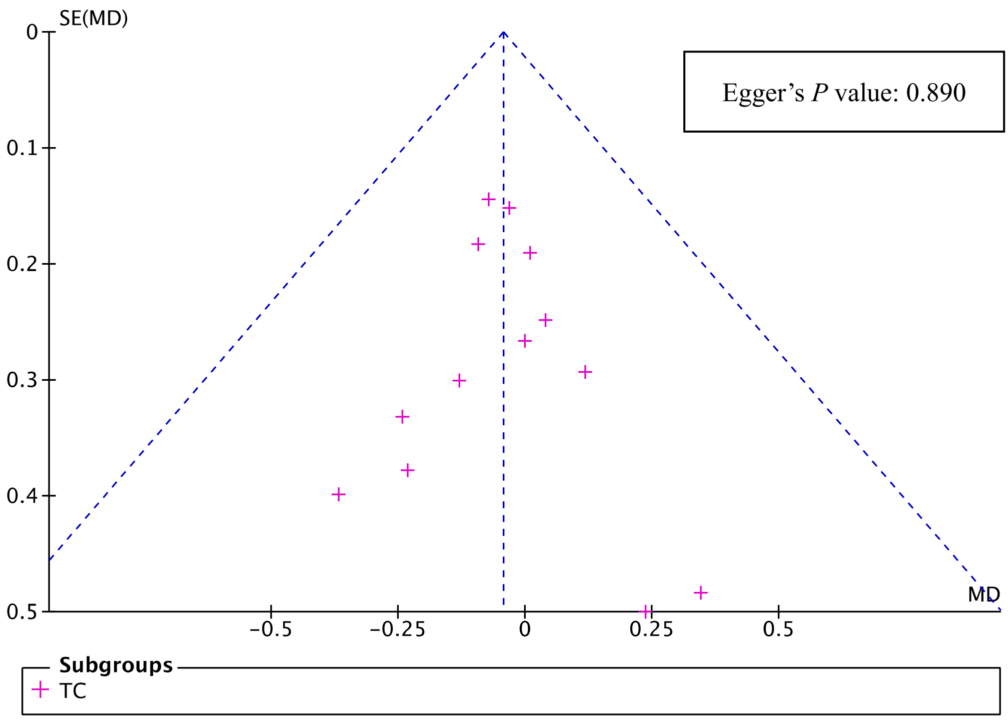

Figure S3-11-6 Funnel plot of RCTs investigating the effect of antioxidant phenolics supplementation on TC in population with obesity.

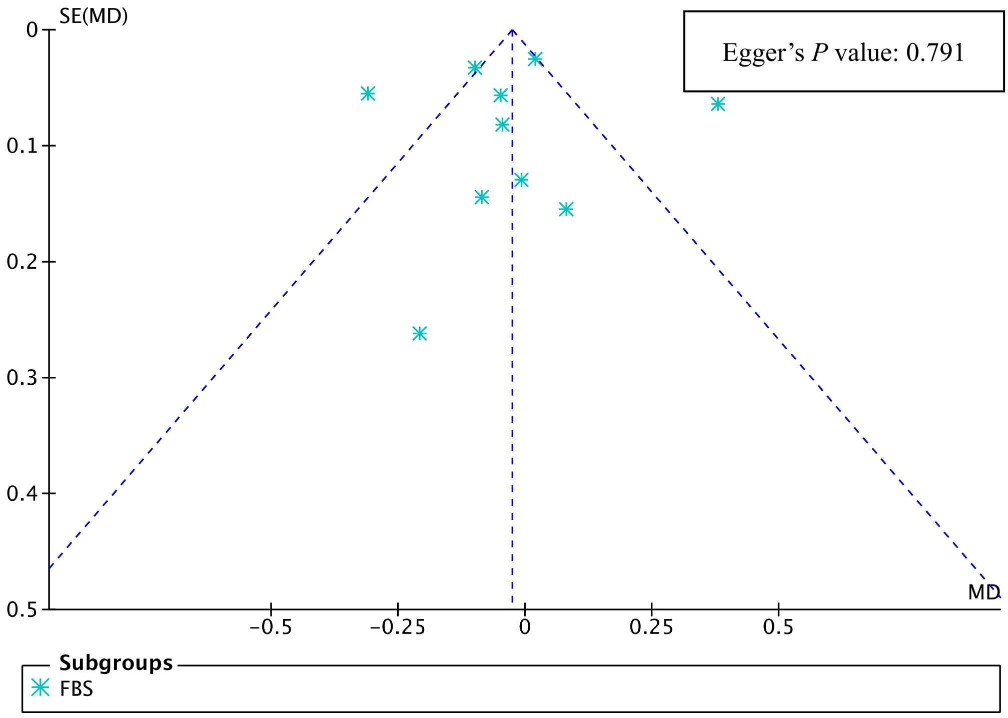

Figure S3-11-7 Funnel plot of RCTs investigating the effect of antioxidant phenolics supplementation on FBG in population with obesity.

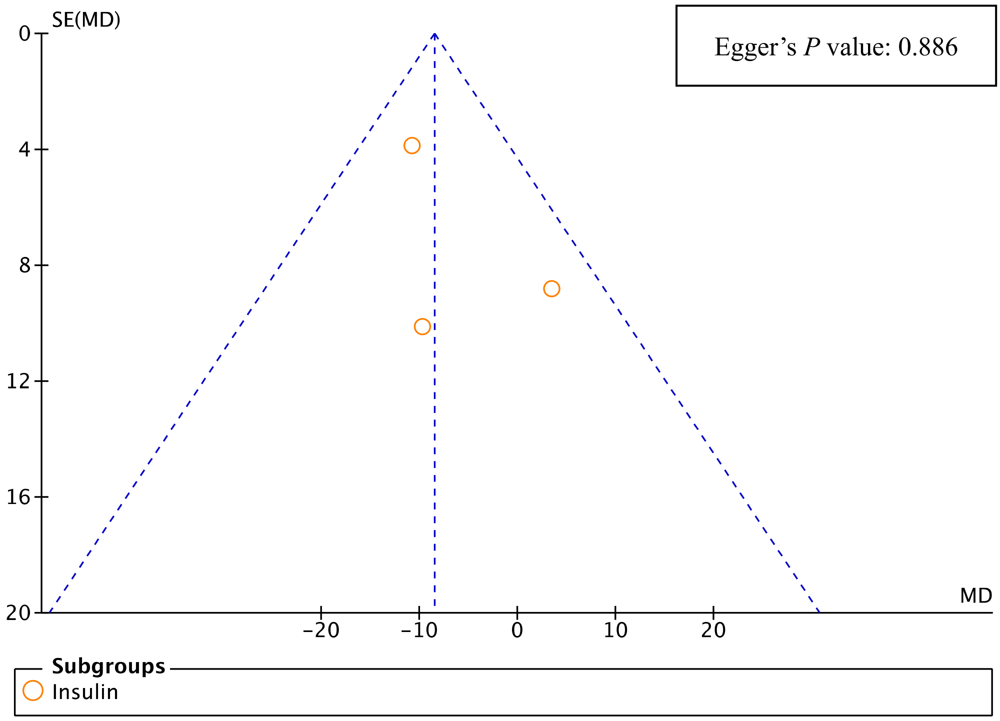

Figure S3-11-8 Funnel plot of RCTs investigating the effect of antioxidant phenolics supplementation on FBI in population with obesity.

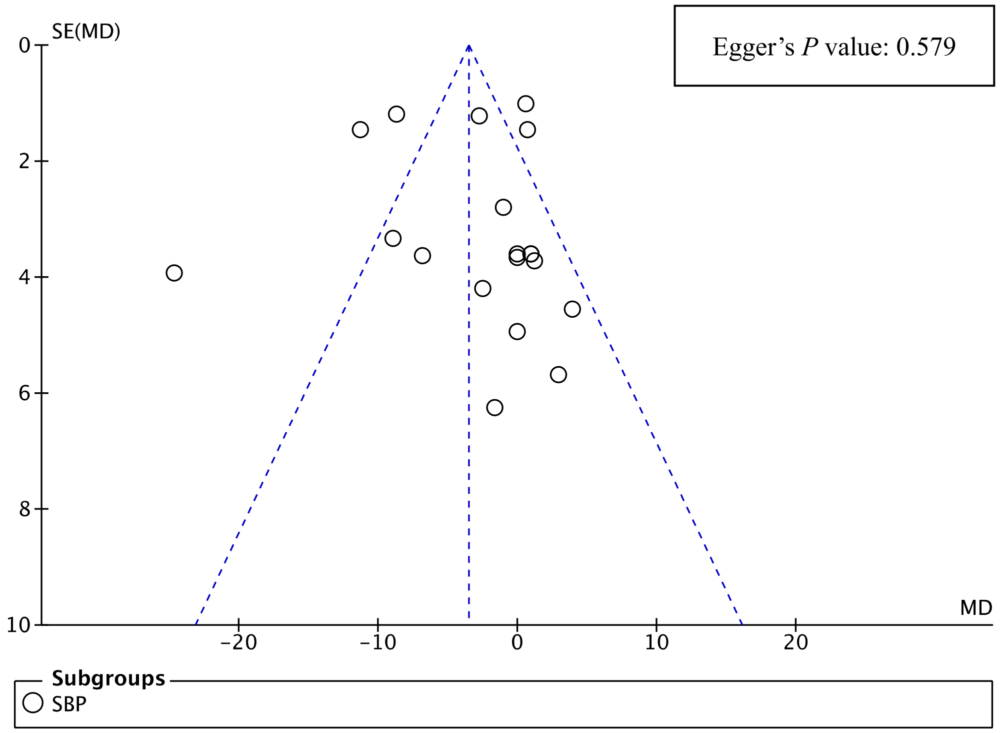

Figure S3-12-1 Funnel plot of RCTs investigating the effect of resveratrol supplementation on SBP.

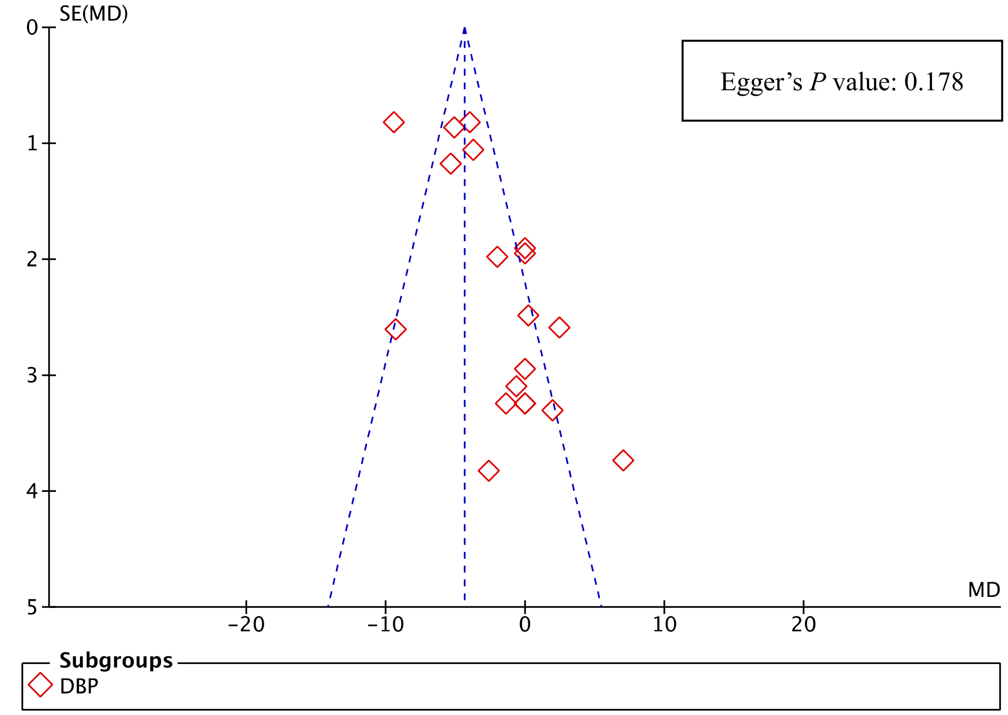

Figure S3-12-2 Funnel plot of RCTs investigating the effect of resveratrol supplementation on DBP.

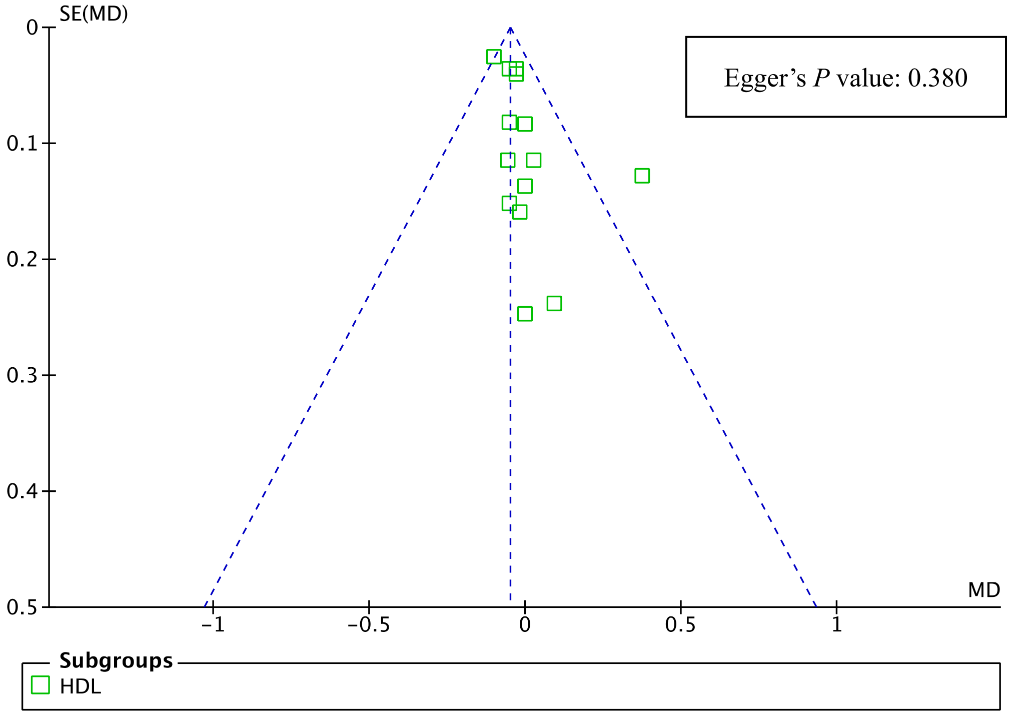

Figure S3-12-3 Funnel plot of RCTs investigating the effect of resveratrol supplementation on HDL.

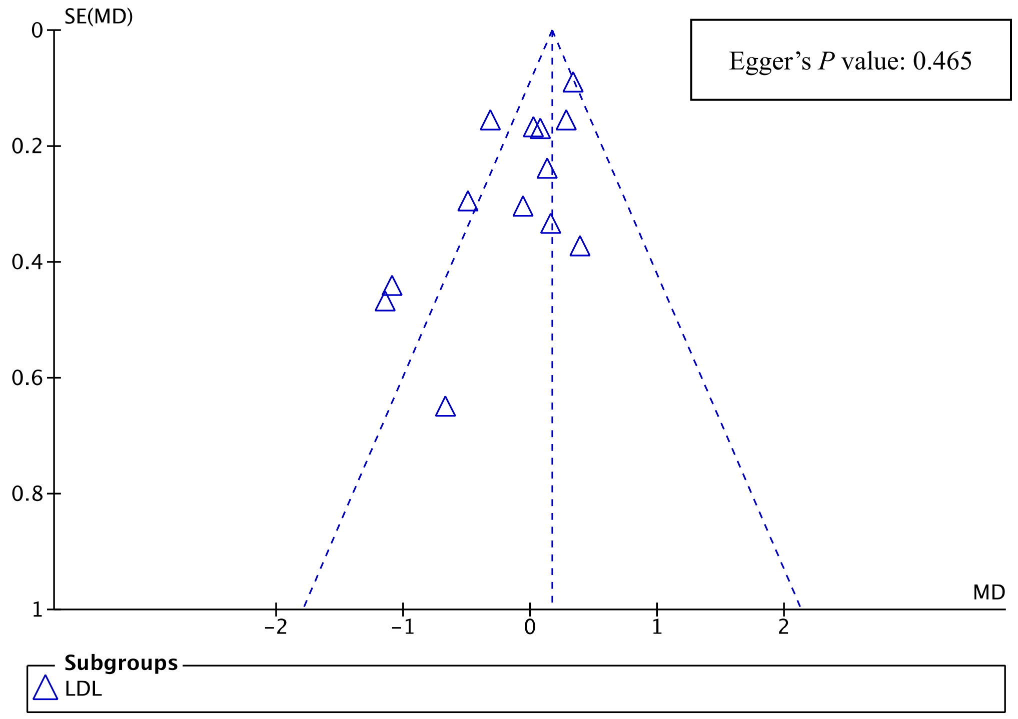

Figure S3-12-4 Funnel plot of RCTs investigating the effect of resveratrol supplementation on LDL.

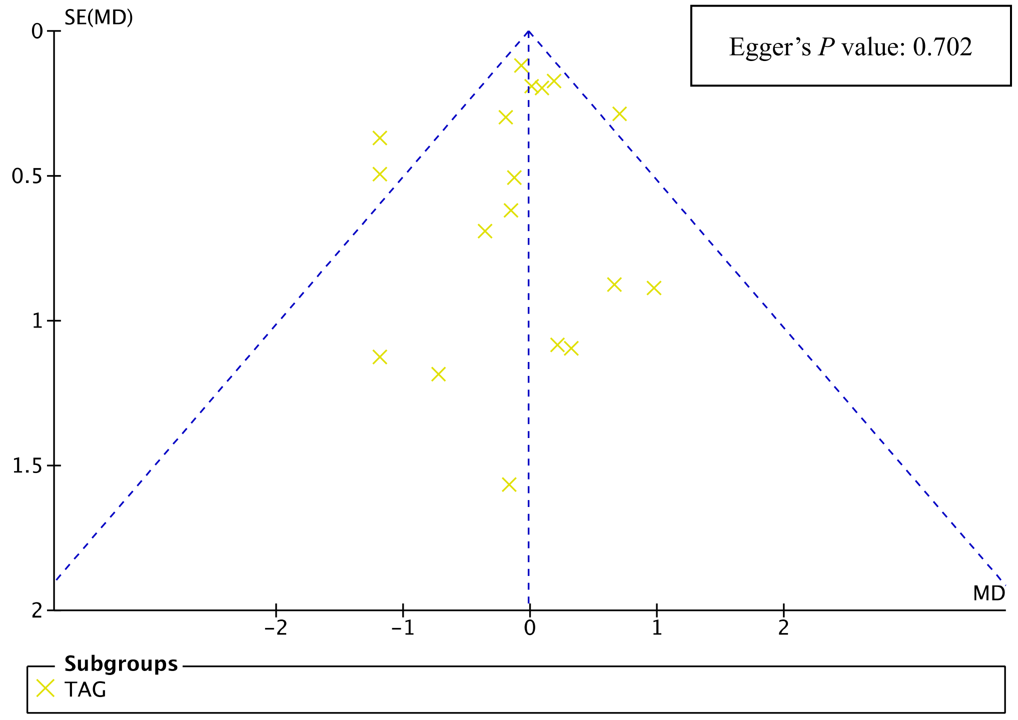

Figure S3-12-5 Funnel plot of RCTs investigating the effect of resveratrol supplementation on TG.

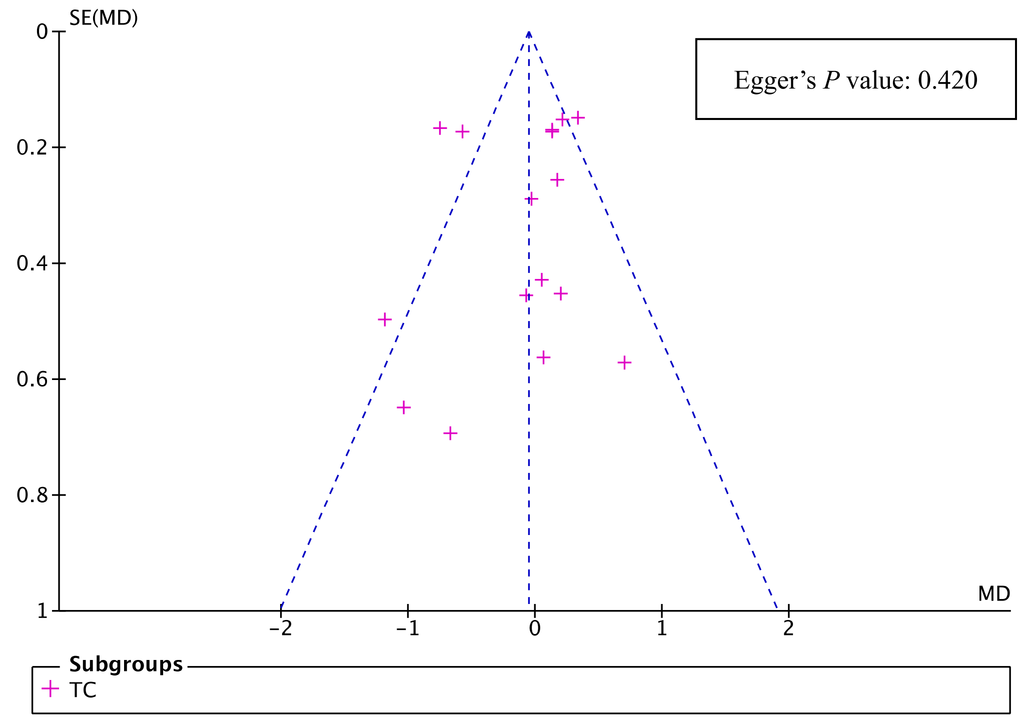

Figure S3-12-6 Funnel plot of RCTs investigating the effect of resveratrol supplementation on TC.

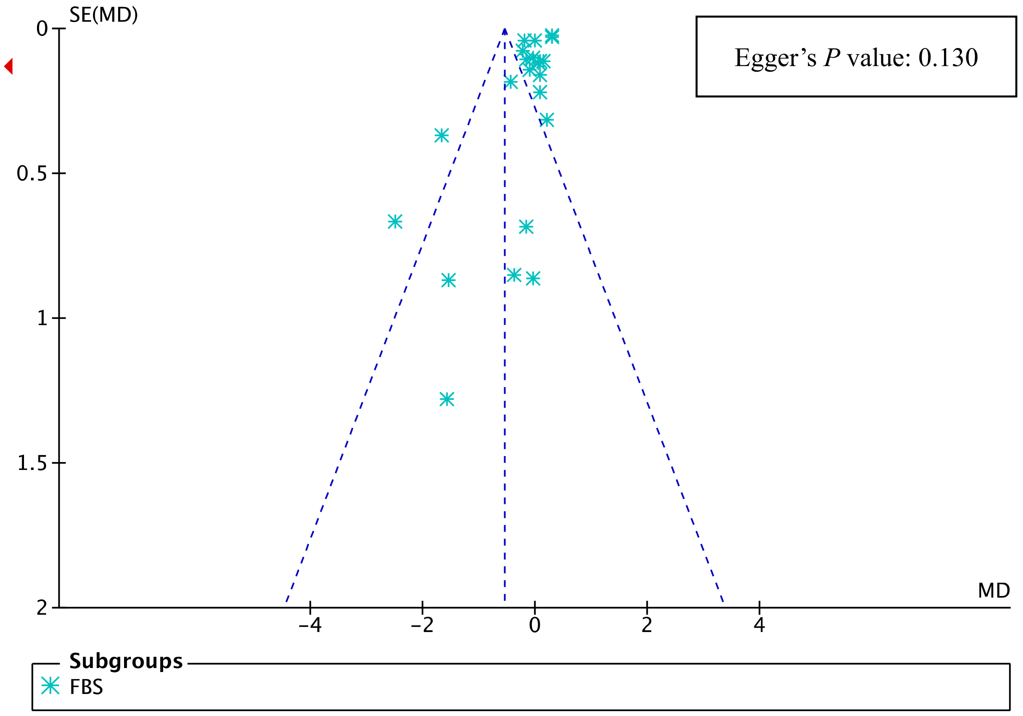

Figure S3-12-7 Funnel plot of RCTs investigating the effect of resveratrol supplementation on FBG.

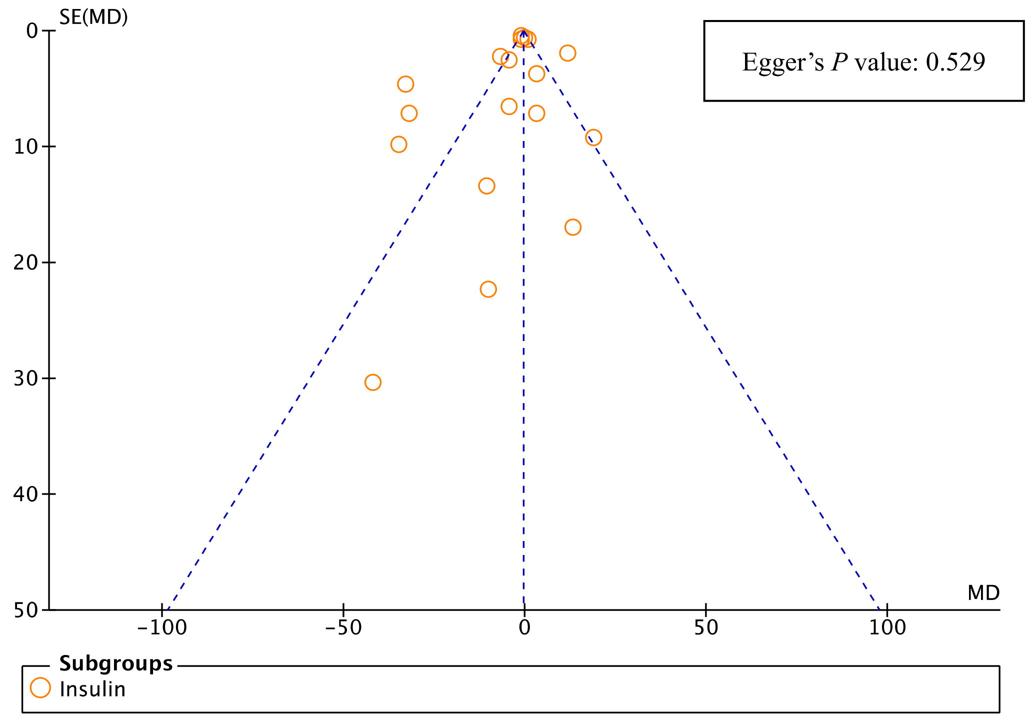

Figure S3-12-8 Funnel plot of RCTs investigating the effect of resveratrol supplementation on FBI.

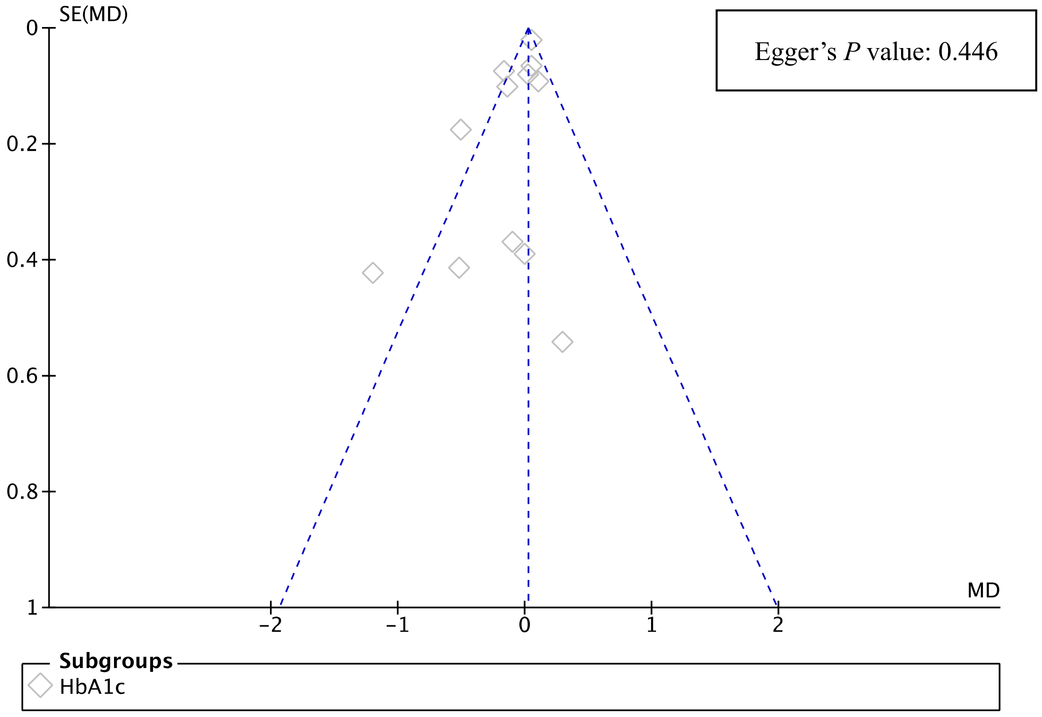

Figure S3-12-9 Funnel plot of RCTs investigating the effect of resveratrol supplementation on A1C.

Appendix 5 – GRADE

Table S2-1. GRADE profile for anthocyanin supplementation.

Certainty assessment							№ of patients		Effect		Certainty	Importance
№ of studies	Study design	Risk of bias	Inconsistency	Indirectness	Imprecision	Other considerations	Anthocyanin	Placebo	Relative (95% CI)	Absolute (95% CI)		
**Outcome - SBP**												
29	randomised trials	not serious	not serious	not serious	serious[a]	none	717	708	-	MD **0.43 lower** (1.82 lower to 0.96 higher)	⨁⨁⨁◯ Moderate	
**Outcome - DBP**												
28	randomised trials	not serious	not serious	not serious	serious[a]	none	633	681	-	MD **0.57 lower** (1.87 lower to 0.74 higher)	⨁⨁⨁◯ Moderate	
**Outcome - HDL**												

Certainty assessment							№ of patients		Effect		Certainty	Importance
№ of studies	Study design	Risk of bias	Inconsistency	Indirectness	Imprecision	Other considerations	Anthocyanin	Placebo	Relative (95% CI)	Absolute (95% CI)		
35	randomised trials	not serious	serious[b]	not serious	not serious	none	1010	980	-	MD **0.18 higher** (0.12 higher to 0.25 higher)	⊕⊕⊕◯ Moderate	
**Outcome - LDL**												
34	randomised trials	not serious	serious[b]	not serious	serious[a]	none	976	940	-	MD **0.18 lower** (0.31 lower to 0.06 lower)	⊕⊕◯◯ Low	
**Outcome - TAG**												
34	randomised trials	not serious	serious[b]	not serious	not serious	none	994	958	-	MD **0.47 lower** (0.7 lower to 0.24 lower)	⊕⊕⊕◯ Moderate	
**Outcome - TC**												

Certainty assessment							№ of patients		Effect		Certainty	Importance
№ of studies	Study design	Risk of bias	Inconsistency	Indirectness	Imprecision	Other considerations	Anthocyanin	Placebo	Relative (95% CI)	Absolute (95% CI)		
36	randomised trials	not serious	serious[b]	not serious	serious[a]	none	1045	1009	-	MD **0.18 lower** (0.33 lower to 0.02 lower)	⊕⊕◯◯ Low	
**Outcome - FBS**												
30	randomised trials	not serious	serious[b]	not serious	serious[a]	none	799	776	-	MD **0.09 lower** (0.17 lower to 0.02 lower)	⊕⊕◯◯ Low	
**Outcome - Insulin**												
15	randomised trials	not serious	serious[b]	not serious	serious[a]	none	446	451	-	MD **4.34 higher** (0.74 lower to 9.43 higher)	⊕⊕◯◯ Low	
**Outcome - HbA1c**												

Certainty assessment							№ of patients		Effect		Certainty	Importance
№ of studies	Study design	Risk of bias	Inconsistency	Indirectness	Imprecision	Other considerations	Anthocyanin	Placebo	Relative (95% CI)	Absolute (95% CI)		
9	randomised trials	not serious	not serious	not serious	serious[a]	none	291	291	-	MD **0** (0.09 lower to 0.1 higher)	⨁⨁⨁◯ Moderate	

CI: confidence interval; **MD:** mean difference

Explanations

a. Rated down for imprecision.

b. Rated down for inconsistency.

Table S2-2. GRADE profile for catechin supplementation.

Certainty assessment							№ of patients		Effect		Certainty	Importance
№ of studies	Study design	Risk of bias	Inconsistency	Indirectness	Imprecision	Other considerations	Catechin	Placebo	Relative (95% CI)	Absolute (95% CI)		

Outcome - HDL

Certainty assessment							№ of patients		Effect		Certainty	Importance
№ of studies	Study design	Risk of bias	Inconsistency	Indirectness	Imprecision	Other considerations	Catechin	Placebo	Relative (95% CI)	Absolute (95% CI)		
30	randomised trials	not serious	not serious	not serious	serious[a]	none	947	926	-	MD **0.01 lower** (0.04 lower to 0.02 higher)	⊕⊕⊕◯ Moderate[a]	

Outcome - LDL

27	randomised trials	not serious	not serious	not serious	serious[a]	none	890	870	-	MD **0.06 lower** (0.14 lower to 0.02 higher)	⊕⊕⊕◯ Moderate[a]	

Outcome - TAG

31	randomised trials	not serious	not serious	not serious	serious[a]	none	965	940	-	MD **0.06 lower** (0.14 lower to 0.02 higher)	⊕⊕⊕◯ Moderate[a]	

Outcome - TC

Certainty assessment							№ of patients		Effect		Certainty	Importance
№ of studies	Study design	Risk of bias	Inconsistency	Indirectness	Imprecision	Other considerations	Catechin	Placebo	Relative (95% CI)	Absolute (95% CI)		
28	randomised trials	not serious	not serious	not serious	serious[a]	none	895	870	-	MD **0.15 lower** (0.24 lower to 0.07 lower)	⊕⊕⊕◯ Moderate[a]	
**Outcome - FBS**												
28	randomised trials	not serious	serious[b]	not serious	serious[a]	none	1045	1029	-	MD **0.1 lower** (0.18 lower to 0.03 lower)	⊕⊕◯◯ Low[a,b]	
**Outcome - Insulin**												
22	randomised trials	not serious	serious[b]	not serious	serious[a]	none	806	797	-	MD **0.08 lower** (1.22 lower to 1.06 higher)	⊕⊕◯◯ Low[a,b]	
**Outcome - HbA1c**												

Certainty assessment							№ of patients		Effect		Certainty	Importance
№ of studies	Study design	Risk of bias	Inconsistency	Indirectness	Imprecision	Other considerations	Catechin	Placebo	Relative (95% CI)	Absolute (95% CI)		
9	randomised trials	not serious	serious[b]	not serious	serious[a]	none	315	308	-	MD **0.12 lower** (0.23 lower to 0.02 lower)	⊕⊕◯◯ Low[a,b]	
**Outcome - SBP**												
31	randomised trials	not serious	not serious	not serious	serious[a]	none	949	920	-	MD **1.56 lower** (2.75 lower to 0.37 lower)	⊕⊕⊕◯ Moderate[a]	
**Outcome - DBP**												
29	randomised trials	not serious	not serious	not serious	serious[a]	none	859	834	-	MD **0.95 lower** (1.69 lower to 0.2 lower)	⊕⊕⊕◯ Moderate[a]	

CI: confidence interval; **MD:** mean difference

Explanations

a. Rated down for imprecision.

b. Rated down for inconsistency.

Table S2-3. GRADE profile for chlorogenic acid supplementation.

Certainty assessment							№ of patients		Effect		Certainty	Importance
№ of studies	Study design	Risk of bias	Inconsistency	Indirectness	Imprecision	Other considerations	Chlorogenic acid	Placebo	Relative (95% CI)	Absolute (95% CI)		
**CVD - SBP**												
10	randomised trials	not serious	serious[a]	not serious	serious[b]	none	331	331	-	MD **1.04 lower** (3.52 lower to 1.44 higher)	⨁⨁◯◯ Low	
**CVD - DBP**												
10	randomised trials	not serious	serious[a]	not serious	serious[b]	none	331	331	-	MD **0.74 lower** (2.1 lower to 0.61 higher)	⨁⨁◯◯ Low	

Certainty assessment							№ of patients		Effect		Certainty	Importance
№ of studies	Study design	Risk of bias	Inconsistency	Indirectness	Imprecision	Other considerations	Chlorogenic acid	Placebo	Relative (95% CI)	Absolute (95% CI)		
**CVD - HDL**												
21	randomised trials	not serious	serious[a]	not serious	serious[b]	none	562	553	-	MD **0.01 lower** (0.05 lower to 0.03 higher)	⨁⨁◯◯ Low	
**CVD - LDL**												
18	randomised trials	not serious	not serious	not serious	serious[b]	none	413	404	-	MD **0.24 lower** (0.38 lower to 0.11 lower)	⨁⨁⨁◯ Moderate	
**CVD - TG**												
23	randomised trials	not serious	serious[a]	not serious	serious[b]	none	614	605	-	MD **0.1 lower** (0.15 lower to 0.04 lower)	⨁⨁◯◯ Low	

Certainty assessment							№ of patients		Effect		Certainty	Importance
№ of studies	Study design	Risk of bias	Inconsistency	Indirectness	Imprecision	Other considerations	Chlorogenic acid	Placebo	Relative (95% CI)	Absolute (95% CI)		
**CVD - TC**												
19	randomised trials	not serious	serious[a]	not serious	serious[b]	none	423	414	-	MD **0.39 lower** (0.62 lower to 0.16 lower)	⊕⊕◯◯ Low	
**CVD - FBG**												
16	randomised trials	not serious	serious[a]	not serious	serious[b]	none	404	401	-	MD **0.16 lower** (0.27 lower to 0.06 lower)	⊕⊕◯◯ Low	
**CVD - FBI**												
7	randomised trials	not serious	serious[a]	not serious	serious[b]	none	171	176	-	MD **5.36 lower** (9.62 lower to 1.1 lower)	⊕⊕◯◯ Low	

Certainty assessment							№ of patients		Effect		Certainty	Importance
№ of studies	Study design	Risk of bias	Inconsistency	Indirectness	Imprecision	Other considerations	Chlorogenic acid	Placebo	Relative (95% CI)	Absolute (95% CI)		
**CVD - HbA1c**												
2	randomised trials	not serious	serious[a]	not serious	serious[b]	none	58	42	-	MD **0.36 lower** (1.25 lower to 0.53 higher)	⨁⨁◯◯ Low	

CI: confidence interval; **MD:** mean difference

Explanations

a. Rated down for inconsistency.

b. Rate down for imprecision.

Table S2-4. GRADE profile for curcumin supplementation.

Certainty assessment							№ of patients		Effect		Certainty	Importance
№ of studies	Study design	Risk of bias	Inconsistency	Indirectness	Imprecision	Other considerations	Curcumin	Placebo	Relative (95% CI)	Absolute (95% CI)		
**Outcome - SBP**												
14	randomised trials	not serious	not serious	not serious	serious[a]	none	456	456	-	MD **1.42 lower** (2.56 lower to 0.28 lower)	⨁⨁⨁◯ Moderate[a]	
**Outcome - DBP**												
13	randomised trials	not serious	serious[b]	not serious	serious[a]	none	451	443	-	MD **0.03 higher** (1.18 lower to 1.24 higher)	⨁⨁◯◯ Low[a,b]	
**Outcome - HDL**												
18	randomised trials	not serious	serious[b]	not serious	serious[a]	none	542	545	-	MD **0.39 higher** (0.22 higher to 0.56 higher)	⨁⨁◯◯ Low[a,b]	

Certainty assessment							№ of patients		Effect		Certainty	Importance
№ of studies	Study design	Risk of bias	Inconsistency	Indirectness	Imprecision	Other considerations	Curcumin	Placebo	Relative (95% CI)	Absolute (95% CI)		
**Outcome - TAG**												
15	randomised trials	not serious	serious[b]	not serious	serious[a]	none	505	509	-	MD 0.19 **lower** (0.45 lower to 0.06 higher)	⨁⨁◯◯ Low[a,b]	
**Outcome - TC**												
13	randomised trials	not serious	not serious	not serious	serious[a]	none	390	432	-	MD 0.11 **lower** (0.36 lower to 0.15 higher)	⨁⨁⨁◯ Moderate[a]	
**Outcome - LDL**												
17	randomised trials	not serious	serious[b]	not serious	serious[a]	none	638	638	-	MD 0.19 **lower** (0.39 lower to 0)	⨁⨁◯◯ Low[a,b]	

Certainty assessment							№ of patients		Effect		Certainty	Importance
№ of studies	Study design	Risk of bias	Inconsistency	Indirectness	Imprecision	Other considerations	Curcumin	Placebo	Relative (95% CI)	Absolute (95% CI)		
**Outcome - FBS**												
15	randomised trials	not serious	serious[b]	not serious	serious[a]	none	671	679	-	MD **0.43 lower** (0.68 lower to 0.19 lower)	⨁⨁◯◯ Low[a,b]	
**Outcome - Insulin**												
7	randomised trials	not serious	not serious	not serious	not serious	none	288	293	-	MD **10.14 lower** (14.13 lower to 6.14 lower)	⨁⨁⨁⨁ High	
**Outcome - HbA1c**												
10	randomised trials	not serious	serious[b]	not serious	serious[a]	none	518	520	-	MD **0.49 lower** (0.83 lower to 0.14 lower)	⨁⨁◯◯ Low[a,b]	

CI: confidence interval; **MD:** mean difference

Explanations

a. Rated down for imprecision.

b. Rated down for inconsistency.

Table S2-5. GRADE profile for flavanol supplementation.

Certainty assessment							№ of patients		Effect		Certainty	Importance
№ of studies	Study design	Risk of bias	Inconsistency	Indirectness	Imprecision	Other considerations	Flavanols	Placebo	Relative (95% CI)	Absolute (95% CI)		
**Outcome - SBP**												
19	randomised trials	not serious	serious[a]	not serious	serious[b]	none	534	508	-	MD **1.47 lower** (2.89 lower to 0.06 lower)	⨁⨁◯◯ Low	
**Outcome - DBP**												

Certainty assessment							№ of patients		Effect		Certainty	Importance
№ of studies	Study design	Risk of bias	Inconsistency	Indirectness	Imprecision	Other considerations	Flavanols	Placebo	Relative (95% CI)	Absolute (95% CI)		
17	randomised trials	not serious	serious[a]	not serious	serious[b]	none	456	439	-	MD **0.86 lower** (1.99 lower to 0.28 higher)	⊕⊕◯◯ Low	
**Outcome - HDL**												
18	randomised trials	not serious	serious[a]	not serious	serious[b]	none	624	607	-	MD **0.09 higher** (0.04 higher to 0.13 higher)	⊕⊕◯◯ Low	
**Outcome - LDL**												
17	randomised trials	not serious	serious[a]	not serious	serious[b]	none	492	480	-	MD **0.23 lower** (0.67 lower to 0.21 higher)	⊕⊕◯◯ Low	
**Outcome - TG**												

Certainty assessment							№ of patients		Effect		Certainty	Importance
№ of studies	Study design	Risk of bias	Inconsistency	Indirectness	Imprecision	Other considerations	Flavanols	Placebo	Relative (95% CI)	Absolute (95% CI)		
18	randomised trials	not serious	serious[a]	not serious	serious[b]	none	624	607	-	MD **0.04 lower** (0.13 lower to 0.05 higher)	⨁⨁◯◯ Low	

Outcome - TC

18	randomised trials	not serious	serious[a]	not serious	serious[b]	none	617	601	-	MD **0.03 lower** (0.09 lower to 0.04 higher)	⨁⨁◯◯ Low	

Outcome - FBS

15	randomised trials	not serious	serious[a]	not serious	serious[b]	none	469	452	-	MD **0.17 lower** (0.3 lower to 0.03 lower)	⨁⨁◯◯ Low	

Outcome - Insulin

Certainty assessment							№ of patients		Effect		Certainty	Importance
№ of studies	Study design	Risk of bias	Inconsistency	Indirectness	Imprecision	Other considerations	Flavanols	Placebo	Relative (95% CI)	Absolute (95% CI)		
8	randomised trials	not serious	serious[a]	not serious	not serious	none	197	187	-	MD **14.86 lower** (21.02 lower to 8.71 lower)	⊕⊕⊕◯ Moderate	
**Outcome - HbA1c**												
3	randomised trials	not serious	serious[a]	not serious	serious[b]	none	92	82	-	MD **0.2 lower** (0.51 lower to 0.1 higher)	⊕⊕◯◯ Low	

CI: confidence interval; **MD:** mean difference

Explanations

a. Rated down for inconsistency.

b. Rated down for imprecision.

Table S2-6. GRADE profile for flavonoid supplementation.

Certainty assessment							№ of patients		Effect		Certainty	Importance
№ of studies	Study design	Risk of bias	Inconsistency	Indirectness	Imprecision	Other considerations	Flavonoid	Placebo	Relative (95% CI)	Absolute (95% CI)		
**Outcome - SBP**												
11	randomised trials	not serious	serious[a]	not serious	serious[b]	none	345	339	-	MD **1.73 lower** (4.15 lower to 0.69 higher)	⨁⨁◯◯ Low	
**Outcome - DBP**												
11	randomised trials	not serious	serious[a]	not serious	serious[b]	none	345	340	-	MD **1.68 lower** (3.34 lower to 0.03 lower)	⨁⨁◯◯ Low	
**Outcome - HDL**												
6	randomised trials	not serious	serious[a]	not serious	serious[b]	none	167	163	-	MD **0.15 higher** (0.02 higher to 0.29 higher)	⨁⨁◯◯ Low	

Certainty assessment							№ of patients		Effect		Certainty	Importance
№ of studies	Study design	Risk of bias	Inconsistency	Indirectness	Imprecision	Other considerations	Flavonoid	Placebo	Relative (95% CI)	Absolute (95% CI)		
**Outcome - LDL**												
5	randomised trials	not serious	serious[a]	not serious	serious[b]	none	126	124	-	MD **0.32 lower** (0.61 lower to 0.04 lower)	⨁⨁◯◯ Low	
**Outcome - TAG**												
6	randomised trials	not serious	serious[a]	not serious	serious[b]	none	166	163	-	MD **0.7 lower** (1.37 lower to 0.03 lower)	⨁⨁◯◯ Low	
**Outcome - TC**												
6	randomised trials	not serious	serious[a]	not serious	serious[b]	none	166	139	-	MD **0.33 lower** (0.67 lower to 0.02 higher)	⨁⨁◯◯ Low	

CI: confidence interval; **MD:** mean difference

Explanations

a. Rated down for inconsistency.

b. Rated down for imprecision.

Table S2-7. GRADE profile for gallic acid supplementation.

Bibliography:

Certainty assessment							№ of patients		Effect		Certainty	Importance
№ of studies	Study design	Risk of bias	Inconsistency	Indirectness	Imprecision	Other considerations	gallic acid	Placebo	Relative (95% CI)	Absolute (95% CI)		
**CVD - SBP**												
5	randomised trials	not serious	serious[a]	not serious	serious[b]	none	139	140	-	MD **0.31 lower** (3.23 lower to 2.6 higher)	⨁⨁◯◯ Low	
**CVD - DBP**												

Certainty assessment							№ of patients		Effect		Certainty	Importance
№ of studies	Study design	Risk of bias	Inconsistency	Indirectness	Imprecision	Other considerations	gallic acid	Placebo	Relative (95% CI)	Absolute (95% CI)		
5	randomised trials	not serious	not serious	not serious	serious[b]	none	139	140	-	MD **1.16 lower** (2.4 lower to 0.07 higher)	⊕⊕⊕◯ Moderate	
**CVD - HDL**												
5	randomised trials	not serious	serious[a]	not serious	serious[b]	none	135	136	-	MD **0** (0.22 lower to 0.21 higher)	⊕⊕◯◯ Low	
**CVD - LDL**												
7	randomised trials	not serious	not serious	not serious	serious[b]	none	219	220	-	MD **0.06 higher** (0.1 lower to 0.22 higher)	⊕⊕⊕◯ Moderate	
**CVD - TG**												

Certainty assessment							№ of patients		Effect		Certainty	Importance
№ of studies	Study design	Risk of bias	Inconsistency	Indirectness	Imprecision	Other considerations	gallic acid	Placebo	Relative (95% CI)	Absolute (95% CI)		
7	randomised trials	not serious	not serious	not serious	serious[b]	none	219	220	-	MD **0.15 higher** (0.31 lower to 0.61 higher)	⨁⨁⨁◯ Moderate	
**CVD - TC**												
7	randomised trials	not serious	not serious	not serious	serious[b]	none	219	220	-	MD **0.06 higher** (0.18 lower to 0.3 higher)	⨁⨁⨁◯ Moderate	
**CVD - FBG**												
4	randomised trials	not serious	not serious	not serious	serious[b]	none	115	116	-	MD **0.08 higher** (0.01 lower to 0.18 higher)	⨁⨁⨁◯ Moderate	

CI: confidence interval; **MD:** mean difference

Explanations

a. Rated down for inconsistency.

b. Rate down for imprecision.

Table S2-8. GRADE profile for genistein supplementation.

Certainty assessment							№ of patients		Effect		Certainty	Importance
№ of studies	Study design	Risk of bias	Inconsistency	Indirectness	Imprecision	Other considerations	Genistein	Placebo	Relative (95% CI)	Absolute (95% CI)		
**Outcome - SBP**												
2	randomised trials	not serious	not serious	not serious	not serious	none	71	71	-	MD **10.02 lower** (11.55 lower to 8.49 lower)	⨁⨁⨁⨁ High	
**Outcome - DBP**												
2	randomised trials	not serious	not serious	not serious	not serious	none	71	71	-	MD **9.13 lower** (12.8 lower to 5.46 lower)	⨁⨁⨁⨁ High	

Certainty assessment							№ of patients		Effect		Certainty	Importance
№ of studies	Study design	Risk of bias	Inconsistency	Indirectness	Imprecision	Other considerations	Genistein	Placebo	Relative (95% CI)	Absolute (95% CI)		
**Outcome - HDL-C**												
10	randomised trials	not serious	serious[a]	not serious	serious[b]	none	656	630	-	MD **0.12 higher** (0.01 lower to 0.24 higher)	⨁⨁◯◯ Low	
**Outcome - LDL-C**												
9	randomised trials	not serious	serious[a]	not serious	serious[b]	none	580	589	-	MD **0.43 lower** (0.81 lower to 0.04 lower)	⨁⨁◯◯ Low	
**Outcome - TAG**												
10	randomised trials	not serious	not serious	not serious	serious[b]	none	656	660	-	MD **0.1 lower** (0.32 lower to 0.11 higher)	⨁⨁⨁◯ Moderate	

Certainty assessment							№ of patients		Effect		Certainty	Importance
№ of studies	Study design	Risk of bias	Inconsistency	Indirectness	Imprecision	Other considerations	Genistein	Placebo	Relative (95% CI)	Absolute (95% CI)		
**Outcome - TC**												
9	randomised trials	not serious	not serious	not serious	serious[b]	none	589	589	-	MD **0.22 lower** (0.35 lower to 0.08 lower)	⨁⨁⨁◯ Moderate	
**Outcome - FBS**												
7	randomised trials	not serious	serious[a]	not serious	serious[b]	none	410	426	-	MD **0.44 lower** (0.52 lower to 0.37 lower)	⨁⨁◯◯ Low	
**Outcome - Insulin**												
7	randomised trials	not serious	serious[a]	not serious	not serious	none	414	422	-	MD **11.61 lower** (15.4 lower to 7.82 lower)	⨁⨁⨁◯ Moderate	

CI: confidence interval; **MD:** mean difference

Explanations

a. Rated down for inconsistency.

b. Rated down for imprecision.

Table S2-9. GRADE profile for hesperidin supplementation.

Certainty assessment							№ of patients		Effect		Certainty	Importance
№ of studies	Study design	Risk of bias	Inconsistency	Indirectness	Imprecision	Other considerations	Hesperidin	Placebo	Relative (95% CI)	Absolute (95% CI)		
**Outcome - SBP**												
5	randomised trials	not serious	not serious	not serious	serious[a]	none	137	128	-	MD **1.01 higher** (0.7 lower to 2.72 higher)	⨁⨁⨁◯ Moderate	
**Outcome - DBP**												

Certainty assessment							№ of patients		Effect		Certainty	Importance
№ of studies	Study design	Risk of bias	Inconsistency	Indirectness	Imprecision	Other considerations	Hesperidin	Placebo	Relative (95% CI)	Absolute (95% CI)		
4	randomised trials	not serious	not serious	not serious	serious[a]	none	125	116	-	MD **1.39 lower** (3.92 lower to 1.14 higher)	⊕⊕⊕◯ Moderate	

Outcome - HDL

5	randomised trials	not serious	not serious	not serious	serious[a]	none	137	127	-	MD **0.04 higher** (0.11 lower to 0.19 higher)	⊕⊕⊕◯ Moderate	

Outcome - LDL

6	randomised trials	not serious	not serious	not serious	serious[a]	none	150	139	-	MD **0.15 lower** (0.3 lower to 0.01 higher)	⊕⊕⊕◯ Moderate	

Outcome - TAG

Certainty assessment							№ of patients		Effect		Certainty	Importance
№ of studies	Study design	Risk of bias	Inconsistency	Indirectness	Imprecision	Other considerations	Hesperidin	Placebo	Relative (95% CI)	Absolute (95% CI)		
6	randomised trials	not serious	not serious	not serious	serious[a]	none	150	139	-	MD **0.26 lower** (0.59 lower to 0.07 higher)	⨁⨁⨁◯ Moderate	
**Outcome - TC**												
6	randomised trials	not serious	not serious	not serious	serious[a]	none	149	139	-	MD **0.24 lower** (0.5 lower to 0.02 higher)	⨁⨁⨁◯ Moderate	
**Outcome - FBS**												
6	randomised trials	not serious	not serious	not serious	serious[a]	none	168	157	-	MD **0.01 higher** (0.12 lower to 0.14 higher)	⨁⨁⨁◯ Moderate	
**Outcome - Insulin**												

Certainty assessment							№ of patients		Effect		Certainty	Importance
№ of studies	Study design	Risk of bias	Inconsistency	Indirectness	Imprecision	Other considerations	Hesperidin	Placebo	Relative (95% CI)	Absolute (95% CI)		
5	randomised trials	not serious	not serious	not serious	serious[a]	none	137	128	-	MD **0.63 higher** (5.06 lower to 6.32 higher)	⨁⨁⨁◯ Moderate	

CI: confidence interval; **MD:** mean difference

Explanations

a. Rated down for imprecision.

Table S2-10. GRADE profile for isoflavone supplementation.

Certainty assessment							№ of patients		Effect		Certainty	Importance
№ of studies	Study design	Risk of bias	Inconsistency	Indirectness	Imprecision	Other considerations	Isoflavones	Placebo	Relative (95% CI)	Absolute (95% CI)		

Outcome - SBP

Certainty assessment							№ of patients		Effect		Certainty	Importance
№ of studies	Study design	Risk of bias	Inconsistency	Indirectness	Imprecision	Other considerations	Isoflavones	Placebo	Relative (95% CI)	Absolute (95% CI)		
24	randomised trials	not serious	not serious	not serious	serious[a]	none	898	901	-	MD **0.87 lower** (2.32 lower to 0.59 higher)	⊕⊕⊕◯ Moderate	
**Outcome - DBP**												
24	randomised trials	not serious	not serious	not serious	serious[a]	none	944	944	-	MD **0.54 lower** (1.47 lower to 0.4 higher)	⊕⊕⊕◯ Moderate	
**Outcome - HDL**												
26	randomised trials	not serious	not serious	not serious	serious[a]	none	1014	1036	-	MD **0.02 lower** (0.08 lower to 0.04 higher)	⊕⊕⊕◯ Moderate	
**Outcome - LDL**												

Certainty assessment							№ of patients		Effect		Certainty	Importance
№ of studies	Study design	Risk of bias	Inconsistency	Indirectness	Imprecision	Other considerations	Isoflavones	Placebo	Relative (95% CI)	Absolute (95% CI)		
25	randomised trials	not serious	not serious	not serious	serious[a]	none	987	1011	-	MD **0.1 higher** (0.15 lower to 0.35 higher)	⊕⊕⊕◯ Moderate	
**Outcome - TAG**												
25	randomised trials	not serious	not serious	not serious	serious[a]	none	988	1012	-	MD **0.05 lower** (0.12 lower to 0.02 higher)	⊕⊕⊕◯ Moderate	
**Outcome - TC**												
24	randomised trials	not serious	serious[b]	not serious	serious[a]	none	904	924	-	MD **0.14 lower** (0.31 lower to 0.02 higher)	⊕⊕◯◯ Low	

Outcome - FBS

Certainty assessment							№ of patients		Effect		Certainty	Importance
№ of studies	Study design	Risk of bias	Inconsistency	Indirectness	Imprecision	Other considerations	Isoflavones	Placebo	Relative (95% CI)	Absolute (95% CI)		
12	randomised trials	not serious	not serious	not serious	serious[a]	none	496	520	-	MD **0.05 lower** (0.24 lower to 0.13 higher)	⊕⊕⊕◯ Moderate	
**Outcome - Insulin**												
11	randomised trials	not serious	not serious	not serious	serious[a]	none	475	500	-	MD **1.63 lower** (10.61 lower to 7.35 higher)	⊕⊕⊕◯ Moderate	
**Outcome - HbA1c**												
5	randomised trials	not serious	not serious	not serious	serious[a]	none	73	74	-	MD **0** (0.25 lower to 0.24 higher)	⊕⊕⊕◯ Moderate	

CI: confidence interval; **MD:** mean difference

Explanations

a. Rated down for imprecision.

b. Rated down for inconsistency.

Table S2-11. GRADE profile for quercetin supplementation.

Certainty assessment							№ of patients		Effect		Certainty	Importance
№ of studies	Study design	Risk of bias	Inconsistency	Indirectness	Imprecision	Other considerations	Quercetin	Placebo	Relative (95% CI)	Absolute (95% CI)		
**Outcome - SBP**												
13	randomised trials	not serious	not serious	not serious	serious[a]	none	300	298	-	MD **1.38 lower** (2.63 lower to 0.13 lower)	⊕⊕⊕◯ Moderate	
**Outcome - DBP**												
13	randomised trials	not serious	not serious	not serious	serious[a]	none	300	298	-	MD **1.11 lower** (2.25 lower to 0.02 higher)	⊕⊕⊕◯ Moderate	

Certainty assessment							№ of patients		Effect		Certainty	Importance
№ of studies	Study design	Risk of bias	Inconsistency	Indirectness	Imprecision	Other considerations	Quercetin	Placebo	Relative (95% CI)	Absolute (95% CI)		
**Outcome - HDL**												
13	randomised trials	not serious	not serious	not serious	serious[a]	none	505	499	-	MD **0.03 higher** (0.03 lower to 0.09 higher)	⨁⨁⨁◯ Moderate	
**Outcome - LDL**												
13	randomised trials	not serious	not serious	not serious	serious[a]	none	505	499	-	MD **0.03 lower** (0.14 lower to 0.08 higher)	⨁⨁⨁◯ Moderate	
**Outcome - TAG**												
13	randomised trials	not serious	not serious	not serious	serious[a]	none	505	499	-	MD **0.06 lower** (0.21 lower to 0.08 higher)	⨁⨁⨁◯ Moderate	

Certainty assessment							№ of patients		Effect		Certainty	Importance
№ of studies	Study design	Risk of bias	Inconsistency	Indirectness	Imprecision	Other considerations	Quercetin	Placebo	Relative (95% CI)	Absolute (95% CI)		
**Outcome - TC**												
13	randomised trials	not serious	not serious	not serious	serious[a]	none	505	499	-	MD **0.04 lower** (0.17 lower to 0.08 higher)	⨁⨁⨁◯ Moderate	
**Outcome - FBS**												
10	randomised trials	not serious	not serious	not serious	serious[a]	none	330	324	-	MD **0.02 lower** (0.14 lower to 0.09 higher)	⨁⨁⨁◯ Moderate	
**Outcome - Insulin**												
3	randomised trials	not serious	not serious	not serious	serious[a]	none	115	115	-	MD **8.09 lower** (15.53 lower to 0.66 lower)	⨁⨁⨁◯ Moderate	

CI: confidence interval; **MD:** mean difference

Explanations

a. Rated down for imprecision.

Table S2-12. GRADE profile for resveratrol supplementation.

Certainty assessment							№ of patients		Effect		Certainty	Importance
№ of studies	Study design	Risk of bias	Inconsistency	Indirectness	Imprecision	Other considerations	Resveratrol	Placebo	Relative (95% CI)	Absolute (95% CI)		
**Outcome - SBP**												
19	randomised trials	not serious	serious[a]	not serious	serious[b]	none	467	469	-	MD **3.25 lower** (6.03 lower to 0.48 lower)	⨁⨁◯◯ Low	
**Outcome - DBP**												

Certainty assessment							№ of patients		Effect		Certainty	Importance
№ of studies	Study design	Risk of bias	Inconsistency	Indirectness	Imprecision	Other considerations	Resveratrol	Placebo	Relative (95% CI)	Absolute (95% CI)		
19	randomised trials	not serious	serious[a]	not serious	serious[b]	none	467	469	-	MD **2.32 lower** (4.07 lower to 0.57 lower)	⊕⊕◯◯ Low	

Outcome - HDL

15	randomised trials	not serious	not serious	not serious	serious[b]	none	412	410	-	MD **0.02 lower** (0.06 lower to 0.03 higher)	⊕⊕⊕◯ Moderate	

Outcome - LDL

14	randomised trials	not serious	serious[a]	not serious	serious[b]	none	386	385	-	MD **0.19 higher** (0.22 lower to 0.61 higher)	⊕⊕◯◯ Low	

Outcome - TAG

Certainty assessment							№ of patients		Effect		Certainty	Importance
№ of studies	Study design	Risk of bias	Inconsistency	Indirectness	Imprecision	Other considerations	Resveratrol	Placebo	Relative (95% CI)	Absolute (95% CI)		
18	randomised trials	not serious	not serious	not serious	serious[b]	none	458	467	-	MD **0.06 lower** (0.28 lower to 0.17 higher)	⊕⊕⊕◯ Moderate	

Outcome - TC

16	randomised trials	not serious	serious[a]	not serious	serious[b]	none	435	433	-	MD **0.09 lower** (0.33 lower to 0.15 higher)	⊕⊕◯◯ Low	

Outcome - FBS

23	randomised trials	not serious	serious[a]	not serious	serious[b]	none	581	592	-	MD **2.31 lower** (4.86 lower to 0.24 higher)	⊕⊕◯◯ Low	

Outcome - Insulin

Certainty assessment							№ of patients		Effect		Certainty	Importance
№ of studies	Study design	Risk of bias	Inconsistency	Indirectness	Imprecision	Other considerations	Resveratrol	Placebo	Relative (95% CI)	Absolute (95% CI)		
18	randomised trials	not serious	serious[a]	not serious	serious[b]	none	444	452	-	MD **2.84 lower** (5.61 lower to 0.06 lower)	⊕⊕◯◯ Low	
**Outcome - HbA1c**												
12	randomised trials	not serious	serious[a]	not serious	serious[b]	none	314	315	-	MD **0.06 lower** (0.16 lower to 0.05 higher)	⊕⊕◯◯ Low	

CI: confidence interval; **MD:** mean difference

Explanations

a. Rated down for inconsistency.

b. Rate down for imprecision.

Appendix 6 - Results of sensitivity analysis of the systematic removal of each study

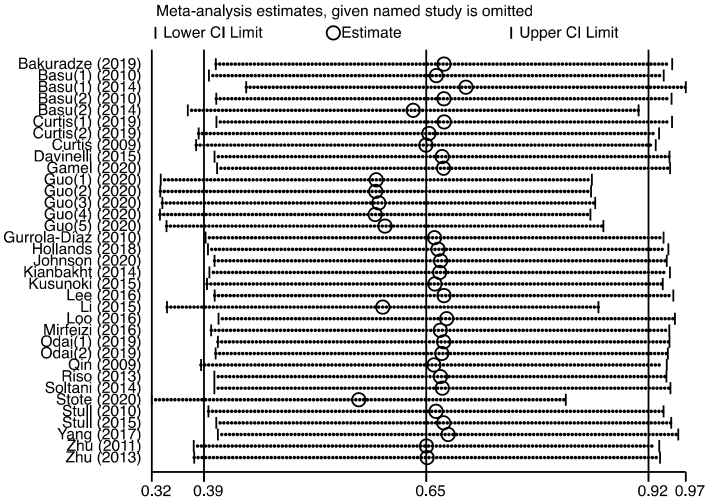

Figure S4-1 Sensitivity analysis of the systematic removal of each study for HDL-C in participants supplemented with anthocyanin.

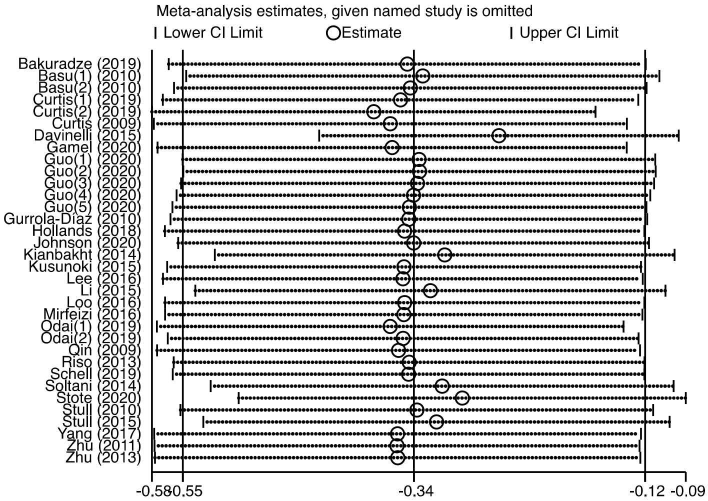

Figure S4-2 Sensitivity analysis of the systematic removal of each study for TG in participants supplemented with anthocyanin.

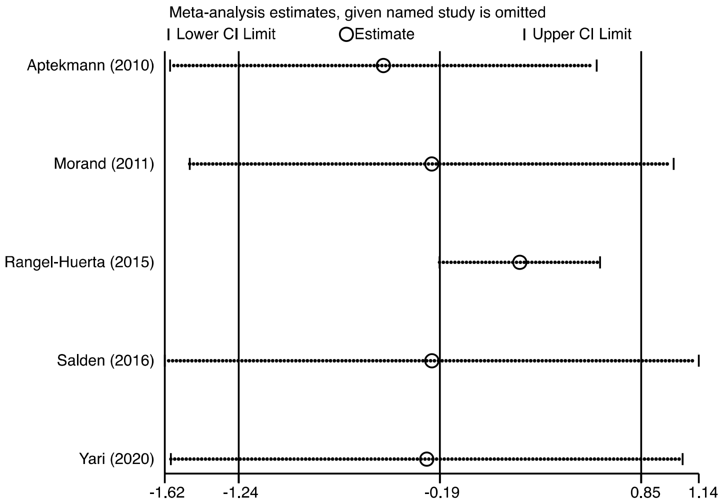

Figure S4-3 Sensitivity analysis of the systematic removal of each study for LDL-C in participants supplemented with hesperidin.
